# Interventions to improve outdoor mobility among people living with disabilities: A systematic review

**DOI:** 10.1002/cl2.1407

**Published:** 2024-06-14

**Authors:** Martin Ringsten, Branimir Ivanic, Susanne Iwarsson, Eva Månsson Lexell

**Affiliations:** ^1^ Cochrane Sweden, Research and Development Skåne University Hospital Lund Sweden; ^2^ Department of Health Sciences Lund University Lund Sweden; ^3^ Department of Neurology, Rehabilitation Medicine, Cognitive Medicine and Geriatrics Skåne University Hospital Lund‐Malmö Sweden

## Abstract

**Background:**

Around 15% of the global population live with some form of disabilities and experience worse health outcomes, less participation in the community and are part of fewer activities outside the home. Outdoor mobility interventions aim to improve the ability to move, travel and orient outside the home and could influence the number of activities outside the home, participation and quality of life. However, outdoor mobility interventions may also lead to harm like falls or injuries or have unforeseen effects which could lead to mortality or hospitalization.

**Objectives:**

To assess the efficacy of interventions aiming to improve outdoor mobility for adults living with disabilities and to explore if the efficacy varies between different conditions and different intervention components.

**Search Methods:**

Standard, extensive Campbell search methods were used, including a total of 12 databases searched during January 2023, including trial registries.

**Selection Criteria:**

Only randomized controlled trials were included, focusing on people living with disabilities, comparing interventions to improve outdoor mobility to control interventions as well as comparing different types of interventions to improve outdoor mobility.

**Data Collection and Analysis:**

Standard methodological procedures expected by Campbell were used. The following important outcomes were 1. Activity outside the home; 2. Engagement in everyday life activities; 3. Participation; 4. Health‐related Quality of Life; 5. Major harms; 6. Minor harms. The impact of the interventions was evaluated in the shorter (≤6 months) and longer term (≥7 months) after starting the intervention. Results are presented using risk ratios (RR), risk difference (RD), and standardized mean differences (SMD), with the associated confidence intervals (CI). The risk of bias 2‐tool and the GRADE‐framework were used to assess the certainty of the evidence.

**Main Results:**

The screening comprised of 12.894 studies and included 22 studies involving 2.675 people living with disabilities and identified 12 ongoing studies. All reported outcomes except one (reported in one study, some concerns of bias) had overall high risk of bias. Thirteen studies were conducted in participants with disabilities due to stroke, five studies with older adults living with disabilities, two studies with wheelchair users, one study in participants with disabilities after a hip fracture, and one study in participants with cognitive impairments.

**Skill training interventions versus control interventions (16 studies)**

The evidence is very uncertain about the benefits and harms of skill training interventions versus control interventions not aimed to improve outdoor mobility among all people living with disabilities both in the shorter term (≤6 months) and longer term (≥7 months) for Activity outside the home; Participation; Health‐related Quality of Life; Major harms; and Minor harms, based on very low certainty evidence. Skill training interventions may improve engagement in everyday life activities among people with disabilities in the shorter term (RR: 1.46; 95% CI: 1.16 to 1.84; *I* ^2^ = 7%; RD: 0.15; 95% CI: −0.02 to 0.32; *I* ^2^ = 71%; 692 participants; three studies; low certainty evidence), but the evidence is very uncertain in the longer term, based on very low certainty evidence. Subgroup analysis of skill training interventions among people living with disabilities due to cognitive impairments suggests that such interventions may improve activity outside the home in the shorter term (SMD: 0.44; 95% CI: 0.07 to 0.81; *I* ^2^ = NA; 118 participants; one study; low certainty evidence). Subgroup analysis of skill training interventions among people living with cognitive impairments suggests that such interventions may improve health‐related quality of life in the shorter term (SMD: 0.49; 95% CI: 0.12 to 0.88; *I* ^2^ = NA; 118 participants; one study; low certainty evidence).

**Physical training interventions versus control interventions (five studies)**

The evidence is very uncertain about the benefits and harms of physical training interventions versus control interventions not aimed to improve outdoor mobility in the shorter term (≤6 months) and longer term (≥7 months) for: Engagement in everyday life activities; Participation; Health‐related Quality of Life; Major harms; and Minor harms, based on very low certainty evidence. Physical training interventions may improve activity outside the home in the shorter (SMD: 0.35; 95% CI: 0.08 to 0.61; *I* ^2^ = NA; 228 participants; one study; low certainty evidence) and longer term (≥7 months) (SMD: 0.27; 95% CI: 0.00 to 0.54; *I* ^2^ = NA; 216 participants; one study; low certainty evidence).

**Comparison of different outdoor mobility interventions (one study)**

The evidence is very uncertain about the benefits and harms of outdoor mobility interventions of different lengths in the shorter term (≤6 months) and longer term (≥7 months) for Activity outside the home; Engagement in everyday life activities; Participation; Health‐related Quality of Life; Major harms; and Minor harms, based on very low certainty evidence. No studies explored the efficacy of other types of interventions.

**Authors’ Conclusions:**

Twenty‐two studies of interventions to improve outdoor mobility for people living with disabilities were identified, but the evidence still remains uncertain about most benefits and harms of these interventions, both in the short‐ and long term. This is primarily related to risk of bias, small underpowered studies and limited reporting of important outcomes for people living with disabilities. For people with disabilities, skill training interventions may improve engagement in everyday life in the short term, and improve activity outside the home and health‐related quality of life for people with cognitive impairments in the short term. Still, this is based on low certainty evidence from few studies and should be interpreted with caution. One study with low certainty evidence suggests that physical training interventions may improve activity outside the home in the short term. In addition, the effect sizes across all outcomes were considered small or trivial, and could be of limited relevance to people living with disabilities. The evidence is currently uncertain if there are interventions that can improve outdoor mobility for people with disabilities, and can improve other important outcomes, while avoiding harms. To guide decisions about the use of interventions to improve outdoor mobility, future studies should use more rigorous design and report important outcomes for people with disabilities to reduce the current uncertainty.

## 1 PLAIN LANGUAGE SUMMARY

### 1.1 Limited evidence of benefits and harms for interventions to improve outdoor mobility for people living with disabilities

#### 1.1.1 The review in brief

The evidence is very uncertain about most benefits and harms of intervention options aimed to improve outdoor mobility for adults with disabilities, both in the short and long term, and it is currently unclear if any intervention options to improve outdoor mobility are effective and safe.

#### 1.1.2 What is this review about?

Around 15% of the global population live with disabilities and participate in fewer activities outside the home and in the community, and commonly also experience a reduced quality of life. Interventions in this review target outdoor mobility aiming to improve the ability to move, travel and manage orientation outside the home and in the community. These interventions can influence the number of activities outside the home, participation and quality of life but may also lead to harm like falls, injuries and hospitalization.

#### 1.1.3 What is the aim of this review?

The review examines the benefits and harms of interventions aimed to improve outdoor mobility for adults living with different disabilities.

#### 1.1.4 What are the main findings of this review?

***What studies are included?***

A total of 12,894 studies were reviewed. Twenty‐two studies that evaluated interventions aiming to improve outdoor mobility compared to interventions that did not aim to improve outdoor mobility were identified. Twelve ongoing studies with upcoming results were also located. Sixteen studies examined interventions where skills aimed to be used in the outdoor environment were trained. Five studies examined physical training aiming to improve the capacity to move around in outdoor environments, such as treadmill walking. No studies were identified for other types of interventions, such as educational or cognitive behavioural interventions. Most interventions were delivered to people after stroke (13 studies) and older adults living with disabilities (five studies) and were delivered mainly in high income countries.

***What are the benefits and harms of skill training?***

The evidence is uncertain about the benefits and harms of skill training for all people living with disabilities, both in the shorter and longer term for the possibility to be active outside the home, participation, quality of life and for minor and major harms.

Skill training may improve engagement in everyday life activities in people living with disabilities in the shorter term, but we are uncertain in the longer term. Skill training may improve both activity outside the home and health‐related quality of life for people living with cognitive impairments in the shorter term, but we are uncertain in the longer term.

***What are the benefits and harms of physical training?***

The evidence is uncertain about the benefits and harms of physical training for all people living with disabilities, both in the shorter and longer term for the possibility to be engaged in everyday life activities, participation, quality of life and for minor and major harms. Physical training may improve activity outside the home in people living with disabilities in the shorter term, but we are uncertain in the longer term.

***What are the benefits and harms of different treatment options aimed to improve outdoor mobility?***

The evidence is uncertain about any difference for benefits and harms of different outdoor mobility treatment options.

#### 1.1.5 What do the findings of this review mean?

Despite identifying 22 studies, the evidence still remains uncertain about most benefits and harms of treatments to improve outdoor mobility, both in the short‐ and long term. Furthermore, the reported effects of these interventions were considered either small or trivial, and could be of limited relevance to people living with disabilities. The evidence is currently uncertain if there are interventions that can improve outdoor mobility for people living with disabilities and can improve other important outcomes, while avoiding harms.

Future studies that are using more rigorous study designs and include important measurements of improvement for people living with disabilities are needed to reduce this uncertainty to guide policy decisions, treatment decisions of healthcare providers and carers, and decisions to engage in interventions to improve outdoor mobility for people living with disabilities.

#### 1.1.6 How up‐to‐date is this review?

The evidence is up to date covering publications until January 2023.

## 2 SUMMARY OF FINDINGS

Summary of findings 1, 2, 3, 4

**Table 1:** Summary of findings: Skill training in the shorter term (≤6 months).

Outcome № of participants (studies)	Relative effect (95% CI)	Anticipated absolute effects (95% CI)			Certainty	What happens
		Without intervention	With skill training intervention	Difference		
Activity outside the home ≤6 months № of participants: 925 (6 RCTs)	–	–	–	SMD **0.18 SD higher** (0.2 lower to 0.56 higher)	⨁◯◯◯ Very low^a^, ^b^, ^c^	The evidence is very uncertain about the effect of skill training interventions to improve outdoor mobility on activity outside the home ≤6 months.
Engagement in everyday life activities ≤6 months № of participants: 692 (3 RCTs)	**RR 1.46** (1.16 to 1.84)	26.4%	**38.6%** (30.7 to 48.7)	**12.2% more** (4.2 more to 22.2 more)	⨁⨁◯◯ Low^g^	Skill training interventions to improve outdoor mobility may increase engagement in everyday life activities ≤6 months.
Participation ≤6 months № of participants: 886 (7 RCTs)	–	–	–	SMD **0.01 SD higher** (0.23 lower to 0.25 higher)	⨁◯◯◯ Very low^a^, ^b^, ^d^	The evidence is very uncertain about the effect of skill training interventions to improve outdoor mobility on participation ≤6 months.
Health‐related quality of life ≤6 months № of participants: 779 (4 RCTs)	–	–	–	SMD **0.13 SD higher** (0.2 lower to 0.46 higher)	⨁◯◯◯ Very low^c^, ^e^, ^f^	The evidence is very uncertain about the effect of skill training interventions to improve outdoor mobility on health‐related quality of life ≤6 months.
Major harms ≤6 months № of participants: 99 (2 RCTs)	**RR 0.83** (0.27 to 2.54)	12.0%	**10.0%** (3.2 to 30.5)	**2.0% fewer** (8.8 fewer to 18.5 more)	⨁◯◯◯ Very low^a^, ^c^	The evidence is very uncertain about the effect of skill training interventions to improve outdoor mobility on major harms ≤6 months.
Minor harms ≤6 months № of participants: 124 (2 RCTs)	**RR 1.14** (0.63 to 2.04)	24.6%	**28.0%** (15.5 to 50.2)	**3.4% more** (9.1 fewer to 25.6 more)	⨁◯◯◯ Very low^a^, ^c^	The evidence is very uncertain about the effect of skill training interventions to improve outdoor mobility on minor harms ≤6 months.

^a^ Downgraded one level for very serious risk of bias in all included studies.

^b^ Downgraded one level due to very serious inconsistency due to very heterogeneous effects without any clear reasons.

^c^ Downgraded one level due to very serious imprecision due to large confidence intervals containing both large benefits, no benefits and harms.

^d^ Downgraded one level due to serious imprecision due to confidence intervals containing both some benefits, no benefits and some harms.

^e^ Downgraded one level for serious risk of bias in all included studies.

^f^ Downgraded one level due to serious inconsistency due to heterogeneous effects without any clear reasons.

^g^ Downgraded two levels for very serious risk of bias in all included studies.

**Table 2:** Summary of findings: Skill training in the longer term (≥7 months).

Outcome № of participants (studies)	Relative effect (95% CI)	Anticipated absolute effects (95% CI)			Certainty	What happens
		Without intervention	With skill training intervention	Difference		
Activity outside the home ≥7 months № of participants: 672 (2 RCTs)	–	–	–	SMD **0.38 SD higher** (0.55 lower to 1.3 higher)	⨁◯◯◯ Very low^a^, ^b^, ^c^	The evidence is very uncertain about the effect of skill training interventions to improve outdoor mobility on activity outside the home ≥7 months.
Engagement in everyday life activities ≥7 months № of participants: 600 (2 RCTs)	**RR 1.40** (0.91 to 2.15)	25.8%	**36.1%** (23.5 to 55.4)	**10.3% more** (2.3 fewer to 29.7 more)	⨁◯◯◯ Very low^a^, ^b^, ^c^	The evidence is very uncertain about the effect of skill training interventions to improve engagement in everyday life activities ≥7 months.
Participation ≥7 months № of participants: 674 (3 RCTs)	–	–	–	SMD **0.05 SD lower** (0.31 lower to 0.2 higher)	⨁◯◯◯ Very low^a^, ^d^, ^e^	The evidence is very uncertain about the effect of skill training interventions to improve outdoor mobility on participation ≥7 months.
Health‐related quality of life ≥7 months № of participants: 495 (2 RCTs)	–	–	–	SMD **0.05 SD lower** (0.23 lower to 0.13 higher)	⨁◯◯◯ Very low^e^, ^f^	The evidence is very uncertain about the effect of skill training interventions to improve outdoor mobility on health‐related quality of life ≥7 months.
Major harms ≥7 months № of participants: 568 (1 RCT)	**RR 0.98** (0.45 to 2.14)	4.3%	**4.2%** (1.9 to 9.1)	**0.1% fewer** (2.3 fewer to 4.9 more)	⨁◯◯◯ Very low^c^, ^f^	The evidence is very uncertain about the effect of skill training interventions to improve outdoor mobility on major harms ≥7 months.
Minor harms ≥7 months № of participants: 627 (2 RCTs)	**RR 1.00** (0.84 to 1.18)	45.8%	**45.8%** (38.5 to 54)	**0.0% fewer** (7.3 fewer to 8.2 more)	⨁◯◯◯ Very low^e^, ^f^	The evidence is very uncertain about the effect of skill training interventions to improve outdoor mobility on minor harms ≥7months.

^a^ Downgraded one level for very serious risk of bias in all included studies.

^b^ Downgraded one level due to very serious inconsistency due to very heterogeneous effects without any clear reasons why.

^c^ Downgraded one level due to very serious imprecision due to large confidence intervals containing both large benefits, no benefits and harms.

^d^ Downgraded one level due to serious inconsistency due to heterogeneous effects without any clear reasons why.

^e^ Downgraded one level due to serious imprecision due to confidence intervals containing both some benefits, no benefits and some harms.

^f^ Downgraded two levels for very serious risk of bias in all included studies.

**Table 3:** Summary of findings: Physical training in the shorter term (≤6 months).

Outcome № of participants (studies)	Relative effect (95% CI)	Anticipated absolute effects (95% CI)			Certainty	What happens
		Without intervention	With skill training intervention	Difference		
Activity outside the home ≤6 months № of participants: 228 (1 RCT)	–	–	–	SMD **0.35 SD higher** (0.08 higher to 0.61 higher)	⨁⨁◯◯ Low^a^	Physical training interventions to improve outdoor mobility may increase activity outside the home ≤6 months.
Engagement in everyday life activities ≤6 months № of participants: 337 (2 RCTs)	**RR 1.01** (0.79 to 0.29)	42.4%	**42.8%** (12.3 to 33.5)	**0.4% more** (30.1 fewer to 8.9 fewer)	⨁◯◯◯ Very low^a^, ^b^	The evidence is very uncertain about the effect of physical training interventions to improve outdoor mobility on engagement in everyday life activities ≤6 months.
Participation ≤6 months № of participants: 228 (1 RCT)	–	–	–	SMD **0.12 SD higher** (0.14 lower to 0.38 higher)	⨁◯◯◯ Very low^a^, ^b^	The evidence is very uncertain about the effect of physical training interventions to improve outdoor mobility on participation ≤6 months.
Health‐related quality of life ≤6 months № of participants: 225 (1 RCT)	–	–	–	SMD **0.02 SD higher** (0.25 lower to 0.28 higher)	⨁◯◯◯ Very low^a^, ^b^	The evidence is very uncertain about the effect of physical training interventions to improve outdoor mobility on health‐related quality of life ≤6 months.
Major harms ≤6 months № of participants: 319 (2 RCTs)	**RR 0.63** (0.20 to 2.03)	11.4%	**7.2%** (2.3 to 23.1)	**4.2% fewer** (9.1 fewer to 11.7 more)	⨁◯◯◯ Very low^a^^,^ b ^,^ c	The evidence is very uncertain about the effect of physical training interventions to improve outdoor mobility on major harms ≤6 months.
Minor harms ≤6 months № of participants: 319 (2 RCTs)	**RR 0.81** (0.56 to 1.16)	24.1%	**19.5%** (13.5 to 27.9)	**4.6% fewer** (10.6 fewer to 3.8 more)	⨁◯◯◯ Very low^a^, ^b^	The evidence is very uncertain about the effect of physical training interventions to improve outdoor mobility on minor harms ≤6 months.

^a^ Downgraded two levels for very serious risk of bias in all included studies.

^b^ Downgraded one level due to serious imprecision due to confidence intervals containing both some benefits, no benefits and some harms.

^c^ Downgraded one level due to serious inconsistency due to heterogeneous effects without any clear reasons why.

**Table 4:** Summary of findings: Physical training in the longer term (≥7 months).

Outcome № of participants (studies)	Relative effect (95% CI)	Anticipated absolute effects (95% CI)			Certainty	What happens
		Without intervention	With skill training intervention	Difference		
Activity outside the home ≥7 months № of participants: 216 (1 RCT)	‐	‐	‐	SMD **0.27 SD higher** (0 to 0.54 higher)	⨁⨁◯◯ Low^a^	Physical training interventions to improve outdoor mobility may increase activity outside the home ≥7 months.
Engagement in everyday life activities ≥7 months № of participants: 313 (2 RCTs)	**RR 0.87** (0.67 to 1.13)	44.2%	**38.4%** (29.6 to 49.9)	**5.7% fewer** (14.6 fewer to 5.7 more)	⨁◯◯◯ Very low^a^, ^b^	The evidence is very uncertain about the effect of physical training interventions to improve outdoor mobility on engagement in everyday life activities ≥7 months.
Participation ≥7 months № of participants: 216 (1 RCT)	‐	‐	‐	SMD **0.06 SD higher** (0.21 lower to 0.32 higher)	⨁◯◯◯ Very low^a^, ^b^	The evidence is very uncertain about the effect of physical training interventions to improve outdoor mobility on participation ≥7 months.
Health‐related quality of life ≥7 months № of participants: 215 (1 RCT)	‐	‐	‐	SMD **0.01 SD lower** (0.28 lower to 0.26 higher)	⨁◯◯◯ Very low^a^, ^b^	The evidence is very uncertain about the effect of physical training interventions to improve outdoor mobility on health‐related quality of life ≥7 months.
Major harms ≥7 months № of participants: 338 (2 RCTs)	**RR 0.96** (0.57 to 1.60)	46.4%	**44.5%** (26.4 to 74.2)	**1.9% fewer** (19.9 fewer to 27.8 more)	⨁◯◯◯ Very low^a^, ^b^	The evidence is very uncertain about the effect of physical training interventions to improve outdoor mobility on major harms ≥7 months.
Minor harms ≥7 months № of participants: 335 (2 RCTs)	**RR 1.04** (0.84 to 1.26)	53.7%	**55.8%** (45.1 to 67.6)	**2.1% more** (8.6 fewer to 14 more)	⨁◯◯◯ Very low^a^, ^b^	The evidence is very uncertain about the effect of physical training interventions to improve outdoor mobility on minor harms ≥7 months.

^a^ Downgraded two levels for very serious risk of bias in all included studies.

^b^ Downgraded one level due to serious imprecision due to confidence intervals containing both some benefits, no benefits and some harms.

## 3 BACKGROUND

### 3.1 The problem, condition or issue

Around 15% of the global population, which accounts for around one billion people, live with some form of disability and the number is increasing (WHO, 2020). The International Classification of Functioning, Disability and Health (ICF) is an international conceptual framework used to describe and measure health and disability on both the individual and population level (ICF). Disability, according to the ICF terminology, is the term for any impairments, activity limitations or participation restrictions and includes all traumatic, congenital and acquired conditions. The ICF focuses on health and the consequences of disease, and is closely related to the International Statistical Classification of Diseases and Related Health Problems (ICD‐10), a framework that describes and classifies diseases, disorders and other health conditions by diagnosis (ICD‐10). The definition of disability used in the ICF includes many potential causes of disability. The causes of disability are often multifactorial and can include, but are not limited to, impairments caused by specific diseases or disorders, the most immediate as well as general environment, and the interaction between the person and the environment.

People living with disabilities can experience worse health outcomes (WHO, 2020), experience less participation in the community as well as be part of fewer activities outside the home (Eurostat, 2015). For example, a systematic review exploring un‐met long‐term needs reported that 74% of people after stroke perceive at least one or more unmet needs in their life (Chen, 2019). Moreover, people with disability due to dementia have reported decreased out‐of‐home participation and life space outside the home (Margot‐Cattin, 2021).

There are several international and national strategies and policies to guide initiatives to reduce the prevalence and impact of living with disabilities. For example, the United Nation's Convention on the Rights of Persons with Disabilities (CRPD, 2006) and the European Disability Strategy (EU, 2010). In addition, the EU priority areas for people living with disabilities lists essential areas and goals for further initiatives for people living with disabilities. This includes accessibility to goods and services, participation in public life, activities and community services, and equality to combat discrimination and promote equal opportunities.

People living with disabilities need to be able to be mobile outside the home to reach places and destinations that are important to them. Being able to move outdoors can improve participation and valuable activities and being familiar with territories outdoors can support people living with disabilities to be mobile outside the home. Outdoor mobility includes any way of transportation and mobility in the outdoor environment. This can include using your own body for transportation, such as walking or using vehicles for transportation, like cars, buses, trains, trams, bicycles or scooters (Mollenkopf, 2006). For people living with disabilities, using different mobility devices such as a rollator or a manual/electric wheelchair can also facilitate outdoor mobility. Outdoor mobility is necessary to access commodities like grocery stores, pharmacies or healthcare and to participate in social, physical or other meaningful activities in the community (Rantanen, 2013).

Many people living with disabilities experience limitations related to mobility outside the home (Sainio, 2006). Limitations in outdoor mobility can be viewed as a result of the complex transaction between the person, his or her activities and the environment where activities take place. That is, outdoor mobility can be limited by personal factors that can decrease the capacity to be mobile outside the home, and impact the performance in the current outdoor environment. This can be due to physical, cognitive and mental impairments caused by, for example injuries, specific diseases, conditions, or processes associated with ageing. Reflecting on environmental factors, limitations in outdoor mobility can also arise when the demands of the environment exceed the capacity of the person. Stairs, long distances to geographical locations, or insufficient information to navigate are examples of such demands. The possibility to be mobile outdoors is also related to the societal level, where policies, regulations and laws influence the availability of welfare services, such as Special Transport Services.

Decreased mobility in life is associated with decreased quality of life (Rantakokko, 2016) and increased social isolation (Schrempft, 2019). Decreased walking performance is also associated with higher all‐cause mortality (Newman, 2006). Consequently, interventions aiming to increase outdoor mobility could impact quality of life and influence activity limitations and participation restrictions in the community and in life overall in a positive way.

The population and target of this review will be people living with disabilities that receive interventions aimed to improve outdoor mobility.

### 3.2 The intervention

**Figure 1:**
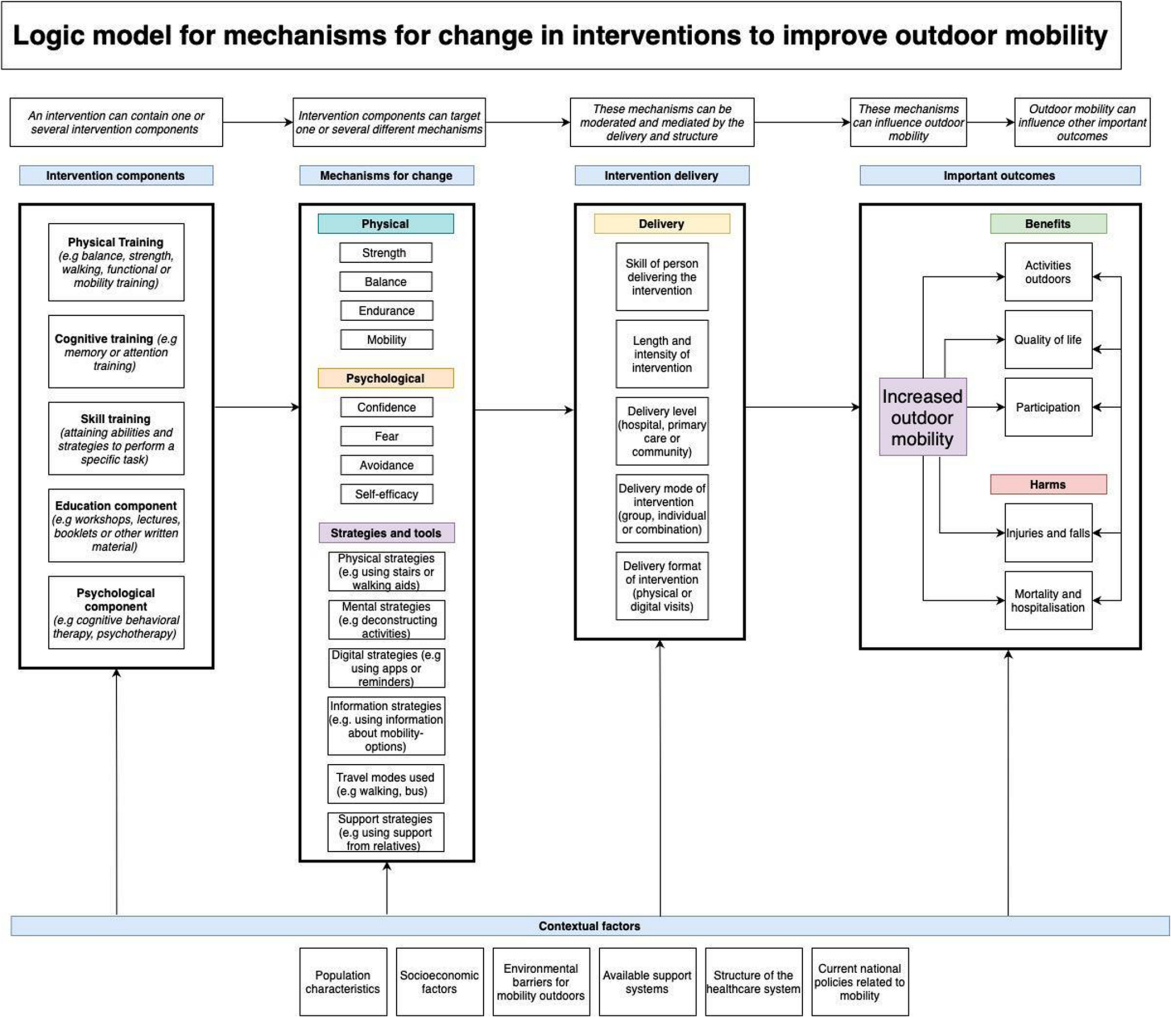
Logic model for interventions to improve outdoor mobility.

This review will explore different types of interventions targeted to improve outdoor mobility among adults living with disabilities. Interventions can include, but are not limited to, educational or psychological/behavioural therapies, physical training interventions, cognitive training interventions or skill training interventions. Interventions can also use and combine several different intervention components within a complex intervention. The interventions are described more in detail in Figure 1.

For example, a physical training intervention can be aimed to increase balance by training of physical exercises, a skill training intervention can aim to facilitate the use of mental strategies to deconstruct and train specific parts of movement or travel or a cognitive training intervention can aim to reduce fear and avoidance to increase walking outside.

Interventions could be delivered in a variety of settings, including healthcare, the workplace, in the home or in the community and/or digitally. The delivery of the intervention can also be done in different ways and by different persons, including rehabilitation personnel, social workers, by digital content or by a combination of sources. The interventions can also be delivered in individual sessions, in groups or a combination of the two. Interventions to improve outdoor mobility can vary in intensity and length and include brief educational information in a single session, or include longer and intensive rehabilitation programs containing many intervention components. The interventions can also be tailored to the individual or use a standardized intervention protocol.

Interventions that solely target alterations in the environment (e.g., new infrastructure, home adjustments, or increased access to parks) will not be within the scope of the review.

### 3.3 How the intervention might work

Outdoor mobility interventions targeting people living with disabilities aim to improve the ability to move, travel and orient outside the home. In turn, this can influence the number of activities outside the home, increase participation, or improve quality of life. However, outdoor mobility interventions may also lead to harm like falls or injuries or have unforeseen effects which could lead to mortality or hospitalization, which can be related to an increased time walking outdoors and exposure to new environments.

An additional unintended harmful effect could be related to interventions making participants more aware of their disability and their actual activity limitations, especially if they have a chronic condition, which could decrease quality of life.

Outdoor mobility is a complex activity and interventions to improve outdoor mobility can be more or less complex and include one or several intervention components (Craig, 2008). An intervention component can be defined as ‘a discrete, active element of the intervention that could be implemented independently of other elements’ (Lewin, 2017). For example, a physical training component in an intervention can aim to improve outdoor mobility through mechanisms like increasing strength, coordination or balance. A cognitive training component can aim to improve outdoor mobility through mechanisms like increasing attention or memory to be able to navigate the outdoor environment. In addition, skill training components can aim to improve outdoor mobility through mechanisms like attaining abilities to use tools or strategies for outdoor mobility. Education components can try to inform and create knowledge about available travel modes and psychological components can aim to improve outdoor mobility through reducing fear or avoidance of outdoor mobility or increasing self‐efficacy.

Furthermore, the delivery and structure of the intervention could influence the outcomes. For instance, if the intervention is delivered in the home or in a hospital, or if the intervention is based on a digital platform or group‐ or individual‐based (Kubina, 2013) could make a difference. In addition, the number and intensity of treatment sessions or the length of the treatment period could also influence important outcomes. Moreover, important outcomes can be influenced by the skill of the person delivering the intervention, if it is delivered by interdisciplinary teams and if it requires certain interventionist skills. In addition, outcomes could be affected if the intervention includes motivational components or platforms that could facilitate actual outdoor mobility.

The potential effects of interventions that aim to improve outdoor mobility can differ depending on the specific target population, and the context and outdoor environment the population will be mobile in. In addition, national policies, available support systems for outdoor mobility and the structure of the healthcare system could influence the effects of the interventions in multiple and complex ways.

### 3.4 Why it is important to do this review

Systematic reviews evaluating interventions for people with disabilities—a broad population including but not limited to different diseases and conditions—are scarce. For example, two systematic reviews have explored interventions to support community participation for people living with disabilities (Gross, 2020) and interventions to increase motor skills (Bishop, 2018). Recently published trials as well as ongoing trials (Chang, 2020), have the potential to be included in this current review and further deepen the evidence base for outdoor mobility interventions.

Previously published systematic reviews of outdoor mobility interventions were limited to walking as a means of outdoor mobility but left out other modes of transport like buses, trains or bikes. For example, a previous systematic review evaluated interventions for improving community ambulation for people with stroke (Barclay, 2015). This review focused specifically on interventions improving walking in the community and excluded trials exploring other modes of mobility and travel. The literature search was conducted in 2014 and only five trials presenting a diverse set of outcome measures could be included, which resulted in no conclusions being able to be drawn from the limited sample. An upcoming systematic review (Barclay, 2020) will explore interventions to improve walking in the community but will only include populations of older adults, regardless of disability. Furthermore, a previous systematic review explored factors in relation to outdoor mobility for people with multiple sclerosis (van der Feen, 2020) but did not locate any randomized controlled trials (RCT) to evaluate the efficacy of interventions to improve outdoor mobility.

In summary, previous systematic reviews have explored different modes of outdoor mobility for specific populations based on diagnoses but have not found sufficient published evidence to be able to draw any conclusions. To our knowledge, no previous systematic review has specifically explored whether interventions to improve outdoor mobility are effective for people with disabilities.

Furthermore, to our knowledge, no systematic review has explored differences in effect estimates between causes, diseases or disorders that can lead to disabilities, or explored the potential intervention mechanisms of action or intervention components which could have an influence on important outcomes. Thus, previous systematic reviews have not been able to guide policymakers or clinicians in decisions about implementation of interventions to promote outdoor mobility. There is uncertainty related to what population interventions to improve outdoor mobility have been previously evaluated. Moreover, previous reviews have not been able to provide guidance on potentially effective components or modes of delivery for the design of future interventions aimed at improving outdoor mobility for adults living with disabilities.

This systematic review, **with the aim to evaluate the efficacy of outdoor mobility interventions**, has the potential to guide policymakers and clinicians in making evidence‐informed decisions towards the goal of increasing accessibility, participation and activities in the community and to work towards equal opportunities and increase health and quality of life for people living with disabilities. The conclusions of this review could also inform the development and implementation of future interventions designed to improve outdoor mobility for people with disabilities.

## 4 OBJECTIVES

1. To assess the efficacy of interventions aiming to improve outdoor mobility for adults living with disabilities.
2. To explore if the efficacy varies between different populations and different intervention components.

## 5 METHODS

### 5.1 Criteria for considering studies for this review

A pre‐specified protocol was created and is available (Ringsten, 2022).

#### 5.1.1 Types of studies

Eligible study designs included in the review are RCTs where participants have been randomly allocated to an intervention or control group. To minimize the risk of bias of the effect estimates we excluded cluster randomized trials, cross‐over trials and quasi‐randomized trials in this review. We also excluded non‐randomized studies, observational studies and case reports.

The decision to restrict the study design to only randomized controlled studies of interventions was done because it minimizes the risk of any bias influencing the effect estimates of these interventions. Randomization is the only approach to prevent systematic differences in  baseline characteristics of participants within the intervention and control group in terms of both known, unknown or unmeasured confounders (Cochrane Handbook). We acknowledged the trade‐off between our restrictive criteria for study designs—which could lead to including fewer studies within the review—compared with a broader criterion including more studies but subsequently with a higher risk of bias.

#### 5.1.2 Types of participants

Participants eligible for inclusion were all adults, aged 18 or above, living with one or multiple disabilities. All disabilities, including physical, mental, cognitive or sensory impairments, or activity limitations and participation restrictions, were included. The underlying disease, disorder or cause of disability, as described in the included studies, were used to describe the study populations. Additional measures and classifications were used, if available, to further describe each study population.

For example, we planned to include study populations living with:

- Diseases of the musculoskeletal system or connective tissue, like osteoarthritis and fractures.
- Mental, behavioural or neurodevelopmental disorders, like depression and autism.
- Diseases of the nervous system, like multiple sclerosis and stroke.
- Diseases of the visual system, like vision impairments.
- Injury, poisoning or certain other consequences of external causes, like traumatic amputations.
- Diseases of the circulatory system, like ischaemic heart disease.
- Diseases of the respiratory system, like chronic obstructive pulmonary disease.
- Diseases related to ageing, like pathological processes which lead to the loss of adaptation and progress in older ages.

We included participants in all types of settings and did not limit the inclusion to any specific demographics.

We excluded studies where more than 50% of the population were below the age of 18. We also excluded studies where more than 50% of the population did not have disabilities, defined as impairments in a person's body structure or mental functioning, activity limitations or participation restrictions.

#### 5.1.3 Types of interventions

The review included interventions that aimed to improve outdoor mobility for people living with disabilities. It includes, for example, educational interventions, physical training interventions, cognitive or behavioural training interventions or skill training interventions. Interventions could be delivered on their own or in packages of several interventions by a single health care provider or a multidisciplinary team.

The included interventions could be delivered in the community, for example, within healthcare, the workplace, within the home, digitally or by groups in the community. The interventions could be delivered directly related to an injury, but also several years after having received a diagnosis, commonly for people living with a progressive disorder. Interventions aimed solely aimed at health promotion and/or primary prevention were excluded. For example, interventions aiming to prevent a future decline in outdoor mobility for older adults who currently do not have limited mobility outdoors were excluded. The reason was that such health promotive or preventative interventions are substantially different from those aiming to improve already limited mobility, and the benefits and harms would be different, particularly regarding falls. In addition, such interventions could be delivered to populations without disabilities and limited outdoor mobility, which is different from the population targeted in this review.

No restrictions were placed on duration, intensity and way of delivery of the intervention, as it can vary due to different needs of the participants and resources for delivery in the trial context. We extracted and described the duration, intensity and frequency for all included interventions.

The focus of the intervention to improve outdoor mobility had to be clearly described as an aim or purpose of the intervention to be included. Such descriptions were extracted for all included interventions. Interventions without an aim or purpose to improve outdoor mobility were excluded. For example, interventions aiming to only improve physical activity levels or walking speed for the participants. In addition, interventions with a focus on only improving mobility indoors or personal ADL were excluded.

Environmental components could be included as a part of an intervention only if they were part of multi‐component interventions that include components aimed at the capacity or performance of the participants as well. Such interventions could include home modifications in combination with skill training aiming to increase the performance to travel outside, or skill training to use buses.

Interventions that solely target alterations in the environment were excluded from this review, because such interventions have fundamentally different mechanisms of action and delivery. Further, alterations in the environment often serve as a mediator for other interventions and it may also be difficult to use randomized controlled trials since blinding may be problematic. This includes interventions targeting infrastructure, changes in organizations, routines or policies, implementation of ramps or lifts, implementation of digital support systems, only providing mobility aids, adjustments in the home or increased access to training sites or parks.

We included two comparisons in the review:

1. Outdoor mobility intervention compared with a control intervention not expected to improve outdoor mobility. The control interventions included:
  - A usual care‐control group as stated by the trial authors.
  - A wait‐list control group.
  - A control group receiving no intervention.
  - An attention control group, that is, a control intervention not aimed to improve outdoor mobility.
2. An outdoor mobility intervention compared to another outdoor mobility intervention. This included interventions containing different components or different forms of delivery, for example individual versus group delivery, digital versus physical delivery or including balance training in an educational intervention to improve outdoor mobility in only one group.

### 5.2 Types of outcome measures

We extracted outcome data from studies using both self‐reported and observer‐reported outcome measures. The outcome measures could be reported by digital means (e.g., activity monitors and GPS monitors), by the use of journals, questionnaires or other scales or assessments.

#### 5.2.1 Important outcomes

We included any of the six following important outcomes in the review:

**1. Activity outside the home**

Activity outside the home (defined according to ICF as ‘the execution of a task or action by an individual’) could be measured in continuous outcome measures, for example:

- Number of times going outside the home
- Journeys outside the home
- Activities outside the home
- Steps taken outside the home
- Specific assessment tools such as the ‘Life Space Assessment Questionnaire’ (Baker, 2003).

The outcome measures were extracted and categorized according to at what time after starting the intervention they were collected, defined as short‐term (≤6 months) or long‐term (≥7 months).

**2. Engagement in everyday life activities**

Engagement in everyday life activities (defined according to Kielhofner and colleagues (Taylor, 2017) as ‘engagement in work, play, or activities of daily living that are part of one's socio‐cultural context and that are desired and/or necessary to one's well‐being’) can be measured by dichotomous outcome measures, for example, where study participants answered ‘yes’ or ‘no’ to questions such as:

- ‘Can you do what you want to do in your life?’
- ‘Are you able to engage in valuable activities?’
- ‘Do you get out of the house as much as you would like?’
- ‘Do you avoid certain activities outdoors?’

The outcome measures were extracted and categorized according to at what time after starting the intervention they were collected, defined as short‐term (≤6 months) or long‐term (≥7 months).

**3. Participation**

Participation (defined according to ICF as ‘involvement in a life situation’) could be measured in continuous outcome measures, for example:

- Impact of Participation and Autonomy Questionnaire (IPA) (Cardol, 2001)
- WHO Disability Assessment Schedule 2.0 (WHODAS 2.0) (Ustun, 2010)
- Participation Objective Participation Subjective Instrument (POPS) (Brown, 2004)
- Participation measure for post‐acute care (PM‐PAC) (Gandek, 2007)
- Rating of Perceived Participation (ROPP) (Sandström 2007)
- The Participation Scale (Van Brakel, 2006)
- The Keele Assessment of Participation (KAP) (Wilkie, 2005)
- Other disease‐specific measurements of Participation, if the content and construct is similar to the generic measurements

The outcome measures were extracted and categorized according to at what time after starting the intervention they were collected, defined as short‐term (≤6 months) or long‐term (≥7 months).

**4. Health‐related Quality of Life**

Health‐related quality of life (defined according to WHO, 2012 as ‘individuals’ perceptions of their position in life in the context of the culture and value systems in which they live and in relation to their goals, expectations, standards and concerns’) could be measured in continuous outcome measures, for example:

- SF36 (Ware, 2007)
- SF12 (Ware, 1996)
- EQ. 5D (Rabin, 2001)
- WHOQOL (WHO, 2012)
- Health Utilities Index (HUI) (Furlong, 2001)
- CDC HRQOL‐4 (Moriarty, 2003)
- Quality of Life Questionnaire for Older People (OPQOL‐brief) (Bowling, 2013)
- Other disease‐specific measurements of HRQOL, if the content and construct is similar to the generic measurements.

The outcome measures were extracted and categorized according to at what time after starting the intervention they were collected, defined as short‐term (≤6 months) or long‐term (≥7 months).

**5. Major adverse events**

Major adverse events could be based on dichotomous or continuous outcome measures, and included:

- Mortality
- Hospitalization
- Injuries requiring medical attention in a hospital.

The outcome measures were extracted and categorized according to at what time after starting the intervention they were collected, defined as short‐term (≤6 months) or long‐term (≥7 months).

**6. Minor adverse events**

Minor adverse events could be based on dichotomous or continuous outcome measures, for example:

- Falls
- Injuries not requiring hospitalization
- Other possible adverse events reported in the included studies.

The outcome measures were extracted and categorized according to at what time after starting the intervention they were collected, defined as short‐term (≤6 months) or long‐term (≥7 months).

Falls leading to hospitalization or injuries requiring medical attention was be classified as Major Adverse Events. Other falls were subsequently be classified as a Minor Adverse event, which has been estimated to be approximately 98.8% of all falls in a hospital setting (Kobayashi, 2017).

#### 5.2.2 Duration of follow‐up

We included all duration of follow‐up, but they were separated into two different categories, that is, short‐term (6 months or less) and long‐term (7 months or more) after starting the intervention.

#### 5.2.3 Types of settings

We included interventions delivered in any setting, for example, in hospitals, clinics, the workplace, within the home, digitally or by groups or organizations in the community. We expected that the different settings could have varying impacts on the estimated effects of the intervention and explored this heterogeneity within a subgroup analysis.

### 5.3 Search methods for identification of studies

A search strategy was developed involving three information specialists with experience in conducting search strategies for systematic reviews according to the Cochrane Methodology, in close collaboration with the review authors.

We searched:

- MEDLINE (EbscoHost)
- CINAHL Complete via EBSCOhost
- Embase via Embase.com
- Cochrane Central Register of Controlled Trials (CENTRAL) via Wiley Cochrane Library
- AMED (The Allied and Complementary Medicine Database) via EBSCOhost
- PsycInfo via EBSCOhost
- PEDro (Physiotherapy Evidence Database)
- ERIC (Education resource information centre) via EBSCOhost
- Scopus
- Web of Science Core Collection: Conference Proceedings Citation Index‐Science (CPCI‐S), and Conference Proceedings Citation Index‐ Social Science & Humanities (CPCI‐SSH)

We also searched for ongoing or recently completed studies at:

- Clinicaltrials.gov
- The World Health Organization International Clinical Trials Registry Platform (WHO ICTRP)

An updated search was done when the screening and data extraction were completed. We did not apply any language restrictions. When help with translation was needed we primarily reached out to the Cochrane network for support.

#### 5.3.1 Electronic searches

The search strategy for all databases are available in Supporting Information: Appendix 1. The search was conducted in June 2022 and an updated search was conducted in January 2023 for all databases.

#### 5.3.2 Searching other resources

An additional hand search was made in the reference list of systematic reviews identified in our literature search, together with a manual search of the reference lists of the studies included in the review.

### 5.4 Data collection and analysis

#### 5.4.1 Description of methods used in primary research

We expected primary research to use a variety of methods for determining the efficacy of interventions to improve outdoor mobility, and that some of these studies used a randomized controlled design. The interventions differed in their delivery and had different active components. We anticipated that most comparison groups would be categorized as treatment as usual or no intervention. The studied populations with disabilities would vary, but we expected that a large proportion of the populations in the included studies would be people living with disabilities after stroke.

#### 5.4.2 Selection of studies

Two reviewers (MR, BI) independently screened titles and abstracts and excluded reports which did not match our inclusion and exclusion criteria. The Covidence software was used for the screening process. Any disagreements were solved by discussion together with a third reviewer (EML). When insufficient information to assess eligibility was available in the title or abstract the report was assessed in full text.

Two reviewers (MR, EML) independently assessed the full‐text versions of the reports in Covidence and disagreements were solved by discussion or together with a third reviewer by consensus (SI). If the eligibility of the report was still uncertain, attempts were made to contact the primary investigators of the report for clarification.

#### 5.4.3 Data extraction and management

Two reviewers (MR, BI) independently extracted data from the included reports. The data extraction form (Supporting Information Appendix 2) was piloted by the reviewers in Covidence and revised before starting data extraction. Disagreements in data extraction were solved by discussion or together with a third reviewer (EML) by consensus. The analyses were conducted in RevMan Web.

#### 5.4.4 Assessment of risk of bias in included studies

Two reviewers (MR, BI) independently assessed the risk of bias in each study outcome using 'Risk of bias in randomized trials (RoB 2)' tool (Sterne, 2019). Disagreements in the risk of bias‐evaluations were solved together with a third reviewer (EML) by consensus.

We assessed risk of bias for each study outcome using the 5 domains from RoB2.

- Domain 1: Bias arising from the randomization process
- Domain 2: Bias due to deviations from intended interventions
- Domain 3: Bias due to missing outcome data
- Domain 4: Bias in measurement of the outcome
- Domain 5: Bias in selection of the reported result

For each domain, a series of signalling questions (yes, probably yes, no information, probably no, no) will guide the judgement of risk of bias (low risk, some concerns and high risk). We included text alongside these judgements to provide supporting information for our decisions. The risk of bias judgements is presented alongside the effect estimates in the forest plot of a meta‐analysis.

#### 5.4.5 Measures of treatment effect

Statistical analyses were done using Review Manager (RevMan Web) and outcome data was synthesized and summarized in meta‐analyses if they were sufficiently homogeneous, both clinically and statistically. If a meta‐analysis was not feasible, we present the results narratively and, if applicable, within a forest plot.

Dichotomous data were analysed using relative risk ratio (RR) together with 95% confidence intervals (CI).

Continuous data were converted to standardized mean difference (SMD) together with 95% CIs for analysis. If data in the reports were not presented in means, standard deviations or effect sizes, methods suggested in the Cochrane Handbook was used to calculate the standardized mean difference.

If both adjusted and non‐adjusted effect estimates were reported in the same study, we extracted data from the adjusted estimate if sufficient data and assumptions were reported to use this effect estimate in a pooled analysis.

#### 5.4.6 Unit of analysis issues

Studies with multiple outcome measurement time‐points were extracted and categorized according to what time after commencing treatment they were collected, that is, short‐term (≤6 months) or long‐term (≥7 months). If several time points were reported within one of the categories, we used and extracted the time point with the longest follow‐up.

When a study with three or more intervention arms fit our inclusion criteria for more than one intervention or comparison we only included the intervention arm that is most closely aligned with the study aim and inclusion criteria. In addition, we performed a sensitivity analysis exploring the potential impact of our choice.

#### 5.4.7 Criteria for determination of independent findings

The review focused on each eligible study and not on each report of a study. Different reports from the same study population was handled as one study.

If several outcome measures were used to measure the same or a similar outcome in an included study, we only used one of the outcome measures as described in the Cochrane Handbook (Cochrane Handbook). All outcomes are transparently reported in the Characteristics of included studies‐table and sensitivity analysis was planned to be conducted, when applicable, using all outcomes reported in the outcome category to test the robustness (see Sensitivity Analysis section).

To mitigate any risk of outcome‐driven choices when several outcome measures are reported within the same outcome category within a study, we extracted outcomes based on a hierarchy inspired by examples in the Cochrane Handbook.

1. When possible, we extracted the outcome measure that is the primary outcome in each included study.
2. When possible, we extracted the outcome used in the sample size calculation within the study.
3. If the above was not possible, we extracted the outcome from the list of outcome measures presented in the ‘Important outcome’ section in the protocol. If several of the outcome measures are presented, the measures closest to the top of the list of outcome measures was extracted.

In addition, we planned to consult a statistician specialized in psychometrics for guidance if two measurements within the same outcome domain are identified and reported within a study.

A sensitivity analysis was planned when several outcomes are reported within the same domain to explore if our choice of outcome has an impact on the results.

If only a part of a measurement or a subscale were presented (e.g., only the Social Function‐domain in SF36) and full data for this outcome measure were not available through contact with study authors, we explored if the part or subscale is overlapping with the construct of the intended outcome in a meaningful way. If not, the reported outcome measure was excluded from the analysis.

#### 5.4.8 Dealing with missing data

Missing outcome‐related data were identified in the data extraction process of the included full‐text reports. We attempted to contact the primary investigators of the report to obtain missing data. If missing data were not possible to obtain from the primary investigators, or calculated based on guidance from the Cochrane Handbook which prevents calculating a SMD or a RR, the report was excluded from a meta‐analysis and the Summary of Findings‐table (SoF‐table) but were planned to be presented separately in the result section. The missingness of outcome data was handled according to the guidance provided in the Cochrane Handbook for data extraction and Risk of Bias judgements.

#### 5.4.9 Assessment of heterogeneity

To assess the heterogeneity of the effect estimates we used the *I* ^2^ statistic and the *τ* ^2^ statistic together with a visual assessment of the forest plots.

We used the thresholds, as reported in the Cochrane Handbook, as a rough guide to the interpretation of the *I* ^2^ statistic:

- 0%–40%: might not be important
- 30%–60%: may represent moderate heterogeneity
- 50%–90%: may represent substantial heterogeneity
- 75%–100%: considerable heterogeneity

Heterogeneity was further explored in subgroup analysis and sensitivity analysis.

#### 5.4.10 Assessment of reporting biases

We planned to create and examine a funnel plot to explore the possibility of small‐study biases. In interpreting the funnel plot, we planned to examine the different reasons possible for funnel plot asymmetry as outlined in section 10.4 of the Cochrane Handbook, and relate this to the results of the review. If we were able to pool more than 10 trials, we planned to undertake formal statistical tests to investigate funnel plot asymmetry and follow the recommendations in section 10.4 of the Cochrane Handbook. To assess outcome reporting bias, we checked trial protocols against published reports. For studies published after 1 July 2005, we screened the Clinical Trial Register at the International Clinical Trials Registry Platform of the World Health Organization (http://apps.who.int/trialssearch) for the trial protocol.

#### 5.4.11 Data synthesis

Based on guidance provided in the Cochrane Handbook, the decision to conduct a meta‐analysis was based on the level of heterogeneity of the interventions included, the included populations and based on the effect estimate in our comparisons and the predefined subgroups. For example, if the clinical heterogeneity is considered too large and a statistical synthesis of effect estimates from very different studies could lead to misleading effect estimates, no meta‐analysis was conducted.

We expected a large variation between different interventions included in this review and, due to the heterogeneity, we used a random‐effects model. The heterogeneity was further explored within subgroup analyses and sensitivity analyses. If we judged meta‐analysis to be inappropriate according to guidance from the Cochrane Handbook, we planned to analyse and interpret individual studies separately in a narrative synthesis.

#### 5.4.12 Subgroup analysis and investigation of heterogeneity

Subgroup analyses was planned to be conducted in three different areas based on:

1. Population characteristics. Subgroup analysis of population characteristics will be grouped according to diagnose group as defined in the included studies. For example: stroke, dementia, fractures, osteoarthritis and older adults.
2. Risk of Bias. Subgroup analysis of risk of bias judgements will be grouped according to the different overall risk of bias judgements: Low risk of bias versus high risk of bias or some concern.
3. Intervention components and intervention characteristics. Due to the number of subgroup analyses planned in this area, they will be considered exploratory in nature. These subgroups consisted of:
  3.1 Different intervention components:
    - Physical training component (e.g., balance training, resistance training, walking training)
    - Cognitive training component (e.g., memory and attention training)
    - Skill training component (Skill training, e.g., attaining abilities needed to perform a task, which can be more or less complex. For example, learning to use an app or mobility aid, learning new modes of doing a certain task or training to perform all the parts of a specific activity. For instance, perform all steps included in the travel chain when travelling with public transport)
    - Educational component (e.g., seminars, presentations, workshops or written material)
    - Psychological component (e.g., Cognitive Behavioural Therapy or Psychotherapy)
    - A combination of different intervention components that together constitute the study intervention.
  3.2 Different control interventions
    - Usual care‐control as stated by the trial authors, wait‐list control and control group receiving no intervention
    - Attention control group (i.e., an active control intervention not aimed to improve outdoor mobility)
  3.3 Different intervention areas (e.g., intervention components including changes in the environment):
    - Including environmental components in the intervention together with intervention to increase capacity and performance.
    - Including only interventions aimed to increase capacity and performance.
  3.4 Different intervention delivery:
    - Group‐based
    - Individual‐based
    - Combination of group and individual
  3.5 Different intervention duration:
    - Subgroup analysis based on the length of the intervention period. The subgroups will be defined after data collection and will be regarded as exploratory in nature.
  3.6 Different intervention mobility modes:
    - Walking (e.g., with or without walking aids)
    - Buss or tram
    - Train
    - Electric vehicles (e.g., mobility scooters, e‐bikes)
    - Combination of mobility modes
  3.7 Different intervention settings:
    - Community/outpatient (e.g., intervention delivered in your own home environment, in primary care, or by groups in the broader community)
    - Hospital
    - Nursing home

#### 5.4.13 Sensitivity analysis

Sensitivity analyses was conducted to explore the potential impact the methods chosen and used in the review could have on our conclusions. Exploration of the potential impact was planned to be done by:

- Removing studies using ‘per protocol’ or ‘as treated’ analysis (adherence to intervention) instead of intention to treat (assignment to intervention).
- Removing studies having a high risk of bias due to missing outcome data (attrition bias).
- Including the reported outcomes for the second active intervention arm in studies having more than two arms.
- Including the second reported outcome measure in studies reporting multiple outcome measures for the same outcome.
- Removing outcome measures being reported by subscales or parts of measurements, which is applicable when full outcome measures have not been reported in a study.
- Removing studies where environmental interventions are combined with interventions to increase individuals’ capacity and performance.
- Removing studies using balance training as an intervention for the outcome of adverse events, including falls.

#### 5.4.14 Treatment of qualitative research

We did not include qualitative research.

#### 5.4.15 Summary of findings and assessment of the certainty of the evidence

Two reviewers independently rated the certainty of the body of evidence for the different included outcomes. We used the GRADE system to rank the certainty of the evidence using the GRADEprofiler Guideline Development Tool software (GRADEpro) following the guidelines in the Cochrane Handbook and GRADE Handbook.

The GRADE approach includes five domains: study limitations (risk of bias), unexplained heterogeneity and inconsistency of effect, imprecision, indirectness, and publication bias to assess the certainty of the body of evidence for each outcome.

The GRADE system uses the following criteria for determining the certainty of evidence:

- High: we are very confident that the true effect lies close to that of the estimate of the effect
- Moderate: we are moderately confident in the effect estimate; the true effect is likely to be close to the estimate of effect, but there is a possibility that it is substantially different
- Low: our confidence in the effect estimate is limited; the true effect may be substantially different from the estimate of the effect
- Very low: we have very little confidence in the effect estimate; the true effect is likely to be substantially different from the estimate of effect

The GRADE system uses study design as a marker of quality. RCTs are considered to be a higher certainty of evidence but can be downgraded for important limitations.

Factors that may decrease the certainty in a body of evidence are:

- Serious or very serious study limitations (risk of bias)
- Important or serious inconsistency of results
- Some or major indirectness of evidence
- Serious or very serious imprecision
- Probability of publication bias

We included a total of 4 ‘Summary of findings’ (SoF) tables to present the main findings of the review which will follow the structure as described in the (Cochrane Handbook Chapter 14). We included key information concerning the certainty of evidence, the magnitude and confidence interval of the effect of the interventions examined, and the sum of available data on the outcomes.

1. Summary of Findings‐Tables 1 and 2 summarized the 6 outcomes measured in the short term (≤6 months) for outdoor mobility interventions compared with a control intervention.
2. Summary of Findings‐Tables 3 and 4 summarized the 6 outcomes measured in the long term (≥7 months) for outdoor mobility interventions compared with a control intervention.

## 6 RESULTS

### 6.1 Description of studies

#### 6.1.1 Results of the search

The PRISMA flowchart (Figure, 2) illustrates the full search process and the specific search strategies in each database and the number of identified articles is available in Supporting Information: Appendix 1.

**Figure 2:**
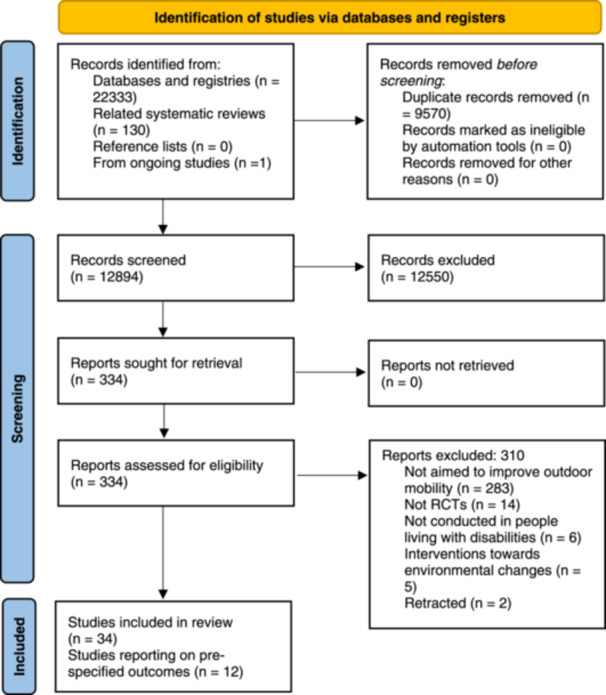
PRISMA 2020 flow diagram for new systematic reviews which included searches of databases and registers only.

We identified a total of 22,333 articles from our electronic and supplementary searches and 12,894 articles were left after duplicates were removed. We screened these records and excluded 12,550 irrelevant articles. We assessed 344 articles in full‐text, excluded 310 studies not matching our inclusion criteria and finally included 34 studies. Of these, 12 studies reported our prespecified outcomes, 10 studies did not report any of our prespecified outcomes, and 12 studies are still ongoing.

#### 6.1.2 Included studies

Detailed population‐, outcome‐ and study‐characteristics are described in Table 1 and detailed intervention characteristics are described in Table 2. The 22 studies included a total of 2675 participants and examined a total of 24 different outdoor mobility interventions. Two studies explored the efficacy of two different outdoor mobility interventions (Ada, 2012; Kim, 2016) to a control intervention and 20 studies compared one outdoor mobility intervention to a control intervention. The included sample sizes ranged from 18 (Mendoza, 2015) to 632 participants (Mänty, 2009). All studies included were individually randomized controlled trials.

**Table 5:** Characteristics of the included studies.

Study ID	Condition	Population characteristics	Study design	Country	Outcome domain(s) measured	Time‐points of measurement(s)	Recruitment setting
Ada, 2012	Stroke	Intervention group (high intensity) mean age 70 years (SD: 11), intervention group 2 (low intensity) 64 years (SD: 12) and Control group 63 years (SD: 13) years. Overall the groups were 5 years after a stroke and a majority could walk unaided (68%, 59%, 59%).	Randomized controlled trial	Australia	Health‐related quality of life by EQ‐5D; Minor adverse event by falls reported in calendars; Participation: Adelaide Activities Profile.	2, 4, 6 and 12 months	The community via advertisement (newspaper, stroke club, and media release) or referral (hospital or community therapists, stroke liaison officer, and word of mouth).
Best, 2016	Wheelchair users	Intervention group mean age 49.1 (SD: 18.7) years and comparison group 48.5 (SD: 15.2) years. Overall 68% in the groups had a previous spinal cord injury, and intervention group had a mean of 9 years and control group 18 years of experience using wheelchairs.	Randomized controlled trial	Canada	Activity outside the home by Life Space Assessment	6 weeks	After discharge from rehabilitation, in community (through clinicians, wheelchair vendors, word of mouth, and posters) and snowball sampling
Brock, 2011	Stroke	Intervention group mean age 61.3 (SD: 13.0) years and comparison group 56.6 (SD: 15.8) years. Overall the groups were 2 months after stroke and 35% used a walking aid.	Randomized controlled trial	Australia and Germany	None of our specified outcomes were reported. The study reported the following outcomes: 6MWT, Gait velocity, Balance	NA	Though rehabilitation centres
DePaul, 2015	Stroke	Intervention group mean age 66.4 (SD: 11.0) years and comparison group 69.0 (SD: 12.3) years. Overall the groups were a median of 4 months after stroke and 78% used a walking aid.	Randomized controlled trial	Canada	Activity outside the home with Life Space Assessment; Participation with Stroke Impact Scale; Minor adverse events with falls; Major adverse events with incidence of stroke, cardiovascular event or mortality	1 week and 2 months	Clinician referrals from hospital and rehabilitation units and outpatient rehabilitation programs at 3 teaching hospitals, and through newspaper advertisements and local stroke survivor groups.
Fairhall, 2012	Older adults	Intervention group mean age 83 (SD: 5.8) years and comparison group age 83 (SD: 5.9) years. Overall the groups included frail older adults with a Barthel scores of 93 and 94 respectively, and lived with an overall mean of 7 medical conditions.	Randomized controlled trial	Australia	Activity outside the home with Life‐space Assessment; Engagement in everyday activities with the question ‘do you get out of the house as much as you would like?’; Participation with Revised Reintegration to Normal Living Index; Health‐related Quality of life with EQ‐5D VAS; Major adverse events with any hospitalization	3 months and 12 months	After discharge from hospital or the community arm of the hospital
Jeong, 2016	Stroke	Intervention group mean age 73.7 (SD: 3.8) years and comparison group 71.4 (SD: 4.1). Overall the groups were mean 9 and 10 months respectively after a stroke, 59% walked with a cane, and 41% without any need of support.	Randomized controlled trial	South Korea	None of our specified outcomes were reported. The study reported the following outcomes: 6MWT, TUG, Berg Balance Scale, Activities‐Specific Balance Confidence	NA	No information provided
Kim, 2014	Stroke	Intervention group mean age 50 (SD: 10) years and comparison group 51 (SD: 7) years. Overall the groups were mean 6 months and 9 months respectively after a stroke.	Randomized controlled trial	South Korea	Participation with Stroke Impact Scale	3 days post‐intervention (4 weeks + 3 days after first intervention visit)	People doing through standard inpatient rehabilitation in a hospital setting.
Kim, 2016	Stroke	Intervention group (virtual reality) mean age 56.2 (SD: 7.6) years, intervention group (community ambulation) 52.0 (7.3) years and comparison group 48.71 (9.27) years. Overall the groups were 8, 13 and 17 months after a stroke respectively.	Randomized controlled trial	South Korea	None of our specified outcomes were reported. The study reported the following outcomes: TUG, Balance confidence, 6MWT	NA	Inpatient rehabilitation hospital
Logan, 2004	Stroke	Intervention group mean age 74 (SD: 8.4) years and comparison group 74 (SD: 8.6) years. Overall the groups were a mean 11 and 10 months after a stroke.	Randomized controlled trial	United Kingdom	Activity outside the home by number of journeys outdoors; Engagement in everyday activities by a single question related reaching the level of outdoor mobility one would like to have; Participation by Nottingham extended activities of daily living scale	4 months, 10 months	General practice registers and sources in the community, including persons living in care homes.
Logan, 2014	Stroke	Intervention group mean age 71.5 (SD: 12.1) years and comparison group 71.7 (SD: 12.1) years. Overall the group were a mean 37 months and 43.2 months respectively after a stroke and a social functioning score SF36 was 50.1 and 45.9 respectively out of 100.	Randomized controlled trial	United Kingdom	Activity outside the home by Number of journeys outdoors; Health‐related quality of life by SF‐36 and EQ. 5D; Engagement in everyday activities by Overall satisfaction with outdoor mobility; Participation by Nottingham Extended Activities of Daily Living (NEADL) scale; Minor adverse events by number of falls; Major adverse events by mortality	6 months and 12 months	General practice databases, stroke registers and approaching patients attending post‐stroke outpatient clinics as they were discharged from rehabilitation, adverts within local newspapers, local trust publications and relevant websites, establishing a study website, various articles within local publications and local press, visiting local stroke groups and widespread distribution of the study poster to relevant areas.
Lord, 2008	Stroke	Intervention group mean age 64.2 years and comparison group 60.7 years. Overall the groups were hospitalized on average 55 days and 49.5 days respectively.	Randomized controlled trial	New Zealand	Engagement in everyday life activities by question about ability to be independent i outdoor mobility and ability to go to a grocery store; Participation by Subjective Index of Physical and Social Outcome; Major adverse events by recurrence of stroke	Post‐intervention and at 6 months.	After discharge from hospital and rehabilitation units
Magaziner, 2019	Post hip fracture	Intervention group mean age 80.3 (SD: 8.0) years and comparison group mean age 81.2 (SD: 8.8) years. Overall the groups were 14 weeks since the hip fracture.	Randomized controlled trial	USA	Minor adverse events by composite score of ‘mild or moderate harms’, including falls; Major adverse events by composite score of ‘Severe or life‐threatening’ and ‘fatal’ harms	16 weeks	Medical chart review after admission to hospital, rehabilitation centres, or other agencies that care for older adults or study recruitment flyers, advertisements, social media, or referral from a clinician.
Mänty, 2009	Older adults	Intervention group mean age 77.6 (SD: 1.9) years and comparison group 77.6 (SD: 1.9) years. Overall the groups had 3 chronic conditions and used 4 prescribed medications.	Randomized controlled trial	Finland	None of our specified outcomes were reported. The study reported the following outcomes: Perceived difficulty in walking 2 km and 0.5 km, babitual physical activity, physician‐diagnosed chronic conditions, Center for Epidemiological Studies Depression scale, MMSE	NA	Finish population registry of all 75‐81 year olds
Mendoza, 2015	Stroke	Intervention group mean age 47 years comparison group 49 years. Overall the groups were 39 and 59 months after a stroke respectively and had a Barthel Index Score of 93 and 92 respectively.	Randomized controlled trial	Philippines	None of our specified outcomes were reported. The study reported the following outcomes: The Six Minute Walk Test (6MWT), Ten Meter Walk Test (10MWT), Timed Up and Down Stairs (TUDS)	NA	Convenience sampling, word‐of‐mouth advertisement in a hospital‐based stroke support group and their community‐based network
Miller, 2019	Wheelchair users	Intervention group mean age 66.2 (SD: 7) years and comparison group 63.1 (SD: 8.7) years. Overall the groups had used a wheelchair for 4 and 9 years respectively and 77% and 78% used the wheelchair daily.	Randomized controlled trial	Canada	Activity outside the home by Life Space Assessment; Participation by Late life function and disability index; Health‐related quality of life by Health Utility Index	6 months	Rehabilitation clinics, wheelchair seating programs, wheelchair vendors, and through word of mouth.
Park, 2011	Stroke	Intervention group mean age 59 (SD: 8) years and comparison group mean age 57 (SD: 8) years. Overall the groups walked with a cane 48% and 28% walked without any walking aid.	Randomized controlled trial	South Korea	None of our specified outcomes were reported. The study reported the following outcomes: 10‐m walk test, 6‐min walk test, 300‐m walking test, walking ability questionnaire, Activities‐specific balance confidence scale	NA	Inpatient rehabilitation hospital
Park, 2016	Stroke	Intervention group mean age 57 (SD: 7) years and comparison group mean age 55 (SD: 8) years. Overall the groups were 34 and 21 months after a stroke respectively, and had a score of 80 and 79 in Modified Barthel Index respectively.	Randomized controlled trial	South Korea	None of our specified outcomes were reported. The study reported the following outcomes: 10‐m walk test, 300‐m walking test, Activities‐specific balance confidence scale, temporal and spa‐tial parameters of gait,	NA	Inpatient local rehabilitation hospital
Rantanen, 2015	Older adults	Intervention group mean age 81.9 (SD: 5.6) and comparison group 82.0 (SD: 6.3). Overall 67% in the groups had difficulties in mobility more than 500 m and had 3 chronic conditions.	Randomized controlled trial	Finland	Health‐related quality of life by The World Health Organization Quality of Life	3 months	Municipal home care services and other outpatient care facilities
Turunen, 2020	Older adults	Intervention group mean age 79 (SD: 8.4) and comparison group 79.7 (SD: 8.1). Overall the groups had 39% and 41% hospitalized after a fracture and 39% and 31% after joint replacement respectively. 68% in the intervention group and 69% used walking aids outside before hospitalization.	Randomized controlled trial	Finland	Engagement in everyday life activities by ability to walk outside without any hindrance; Major adverse events by need of emergency room service; Minor adverse events by need for healthcare service	6 and 12 months	Hospital setting, and review of medical records for inclusion by a nurse.
Ullrich, 2021	Cognitive impairment	Intervention group mean age 82.2 (SD: 5.8) and comparison group 82.4 (6.2). Overall the groups had a Mini‐mental state examination scores of 23 and used approximately 9 medications.	Randomized controlled trial	Germany	Activity outside the home by Life Space Assessment Persons with Cognitive Impairment; Health‐related quality of life by EQ‐5D VAS	12 and 24 weeks	Rehabilitation wards of a geriatric hospital during conduction of a geriatric rehabilitation program
Wang, 2021	Older adults	Intervention group mean age 71.5 (SD: 5.3) years and comparison group 73.5 (SD: 5.6) years. Overall the groups had a Mini‐mental state examination score 29 in both groups.	Randomized controlled trial	Taiwan	None of our specified outcomes were reported. The study reported the following outcomes: Executive interview (EXIT‐25), Trail‐making test, Stroop Color and word‐test, Timed up‐and‐go, time to walk 400 m.	NA	Through local communities
Yang, 2008	Stroke	Intervention group mean age 61 (SD: 9) years and comparison group 55 (SD: 12) years. Overall the groups were 6 years after a stroke, 35% used no walking aids and 40% used a cane.	Randomized controlled trial	Taiwan	None of our specified outcomes were reported. The study reported the following outcomes: 10MWT, Community walk speed test, Walking ability questionnaire, Activities‐specific balance confidence (ABC) scale	NA	Though community groups

**Table 6:** Intervention characteristics.

			*What?*			*Who?*	*Where?*
Study ID	Intervention category	Comparison	Intervention description	Tailoring of intervention	Intensity of intervention	Intervention deliverers	Setting and delivery
Ada, 2012	Skill training indoors and outdoors by walking (high intensity)	Inactive control—No treatment	Skill training inside by treadmill and outside in the community. By treadmill training focused on gait‐pattern, slopes and cognitive tasks. Outside training included stairs, curbs and rough terrain.	Tailored to progression of training	48 sessions of 0.5 h over 16 weeks	Physiotherapists	Individual delivery in an outpatient clinic and in the community
Ada, 2012	Skill training indoors and outdoors by walking (low intensity)	Inactive control—No treatment	Skill training inside by treadmill and outside in the community. Training on treadmill focused on gait‐pattern, slopes and cognitive tasks. Outside training included stairs, curbs and rough terrain.	Tailored to progression of training	24 sessions of 0.5 h over 8 weeks	Physiotherapists	Individual delivery in an outpatient clinic and in the community
Best, 2016	Skill training outdoors using wheelchairs	Inactive control—No treatment	Skill training outside to reach participants selected goals related to performing activities and negotiating the physical environment together with a peer trainer. For example, it included manoeuvring the wheelchair in an outside environment, descending a curb, problem‐solving skills, managing social situations and controlling emotions delivered at different community locations	Fully tailored to participant goals	6 sessions of 1.5‐h over 3‐6 weeks	A peer‐trainer and a support trainer. Family members where sometimes involved.	Pairs of wheelchair users in the community
Brock, 2011	Physical training indoors	Active control—General physiotherapy interventions	Physical training by Bobath concept to improve walking in different environmental contexts.	Not stated	6 sessions of 1 h over 2 weeks	Physiotherapists	Individual delivery in the community
DePaul, 2015	Skill training indoors by walking	Active control—Body‐weight supported treadmill walking	Skill training inside related to walking and negotiating challenges in the physical environment. For example it included shorter and longer walks, going over steps, slopes and curbs, avoiding obstacles and changing direction.	Tailored to progression of training	15 sessions of 1 h over 5 weeks	Physiotherapists	Individual delivery in an outpatient clinic
Fairhall, 2012	Physical training indoors	Inactive control—No treatment	Physical training and home training recommendation containing strength, balance and endurance exercises to reach mobility goals. It also included regular case conferences to coordinate, if indicated, recommendations of mobility aids, weight loss, chronic disease management, psychologist support, or follow‐up contacts with volunteers.	Fully tailored to participant goals	10 sessions of 0.75–1 h	Physiotherapists in collaboration with an interdisciplinary team (dietician, geriatrician, rehabilitation physician, nurse and when indicated an occupational therapist).	Individual delivery in the home and community
Jeong, 2016	Skill training indoors by walking	Active control—General physiotherapy interventions and regular treadmill walking	Skill training inside including general physiotherapy interventions in combination with obstacle walking on a treadmill representing obstacles likely to be encountered in the home and community.	Probably tailored to progression of training (but not stated)	20 sessions of 1 h over 4 weeks	Physiotherapists	Not stated
Kim, 2014	Skill training outdoors by walking	Inactive control—Usual care	Skill training outside in the community. For example this included walking over pavements, stairs, ramps, over obstacles or at a shopping centre.	Probably tailored to progression of training (but not stated)	20 sessions of 30 min over four weeks.	Therapists (no more information available)	Individual delivery in the community around a hospital
Kim, 2016	Skill training indoors by walking (VR)	Inactive control—Usual care	Skill training inside using virtual reality‐based training while walking on a treadmill in a virtual community to train community ambulation. It included an increase in speed, uphill walking, stepping over obstacles and walking on sidewalks	No information	12 of 0.5 h over 4 weeks	No information	Individual delivery in an outpatient clinic
Kim, 2016	Skill training outdoors by walking	Inactive control—Usual care	Skill training outdoors in the community. This included overground walking, stair walking, slope walking, and walking on unstable surfaces.	No information	12 of 0.5 h over 4 weeks	No information	Individual delivery in an outpatient clinic and in the community
Logan, 2004	Skill training outdoors using multiple mobility modes	Inactive control—Usual care	Skill training outside to reach participant selected mobility goals. This included clinical assessments, information about local mobility options, the use of minor aids or adaptations such as walking aids, overcoming fear by, for example, accompanying participants in their mobility mode until confidence was restored.	Fully tailored to participant goals	Up to 8 sessions over 3 months. Mean contact time was estimated to 1.9 h in total.	Occupational therapists	Individual delivery in an outpatient clinic, the community and the home
Logan, 2014	Skill training outdoors using multiple mobility modes	Inactive control—Usual care	Skill training outside to increase outdoor mobility and participant mobility goals by targeting physical difficulties, developing skills to maximize the individuals potential and overcoming psychological barriers. The main component of the intervention was for participants to repeatedly practise outdoor mobility, but also goal‐setting and strategies to remove barriers and enhance skills. For example this included walking, using buses, taxis, walking, voluntary drivers and mobility scooters.	Fully tailored to participant goals	Up to 12 sessions, median 7 in the study.	Therapists (3 physiotherapists, 17 were occupational therapists and 9 assistant practitioners)	Individual delivery in an outpatient clinic, the community and the home
Lord, 2008	Skill training outdoors by walking	Active control—General physiotherapy interventions	Skill training outside in the community. This included walking in the local shopping centre, bowling club or local park.	Fully tailored to participant goals	14 sessions over 7 weeks	Physiotherapists and assistants	Individual delivery in the community
Magaziner, 2019	Physical training indoors	Active control—Range‐of‐motion exercises and TENS	Physical training including focused on lower extremity strength, endurance, balance, and function.	Not stated	32–40 sessions of 1 h over 16 weeks	Physiotherapists	Individual delivery in the home
Mänty, 2009	Physical training outdoors	Inactive control—No treatment	Physical training intervention to promote and motivate to physical activity and outdoor mobility. Includes a counselling approach, making plans to start physical activity and strategies to overcome barriers to increase exercise and physical activity to improve outdoor mobility.	Fully tailored to participant goals	1 session of 0.8 h and telephone contacts every 4 months	Physiotherapists	Individual delivery in an outpatient clinic and in the community
Mendoza, 2021	Skill training inside	Active control—Training program	Physical training inside in a circuit training format aimed to mimic mobility demands inside and outside the home. This included walking on unstable surfaces, ascending and descending ramps and walking while turning the head.	Probably tailored to progression of training (but not stated)	12 sessions of 1 h over 4 weeks three times a week for 4 weeks with each session lasting for 60 min	Physiotherapists	Group delivery in an outpatient clinic
Miller, 2019	Skill training outdoors using wheelchairs	Active control—Wheelchair‐focused seminars	Skill training outside using wheelchairs to reach skill and mobility goals in the community. This could include to go descend curbs, using public transport, shopping, or be confident to leave the home.	Fully tailored to participant goals	6 sessions of 1.5 h over 3–6 weeks	Peer‐trainers (wheelchair users from the community) and support trainers (health care professionals)	Individual delivery in outpatient clinic and in the community
Park, 2011	Skill training outdoors by walking	Inactive control—Usual care	Skill training outside by walking in various community situations. This included walking on pavements, stairs, ramps and shopping centres.	Probably tailored to progression of training (but not stated)	12 sessions of 1 h over 4 weeks	Physiotherapists	Individual delivery in the community
Park, 2016	Skill training outdoors by walking	Active control—Video clips	Skill training outside by walking in the community, and observing video‐clips of community walking. The training included walking in complex and unpredictable environments as parking lots and shopping centres.	Probably tailored to progression of training (but not stated)	20 sessions of 0.5–1 h over 4 weeks	Physiotherapists	Individual delivery in the community
Rantanen, 2015	Skill training outdoors by walking	Inactive control—No treatment	Skill training outside to reach out‐of‐home activities based on needs and interest. This included making an activity plan that could include going for walks, visiting places outside the home like cultural events or parks, and doing daily errands.	Fully tailored to participant goals	12 sessions over 12 weeks	Peer trainers (retired older adults) and a coordinator (healthcare professional)	Individual delivery in the community
Turunen, 2020	Physical training indoors	Inactive control—Usual care	Physical training to improve mobility and increase physical activity by going outside and exercising. This included setting activity‐goals, setting up an exercise program and counselling to motivate continued training.	Tailored to progression of training	7 sessions and 4 follow up contacts	Physiotherapists	Individual delivery in the home and the community
Ullrich, 2021	Skill training indoors and outdoors by walking	Active control—Training manual of flexibility and strength exercises, newsletter, general home‐visits and phone‐calls.	Skill training outside and inside by daily conduction of an outdoor walking course and supported by an inside exercise program focusing on balance and strength. It also included a printed poster with instructions, motivational approaches to motivate participants to behaviour change, a training diary and a pedometer.	Fully tailored to participant goals	5 sessions and daily home training	Sport scientists	Individual delivery in the home environment and outdoors
Wang, 2021	Skill training indoors by walking	Active control—Training program	Skill training inside by treadmill walking, performing video games as dual tasks. For example, this includes video‐based motor and cognitive games while walking at a self‐selected speed on a treadmill.	Tailored to progression of training	36 sessions of 1 h over 12 weeks	Physiotherapists	Individual delivery in an outpatient clinic
Yang, 2008	Skill training indoors by walking (VR)	Active control—Treadmill walking with tasks	Skill training inside by walking on a treadmill using virtual‐reality glasses projecting images of walking in a typical community. For example this virtual reality setting included crossing streets, stepping over obstacles, walking in a park and slopes.	Not stated	9 sessions of 20 min over 3 weeks	Physiotherapists	Individual delivery in an outpatient clinic

##### 6.1.2.1 Participants

Five studies were conducted in South Korea (Jeong, 2016; Kim, 2014; Kim, 2016; Park, 2011; Park, 2016), three studies were conducted in Finland (Mänty, 2009; Rantanen, 2015; Turunen, 2020), three studies in Canada (Best, 2016; DePaul, 2015; Miller, 2019), two studies in Australia (Fairhall, Ada), two studies in the United Kingdom (lLogan, 2004; Logan, 2014), two studies in Taiwan (Wang, 2021; Yang, 2008), one study in the Philippines (Mendoza, 2015), one study in Germany (Ullrich, 2021), one study in the USA (Magaziner, 2019), one study in New Zealand (Lord, 2008) and one study in both Germany and Australia (Brock, 2011).

Thirteen studies were conducted with participants with disability due to stroke (Ada, 2012; Brock, 2011; DePaul, 2015; Jeong, 2016; Kim, 2014; Kim, 2016; Logan, 2004; Logan, 2014; Lord, 2008; Mendoza, 2015; Park, 2011; Park, 2016; Yang, 2008), five studies among older adults living with a disabilities (Fairhall, 2012; Mänty, 2009; Rantanen, 2015; Turunen, 2020; Wang, 2021), two studies among wheelchair users (Best, 2016; Miller, 2019), one study among people living with disabilities after a hip fracture (Magaziner, 2019) and one study among people with cognitive impairments (Ullrich, 2021).

The mean age of the included participants varied between the studies and ranged from mean 49, SD: 17 years (Best, 2016) to 82, SD: 6 years (Ullrich, 2021).

We did not identify any studies conducted with participants with mental, behavioural or neurodevelopmental disorders, diseases of the visual system, diseases of the circulatory system or diseases of the respiratory system.

##### 6.1.2.2 Interventions

Intervention characteristics are described in detail in Table 2. Skills training to improve outdoor mobility were used in 19 different interventions reported by 16 different studies. Ten skill training interventions were delivered in the outdoor environment. Six of these interventions used walking as a mobility mode (Kim, 2014; Kim, 2016; Lord, 2008; Park, 2011; Park, 2016; Rantanen, 2015), two interventions focused on skills in relation to using wheelchairs (Best, 2016; Miller, 2019) and two used multiple mobility modes (Logan, 2004; Logan, 2014). Six skill training interventions were delivered in an indoor environment in clinics using walking as a mobility mode. Two of these interventions used treadmills (Jeong, 2016; Wang, 2021), two focused on challenges in the indoor physical environment (DePaul, 2015; Mendoza, 2015), and two used virtual reality techniques to mimic walking in a community (Kim, 2016; Yang, 2008). Three skill training interventions were delivered both in the outdoor and indoor environment. Two of these intervention arms (Ada, 2012) combined treadmills inside and walking outside, and one intervention (Ullrich, 2021) comprised an outdoor walking training path in combination with an indoor training program.

Physical training interventions to improve outdoor mobility was used in five different interventions reported in five studies. Two interventions used strength, balance and endurance training (Fairhall, 2012; Magaziner, 2019), two interventions used physical activity support and motivational approaches to improve physical activity (Mänty, 2009; Turunen, 2020), and one intervention used Bobath‐concept training (Brock, 2011).

We did not identify any studies aiming to improve outdoor mobility through other types of interventions.

##### Comparisons

Thirteen interventions were compared to an inactive comparison group in 11 studies. Five studies used a ‘no treatment’ control (Ada, 2012; Best, 2016; Fairhall, 2012; Mänty, 2009; Rantanen, 2015) and six studies used a ‘usual care’ control (Kim, 2014; Kim, 2016; Logan, 2004; Logan, 2014; Park, 2011).

Eleven interventions were compared to active and attention control groups not aimed at improving outdoor mobility in 11 studies. Seven studies (Brock, 2011; Jeong, 2016; Lord, 2008; Magaziner, 2019; Mendoza, 2015; Ullrich, 2021; Wang, 2021) used general training programs and general physiotherapy not aimed to improve outdoor mobility, two studies used general treadmill walking with minimal challenges (DePaul, 2015; Yang, 2008), and two studies used educational material, for example, video clips and seminars (Miller, 2019; Park, 2011).

##### Outcomes

Out of the 22 studies exploring outdoor mobility interventions, seven studies (Best, 2016; DePaul, 2015; Fairhall, 2012; Logan, 2004; Logan, 2014; Miller, 2019; Ullrich, 2021) reported on activity outside the home in the short term (≤6 months) and three studies (Fairhall, 2012; Logan, 2004; Logan, 2014) reported in the longer term (≥7 months). Five studies reported on engagement in everyday life activities (Fairhall, 2012; Logan, 2004; Logan, 2014; Lord, 2008; Turunen, 2020) in the short term (≤6 months) and four studies (Fairhall, 2012; Logan, 2004; Logan, 2014; Turunen, 2020) reported in the longer term (≥7 months). Eight studies reported on participation (Ada, 2012; DePaul, 2015; Fairhall, 2012; Kim, 2014; Logan, 2004; Logan, 2014; Lord, 2008; Miller, 2019) in the short term (≤6 months) and four studies (Ada, 2012; Fairhall, 2012; Logan, 2004; Logan, 2014) reported in the longer term (≥7 months). Five studies reported on health‐related quality of life (Ada, 2012; Fairhall, 2012; Logan, 2014; Rantanen, 2015; Ullrich, 2021) in the short term (≤6 months) and three studies (Ada, 2012; Fairhall, 2012; Logan, 2014) reported in the longer term (≥7 months). Four studies reported on major adverse events (DePaul, 2015; Lord, 2008; Magaziner, 2019; Turunen, 2020) in the short term (≤6 months) and three studies (Fairhall, 2012; Logan, 2014; Turunen, 2020) reported in the longer term (≥7 months). Four studies reported on minor adverse events (Ada, 2012; DePaul, 2015; Magaziner, 2019; Turunen, 2020) in the short term (≤6 months) and four studies (Ada, 2012; Fairhall, 2012; Logan, 2014; Turunen, 2020) reported in the longer term (≥7 months). A total of 10 studies did not report on any of our important outcomes.

#### 6.1.3 Excluded studies

We excluded 310 articles after assessing the full text. All excluded articles are available in the Excluded studies section. Of these, 283 articles were not aimed to evaluate interventions to improve outdoor mobility, 14 articles did not use a randomized design, six articles were conducted in populations without disabilities, five articles evaluated interventions aimed to change the environment or infrastructure and two articles were retracted.

#### 6.1.4 Ongoing studies

We identified 12 ongoing studies.

Seven of the ongoing studies (Dean, 2021; Drks, 2021; Edgren, 2019; Haeger, 2022; Nct, 2015; Nct, 2020a; Salbach, 2019) are exploring outdoor mobility interventions in older adults. Two studies (Isrctn, 2016; Nct, 2020) are exploring outdoor mobility interventions after stroke, one in people with Parkinson's Disease (Nct, 2018a), one in mobility scooter users (Mortenson, 2017) and one in a general population of adults living with long‐term disabilities (Nct, 2018). The specific type of outdoor mobility interventions could not be described due to limited descriptions in trial registries and protocols of most of the ongoing studies.

### 6.2 Risk of bias in included studies

#### 6.2.1 Overall risk of bias

**Figure 3:**
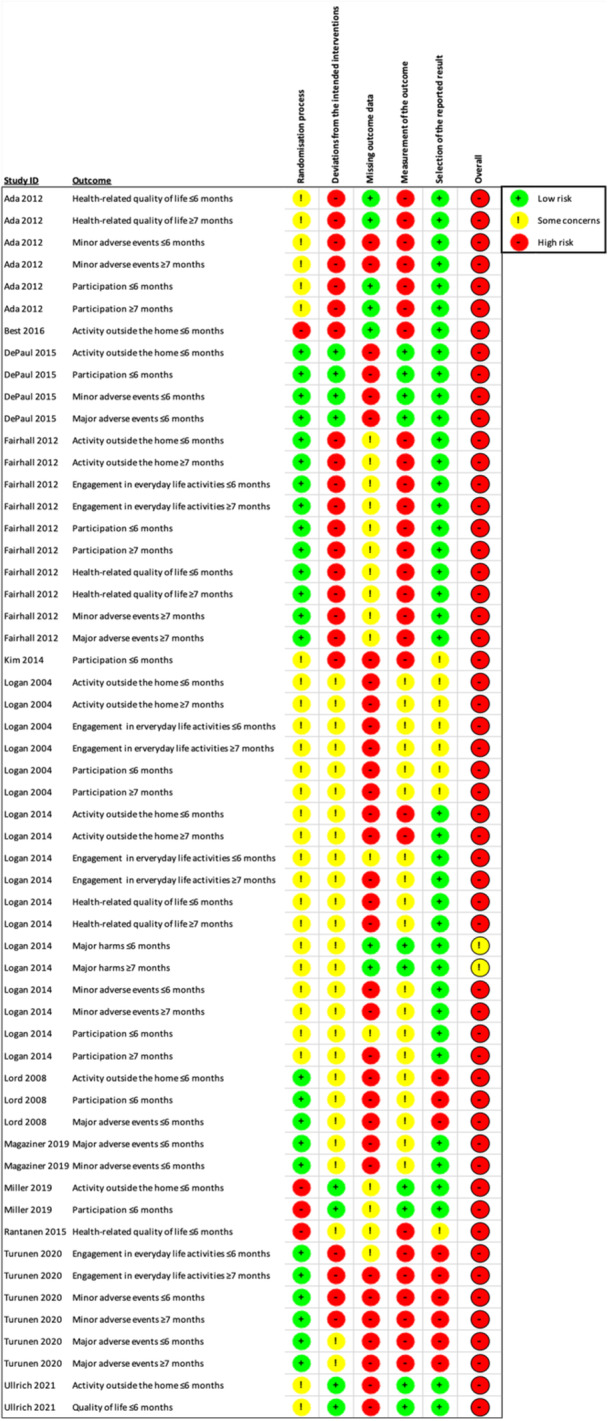
Detailed risk of bias for each outcome.

Risk of bias for each study and the reported outcome is presented in Figure 3. Detailed risk of bias assessments and consensus responses to each signalling question is available in Supporting Information Appendix 3.

Out of the 56 reported outcomes in the included studies, 55 outcomes had an overall high risk of bias, one outcome had some concerns and no outcome had an overall low risk of bias.

#### 6.2.2 Bias arising from the randomization process

We judged 25 outcomes reported in five studies (DePaul, 2015; Fairhall, 2012; Lord, 2008; Magaziner, 2019; Turunen, 2020) to have low risk of bias arising from the randomization process.

We judged 27 outcomes reported in five studies (Ada, 2012; Kim, 2014; Logan, 2004; Logan, 2014; Ullrich, 2021) to have some concern of bias arising from the randomization process, due to unclear reporting of allocation concealment and baseline imbalances in potential prognostic factors for outdoor mobility.

We judged four outcomes reported in three studies (Best, 2016; Miller, 2019; Rantanen, 2015) to have high risk of bias arising from the randomization process due to having no allocation concealment and large baseline imbalances in potential prognostic factors for outdoor mobility.

#### 6.2.3 Bias due to deviations from intended interventions

We judged eight outcomes reported in three studies (DePaul, 2015; Miller, 2019; Ullrich, 2021) to have low risk of bias due to deviations from intended interventions due to using well‐designed attention control groups and thus minimizing participant knowledge and preferences about group allocations.

We judged 26 outcomes reported in six studies (Logan, 2004; Logan, 2014; Lord, 2008; Magaziner, 2019; Rantanen, 2015; Turunen, 2020) to have some concern of bias due to deviations from intended interventions, mainly due to using comparison groups with only some attention control, or common practice interventions that do not aim to improve outdoor mobility. This would lead to knowledge about the group allocations, potential participant preference and change in health‐related behaviours with the potential to impact outcomes.

We judged 22 outcomes reported in five studies (Ada, 2012; Best, 2016; Fairhall, 2012; Kim, 2014; Turunen, 2020) to have high risk of bias due to deviations from intended interventions, mainly due to using comparison groups with no intervention delivered and with a strong likelihood of participant preference, knowledge about the group allocations and changed health‐related behaviours impacting the outcomes.

#### 6.2.4 Bias due to missing outcome data

We judged seven outcomes reported in three studies (Ada, 2012; Best, 2016; Logan, 2014) to have low risk of bias due to missing outcome data, due to having minimal loss of participants at follow‐ups and no or minimal differences between groups in reasons for discontinuing and patient characteristics.

We judged 16 outcomes reported in five studies (Fairhall, 2012; Logan, 2014; Miller, 2019; Rantanen, 2015; Turunen, 2020) to have some concern of bias due to missing outcome data, mainly due to moderate loss of participants at follow‐ups, but with no, or unclear attempts to correct for any bias and a potential impact on the results.

We judged 33 outcomes reported in nine studies (Ada, 2012; DePaul, 2015; Kim, 2014; Logan, 2004; Logan, 2014; Lord, 2008; Magaziner, 2019; Turunen, 2020; Ullrich, 2021) to have high risk of bias due to missing outcome data, mainly due to substantial loss of participants at follow‐ups with a potential of major impact on the results, together with no or unsuccessful attempts to correct for any bias.

#### 6.2.5 Bias in measurement of the outcome

We judged 10 outcomes reported in four studies (DePaul, 2015; Logan, 2014; Miller, 2019; Ullrich, 2021) to have low risk of bias in measurement of the outcome, mainly due to well‐designed attention control groups and thus minimizing participant preference and knowledge about the group allocations and limiting the impact on self‐reporting outcomes, or using objective outcomes such as mortality measured in registries with limited possibility of introducing bias.

We judged 19 outcomes reported in four studies (Logan, 2004; Logan, 2014; Lord, 2008; Magaziner, 2019) to have some concerns of bias in measurement of the outcome, mainly due to using self‐reported measures and using comparison groups with only some attention control, or common practice interventions that do not aim to improve outdoor mobility with a potential to lead to participant preference with a possibility to impact outcome reporting.

We judged 27 outcomes reported in seven studies (Ada, 2012; Best, 2016; Fairhall, 2012; Kim, 2014; Logan, 2014; Rantanen, 2015; Turunen, 2020) to have high risk of bias in measurement of the outcome. This was mainly due to using self‐reported outcome measures and using comparison groups with no intervention with a strong likelihood of participant preference and a strong likelihood to impact outcome reporting.

#### 6.2.6 Bias in selection of the reported result

We judged 39 outcomes reported in eight studies (Ada, 2012; Best, 2016; DePaul, 2015; Fairhall, 2012; Logan, 2014; Magaziner, 2019; Miller, 2019; Ullrich, 2021) to have low risk of bias in the selection of the reported results, due to having pre‐specified protocols available before any data analyses took place and no deviations on statistical or outcome measures.

We judged eight outcomes reported in three studies (Kim, 2014; Logan, 2004; Rantanen, 2015) to have some concern of bias in the selection of reported results mainly due to having retrospectively registered protocols with some potential deviations related to reporting of subscales, or no protocol with some concerns regarding reporting of only certain subscales of an outcome measure.

We judged nine outcomes reported in two studies (Lord, 2008; Turunen, 2020) to have high risk of bias in selection of reported results due to no protocol available and substantial opportunities to report certain outcome measures and scales and selection of certain analyses, or having substantial deviations from important pre‐specified outcomes or selection of new outcome measures without justification.

### 6.3 Effects of interventions

See Summary of Findings table Summary of findings Table 1; Summary of findings Table 2; Summary of findings Table 3; Summary of findings Table 4 for summaries of the effect of interventions. No studies reported the benefits and harms of other interventions than skill training and physical training.

#### 6.3.1 Comparison 1.1: Skill training interventions versus control intervention not aimed at improving outdoor mobility

##### Activity outside the home in the shorter term (≤6 months)

Six studies (Best, 2016; DePaul, 2015; Logan, 2004; Logan, 2014; Miller, 2019; Ullrich, 2021) reported this outcome.

Overall, the evidence is very uncertain if skill training interventions improve activity outside the home among people with disabilities in the shorter term (SMD: 0.18; 95% CI: −0.20 to 0.56; *I* ^2^ = 83%; 925 participants; six studies; very low certainty evidence; Analysis 1.1). The certainty of evidence was downgraded by one level for very serious risk of bias, one level for very serious imprecision and one level for very serious inconsistency.

**Analysis 1.1:**
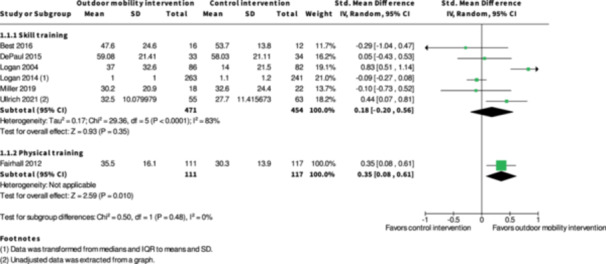
Comparison 1: Specific outdoor mobility interventions versus control interventions, Outcome 1: Activity outside the home ≤6 months.

Subgroup analysis of skill training interventions in people after stroke did not reduce uncertainty and the evidence is very uncertain if skill training interventions may improve activity outside the home among people after a stroke (SMD: −0.26; 95% CI: −0.36 to 0.88; *I* ^2^ = 92%; 739 participants; three studies; very low certainty evidence; Analysis 2.1). The certainty of evidence was downgraded by one level for very serious risk of bias, one level for very serious imprecision and one level for very serious inconsistency.

**Analysis 2.1:**
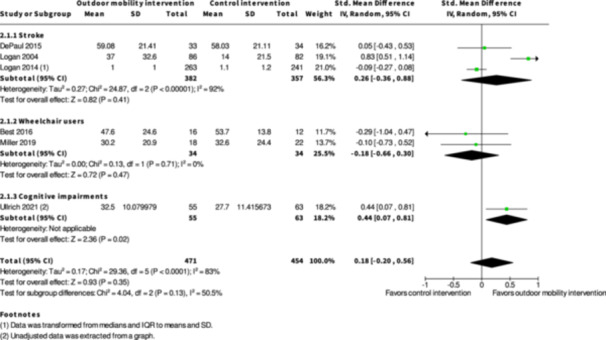
Comparison 2: Specific outdoor mobility interventions versus control interventions—by conditions (Subgroup analyses), Outcome 1: Skill training—Activity outside the home ≤6 months.

Subgroup analysis of skill training interventions in people using wheelchairs did not reduce uncertainty and the evidence is very uncertain if skill training interventions may improve activity outside the home among wheelchair users (SMD: −0.18; 95% CI: −0.66 to 0.30; *I* ^2^ = 0%; 68 participants; two studies; very low certainty evidence; Analysis 2.1). The certainty of evidence was downgraded by one level for very serious risk of bias and two level for very serious imprecision.

Subgroup analysis of skill training interventions in people with cognitive impairments slightly reduced uncertainty and may improve activity outside the home among people living with cognitive impairments (SMD: 0.44; 95% CI: 0.07 to 0.81; *I* ^2^ = NA; 118 participants; one study; low certainty evidence; Analysis 2.1). The certainty of evidence was downgraded by one level for serious risk of bias and one level for serious imprecision.

##### Activity outside the home in the longer‐term (≥7 months)

Two studies (Logan, 2004; Logan, 2014) reported this outcome, and both were conducted among people after a stroke.

Overall the evidence is very uncertain if skill training interventions improve activity outside the home in the longer term (SMD: 0.38; 95% CI: −0.55 to 1.30; *I* ^2^ = 96%; 672 participants; two studies; very low certainty evidence; Analysis 1.2). The certainty of evidence was downgraded by one level for very serious risk of bias, one level for very serious imprecision, and one level for very serious inconsistency.

**Analysis 1.2:**
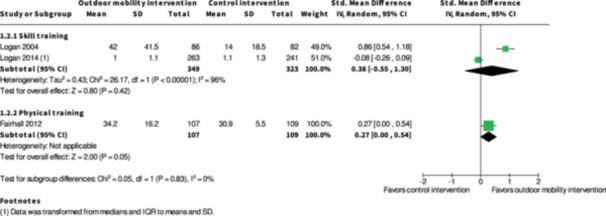
Comparison 1: Specific outdoor mobility interventions versus control interventions, Outcome 2: Activity outside the home ≥7 months.

##### Engagement in everyday life activities in the shorter term (≤6 months)

Three studies (Logan, 2004; Logan, 2014; Lord, 2008) reported this outcome, and all were conducted among people after stroke.

Overall skill training interventions may improve engagement in everyday life activities among people living with disabilities in the shorter term (RR: 1.46; 95% CI: 1.16 to 1.84; *I* ^2^ = 7%; risk difference [RD]: 0.15; 95% CI: −0.02 to 0.32; *I* ^2^ = 71%; 692 participants; three studies; low certainty evidence; Analysis 1.3). The certainty of evidence was downgraded by two levels for very serious risk of bias.

**Analysis 1.3:**
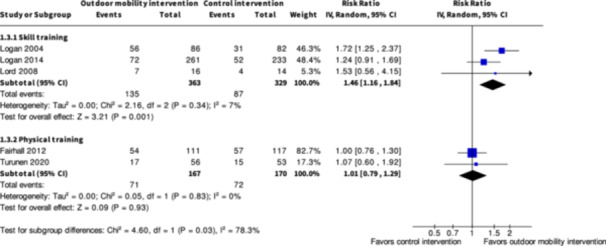
Comparison 1: Specific outdoor mobility interventions versus control interventions, Outcome 3: Engagement in everyday life activities ≤6 months.

##### Engagement in everyday life activities in the longer‐term (≥7 months)

Two studies (Logan, 2004; Logan, 2014) reported this outcome, and both were conducted among people after stroke.

Overall the evidence is very uncertain if skill training interventions improve engagement in everyday life activities in the longer term (RR: 1.40; 95% CI: 0.91 to 2.15; *I* ^2^ = 69%; RD: 0.14; 95% CI: −0.09 to 0.37; 600 participants; two studies; very low certainty evidence; Analysis 1.4). The certainty of evidence was downgraded by one level for very serious risk of bias, one level for very serious imprecision and one level for very serious inconsistency.

**Analysis 1.4:**
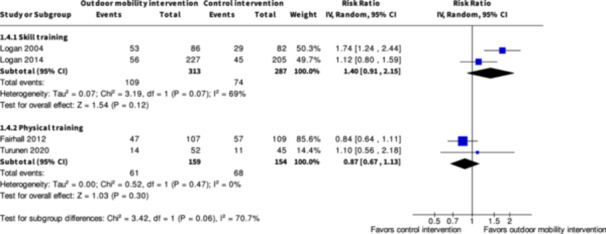
Comparison 1: Specific outdoor mobility interventions versus control interventions, Outcome 4: Engagement in everyday life activities ≥7 months.

##### Participation in the shorter term (≤6 months)

Seven studies (Ada, 2012; DePaul, 2015; Kim, 2014; Logan, 2004; Logan, 2014; Lord, 2008; Miller, 2019) reported this outcome.

Overall, the evidence is very uncertain if skill training interventions improve participation among people living with disabilities in the shorter term (SMD: 0.01; 95% CI: −0.23 to 0.25; *I* ^2^ = 53%; 886 participants; seven studies; very low certainty evidence; Analysis 1.5). The certainty of evidence was downgraded by one level for very serious risk of bias, one level for very serious imprecision and one level for serious inconsistency. Analysis 2.2, 2.3, 2.4, 2.5.

**Analysis 1.5:**
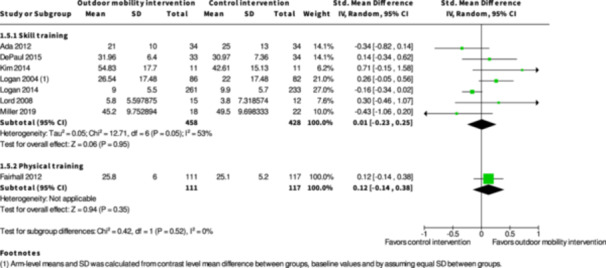
Comparison 1: Specific outdoor mobility interventions versus control interventions, Outcome 5: Participation ≤6 months.

**Analysis 2.2:**
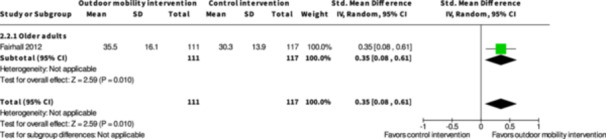
Comparison 2: Specific outdoor mobility interventions versus control interventions—by conditions (Subgroup analyses), Outcome 2: Physical training—Activity outside the home ≤6 months.

**Analysis 2.3:**
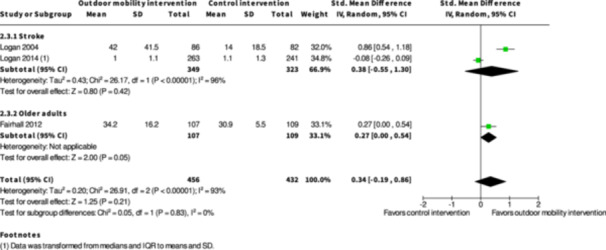
Comparison 2: Specific outdoor mobility interventions versus control interventions—by conditions (Subgroup analyses), Outcome 3: Activity outside the home ≥7 months.

**Analysis 2.4:**
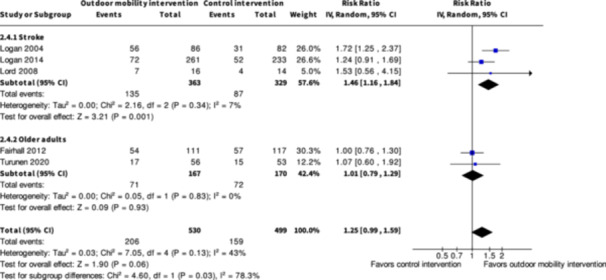
Comparison 2: Specific outdoor mobility interventions versus control interventions—by conditions (Subgroup analyses), Outcome 4: Engagement in everyday life activities ≤6 months.

**Analysis 2.5:**
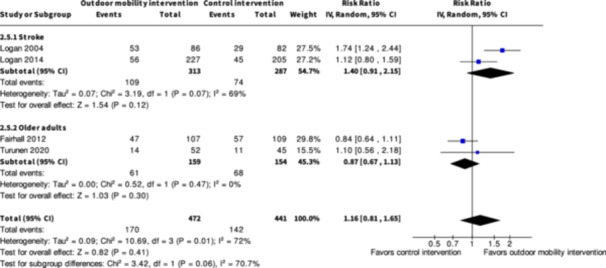
Comparison 2: Specific outdoor mobility interventions versus control interventions—by conditions (Subgroup analyses), Outcome 5: Engagement in everyday life activities ≥7 months.

Subgroup analysis of skill training interventions in people after stroke did not reduce uncertainty and the evidence is very uncertain if skill training interventions may improve participation among people after stroke (SMD: −0.06; 95% CI: −0.20 to 0.31; *I* ^2^ = 55%; 846 participants; six studies; very low certainty evidence; Analysis 2.6). The certainty of evidence was downgraded by one level for very serious risk of bias, one level for very serious imprecision and one level for serious inconsistency.

**Analysis 2.6:**
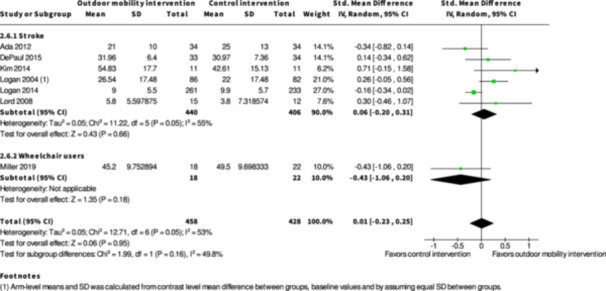
Comparison 2: Specific outdoor mobility interventions versus control interventions—by conditions (Subgroup analyses), Outcome 6: Skill training—Participation ≤6 months.

Subgroup analysis of skill training interventions among people using wheelchairs did not reduce uncertainty and the evidence is very uncertain if skill training interventions may improve participation for people using wheelchairs (SMD: −0.43; 95% CI: −1.06 to 0.20; *I* ^2^ = NA; 40 participants; one study; very low certainty evidence; Analysis 2.6). The certainty of evidence was downgraded by one level for very serious risk of bias, two levels for very serious imprecision.

##### Participation in the longer‐term (≥7 months)

Three studies (Ada, 2012; Logan, 2004; Logan, 2014) reported this outcome, and all were conducted among people after stroke.

Overall, the evidence is very uncertain if skill training interventions improve participation among people living with disabilities in the longer term (SMD: −0.05; 95% CI: −0.31 to 0.20; *I* ^2^ = 54%; 674 participants; three studies; very low certainty evidence; Analysis 1.6). The certainty of evidence was downgraded by one level for very serious risk of bias, one level for serious imprecision and one level for serious inconsistency.

**Analysis 1.6:**
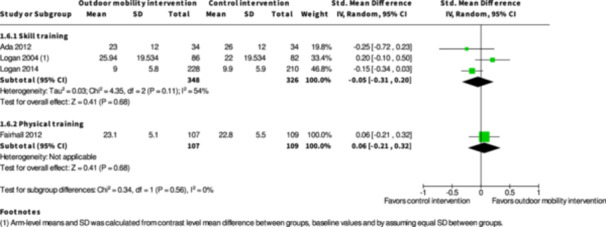
Comparison 1: Specific outdoor mobility interventions versus control interventions, Outcome 6: Participation ≥7 months.

##### Health‐related quality of life in the shorter term (≤6 months)

Four studies (Ada, 2012; Logan, 2014; Rantanen, 2015; Ullrich, 2021) reported this outcome.

Overall, the evidence is very uncertain if skill training interventions improve health‐related quality of life among people living with disabilities in the shorter term (SMD: 0.13; 95% CI: −0.20 to 0.46; *I* ^2^ = 74%; 779 participants; four studies; very low certainty evidence; Analysis 1.7). The certainty of evidence was downgraded by one level for very serious risk of bias, one level for very serious imprecision and one level for serious inconsistency.

**Analysis 1.7:**
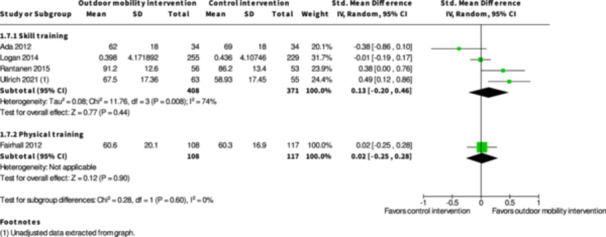
Comparison 1: Specific outdoor mobility interventions versus control interventions, Outcome 7: Health‐related quality of life ≤6 months.

Subgroup analysis of skill training interventions among people after stroke did not reduce uncertainty and the evidence is very uncertain if skill training interventions may improve health‐related quality of life for people after a stroke (SMD: −0.13; 95% CI: −0.47 to 0.21; *I* ^2^ = 52%; 552 participants; two studies; very low certainty evidence; Analysis 2.9). The certainty of evidence was downgraded by one level for very serious risk of bias, one level for very serious imprecision and one level for serious inconsistency.

**Analysis 2.7:**
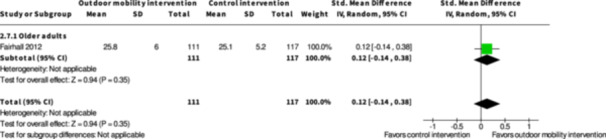
Comparison 2: Specific outdoor mobility interventions versus control interventions—by conditions (Subgroup analyses), Outcome 7: Physical training—Participation ≤6 months.

**Analysis 2.8:**
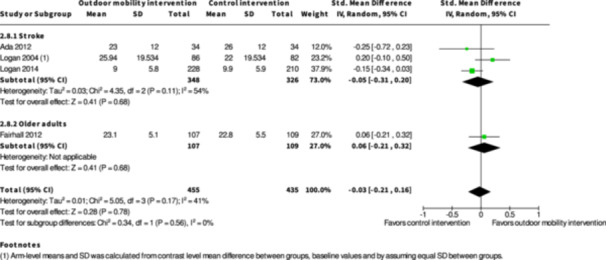
Comparison 2: Specific outdoor mobility interventions versus control interventions—by conditions (Subgroup analyses), Outcome 8: Participation ≥7 months.

**Analysis 2.9:**
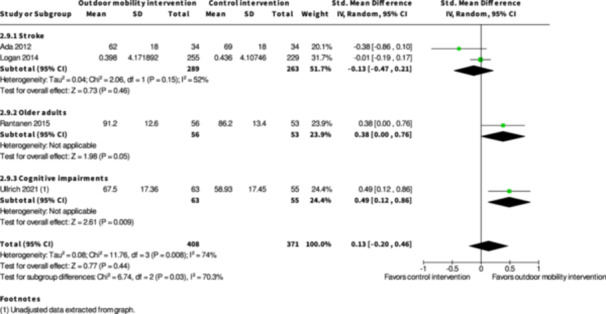
Comparison 2: Specific outdoor mobility interventions versus control interventions—by conditions (Subgroup analyses), Outcome 9: Skill training—Health‐related quality of life ≤6 months.

Subgroup analysis of skill training interventions among people with cognitive impairments reduced uncertainty slightly and skill training interventions may improve health‐related quality of life for people with cognitive impairments (SMD: 0.49; 95% CI: 0.12 to 0.88; *I* ^2^ = NA; 118 participants; one study; low certainty evidence; Analysis 2.9). The certainty of evidence was downgraded by one level for serious risk of bias, one level for serious imprecision.

Subgroup analysis of skill training interventions among older adults with disabilities did not reduce uncertainty and the evidence is very uncertain if skill training interventions may improve health‐related quality of life for older adults living with disabilities (SMD: 0.38; 95% CI: 0.00 to 0.76; *I* ^2^ = NA; 109 participants; one study; very low certainty evidence; Analysis 2.9). The certainty of evidence was downgraded by two levels for very serious risk of bias and one level for serious imprecision.

##### Health‐related quality of life in the longer‐term (≥7 months)

Two studies reported this outcome (Ada, 2012; Logan, 2014), and both were conducted among people after stroke.

Overall, the evidence is very uncertain if skill training interventions improve health‐related quality of life among people living with disabilities in the longer term (SMD: −0.05; 95% CI: −0.23 to 0.13; *I* ^2^ = 0%; 496 participants; two studies; very low certainty evidence; Analysis 1.8). The certainty of evidence was downgraded by two levels for very serious risk of bias and one level for serious imprecision.

**Analysis 1.8:**
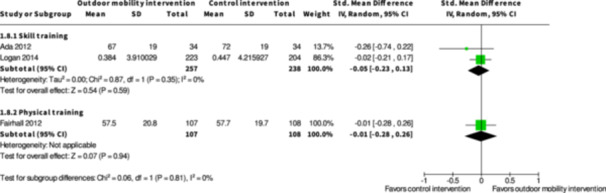
Comparison 1: Specific outdoor mobility interventions versus control interventions, Outcome 8: Health‐related quality of life ≥7 months.

##### Major harms in the shorter term (≤6 months)

Two studies reported this outcome (DePaul, 2015; Lord, 2008) by using: a composite measure of major cardiovascular events, stroke or mortality; and recurrence of stroke as measurements. Both studies were conducted among people after stroke.

Overall, the evidence is very uncertain if skill training interventions lead to major harm among people living with disabilities in the shorter term (RR: 0.83; 95% CI: 0.27 to 2.54; *I* ^2^ = 0%; RD: −0.01; 95% CI: −0.13 to 0.10; 496 participants; two studies; very low certainty evidence; Analysis 1.9). The certainty of evidence was downgraded by one level for very serious risk of bias and two levels for very serious imprecision.

**Analysis 1.9:**
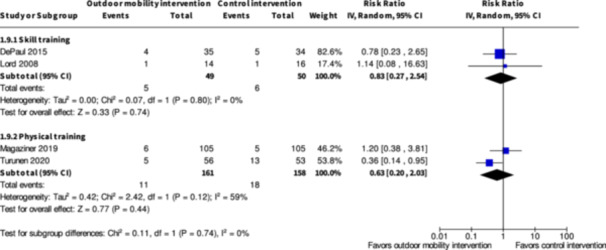
Comparison 1: Specific outdoor mobility interventions versus control interventions, Outcome 9: Major adverse events ≤6 months.

##### Major harms in the longer‐term (≥7 months)

One study reported this outcome (Logan, 2014) by using mortality as a measurement. The study was conducted among people after stroke.

Overall, the evidence is very uncertain if physical training interventions lead to major harm among people living with disabilities in the longer term (RR: 0.98; 95% CI: 0.45 to 2.14; *I* ^2^ = NA; RD: 0.00; 95% CI: −0.03 to 0.03; 568 participants; one study; very low certainty evidence; Analysis 1.10). The certainty of evidence was downgraded by two levels for very serious risk of bias and one level for very serious imprecision.

**Analysis 1.10:**
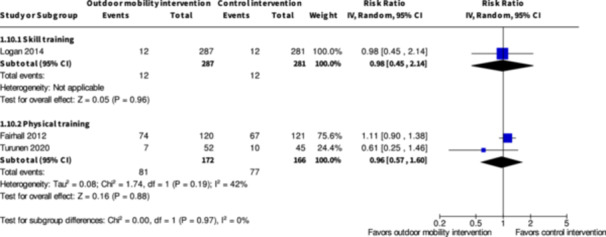
Comparison 1: Specific outdoor mobility interventions versus control interventions, Outcome 10: Major adverse events ≥7 months.

##### Minor harms in the shorter term (≤6 months)

Two studies (Ada, 2012; DePaul, 2015) reported this outcome by using falls as a measurement. Both studies were conducted among people after stroke.

Overall, the evidence is very uncertain if physical training interventions lead to any minor harm among people living with disabilities in the shorter term (RR: 1.14; 95% CI: 0.63 to 2.04; *I* ^2^ = 0%; RD: 0.03; 95% CI: −0.12 to 0.18; 124 participants; two studies study; very low certainty evidence; Analysis 1.11). The certainty of evidence was downgraded by two levels for very serious risk of bias and one level for very serious imprecision.

**Analysis 1.11:**
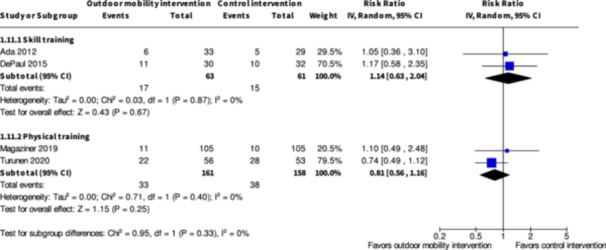
Comparison 1: Specific outdoor mobility interventions versus control interventions, Outcome 11: Minor adverse events ≤6 months.

##### Minor harms in the longer‐term (≥7 months)

Two studies (Ada, 2012; Logan, 2014) reported this outcome by using falls as a measurement. Both studies were conducted among people after stroke.

Overall, the evidence is very uncertain if physical training interventions lead to any minor harm among people living with disabilities in the longer term (RR: 1.00; 95% CI: 0.84 to 1.18; *I* ^2^ = 0%; RD: 0.00; 95% CI: −0.08 to 0.08; 627 participants; two studies study; very low certainty evidence; Analysis 1.12). The certainty of evidence was downgraded by two levels for very serious risk of bias and one level for serious imprecision.

**Analysis 1.12:**
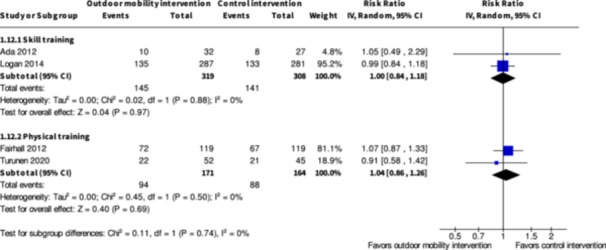
Comparison 1: Specific outdoor mobility interventions versus control interventions, Outcome 12: Minor adverse events ≥7 months.

#### 6.3.2 Comparison 1.2: Physical training interventions versus control intervention not aimed at improving outdoor mobility

##### Activity outside the home in the shorter term (≤6 months)

One study (Fairhall, 2012) reported this outcome, and was conducted among older adults living with disabilities.

Overall physical training interventions may improve activity outside the home in the shorter term (SMD: 0.35; 95% CI: 0.08 to 0.61; *I* ^2^ = NA; 228 participants; one study; low certainty evidence; Analysis 1.1). The certainty of evidence was downgraded by two levels for very serious risk of bias.

##### Activity outside the home in the longer‐term (≥7 months)

One study (Fairhall, 2012) reported this outcome, and was conducted among older adults living with disabilities.

Overall physical training interventions may improve activity outside the home in the longer term (SMD: 0.27; 95% CI: 0.00 to 0.54; *I* ^2^ = NA; 216 participants; one study; low certainty evidence; Analysis 1.2). The certainty of evidence was downgraded by two levels for very serious risk of bias.

##### Engagement in everyday life activities in the shorter term (≤6 months)

Two studies (Fairhall, 2012; Turunen, 2020) reported this outcome, and all were conducted among older adults living with disabilities.

Overall, the evidence is very uncertain if physical training interventions improve engagement in everyday life activities in the shorter term (RR: 1.01; 95% CI: 0.79 to 1.29; *I* ^2^ = 0%; RD: 0.01; 95% CI: −0.10 to 0.11; 337 participants; two studies; very low certainty evidence; Analysis 1.3). The certainty of evidence was downgraded by two levels for very serious risk of bias and one level for serious imprecision.

##### Engagement in everyday life activities in the longer‐term (≥7 months)

Two studies (Fairhall, 2012; Turunen, 2020) reported this outcome, and all were conducted among older adults living with disabilities.

Overall, the evidence is very uncertain if physical training interventions improve engagement in everyday life activities in the longer term (RR: 0.87; 95% CI: 0.67 to 1.13; *I* ^2^ = 0%; RD: −0.04; 95% CI: −0.15 to 0.06; 313 participants; two studies; very low certainty evidence; Analysis 1.4). The certainty of evidence was downgraded by two levels for very serious risk of bias and one level for serious imprecision.

##### Participation in the shorter term (≤6 months)

One study (Fairhall, 2012) reported this outcome, and was conducted among older adults living with disabilities.

Overall, the evidence is very uncertain if physical training interventions improve participation in the shorter term (SMD: 0.12; 95% CI: −0.14 to 0.38; *I* ^2^ = NA; 228 participants; one study; very low certainty evidence; Analysis 1.5). The certainty of evidence was downgraded by two levels for very serious risk of bias and one level for serious imprecision.

##### Participation in the longer‐term (≥7 months)

One study (Fairhall, 2012) reported this outcome, and was conducted among older adults living with disabilities.

Overall, the evidence is very uncertain if physical training interventions improve participation in the longer term (SMD: 0.06; 95% CI: −0.21 to 0.32; *I* ^2^ = NA; 216 participants; one study; very low certainty evidence; Analysis 1.6). The certainty of evidence was downgraded by two levels for very serious risk of bias and one level for serious imprecision.

##### Health‐related quality of life in the shorter term (≤6 months)

One study (Fairhall, 2012) reported this outcome, and was conducted among older adults living with disabilities.

Overall, the evidence is very uncertain if physical training interventions improve participation in the shorter term (SMD: 0.02; 95% CI: −0.25 to 0.28; *I* ^2^ = NA; 225 participants; one study; very low certainty evidence; Analysis 1.7). The certainty of evidence was downgraded by two levels for very serious risk of bias and one level for serious imprecision.

##### Health‐related quality of life in the longer‐term (≥7 months)

One study (Fairhall, 2012) reported this outcome, and was conducted among older adults living with disabilities.

Overall, the evidence is very uncertain if physical training interventions improve participation in the longer term (SMD: −0.01; 95% CI: −0.28 to 0.26; *I* ^2^ = NA; 215 participants; one study; very low certainty evidence; Analysis 1.8). The certainty of evidence was downgraded by two levels for very serious risk of bias and one level for serious imprecision.

##### Major harms in the shorter term (≤6 months)

Two studies (Magaziner, 2019; Turunen, 2020) reported this outcome using: a composite score of severe‐, life‐threatening‐ or fatal harms; and need of emergency room service.

Overall, the evidence is very uncertain if physical training interventions lead to major harm among people living with disabilities in the shorter term (RR: 0.63; 95% CI: 0.20 to 2.03; *I* ^2^ = 59%; RD: −0.06; 95% CI: −0.22 to 0.10; 317 participants; two studies; very low certainty evidence; Analysis 1.9). The certainty of evidence was downgraded by one level for very serious risk of bias, one level for serious imprecision and one level for serious inconsistency.

Subgroup analysis of physical training interventions in older adults living with disabilities did not reduce uncertainty and the evidence is very uncertain if physical training interventions lead to major harm among older adults living with disabilities (RR: 0.36; 95% CI: 0.14 to 0.95; *I* ^2^ = NA; RD: −0.16; 95% CI: −0.29 to −0.02; 109 participants; one study; very low certainty evidence; Analysis 2.12). The certainty of evidence was downgraded by two levels for very serious risk of bias, and one level for serious imprecision.

**Analysis 2.10:**
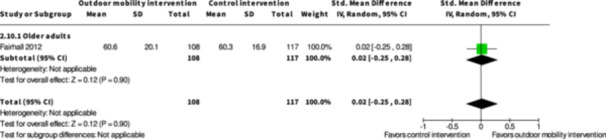
Comparison 2: Specific outdoor mobility interventions versus control interventions—by conditions (Subgroup analyses), Outcome 10: Physical training—Health‐related quality of life ≤6 months.

**Analysis 2.12:**
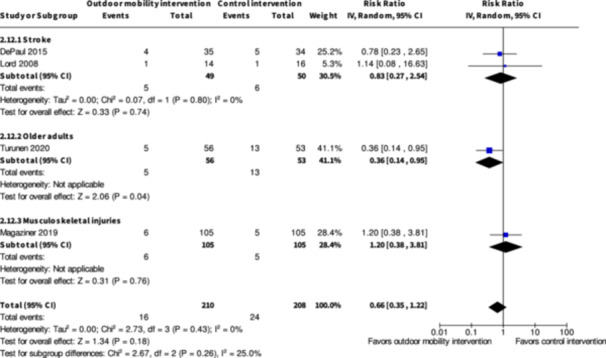
Comparison 2: Specific outdoor mobility interventions versus control interventions—by conditions (Subgroup analyses), Outcome 12: Major adverse events (Dichotomous measures) ≤6 months.

Subgroup analysis of physical training interventions in people with musculoskeletal injuries did not reduce uncertainty and the evidence is very uncertain if physical training interventions lead to major harm among people with musculoskeletal injuries (RR: 1.20; 95% CI: 0.38 to 3.81; *I* ^2^ = NA; RD: −0.01; 95% CI: −0.05 to 0.07; 210 participants; one study; very low certainty evidence; Analysis 2.12). The certainty of evidence was downgraded by one level for serious risk of bias, and two levels for very serious imprecision.

##### Major harms in the longer‐term (≥7 months)

Two studies (Magaziner, 2019; Turunen, 2020) reported this outcome using: a composite score of severe‐, life‐threatening‐ or fatal harms; and need of emergency room service.

Overall, the evidence is very uncertain if physical training interventions lead to major harm among older people living with disabilities in the longer term (RR: 0.96; 95% CI: 0.57 to 1.60; *I* ^2^ = 42%; RD: −0.01; 95% CI: −0.15 to 0.14; 338 participants; two studies; very low certainty evidence; Analysis 1.10). The certainty of evidence was downgraded by two levels for very serious risk of bias, and one level for serious imprecision.

##### Minor harms in the shorter term (≤6 months)

Two studies (Magaziner, 2019; Turunen, 2020) reported this outcome using: need for healthcare services; and a composite score of mild and moderate harms that included falls. Both studies were conducted among older adults with disabilities.

Overall, the evidence is very uncertain if physical training interventions lead to major harm among people living with disabilities in the shorter term (RR: 0.81; 95% CI: 0.56 to 1.16; *I* ^2^ = 0%; RD: −0.04; 95% CI: −0.17 to 0.10; 319 participants; two studies; very low certainty evidence; Analysis 1.11). The certainty of evidence was downgraded by two levels for very serious risk of bias, and one level for serious imprecision.

##### Minor harms in the longer‐term (≥7 months)

Two studies (Fairhall, 2012; Turunen, 2020) reported this outcome using: need for healthcare services; and a composite score of mild and moderate harms that included falls. Both studies were conducted among older adults with disabilities.

Overall, the evidence is very uncertain if physical training interventions lead to major harm among people living with disabilities in the shorter term (RR: 1.04; 95% CI: 0.86 to 1.26; *I* ^2^ = 0%; RD: 0.02; 95% CI: −0.09 to 0.12; 182 participants; two studies; very low certainty evidence; Analysis 1.12). The certainty of evidence was downgraded by two levels for very serious risk of bias, and one level for serious imprecision.

#### 6.3.3 Comparison 2: Skill training interventions of different length

One study (Ada, 2012) compared two different skill training interventions for people with stroke which differed in length of the intervention period (3 sessions a week over 8 weeks versus 3 sessions a week over 16 weeks) and reported this outcome. Analysis 2.7, 2.8, Analysis 2.8, 2.10, 2.11, 2.13, 2.14, 2.15.

**Analysis 2.11:**
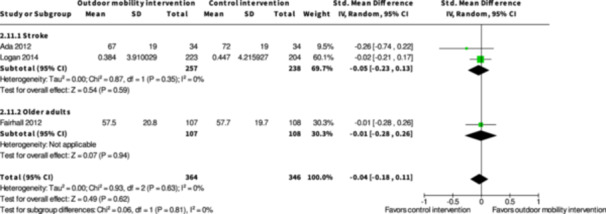
Comparison 2: Specific outdoor mobility interventions versus control interventions—by conditions (Subgroup analyses), Outcome 11: Health‐related quality of life ≥7 months.

**Analysis 2.13:**
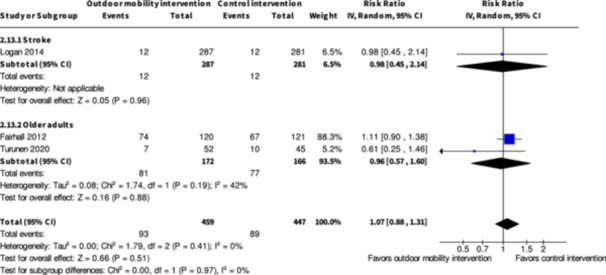
Comparison 2: Specific outdoor mobility interventions versus control interventions—by conditions (Subgroup analyses), Outcome 13: Major adverse events (Dichotomous measures) ≥7 months.

**Analysis 2.14:**
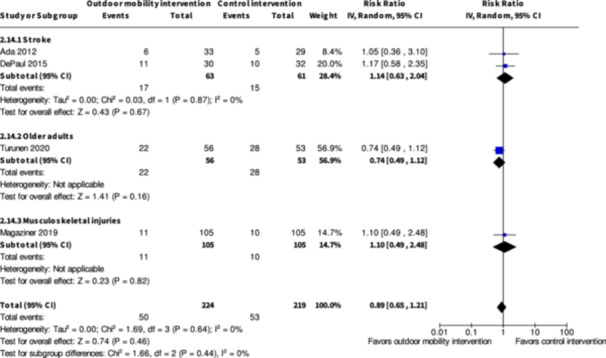
Comparison 2: Specific outdoor mobility interventions versus control interventions—by conditions (Subgroup analyses), Outcome 14: Minor adverse events (Dichotomous measures) ≤6 months.

**Analysis 2.15:**
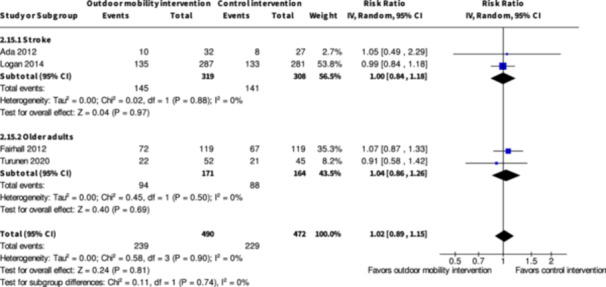
Comparison 2: Specific outdoor mobility interventions versus control interventions—by conditions (Subgroup analyses), Outcome 15: Minor adverse events (Dichotomous measures) ≥7 months.

##### Participation in the shorter term (≤6 months)

Overall, the evidence is very uncertain if skill training interventions of different intensity have an impact on participation in the shorter term (SMD: 0.00; 95% CI: −0.48 to 0.48; *I* ^2^ = NA; 68 participants; one study; very low certainty evidence; Analysis 3.1). The certainty of evidence was downgraded by one level for serious risk of bias, and two levels for very serious imprecision.

**Analysis 3.1:**
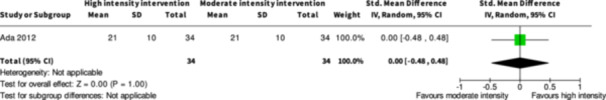
Comparison 3: Different intensity of outdoor mobility interventions, Outcome 1: Participation ≤6 months.

##### Participation in the longer‐term (≥7 months)

Overall, the evidence is very uncertain if skill training interventions of different intensity have an impact on participation in the longer term (SMD: 0.09; 95% CI: −0.39 to 0.57; *I* ^2^ = NA; 68 participants; one study; very low certainty evidence; Analysis 3.2). The certainty of evidence was downgraded by one level for serious risk of bias, and two levels for very serious imprecision.

**Analysis 3.2:**
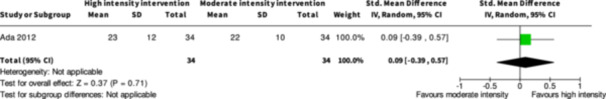
Comparison 3: Different intensity of outdoor mobility interventions, Outcome 2: Participation ≥7 months.

##### Health‐related quality of life in the shorter term (≤6 months)

Overall, the evidence is very uncertain if skill training interventions of different intensity have an impact on health‐related quality of life in the shorter term (SMD: −0.12; 95% CI: −0.59 to 0.36; *I* ^2^ = NA; 68 participants; one study; very low certainty evidence; Analysis 3.3). The certainty of evidence was downgraded by one level for serious risk of bias, and two levels for very serious imprecision.

**Analysis 3.3:**
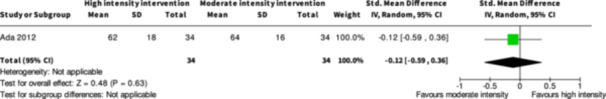
Comparison 3: Different intensity of outdoor mobility interventions, Outcome 3: Health‐related quality of life ≤6 months.

##### Health‐related quality of life in the longer‐term (≥7 months)

Overall, the evidence is very uncertain if skill training interventions of different intensity have an impact on health‐related quality of life in the longer term (SMD: 0.20; 95% CI: −0.27 to 0.68; *I* ^2^ = NA; 68 participants; one study; very low certainty evidence; Analysis 3.4). The certainty of evidence was downgraded by one level for serious risk of bias, and two levels for very serious imprecision.

**Analysis 3.4:**
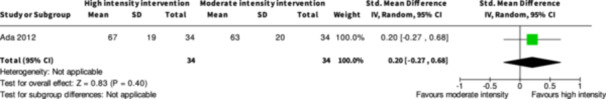
Comparison 3: Different intensity of outdoor mobility interventions, Outcome 4: Health‐related quality of life ≥7 months.

##### Minor harms in the shorter term (≤6 months)

Overall, the evidence is very uncertain if skill training interventions of different intensity have an impact on minor adverse events in the shorter term (RR: 5.45; 95% CI: 0.70 to 42.73; *I* ^2^ = NA; RD: 0.15; 95% CI: 0.00 to 0.29; 68 participants; one study; very low certainty evidence; Analysis 3.5). The certainty of evidence was downgraded by one level for serious risk of bias, and two levels for very serious imprecision.

**Analysis 3.5:**
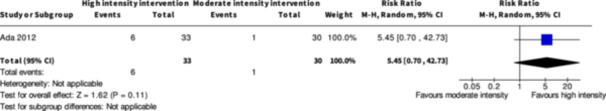
Comparison 3: Different intensity of outdoor mobility interventions, Outcome 5: Minor adverse events ≤6 months.

##### Minor harms in the longer‐term (≥7 months)

Overall, the evidence is very uncertain if skill training interventions of different intensity have an impact on minor adverse events in the shorter term (RR 0.60; 95% CI 0.32 to 1.13; I^2^ = NA; RD −0.21; 95% CI −0.45 to 0.04; 68 participants; one study; very low certainty evidence; Analysis 3.6). The certainty of evidence was downgraded by one level for serious risk of bias, and two levels for very serious imprecision.

**Analysis 3.6:**
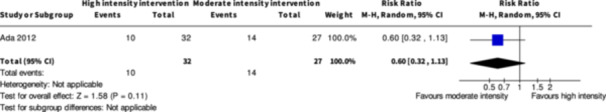
Comparison 3: Different intensity of outdoor mobility interventions, Outcome 6: Minor adverse events ≥7 months.

#### 6.3.4 Subgroup analyses

Due to a limited number of studies conducted for each outcome and type of intervention in combination with a substantial risk of bias, there would be very limited confidence in any results of subgroup analyses when assessed with the ICEMAN tool for the credibility of subgroup effects (Schandelmaier, 2020). Hence, we did not conduct any subgroup analyses for: risk of bias; different control interventions; different intervention areas; different intervention delivery; different intervention duration; different intervention mobility modes; and different intervention settings.

#### 6.3.5 Sensitivity analyses

Removing outcome measures being reported by subscales or parts of measurements: One study, Logan, 2014, measured and presented only subscales of SF36, where Social Functioning was one of the primary outcomes and was included in our primary analyses. The study did not present any formal analyses of full scores or other sub‐scores of SF36, but states that ‘The other domains from the SF‐36v2 were also analysed at 6 months and showed no significant difference between groups’, which was not different from the initial analysis done and did not change our conclusions.

As either all, or none, of the included studies were excluded from the pre‐planned sensitivity analyses, it was not feasible to conduct sensitivity analyses related to: Removing studies using ‘per protocol’ or ‘as treated’ analysis; Removing studies having a high risk of bias due to missing outcome data; Including the reported outcomes for the second active intervention arm; Including the second reported outcome measure in studies reporting multiple outcome measures for the same outcome; Removing studies where environmental interventions are combined with interventions to increase individuals’ capacity and performance, or; Removing studies using balance training as an intervention for the outcome of adverse events, including falls.

## 7 DISCUSSION

### 7.1 Summary of main results

Overall, based on this review, conclusions can only be drawn on low and very low certainty evidence, leading to limited confidence in any reported effect estimate for any benefits or harms of outdoor mobility interventions for people living with disabilities.

Furthermore, all effect estimates across all outcomes had small or trivial effect sizes when translated according to Cohen's *d* (Sullivan, 2012). The largest synthesized effect estimate for beneficial continuous outcomes was a 0.49 standardized mean difference for skill training for older adults for health‐related quality of life. For dichotomous outcomes, the largest effect estimate is a risk difference between groups of 0.15 and or a risk ratio of 1.46 for skill training for people living with disabilities for engagement in everyday life activities. This can be translated to a number needed to treat (NNT) of seven person recieving the intervention to get one person to benefit.

A few interventions may impact some outcomes in the short term. This includes skill training interventions that may improve engagement in everyday life activities among people living with disabilities and physical training interventions that may improve activity outside the home. However, caution has to be taken when interpreting these results due to low certainty evidence that could impact the reported results and the uncertainty about the impact in a longer term than 6 months. A minority of the included studies reported on any harm or adverse events. Based on overall very low certainty evidence, there were no clear differences in minor or major harms for any outdoor mobility interventions, but all confidence intervals include both some reduction and some increase of harms.

Based on the certainty of the evidence and the limited effects sizes reported, the evidence is uncertain if there are interventions assessed by RCT designs that can impact outdoor mobility for people living with disabilities, lead to other beneficial outcomes and do not lead to any harms.

### 7.2 Overall completeness and applicability of evidence

Despite the comprehensive search process we did not identify any studies exploring educational interventions or cognitive, behavioural or other psychological training interventions.

Furthermore, no studies explored comprehensive multi‐component interventions delivered by multi‐professional teams. The use of multiple mobility modes (e.g., buses, trams and trains) was only used by two interventions to improve outdoor mobility. In addition, all interventions were delivered during maximum 12 weeks and did not include any structured motivational or ‘refresher’‐sessions after the intervention period. Such knowledge gaps could be explored in future studies to improve the completeness of the evidence. Furthermore, identifying and exploring mechanisms, effect modifiers and ways of delivering interventions that could impact outdoor mobility in the long term is needed to find effective interventions. Systems for developing complex interventions could be helpful in this regard (Skivington, 2021). We identified a total of 12 ongoing studies of interventions to improve outdoor mobility which could improve the certainty of evidence of future systematic reviews and fill current knowledge gaps.

Most studies were conducted in populations after stroke, or among older adults living with disabilities. Although outdoor mobility issues have been described in other populations, no interventions were identified in populations with mental, behavioural or neurodevelopmental disorders, or with diseases of the visual system, circulatory system, respiratory system or after injuries were identified. Hence, only indirect evidence for benefits and harms of outdoor mobility interventions are available for most people living with disabilities. Future research could include populations without restricting enrolment to specific disease classifications, and specifically target populations with limited outdoor mobility, irrespective of condition to create evidence for people living with disabilities. This would fill several knowledge gaps and could be utilized to guide decision‐making.

The interventions were delivered in a variety of healthcare systems within different countries, and only one study was conducted in a low‐income country, that is, Philippines (Mendoza, 2015). Contextual differences could limit the applicability, implementation and use of these interventions in contexts that differ substantially from the original study. Because of the insufficiency of available data, further exploration of the impact of outdoor mobility interventions on health equity was not possible. This is in accordance with a previous scoping review exploring health equity characteristics in studies of unmet community mobility needs for older adults, which identified poor inclusion and reporting of characteristics to evaluate and follow‐up aspects of health equity in the evidence base (Biljon, 2022).

The very low reporting of any potential harms (e.g., falls, hospitalization and mortality) in the included studies limits the completeness and usage of the evidence in decision‐making. Even though a few studies transparently addressed potential harms of the interventions and found no substantial difference between intervention and control group, it is still uncertain of this is an accurate representation of the harms that outdoor mobility interventions might lead to based on risk of bias and underpowered studies to detect any differences.

Only one study compared different delivery of interventions aimed to improve outdoor mobility, in terms of different length of the same intervention (3 sessions/week during 8 weeks or during 16 weeks). Further studies are needed to reliably compare different intervention components and intervention delivery modes to identify potential effect moderators.

Only one intervention was replicated in two different studies (Logan, 2004; Logan, 2014). The large effect size in Logan, 2004 was not reproduced in the larger multicentre study Logan, 2014. This discrepancy could be due to many reasons, including risk of bias in the different studies, differences in the professionals delivering the intervention, or including a population with more time since the stroke onset and possibly more difficulties in outdoor mobility at baseline. This could indicate that outdoor mobility interventions delivered closer to the ‘real world’ context in the multicentre study could have a different impact than in the initial study. Complex interventions include many different components and there are many aspects that can be influenced when settings are changed or expanded. Thus, future studies of interventions that are conducted need to be designed with the end setting and end users in mind to have reliable evidence of benefits and harms and to be successfully implemented in practice. This should be taken into consideration when applying evidence in practice or when recommendations are made in guidelines based on smaller studies conducted in different settings than the one intended.

### 7.3 Quality of the evidence

#### 7.3.1 Current quality and uncertainties in the evidence base

The vast majority of the studies and interventions had an overall high risk of bias for almost all outcomes due to methodological limitations in several domains. Missing outcome data, possibly due to the longer follow‐up periods and difficulties keeping participants in the trial, where people in the control group dropped out to a larger extent, are the most notable reasons. Additionally, half of the interventions used inactive control groups receiving no treatment or unspecified treatment as usual, which often introduced a risk of bias. Additional risks of bias were often introduced in the reporting of the subjectively participant‐reported outcome measurements due to knowledge and possible preference of the group allocation.

Most of the included studies, albeit with clearly stated aims to improve outdoor mobility, did not assess if this was achieved or not because of lack of any type of outdoor mobility measure. Instead, surrogate outcomes of unclear predictive markers such as measures of walking speed or measures of strength and endurance of the lower extremities were used. Consequently, it was not possible to assess if increased outdoor mobility was achieved. Future studies should aim to include valid measures of outdoor mobility to evaluate if improvements in surrogate measurements also improve outdoor mobility. To be able to evaluate any potential beneficial effects of increased outdoor mobility, such as engagement in activities, quality of life or participation, such outcome measures should be included in future trials. Turning to another quality aspect, most of the included studies enroled few participants and were not powered to detect important estimates of either benefits or harms, resulting in wide confidence intervals and further uncertainty about potential effects. When several studies and their included interventions reported the same outcome, the reported results often had substantial or considerable heterogeneity in the reported effects, adding to the uncertainty about the actual effectiveness of the outdoor mobility interventions. Due to the many potential reasons for this heterogeneity and few included studies reporting on each outcome we were unable to confidently explore and explain these differences.

Due to the low number of included studies reporting on each outcome, funnel plots were not useful to assess any publication bias, which leads to uncertainties regarding the presence of any selective publication that could impact our conclusions.

#### 7.3.2 Improvements in future studies

To improve the certainty of evidence, future studies should be conducted in the context they are supposed to be delivered in by professionals working in these contexts. For example, in multicenter studies including clinics outside of university hospital settings and in close collaboration with clinical units to enrol relevant participants that reflect the actual target population of interest. Collaborations between researchers, clinics and healthcare professionals are likely needed to create reliable evidence and enrol enough participants to identify important benefits and harms of interventions. But this is often difficult due to limitations in funding and often a lack of large scale coordination within the healthcare systems and the clinicians who meet, enrol and support the heterogeneous group of people living with disabilities.

To reduce the uncertainty related to very imprecise effect estimates, future studies should use strategies to reduce attrition and be powered to detect changes in outdoor mobility as well as other outcomes, including harms, in the long term. To improve enrolment and avoid trials using very small sample sizes, outdoor mobility interventions could be implemented and evaluated as part of standard clinical practice. This could improve the number of enroled participants, and lead to more useful evidence due to similar settings and delivery being used in both research and practice.

Careful planning of the randomization process, including allocation concealment procedures to avoid any potential biases and reporting these strategies is needed to improve assessments of the randomization procedure and possible impact on the outcomes. Strategies to deal with potential baseline imbalances between intervention groups that can happen due to chance in smaller studies, could be improved by, for example, plans for adjustments for specific covariates or sensitivity analyses.

Using more rigorous control interventions is needed to reduce bias due to deviation from intended intervention and would improve our confidence in the evidence. Complete blinding using a placebo is not feasible in outdoor mobility interventions research, but matching intervention length or actual time spent with an interventionist, and avoiding using ‘no treatment’ controls would limit the risk of bias. Of note, Miller, 2019 designed an active control intervention using seminars of only the theoretical skills needed for outdoor mobility in combination with health promotion ‐ but without any practical application of skill training. In addition, studies following up and comparing differences in health behaviours or use of additional care that could impact the outcomes between groups would mitigate the risk of bias.

Creating and openly sharing detailed pre‐specified analysis plans would improve our certainty of the evidence and limit any potential risks of bias of selective reporting. Finally, clear reporting of the actual intervention and the delivery is needed to access specific mechanisms of the interventions and compare these across different studies.

Additional implications for research is described in Authors’ conclusions section.

### 7.4 Potential biases in the review process

No additional studies were identified during the screening of other systematic reviews reference list or within reference lists of included studies, making us more confident that our search strategy was comprehensive and sensitive. All steps in the review process (screening, data extraction, risk of bias and GRADE) were made in duplicate, thus minimizing the potential bias in the selection, data extraction and assessment processes.

One part of the review process where potential bias could have been introduced would be in studies with limited information, unclear descriptions of whether the majority of participants had disabilities, and whether the aim to improve outdoor mobility was clear. Clear descriptions of the interventions, their components and delivery are needed to access and fully understand the content in outdoor mobility interventions. We opted for a conservative approach that would limit uncertainty and potential bias by exclusion of such studies. For example, we specified that a clear sentence reflecting an outdoor mobility focus of the intervention had to be presented. If more flexible or broader criteria could lead to more included studies, but subsequently more heterogeneity in the design of the included interventions, in similar reviews.

Within each of our intervention categories (i.e., skill training or physical training), there are a variety of modes of delivery and intervention structure. Highlighting that these are complex interventions with the potential of many different effect modifiers can add some limitations to the generalizability of our findings to other interventions. For example, in the studies by Logan et al. (Logan, 2004, Logan, 2014) the interventions were very similar in content and delivery, but found very different effects on important outcomes. This highlights how possible small differences in intervention delivery, populations or study design limitations could impact potential effect estimates and thereby be difficult to transfer to similar or other contexts.

Furthermore, in this review we only included outcomes deemed directly important for persons living with disabilities, and avoided surrogate outcomes and indirect indicators of outdoor mobility. We did not include potential indirect or surrogate outcome measures assessing, for example, walking speed or speed of movement (e.g., 10‐Meter‐Walk‐Test [10MWT], Timed up and Go‐test [TUG]) or balance tests (e.g., Bergs Balance Scale, Activities‐specific balance confidence scale) or sit‐stand speed from a chair. All the reported outcome measures in each study are reported in Table 1 if considered important to explore in further research.

We used standardized mean difference to be able to summarize and compare measurements in the same outcome category and most studies used different outcome measures to examine the same outcome. The standardized mean difference relies on the assumption that the different outcome scales have similar distributions in the scores, and that the variability in reported outcomes between populations is similar, which is not always clear and could introduce bias. Efforts to use the same outcome measures between studies would facilitate comparisons between studies and could improve future synthesis.

### 7.5 Reviews

This review is, to our knowledge, the first exploring outdoor mobility interventions delivered in RCTs for all populations with disabilities and including important outcomes for people living with disabilities, such as health‐related quality of life, engagement in activities outside, participation and harm.

One related but more narrowly focused systematic review assessed community‐based rehabilitation interventions after hip fracture incorporating outdoor mobility components but assessed only ambulatory ability and falls‐related self‐efficacy (Sheehan, 2021). The review found, similar to our results, very low certainty evidence for the chosen outcomes and the synthesized effect sizes were overall small. A recently systematic review explored community ambulation interventions taking place outdoors targeting older adults (Bhatia, 2022), focusing on the impact on physical and mental health. Five studies were identified, with very low or low certainty evidence of small or trivial effects. The authors recommended further research with a focus on robust designs and possibilities to implement the interventions in healthcare systems.

Yet another systematic review assessed interventions for improving community ambulation after stroke (Barclay, 2015). Five trials were identified, but due to insufficient evidence it was not possible to assess the efficacy of the interventions. The fact that we identified 13 trials addressing the same population highlights that outdoor mobility interventions targeting the stroke population is an active research topic, but there is room for improvement regarding the quality and conduct of future studies.

## 8 AUTHORS’ CONCLUSIONS

### 8.1 Implications for practice and policy

Twenty‐two studies exploring interventions to improve outdoor mobility for adults living with disabilities were identified. There is uncertainty about most benefits and harms of these interventions, both in the short and long term. Furthermore, the reported effects of these interventions were considered either small or trivial, and could be of limited relevance to people living with disabilities. Thus, the evidence is currently uncertain if there are available interventions that can improve outdoor mobility for people living with disabilities and other important outcomes, while avoiding harm.

Any recommendations and clinical use of outdoor mobility interventions need to acknowledge the inherent uncertainty of the benefits and harms. Furthermore, any implementation of outdoor mobility interventions also needs to take into account the transferability of the interventions by considering contextual effect modifiers between the different contexts. For instance, differences in the healthcare systems, intervention delivery, intensity and intervention deliverers. Future studies on interventions to improve outdoor mobility could be evaluated within a clinical practice context to improve the certainty of evidence and subsequently guide decision‐making and policy.

### 8.2 Implications for research

To improve the certainty of evidence to facilitate decision‐making, future studies need to include enough participants to detect changes in outcomes of benefits and harms, and to actually include, assess and report these important outcomes. Upcoming research should use outcome measures that are reliable and preferable, and studies should use the same outcome measures for each important outcome to facilitate future evidence synthesis. Furthermore, future studies should consider evaluating outcomes in the longer term since this is often the main target interventions strive to improve.

There needs to be a careful consideration of factors that can introduce bias in future outdoor mobility intervention research. Strategies on how to deal with substantial baseline differences between groups, designing and using control interventions to reduce potential bias (e.g., attention control interventions), strategies to reduce attrition and participants ‘lost‐to‐follow‐up’ and implementing analyses to assess the impact of these losses (e.g., imputation‐techniques and sensitivity analyses), is needed to reduce bias concerns.

To improve the directness of research evidence, future studies should also enrol participants that reflect the actual target population of interest. Inventions should be delivered by professionals who are or will engage with patients in clinical practice and be conducted in contexts where the intervention can be delivered and implemented.

To fill knowledge gaps where currently no evidence is available, future studies could focus on other approaches than skill training and physical training to improve outdoor mobility. This could include new comprehensive interventions programs containing many different approaches delivered by several healthcare professionals in collaboration, interventions incorporated as additions to a comprehensive intervention program already in use, or interventions that include longer term delivery (e.g., more than 4 months delivery of the intervention).

## DATA AND ANALYSES

**Comparison 1** Specific outdoor mobility interventions versus control interventions

**Table:** 

Outcome or subgroup title	No. of studies	No. of participants	Statistical method	Effect size
1.1 Activity outside the home ≤6 months	7		Std. Mean Difference (IV, Random, 95% CI)	Subtotals only
1.1.1 Skill training	6	925	Std. Mean Difference (IV, Random, 95% CI)	0.18 [−0.20, 0.56]
1.1.2 Physical training	1	228	Std. Mean Difference (IV, Random, 95% CI)	0.35 [0.08, 0.61]
1.2 Activity outside the home ≥7 months	3		Std. Mean Difference (IV, Random, 95% CI)	Subtotals only
1.2.1 Skill training	2	672	Std. Mean Difference (IV, Random, 95% CI)	0.38 [−0.55, 1.30]
1.2.2 Physical training	1	216	Std. Mean Difference (IV, Random, 95% CI)	0.27 [0.00, 0.54]
1.3 Engagement in everyday life activities ≤6 months	5		Risk Ratio (IV, Random, 95% CI)	Subtotals only
1.3.1 Skill training	3	692	Risk Ratio (IV, Random, 95% CI)	1.46 [1.16, 1.84]
1.3.2 Physical training	2	337	Risk Ratio (IV, Random, 95% CI)	1.01 [0.79, 1.29]
1.4 Engagement in everyday life activities ≥7 months	4		Risk Ratio (IV, Random, 95% CI)	Subtotals only
1.4.1 Skill training	2	600	Risk Ratio (IV, Random, 95% CI)	1.40 [0.91, 2.15]
1.4.2 Physical training	2	313	Risk Ratio (IV, Random, 95% CI)	0.87 [0.67, 1.13]
1.5 Participation ≤6 months	8		Std. Mean Difference (IV, Random, 95% CI)	Subtotals only
1.5.1 Skill training	7	886	Std. Mean Difference (IV, Random, 95% CI)	0.01 [−0.23, 0.25]
1.5.2 Physical training	1	228	Std. Mean Difference (IV, Random, 95% CI)	0.12 [−0.14, 0.38]
1.6 Participation ≥7 months	4		Std. Mean Difference (IV, Random, 95% CI)	Subtotals only
1.6.1 Skill training	3	674	Std. Mean Difference (IV, Random, 95% CI)	−0.05 [−0.31, 0.20]
1.6.2 Physical training	1	216	Std. Mean Difference (IV, Random, 95% CI)	0.06 [−0.21, 0.32]
1.7 Health‐related quality of life ≤6 months	5		Std. Mean Difference (IV, Random, 95% CI)	Subtotals only
1.7.1 Skill training	4	779	Std. Mean Difference (IV, Random, 95% CI)	0.13 [−0.20, 0.46]
1.7.2 Physical training	1	225	Std. Mean Difference (IV, Random, 95% CI)	0.02 [−0.25, 0.28]
1.8 Health‐related quality of life ≥7 months	3		Std. Mean Difference (IV, Random, 95% CI)	Subtotals only
1.8.1 Skill training	2	495	Std. Mean Difference (IV, Random, 95% CI)	−0.05 [−0.23, 0.13]
1.8.2 Physical training	1	215	Std. Mean Difference (IV, Random, 95% CI)	−0.01 [−0.28, 0.26]
1.9 Major adverse events ≤6 months	4		Risk Ratio (IV, Random, 95% CI)	Subtotals only
1.9.1 Skill training	2	99	Risk Ratio (IV, Random, 95% CI)	0.83 [0.27, 2.54]
1.9.2 Physical training	2	319	Risk Ratio (IV, Random, 95% CI)	0.63 [0.20, 2.03]
1.10 Major adverse events ≥7 months	3		Risk Ratio (IV, Random, 95% CI)	Subtotals only
1.10.1 Skill training	1	568	Risk Ratio (IV, Random, 95% CI)	0.98 [0.45, 2.14]
1.10.2 Physical training	2	338	Risk Ratio (IV, Random, 95% CI)	0.96 [0.57, 1.60]
1.11 Minor adverse events ≤6 months	4		Risk Ratio (IV, Random, 95% CI)	Subtotals only
1.11.1 Skill training	2	124	Risk Ratio (IV, Random, 95% CI)	1.14 [0.63, 2.04]
1.11.2 Physical training	2	319	Risk Ratio (IV, Random, 95% CI)	0.81 [0.56, 1.16]
1.12 Minor adverse events ≥7 months	4		Risk Ratio (IV, Random, 95% CI)	Subtotals only
1.12.1 Skill training	2	627	Risk Ratio (IV, Random, 95% CI)	1.00 [0.84, 1.18]
1.12.2 Physical training	2	335	Risk Ratio (IV, Random, 95% CI)	1.04 [0.86, 1.26]

**Comparison 2** Specific outdoor mobility interventions versus control interventions—by conditions (Subgroup analyses)

**Table:** 

Outcome or subgroup title	No. of studies	No. of participants	Statistical method	Effect size
2.1 Skill training—Activity outside the home ≤6 months	6	925	Std. Mean Difference (IV, Random, 95% CI)	0.18 [−0.20, 0.56]
2.1.1 Stroke	3	739	Std. Mean Difference (IV, Random, 95% CI)	0.26 [−0.36, 0.88]
2.1.2 Wheelchair users	2	68	Std. Mean Difference (IV, Random, 95% CI)	−0.18 [−0.66, 0.30]
2.1.3 Cognitive impairments	1	118	Std. Mean Difference (IV, Random, 95% CI)	0.44 [0.07, 0.81]
2.2 Physical training—Activity outside the home ≤6 months	1	228	Std. Mean Difference (IV, Random, 95% CI)	0.35 [0.08, 0.61]
2.2.1 Older adults	1	228	Std. Mean Difference (IV, Random, 95% CI)	0.35 [0.08, 0.61]
2.3 Activity outside the home ≥7 months	3	888	Std. Mean Difference (IV, Random, 95% CI)	0.34 [−0.19, 0.86]
2.3.1 Stroke	2	672	Std. Mean Difference (IV, Random, 95% CI)	0.38 [−0.55, 1.30]
2.3.2 Older adults	1	216	Std. Mean Difference (IV, Random, 95% CI)	0.27 [0.00, 0.54]
2.4 Engagement in everyday life activities ≤6 months	5	1029	Risk Ratio (IV, Random, 95% CI)	1.25 [0.99, 1.59]
2.4.1 Stroke	3	692	Risk Ratio (IV, Random, 95% CI)	1.46 [1.16, 1.84]
2.4.2 Older adults	2	337	Risk Ratio (IV, Random, 95% CI)	1.01 [0.79, 1.29]
2.5 Engagement in everyday life activities ≥7 months	4	913	Risk Ratio (IV, Random, 95% CI)	1.16 [0.81, 1.65]
2.5.1 Stroke	2	600	Risk Ratio (IV, Random, 95% CI)	1.40 [0.91, 2.15]
2.5.2 Older adults	2	313	Risk Ratio (IV, Random, 95% CI)	0.87 [0.67, 1.13]
2.6 Skill training—Participation ≤6 months	7	886	Std. Mean Difference (IV, Random, 95% CI)	0.01 [−0.23, 0.25]
2.6.1 Stroke	6	846	Std. Mean Difference (IV, Random, 95% CI)	0.06 [−0.20, 0.31]
2.6.2 Wheelchair users	1	40	Std. Mean Difference (IV, Random, 95% CI)	−0.43 [−1.06, 0.20]
2.7 Physical training—Participation ≤6 months	1	228	Std. Mean Difference (IV, Random, 95% CI)	0.12 [−0.14, 0.38]
2.7.1 Older adults	1	228	Std. Mean Difference (IV, Random, 95% CI)	0.12 [−0.14, 0.38]
2.8 Participation ≥7 months	4	890	Std. Mean Difference (IV, Random, 95% CI)	−0.03 [−0.21, 0.16]
2.8.1 Stroke	3	674	Std. Mean Difference (IV, Random, 95% CI)	−0.05 [−0.31, 0.20]
2.8.2 Older adults	1	216	Std. Mean Difference (IV, Random, 95% CI)	0.06 [−0.21, 0.32]
2.9 Skill training—Health‐related quality of life ≤6 months	4	779	Std. Mean Difference (IV, Random, 95% CI)	0.13 [−0.20, 0.46]
2.9.1 Stroke	2	552	Std. Mean Difference (IV, Random, 95% CI)	−0.13 [−0.47, 0.21]
2.9.2 Older adults	1	109	Std. Mean Difference (IV, Random, 95% CI)	0.38 [0.00, 0.76]
2.9.3 Cognitive impairments	1	118	Std. Mean Difference (IV, Random, 95% CI)	0.49 [0.12, 0.86]
2.10 Physical training—Health‐related quality of life ≤6 months	1	225	Std. Mean Difference (IV, Random, 95% CI)	0.02 [−0.25, 0.28]
2.10.1 Older adults	1	225	Std. Mean Difference (IV, Random, 95% CI)	0.02 [−0.25, 0.28]
2.11 Health‐related quality of life ≥7 months	3	710	Std. Mean Difference (IV, Random, 95% CI)	−0.04 [−0.18, 0.11]
2.11.1 Stroke	2	495	Std. Mean Difference (IV, Random, 95% CI)	−0.05 [−0.23, 0.13]
2.11.2 Older adults	1	215	Std. Mean Difference (IV, Random, 95% CI)	−0.01 [−0.28, 0.26]
2.12 Major adverse events (Dichotomous measures) ≤6 months	4	418	Risk Ratio (IV, Random, 95% CI)	0.66 [0.35, 1.22]
2.12.1 Stroke	2	99	Risk Ratio (IV, Random, 95% CI)	0.83 [0.27, 2.54]
2.12.2 Older adults	1	109	Risk Ratio (IV, Random, 95% CI)	0.36 [0.14, 0.95]
2.12.3 Musculoskeletal injuries	1	210	Risk Ratio (IV, Random, 95% CI)	1.20 [0.38, 3.81]
2.13 Major adverse events (Dichotomous measures) ≥7 months	3	906	Risk Ratio (IV, Random, 95% CI)	1.07 [0.88, 1.31]
2.13.1 Stroke	1	568	Risk Ratio (IV, Random, 95% CI)	0.98 [0.45, 2.14]
2.13.2 Older adults	2	338	Risk Ratio (IV, Random, 95% CI)	0.96 [0.57, 1.60]
2.14 Minor adverse events (Dichotomous measures) ≤6 months	4	443	Risk Ratio (IV, Random, 95% CI)	0.89 [0.65, 1.21]
2.14.1 Stroke	2	124	Risk Ratio (IV, Random, 95% CI)	1.14 [0.63, 2.04]
2.14.2 Older adults	1	109	Risk Ratio (IV, Random, 95% CI)	0.74 [0.49, 1.12]
2.14.3 Musculoskeletal injuries	1	210	Risk Ratio (IV, Random, 95% CI)	1.10 [0.49, 2.48]
2.15 Minor adverse events (Dichotomous measures) ≥7 months	4	962	Risk Ratio (IV, Random, 95% CI)	1.02 [0.89, 1.15]
2.15.1 Stroke	2	627	Risk Ratio (IV, Random, 95% CI)	1.00 [0.84, 1.18]
2.15.2 Older adults	2	335	Risk Ratio (IV, Random, 95% CI)	1.04 [0.86, 1.26]

**Comparison 3** Different intensity of outdoor mobility interventions

**Table:** 

Outcome or subgroup title	No. of studies	No. of participants	Statistical method	Effect size
3.1 Participation ≤6 months	1	68	Std. Mean Difference (IV, Random, 95% CI)	0.00 [−0.48, 0.48]
3.2 Participation ≥7 months	1	68	Std. Mean Difference (IV, Random, 95% CI)	0.09 [−0.39, 0.57]
3.3 Health‐related quality of life ≤6 months	1	68	Std. Mean Difference (IV, Random, 95% CI)	−0.12 [−0.59, 0.36]
3.4 Health‐related quality of life ≥7 months	1	68	Std. Mean Difference (IV, Random, 95% CI)	0.20 [−0.27, 0.68]
3.5 Minor adverse events ≤6 months	1	63	Risk Ratio (M‐H, Random, 95% CI)	5.45 [0.70, 42.73]
3.6 Minor adverse events ≥7 months	1	59	Risk Ratio (M‐H, Random, 95% CI)	0.60 [0.32, 1.13]

## WHAT'S NEW

**Table:** 

Date	Event	Description
23 October 2023	Amended	

## CONTRIBUTIONS OF AUTHORS

All the authors contributed to the conduct of the review. MR conducted the statistical analysis, and MR and BI conducted data extraction, risk of bias and GRADE assessments with EML and SI support with consensus. MR and EML wrote the first draft of the review, with additional revision and input from SI and BI.

## DECLARATIONS OF INTEREST

EML and SI have previously developed an intervention, which has been evaluated in a non‐randomized feasibility study, within the topic of this review. Due to the inclusion criteria of this review, this study will not be included in the upcoming review. MR, EML and SI will use the conclusions from this review in the development of a novel intervention to improve outdoor mobility. An update of this review might include any future studies which will have to adhere to the same methodology as presented in this protocol.

## PLANS FOR UPDATING THIS REVIEW

Updates of this review are planned to be carried out at least every 4 years. The main responsibility to initiate an update will be the contact author of the review.

## SOURCES OF SUPPORT

### Internal sources

- Cochrane Sweden, Sweden
  Staff within Cochrane Sweden were available for methodological support.
- Lund University Library, Sweden

The library at Lund University were available to provide support for conducting, running and reporting the search strategy used in the review.

### External sources

- FORMAS Grant, Sweden

Formas is a government research council in Sweden focusing on sustainable development. FORMAS funds research and innovation, develop strategies, perform analyses and conduct evaluations. The grant will cover the salary for conducting the review.

## DIFFERENCES BETWEEN PROTOCOL AND REVIEW

**Indoor mobility components:** We have clarified the methods for inclusion of studies with an aim to improve outdoor mobility which also included indoor mobility, when the aim was clearly described to have an outdoor mobility focus for either the majority of participants or the majority of intervention components. This is to supplement our guidance in our protocol regarding when to exclude these interventions: ‘Interventions without an aim or purpose to improve outdoor mobility will be excluded, for example interventions aiming to only improve physical activity levels or walking speed for the participants. In addition, interventions with a focus on only improving mobility indoors or personal ADL will be excluded’.

**Pharmacological interventions:** We clarified our exclusion criteria, stating to exclude any pharmacological interventions, to exclude any nutrition interventions that could aim to improve outdoor mobility. These interventions were not included in the prespecified Logic Model in the protocol, but we did not specifically write about this exclusion in text form.

**Additions to subgroup analyses**: At the full‐text screening stage, before data extraction, critical appraisal and any analysis, we noticed that many studies used wheelchair training to improve outdoor mobility, something we did not specify in our subgroup analysis. We added this to the category together with mobility scooters. We also merged the ‘bus and tram’‐category with ‘train’ category, as these are similar modes of transport ‐ to reduce the number of subgroups to get a better overview.

**Environmental components as main intervention**: We clarified our protocol that when environmental components are clearly the main component and other components are just given as a sub‐training, we will exclude these interventions (e.g., studies of training consist of 15 min instructions and the rest of the intervention is delivering large environmental changes or delivering walking aids).

**Analysis groups**: We expected a large variety of different intervention groups, but found only skill training and physical training interventions. For accuracy and being potential major effect modifiers, we have structured the meta‐analyses and results based on these two distinct interventions in subgroups, and not, as specified in the protocol, to just explore all intervention components and report all outdoor mobility interventions in the same meta‐analysis without subgroups. We believe this difference between protocol and review improves the accuracy of the reviews review's conclusions about specific forms of interventions to improve outdoor mobility.

**Presentation of groups in ‘Summary of findings’‐tables**: We revised the presentation of results in the summary of findings‐tables to be in line with the result presentation. This leads us to not summarize all outdoor mobility interventions together, and instead split them into skill training and physical training interventions for each time‐period, and not one SoF for each condition due to the total number of tables becoming too many (above 10). This expanded the number of tables from the pre‐specified two to four in total.

### PEER REVIEW

The peer review history for this article is available at https://www.webofscience.com/api/gateway/wos/peer-review/10.1002/cl2.1407.

## Supporting information

Supporting information.

Supporting information.

## Data Availability

n/a
