# Supplementary material for: Interventions to improve outdoor mobility among people living with disabilities: A systematic review
Source: Campbell Syst Rev. 2024 Jun 14;20(2):e1407. doi: 10.1002/cl2.1407 (PMC11177337; doi:10.1002/cl2.1407)
Supplement: Supplementary file 1 — Supporting information. [file CL2-20-e1407-s002.docx]

| [Öztürk 2011](#STD-_x00d6_zt_x00fc_rk-2011) |  |
| --- | --- |

# Appendices

## Appendix 1: Search strategies and search report

**Appendix 1. Appendix 1: Search strategies and search report**

| **Search strategies, June 2022** |
| --- |
| **MEDLINE (EbscoHost, 1946 to present)** |
| Date of search: 2022-06-01No publication date limitations or language limitations were used. |
| Search filters: A modified version of the Cochrane Highly Sensitive Search Strategy for randomized controlled trials was used. |
| #1 ( (community OR outdoor* OR (outside N3 home) OR "out of home") N3 (ambulat* OR transportation OR walk* OR mobility OR travel*)) OR ( "life space mobility" OR "life space assessment" OR "walking capacity" OR "walking ability" OR "outdoor activit*" OR "outsideactivit*" OR (participation N3 outdoor*) ) OR (MM "Mobility Limitation") 13,375 |
| #2 PT ( randomized controlled trial OR controlled clinical trial ) OR AB ( randomized OR placebo OR randomised OR randomly OR rct ORtrial OR groups ) OR TI ( randomized OR placebo OR randomised OR randomly OR rct OR trial OR groups ) NOT ( (MH "Animals+")NOT MH humans ) 5,175,114 |
| #3 #1 AND #2 5,035 |

| **AMED (EbscoHost, inception to present)** |
| --- |
| Date of search: 2022-06-20No publication date limitations or language limitations were used. |
| #1 ( (community OR outdoor* OR (outside N3 home) OR "out of home") N3 (ambulat* OR transportation OR walk* OR mobility OR travel*)) OR ( "life space mobility" OR "life space assessment" OR "walking capacity" OR "walking ability" OR "outdoor activit*" OR "outsideactivit*" OR (participation N3 outdoor*) ) OR “mobility limitation” 1,433 |
| #2 TI ( randomized OR placebo OR randomised OR randomly OR rct OR trial ) OR AB ( randomized OR placebo OR randomised ORrandomly OR rct OR trial ) OR (ZU "randomized controlled trials") 30,198 |
| #3 #1 AND #2 288 |

| **APA PsycInfo (EbscoHost, inception to present)** |
| --- |
| Date of search: 2022-06-20No publication date limitations or language limitations were used. |
| #1 ( DE "Randomized Controlled Trials" OR DE "Randomized Clinical Trials" ) OR AB ( randomized OR placebo OR randomised ORrandomly OR rct OR trial ) OR TI ( randomized OR placebo OR randomised OR randomly OR rct OR trial ) 308,514 |
| #2 ( (community OR outdoor* OR (outside N3 home) OR "out of home") N3 (ambulat* OR transportation OR walk* OR mobility OR travel*)) OR ( "life space mobility" OR "life space assessment" OR "walking capacity" OR "walking ability" OR "outdoor activit*" OR "outsideactivit*" OR (participation N3 outdoor*) ) OR “mobility limitation” 20,362 |
| #3 #1 AND #2 1,972 |

| **CENTRAL via Wiley Cochrane Library** |
| --- |
| Date of search: 2022-06-20No publication date limitations or language limitations were used. |
| #1 ((community OR outdoor* OR (outside NEAR/3 home) OR "out of home") NEAR/3 (ambulat* OR transportation OR walk* OR mobilityOR travel*)):ti,ab,kw (Word variations have been searched) 1,146 |
| #2 ("life space mobility" OR "life space assessment" OR "walking capacity" OR "walking ability" OR "outdoor activit*" OR "outside activit*"OR (participation NEAR/3 outdoor*)):ti,ab,kw (Word variations have been searched) 1,981 |
| #3 MeSH descriptor: [Mobility Limitation] explode all trees 537 |
| #4 #1 OR #2 OR #3 3,442 |

| **CINAHL Complete** (Cumulative Index to Nursing and Allied Health Literature; |
| --- |
| EbscoHost, inception to present) |
| Date of search: 2022-06-20No publication date limitations or language limitations were used. |
| Search filters: The search filter for randomized controlled trials was adapted from Glanville J. Health Info Libr J. (2019). |
| #1 ( (community OR outdoor* OR (outside N3 home) OR "out of home") N3 (ambulat* OR transportation OR walk* OR mobility OR travel*)) OR ( "life space mobility" OR "life space assessment" OR "walking capacity" OR "walking ability" OR "outdoor activit*" OR "outsideactivit*" OR (participation N3 outdoor*) ) OR “mobility limitation” 24,974 |
| #2 (( MH randomized controlled trials OR MH double blind study OR MH single blind studies OR MH random assignment ) OR TI (randomized OR placebo OR randomised OR randomly OR rct OR trial ) OR AB ( randomized OR placebo OR randomised OR randomlyOR rct OR trial )) NOT (( MH animals+ OR MH animal studies OR TI animal model*) NOT MH human) 558,337 |
| #3 #1 AND #2 3,520 |

| **ERIC, (EbscoHost, inception to present)** |
| --- |
| Date of search: 2022-06-20No publication date limitations or language limitations were used. |

| #1 ( (community OR outdoor* OR (outside N3 home) OR "out of home") N3 (ambulat* OR transportation OR walk* OR mobility OR travel*)) OR ( "life space mobility" OR "life space assessment" OR "walking capacity" OR "walking ability" OR "outdoor activit*" OR "outsideactivit*" OR (participation N3 outdoor*) ) OR "mobility limitation" 11,438 |
| --- |
| #2 TI ( randomized OR placebo OR randomised OR randomly OR rct OR trial ) OR AB ( randomized OR placebo OR randomised ORrandomly OR rct OR trial ) OR DE "Randomized Controlled Trials" 35,198 |
| #3 #1 AND #2 114 |

| **Scopus (Elsevier, inception to present)** |
| --- |
| Date of search: 2022-06-22 |
| No publication date limitations or language limitations were used. |
| #1 (TITLE-ABS-KEY ( ( community OR outdoor* OR ( outside W/3 home ) OR "out of home" ) W/3 ( ambulat* OR transportation OR walk*OR mobility OR travel* ) ) OR TITLE-ABS-KEY ( ( "life space mobility" OR "life space assessment" OR "walking capacity" OR "walkingability" OR "outdoor activit*" OR "outside activit*" OR ( participation W/3 outdoor* ) ) ) OR TITLE-ABS-KEY ( "mobility limitation" ) ) 24,342 |
| #2 (TITLE (randomized OR placebo OR randomised OR randomly OR rct OR trial) OR ABS (randomized OR placebo OR randomisedOR randomly OR rct OR trial) OR KEY ("randomized controlled trial" OR rct OR "randomized controlled study")) 2,702,882 |
| #3 #1 AND #2 3,356 |

| **Web of Science Core Collection (Clarivate Analytics)** |
| --- |
| Date of search: 2022-06-21 |
| No publication date limitations or language limitations were used. |
| Databases searched in Web of Science Core Collection: |
| Conference Proceedings Citation Index- Science (CPCI-S) --1990-present |
| Conference Proceedings Citation Index- Social Science & Humanities (CPCI-SSH) --1990-present |
| #1 TS=((community OR outdoor* OR (outside NEAR/3 home) OR "out of home") NEAR/3 (ambulat* OR transportation OR walk* ORmobility OR travel*)) 974 |
| #2 TS=("life space mobility" OR "life space assessment" OR "walking capacity" OR "walking ability" OR "outdoor activit*" OR "outsideactivit*" OR (participation NEAR/3 outdoor*) OR "mobility limitation") 820 |
| #3 #1 OR #2 1,769 |
| #4 (TI=(randomized OR placebo OR randomised OR randomly OR rct OR trial)) OR AB=(randomized OR placebo OR randomised ORrandomly OR rct OR trial) 230,933 |
| #5 #3 AND #4 103 |

| **Embase (via Embase.com)** |
| --- |
| Date of search: 2022-06-22No publication date limitations or language limitations were used. |
| Search filters: The Cochrane search filter for randomized controlled trials in OVID Embase as published by Cochrane Neonatal. |
| #01. ((community OR outdoor* OR 'outside home' OR 'out of home') NEAR/3 (ambulat* OR transportation OR walk* OR mobility ORtravel*)):ti,ab,kw 4,904 |
| #02. 'life space mobility':ti,ab,kw OR 'life space assessment':ti,ab,kw OR 'walking capacity':ti,ab,kw OR 'walking ability':ti,ab,kw OR'outdoor activit*':ti,ab,kw OR 'outside activit*':ti,ab,kw OR ((participation NEAR/3 outdoor*):ti,ab,kw) OR 'walking difficulty'/mj 10,905 |
| #03. #1 OR #2 15,304 |
| #04. 'randomized controlled trial'/de OR 'controlled clinical trial'/de 892,059 |
| #05. random*:ti,ab,kw 1,806,073 |
| #06. 'randomization'/de 94,010 |
| #07. placebo:ti,ab,kw 343,587 |
| #08. ((double OR single OR doubly OR singly) NEAR/2 (blind OR blinded OR blindly)):ti,ab,kw 261,077 |
| #09. 'double blind procedure'/de 196,469 |
| #10. (controlled NEAR/7 (study OR design OR trial)):ti,ab,kw 419,711 |
| #11. 'parallel group$':ti,ab 29,476 |
| #12. crossover:ti,ab OR 'cross over':ti,ab 117,117 |
| #13. ((assign* OR match OR matched OR allocation) NEAR/5 (alternate OR group$ OR intervention$ OR patient$ OR subject$ ORparticipant$)):ti,ab 380,886 |
| #14. (open NEAR/2 label):ti,ab 97,246 |
| #15. quasirandom*:ti,ab,kw OR 'quasi random*':ti,ab,kw OR randomi*:ti,ab,kw OR randomly:ti,ab,kw 1,472,148 |
| #16. (control* NEAR/2 (group$ OR random*)):ti,ab,kw 1,198,444 |
| #17.#4 OR #5 OR #6 OR #7 OR #8 OR #9 OR #10 OR #11 OR #12 OR #13 OR #14 OR #15 OR #16 3,089,348 |
| #18. ('animal'/exp OR 'invertebrate'/exp OR 'animal experiment'/de OR 'animal model'/de OR 'animal tissue'/de OR 'animal cell'/de OR'nonhuman'/de) AND ('human'/de OR 'normal human'/de OR 'human cell'/de) 24,901,854 |
| #19. 'animal'/exp OR 'invertebrate'/exp OR 'animal experiment'/de OR 'animal model'/de OR 'animal tissue'/de OR 'animal cell'/de OR'nonhuman'/de 32,422,075 |
| #20. #19 NOT #18 7,520,221 |
| #21. #17 NOT #20 2,650,799 |
| #22. #3 AND #21 3,282 |
| Total number of hits from all searches, June 2022 21,112 |

| **Update searches, January 2023** |
| --- |

| The same search strategies were applied for the update searches as for the original searches. Searches were then restricted to 2022-2023. The searches were run between 2023-01-05 and 2023-01-11. |
| --- |
| Results: |
| MEDLINE (EbscoHost) 329 |
| AMED (EbscoHost) 2 |
| APA PsycInfo (EbscoHost) 73 |
| CENTRAL 1 |
| CINAHL Complete (EbscoHost) 170 |
| ERIC (EbscoHost) 2 |
| Scopus (Elsevier) 247 |
| Web of Science Core Collection (Clarivate Analytics) 0 |
| Embase (via Embase.com) 211 |
| Total hits from update searches 1,035 |
| A PEDro search was run on 2023-01-04 |
| Abstract & Title: |
| ”outdoor mobility” 7 hits |
| ”mobility outdoors” 1 hit |
| ”outdoor walking” 19 hits |
| “walking outdoors” 8 hits |
| ”outdoor ambulation” 2 hits |
| “ambulation outdoor” 0 hits |
| “ambulation outdoors” 0 hits |
| ”outdoor traveling” 0 hits |
| “traveling outdoors” 0 hits |
| ”outdoor transportation” 0 hits |
| ”community mobility” 6 hits |
| “mobility in the community” 1 hit |
| ”community walking” 10 hits |
| “walking in the community” 0 hits |
| ”community ambulation” 23 hits |
| “ambulation in the community” 0 hits |
| ”community traveling” 0 hits |
| travel* “in the community” 4 hits |
| ”participation in the community” 6 hits |
| ”community participation” 22 hits |
| ”community transportation” 0 hits |
| life space mobility 13 hits |
| ”life space” 16 hits |
| Outdoor* participation 10 hits |
| Further searches |
| Title only: walking AND Abstract/Title: “in the community” 15 hits |
| Title only: outdoor* 23 hits |
| Unique hits from PEDro 147 |
| Total hits from all database searches 22,333 |

| **Search strategies, June 2022** |
| --- |
|  |

## Appendix 2. Data extraction form

**1. General information about the included trial**

- 1.1. Study ID in Covidence.*Example: “#12345”.*
- 1.2. First author of the main study and year of publication. *Example: “Logan 2014”.*
- 1.3. Name of study. *Example: “Outdoor mobility intervention for disability: a randomized controlled trial”.*
- *1.4. Funding source of the trial. Example: "The NHS research and development department"*
- *1.5. Any declaration or conflicts of interest (CoI). Example: "No CoI stated", "CoI not reported"*
- *1.6. Any rational or goal given (related to outdoor mobility). Quote one (or if needed several paragraphs from the paper): Example:*

**2. Population in the included trial.**

- 2.1. Specific population or condition. *Example: “Stroke”, "Multiple Sclerosis", “Dementia”, “Osteoarthritis”, “Older adults”, "Mixed population (wheelchair users)", etc.*
- 2.2. Age of participants. *Description of mean age and Standard Deviation (SD) for included participants. If mean is not available, use Median and IQR. Example: "Mean age 71 years (SD 5)".*
- 2.3. Gender of participants. *Example: “55% Women”*
- 2.4. Country. *Example: “Sweden”, “United Kingdom”, “Taiwan” etc.*
- 2.5. Recruitment setting. *Example: “Hospital”, “Community” etc.*

**3. Interventions in the included trial.**

- 3.01. Name of intervention. *Example: “GetOut”, “BusTrips”, “Walking-In-Community" or “resistance training”.*
- 3.02. Year when the intervention was initiated and conducted (e.g. not the date published). *Example: “2012” or "Not reported".*
- 3.03. Intervention setting. *Example: “Community”, “hospital”, “primary care”, "community".*
- 3.04. Intervention delivery. *Example: “Individual”, “Group”, "Digital", or "Mix (group and digital)"*
- 3.05. Intervention components. *Example (according to Logic Model): “Physical training”, “Cognitive training”, “Skill training”, “Education”, “Phycological training”, or "Mix (physical training and education".*
- 3.06. Type of intervention components related to travel. *Example: “Walking”, “bus”, “tram”, "wheelchair", “car”, “mobility aids”, “bicycle”, “electronic travel modes”, or "Mix (walking and bus)".*
- 3.07. Tailoring of the intervention. *Was the intervention delivered as a strict program or was it possible to tailor the intervention to the participants? Example (can be a copy paste from the publication if possible): “The intervention was personalized to the participants in accordance to their set goals of activity”.*
- 3.08. Duration of intervention in number of sessions defined in the intervention protocol. *Described as mean with SD or median with IQR. Example: “mean 10 sessions (4 SD)”, "no information".*
- 3.09. Intensity of intervention in frequency and length of intervention sessions.  *Time of average mean session in minutes and number of intervention sessions per week. Example: “60-minute sessions”*
- 3.10. Duration of intervention in the length of the active intervention period. *Described as mean with SD or median with IQR in weeks from start of intervention to end of intervention.  Example: “10 weeks”*
- 3.11. Credentials of the treatment provider. *Example: “Physiotherapist”, “occupational therapist”, “nurses”, “social workers”, “psychologists”, “medical practitioner”, “multiple treatment providers (physiotherapist and occupational therapist)”, "not reported”.*
- 3.12. Materials used (any physical or informational materials used in the intervention, including those provided to participants or used in intervention delivery or in training of intervention providers). Quote, or link to additional material. Example: "Material and intervention delivery can be assessed from LINK", or "No additional intervention information is available in or linked in the publication".

**Comparisons in the included trial**

- 3.12. Comparison intervention. *Example: “Wait-list control”, “treatment as usual”, “attention control” or “no treatment”. Describe additional details about the comparison, for example extent of the control intervention if available in the article.*
- 3.13. If "active" comparison intervention components describe the component. If no active comparison, describe the comparison in as much detail in free text or with quote from study. Leave empty if  *Example (according to Logic Model): “Physical training”, “Cognitive training”, “Skill training”, “Education”, “Phycological training”, or "Mix (physical training and education".*
- 3.14. Type of comparison intervention components related to travel. *Example: “Walking”, “bus”, “tram”, "wheelchair", “car”, “mobility aids”, “bicycle”, “electronic travel modes”, or "Mix (walking and bus)".*
- 3.15. Tailoring of the intervention. *Was the intervention delivered as a strict program or was it possible to tailor the intervention to the participants? Example (can be a copy paste from the publication if possible): “The intervention was personalized to the participants in accordance to their set goals of activity”.*
- 3.16. Duration of intervention in number of sessions defined in the intervention protocol. *Described as mean with SD or median with IQR. Example: “mean 10 sessions (4 SD)”, "no information".*
- 3.17. Intensity of intervention in frequency and length of intervention sessions. *Time of average mean session in minutes and number of intervention sessions per week. Example: “60-minute sessions”*
- 3.18. Duration of intervention in the length of the active intervention period. *Described as mean with SD or median with IQR in weeks from start of intervention to end of intervention. Example: “10 weeks”*
- 3.19. Credentials of the treatment provider. *Example: “Physiotherapist”, “occupational therapist”, “nurses”, “social workers”, “psychologists”, “medical practitioner”, “multiple treatment providers (physiotherapist and occupational therapist)”, "not reported”.*

**4. Outcomes of interest included in the trial**

- 4.1. Reported outcomes we have predefined. *Example: "Outdoor mobility", “Activity outside the home”, “Health-related quality of life”, “Participation”, "Major adverse events”, or "Minor adverse events".*
- 4.2. Specific measure of the outcome. *Example: “SF36”, “journeys outside the home”, “number of falls”, "Stroke impact scale", "Life-space Assessment" or "steps outside".*
- 4.3. Additional details how the outcome was measured. Example "measured using a pedometer", "measured by a diary" or "measured a digital questionnaire".
- 4.4. Outcome measure data report. *Example: “Self-reported”, “carer-reported”, “assessor reported”.*
- 4.5. Time-points of collection of outcome measures. *Specific time-points of reported outcome measures based on time from the beginning of the intervention. Example: Outcomes reported at 1, 3 and 12 months.*

**5. Methods used in the included trial**

- 5.1. Number of participants in each group at randomization. *Example: “100 participants in the intervention group and 99 participants in the control group at randomization”.*
- 5.2. Number of participants at *the*analysis of outcomes. *Example: “At 9 month follow up 80 participants reported outcomes in the intervention group and 50 in the control group”.*
- 5.3. Analysis based on Intention-to-treat or per protocol. *Example. “Analysis carried out as intention-to-treat.”*

## Appendix 3. Risk of bias judgements and details

| Unique ID | Ada 2012 Health-related quality of life 6 months | Study ID | Ada 2012 | Assessor | MR |
| --- | --- | --- | --- | --- | --- |
| Ref or Label |  | Aim | assignment to intervention (the 'intention-to-treat' effect) |  |  |
| Experimental |  | Comparator |  | Source | Journal article(s) |
| Outcome | Health-related quality of life 6 months | Results |  | Weight | 1 |
| Domain | Signalling question | | | Response | Comments |
| Bias arising from the randomization process | 1.1 Was the allocation sequence random? | | | PY | States to be using a computer-generated randomization sequence, enrolling particiants in cohorts of 15 and by further dividing cohorts by matching based on walking speed of 3 in each subcohort. This indicates very small randomisation units. States to be "consealed" allocation but does not give any more information. |
|  | 1.2 Was the allocation sequence concealed until participants were enrolled and assigned to interventions? | | | PY |  |
|  | 1.3 Did baseline differences between intervention groups suggest a problem with the randomization process? | | | Y | Baseline inbalances for QoL (mean 61 vs 73) shows a 1 in around 100 chance of happening by chance, and participation (mean 20 vs 25) for 1 in around 20. Walking speed (232 vs 244) compatible to chance. |
|  | Risk of bias judgement | | | Some concerns | Rating based on on suboptimal randomisation method, unclear specification of allocation consealment and large baseline differences incompatible to chance for QoL and participation outcomes, which are the outcomes we include in our systematic review. |
| Bias due to deviations from intended interventions | 2.1.Were participants aware of their assigned intervention during the trial? | | | Y | Both participants and intervention personnell were aware of the assigned interventions. |
|  | 2.2.Were carers and people delivering the interventions aware of participants' assigned intervention during the trial? | | | Y |  |
|  | 2.3. If Y/PY/NI to 2.1 or 2.2: Were there deviations from the intended intervention that arose because of the experimental context? | | | PY | No clear information on deviations reported. Some indicators of deviations are present in form of behavior differences, i.e. the control group had a drop-off of 10% while intervention group had 0%. |
|  | 2.4 If Y/PY to 2.3: Were these deviations likely to have affected the outcome? | | | PY | Since the control group did not get any intervention, it would be likely that they behaved differently compared to the intervention group due to the trial conext. |
|  | 2.5. If Y/PY/NI to 2.4: Were these deviations from intended intervention balanced between groups? | | | PN | Any deviations would probably not be balanced between the groups. |
|  | 2.6 Was an appropriate analysis used to estimate the effect of assignment to intervention? | | | PY | ITT analysis was used, imputing values for missing data. |
|  | 2.7 If N/PN/NI to 2.6: Was there potential for a substantial impact (on the result) of the failure to analyse participants in the group to which they were randomized? | | | NA |  |
|  | Risk of bias judgement | | | High | Both participants and intervention personnell were aware of the assigned interventions. No clear information on deviations reported. Some indicators of deviations are present in form of behavior differences, i.e. the control group had a drop-off of 10% while intervention group had 0%.  Since the control group did not get any intervention, it would be likely that they behaved differently compared to the intervention group due to the trial conext. Any deviations would probably not be balanced between the groups. ITT analysis was used, imputing values for missing data. |
| Bias due to missing outcome data | 3.1 Were data for this outcome available for all, or nearly all, participants randomized? | | | PY | 94% available data at 6 months in all three groups, while at least 3 out of 34 missing data in the no intervention group. Probably very small or no impact of the overall minor missing data on continuous outcome data. |
|  | 3.2 If N/PN/NI to 3.1: Is there evidence that result was not biased by missing outcome data? | | | NA |  |
|  | 3.3 If N/PN to 3.2: Could missingness in the outcome depend on its true value? | | | NA |  |
|  | 3.4 If Y/PY/NI to 3.3: Is it likely that missingness in the outcome depended on its true value? | | | NA |  |
|  | Risk of bias judgement | | | Low | Rating based on 94% available data at 6 months in all three groups, while at least 3 out of 34 missing data in the no intervention group. Probably very small or no impact of the overall minor missing data on continuous outcome data. Missing data imputed from LOCF. |
| Bias in measurement of the outcome | 4.1 Was the method of measuring the outcome inappropriate? | | | N | Used a validated scale |
|  | 4.2 Could measurement or ascertainment of the outcome have differed between intervention groups? | | | NI | No information provided |
|  | 4.3 Were outcome assessors aware of the intervention received by study participants? | | | Y | Participants rating their outcomes were aware of the intervention recieved |
|  | 4.4 If Y/PY/NI to 4.3: Could assessment of the outcome have been influenced by knowledge of intervention received? | | | PY | Both the intervention group and no intervention group could have rated their participation levels differently due to knowledge of group participation. Intervention groups likely more favorably due to getting to know and trust their therapists, and no intervention group by being aware that they did not get any intervention to improve participation as the other group did. |
|  | 4.5 If Y/PY/NI to 4.4: Is it likely that assessment of the outcome was influenced by knowledge of intervention received? | | | PY |  |
|  | Risk of bias judgement | | | High | Rating based on both the intervention group and no intervention group could have rated their participation levels differently due to knowledge of group participation, and that no information is available for when and how outcome measurements were measured in the groups Intervention groups possibly more favorably due to getting to know and trust their therapists, and control intervention group by being aware that they did not get any intervention to improve participation as the other group did. |
| Bias in selection of the reported result | 5.1 Were the data that produced this result analysed in accordance with a pre-specified analysis plan that was finalized before unblinded outcome data were available for analysis? | | | Y | Protocol published prospectively in 2008. |
|  | 5.2 ... multiple eligible outcome measurements (e.g. scales, definitions, time points) within the outcome domain? | | | N | All outcome measures mentioned in the protocol |
|  | 5.3 ... multiple eligible analyses of the data? | | | PN | Analysis methods reported in the protocol, but number of analyses and which are given priority across all time-points is not completely specified. |
|  | Risk of bias judgement | | | Low | Rating based on a protocol published prospectively in 2008 specifying all reported outcomes and adequately specifying the analyses conducted. |
| Overall bias | Risk of bias judgement | | | High | Overall high risk of bias due to high risk of bias in the measurement of the outcome due to participants rating their own participation levels, and some concers in the randomisation process due to unclear and large baseline differences, no description of methods for allocation consealment and some concerns for deviations from intended interventions due to having an inactive no intervention-comparison group. |
|  |  |  |  |  |  |
|  |  |  |  |  |  |
| Unique ID | Ada 2012 Health-related quality of life 7 months | Study ID | Ada 2012 | Assessor | MR |
| Ref or Label |  | Aim | assignment to intervention (the 'intention-to-treat' effect) |  |  |
| Experimental |  | Comparator |  | Source | Journal article(s) |
| Outcome | Health-related quality of life 7 months | Results |  | Weight | 1 |
| Domain | Signalling question | | | Response | Comments |
| Bias arising from the randomization process | 1.1 Was the allocation sequence random? | | | PY | States to be using a computer-generated randomization sequence, enrolling particiants in cohorts of 15 and by further dividing cohorts by matching based on walking speed of 3 in each subcohort. This indicates very small randomisation units. States to be "consealed" allocation but does not give any more information. |
|  | 1.2 Was the allocation sequence concealed until participants were enrolled and assigned to interventions? | | | PY |  |
|  | 1.3 Did baseline differences between intervention groups suggest a problem with the randomization process? | | | Y | Baseline inbalances for QoL (mean 61 vs 73) shows a 1 in around 100 chance of happening by chance, and participation (mean 20 vs 25) for 1 in around 20. Walking speed (232 vs 244) compatible to chance. |
|  | Risk of bias judgement | | | Some concerns | Rating based on on suboptimal randomisation method, unclear specification of allocation consealment and large baseline differences incompatible to chance for QoL and participation outcomes, which are the outcomes we include in our systematic review. |
| Bias due to deviations from intended interventions | 2.1.Were participants aware of their assigned intervention during the trial? | | | Y | Both participants and intervention personnell were aware of the assigned interventions. |
|  | 2.2.Were carers and people delivering the interventions aware of participants' assigned intervention during the trial? | | | Y |  |
|  | 2.3. If Y/PY/NI to 2.1 or 2.2: Were there deviations from the intended intervention that arose because of the experimental context? | | | PY | No clear information on deviations reported. Some indicators of deviations are present in form of behavior differences, i.e. the control group had a drop-off of 10% while intervention group had 0%. |
|  | 2.4 If Y/PY to 2.3: Were these deviations likely to have affected the outcome? | | | PY | Since the control group did not get any intervention, it would be likely that they behaved differently compared to the intervention group due to the trial conext. |
|  | 2.5. If Y/PY/NI to 2.4: Were these deviations from intended intervention balanced between groups? | | | PN | Any deviations would probably not be balanced between the groups. |
|  | 2.6 Was an appropriate analysis used to estimate the effect of assignment to intervention? | | | PY | ITT analysis was used, imputing values for missing data. |
|  | 2.7 If N/PN/NI to 2.6: Was there potential for a substantial impact (on the result) of the failure to analyse participants in the group to which they were randomized? | | | NA |  |
|  | Risk of bias judgement | | | High | Both participants and intervention personnell were aware of the assigned interventions. No clear information on deviations reported. Some indicators of deviations are present in form of behavior differences, i.e. the control group had a drop-off of 10% while intervention group had 0%.  Since the control group did not get any intervention, it would be likely that they behaved differently compared to the intervention group due to the trial conext.  Any deviations would probably not be balanced between the groups.  ITT analysis was used, imputing values for missing data. |
| Bias due to missing outcome data | 3.1 Were data for this outcome available for all, or nearly all, participants randomized? | | | PY | 94% available data at 6 months in all three groups, while at least 3 out of 34 missing data in the no intervention group. Probably very small or no impact of the overall minor missing data on continuous outcome data. |
|  | 3.2 If N/PN/NI to 3.1: Is there evidence that result was not biased by missing outcome data? | | | NA |  |
|  | 3.3 If N/PN to 3.2: Could missingness in the outcome depend on its true value? | | | NA |  |
|  | 3.4 If Y/PY/NI to 3.3: Is it likely that missingness in the outcome depended on its true value? | | | NA |  |
|  | Risk of bias judgement | | | Low | Rating based on 94% available data at 6 months in all three groups, while at least 3 out of 34 missing data in the no intervention group. Probably very small or no impact of the overall minor missing data on continuous outcome data. Missing data imputed from LOCF. |
| Bias in measurement of the outcome | 4.1 Was the method of measuring the outcome inappropriate? | | | N | Using a validated participation scale |
|  | 4.2 Could measurement or ascertainment of the outcome have differed between intervention groups? | | | NI | No information provided |
|  | 4.3 Were outcome assessors aware of the intervention received by study participants? | | | Y | Participants rating their outcomes were aware of the intervention recieved |
|  | 4.4 If Y/PY/NI to 4.3: Could assessment of the outcome have been influenced by knowledge of intervention received? | | | PY | Both the intervention group and no intervention group could have rated their participation levels differently due to knowledge of group participation. Intervention groups likely more favorably due to getting to know and trust their therapists, and no intervention group by being aware that they did not get any intervention to improve participation as the other group did. |
|  | 4.5 If Y/PY/NI to 4.4: Is it likely that assessment of the outcome was influenced by knowledge of intervention received? | | | PY |  |
|  | Risk of bias judgement | | | High | Rating based on both the intervention group and no intervention group could have rated their participation levels differently due to knowledge of group participation, and that no information is available for when and how outcome measurements were measured in the groups Intervention groups possibly more favorably due to getting to know and trust their therapists, and control intervention group by being aware that they did not get any intervention to improve participation as the other group did. |
| Bias in selection of the reported result | 5.1 Were the data that produced this result analysed in accordance with a pre-specified analysis plan that was finalized before unblinded outcome data were available for analysis? | | | Y | Protocol published prospectively in 2008. |
|  | 5.2 ... multiple eligible outcome measurements (e.g. scales, definitions, time points) within the outcome domain? | | | N | All outcome measures mentioned in the protocol |
|  | 5.3 ... multiple eligible analyses of the data? | | | PN | Analysis methods reported in the protocol, but number of analyses and which are given priority across all time-points is not completely specified. |
|  | Risk of bias judgement | | | Low | Rating based on a protocol published prospectively in 2008 specifying all reported outcomes and adequately specifying the analyses conducted. |
| Overall bias | Risk of bias judgement | | | High | Overall high risk of bias due to high risk of bias in the measurement of the outcome due to participants rating their own participation levels, and some concers in the randomisation process due to unclear and large baseline differences, no description of methods for allocation consealment and some concerns for deviations from intended interventions due to having an inactive no intervention-comparison group. |
|  |  |  |  |  |  |
|  |  |  |  |  |  |
| Unique ID | Ada 2012 Minor adverse events 6 months | Study ID | Ada 2012 | Assessor | MR |
| Ref or Label |  | Aim | assignment to intervention (the 'intention-to-treat' effect) |  |  |
| Experimental |  | Comparator |  | Source | Journal article(s) |
| Outcome | Minor adverse events 6 months | Results |  | Weight | 1 |
| Domain | Signalling question | | | Response | Comments |
| Bias arising from the randomization process | 1.1 Was the allocation sequence random? | | | PY | States to be using a computer-generated randomization sequence, enrolling particiants in cohorts of 15 and by further dividing cohorts by matching based on walking speed of 3 in each subcohort. This indicates very small randomisation units. States to be "consealed" allocation but does not give any more information. |
|  | 1.2 Was the allocation sequence concealed until participants were enrolled and assigned to interventions? | | | PY |  |
|  | 1.3 Did baseline differences between intervention groups suggest a problem with the randomization process? | | | Y | Baseline inbalances for QoL (mean 61 vs 73) shows a 1 in around 100 chance of happening by chance, and participation (mean 20 vs 25) for 1 in around 20. Walking speed (232 vs 244) compatible to chance. |
|  | Risk of bias judgement | | | Some concerns | Rating based on on suboptimal randomisation method, unclear specification of allocation consealment and large baseline differences incompatible to chance for QoL and participation outcomes, which are the outcomes we include in our systematic review. |
| Bias due to deviations from intended interventions | 2.1.Were participants aware of their assigned intervention during the trial? | | | Y | Both participants and intervention personnell were aware of the assigned interventions. |
|  | 2.2.Were carers and people delivering the interventions aware of participants' assigned intervention during the trial? | | | Y |  |
|  | 2.3. If Y/PY/NI to 2.1 or 2.2: Were there deviations from the intended intervention that arose because of the experimental context? | | | PY | No clear information on deviations reported. Some indicators of deviations are present in form of behavior differences, i.e. the control group had a drop-off of 10% while intervention group had 0%. |
|  | 2.4 If Y/PY to 2.3: Were these deviations likely to have affected the outcome? | | | PY | Since the control group did not get any intervention, it would be likely that they behaved differently compared to the intervention group due to the trial conext. |
|  | 2.5. If Y/PY/NI to 2.4: Were these deviations from intended intervention balanced between groups? | | | PN | Any deviations would probably not be balanced between the groups. |
|  | 2.6 Was an appropriate analysis used to estimate the effect of assignment to intervention? | | | PY | ITT analysis was used, imputing values for missing data. |
|  | 2.7 If N/PN/NI to 2.6: Was there potential for a substantial impact (on the result) of the failure to analyse participants in the group to which they were randomized? | | | NA |  |
|  | Risk of bias judgement | | | High | Both participants and intervention personnell were aware of the assigned interventions. No clear information on deviations reported. Some indicators of deviations are present in form of behavior differences, i.e. the control group had a drop-off of 10% while intervention group had 0%.  Since the control group did not get any intervention, it would be likely that they behaved differently compared to the intervention group due to the trial conext.  Any deviations would probably not be balanced between the groups.  ITT analysis was used, imputing values for missing data. |
| Bias due to missing outcome data | 3.1 Were data for this outcome available for all, or nearly all, participants randomized? | | | PN | Missing data for no intervention group is 5/34 |
|  | 3.2 If N/PN/NI to 3.1: Is there evidence that result was not biased by missing outcome data? | | | PN | No evidence of any way to minimize or explore bias |
|  | 3.3 If N/PN to 3.2: Could missingness in the outcome depend on its true value? | | | PY | Participants that have a higher likelyhood of falls could be more prone to leave the no intervention group since they do not get any intervention to tackle this problem. 15%, or 5 out of 34 were missing for thus outcome domain for the control group. |
|  | 3.4 If Y/PY/NI to 3.3: Is it likely that missingness in the outcome depended on its true value? | | | PY |  |
|  | Risk of bias judgement | | | High | Participants that have a higher likelyhood of falls could be more prone to leave the no intervention group since they do not get any intervention to tackle this problem. 15%, or 5 out of 34 were missing for thus outcome domain for the control group. |
| Bias in measurement of the outcome | 4.1 Was the method of measuring the outcome inappropriate? | | | PN | Using self-reported falls collected in a journal reported on a monthly basis, unclear if this is a valid way to measure the number of falls for participants in both the intervention and inactive control group |
|  | 4.2 Could measurement or ascertainment of the outcome have differed between intervention groups? | | | PY | As the intervention group was meeting regularly with therapists and therefore was reminded about filling out falls monthly compared to the inactive control group, it is possible that the reporting could differ between groups |
|  | 4.3 Were outcome assessors aware of the intervention received by study participants? | | | NA |  |
|  | 4.4 If Y/PY/NI to 4.3: Could assessment of the outcome have been influenced by knowledge of intervention received? | | | NA |  |
|  | 4.5 If Y/PY/NI to 4.4: Is it likely that assessment of the outcome was influenced by knowledge of intervention received? | | | NA |  |
|  | Risk of bias judgement | | | High | Using self-reported falls collected in a journal reported on a monthly basis, unclear if this is a valid way to measure the number of falls for participants in both the intervention and inactive control group As the intervention group was meeting regularly with therapists and therefore was reminded about filling out falls monthly compared to the inactive control group, it is possible that the reporting could differ between groups as they were filled out in a journal each month. |
| Bias in selection of the reported result | 5.1 Were the data that produced this result analysed in accordance with a pre-specified analysis plan that was finalized before unblinded outcome data were available for analysis? | | | Y | Protocol published prospectively in 2008. |
|  | 5.2 ... multiple eligible outcome measurements (e.g. scales, definitions, time points) within the outcome domain? | | | N | All outcome measures mentioned in the protocol |
|  | 5.3 ... multiple eligible analyses of the data? | | | PN | Analysis methods reported in the protocol, but number of analyses and which are given priority across all time-points is not completely specified. |
|  | Risk of bias judgement | | | Low | Rating based on a protocol published prospectively in 2008 specifying all reported outcomes and adequately specifying the analyses conducted. |
| Overall bias | Risk of bias judgement | | | High | Overall high risk of bias due to high risk of bias in the measurement of the outcome due to participants rating their own participation levels, and some concers in the randomisation process due to unclear and large baseline differences, no description of methods for allocation consealment and some concerns for deviations from intended interventions due to having an inactive no intervention-comparison group. |
|  |  |  |  |  |  |
|  |  |  |  |  |  |
| Unique ID | Ada 2012 Minor adverse events 7 months | Study ID | Ada 2012 | Assessor | MR |
| Ref or Label |  | Aim | assignment to intervention (the 'intention-to-treat' effect) |  |  |
| Experimental |  | Comparator |  | Source | Journal article(s) |
| Outcome | Minor adverse events 7 months | Results |  | Weight | 1 |
| Domain | Signalling question | | | Response | Comments |
| Bias arising from the randomization process | 1.1 Was the allocation sequence random? | | | PY | States to be using a computer-generated randomization sequence, enrolling particiants in cohorts of 15 and by further dividing cohorts by matching based on walking speed of 3 in each subcohort. This indicates very small randomisation units. States to be "consealed" allocation but does not give any more information. |
|  | 1.2 Was the allocation sequence concealed until participants were enrolled and assigned to interventions? | | | PY |  |
|  | 1.3 Did baseline differences between intervention groups suggest a problem with the randomization process? | | | Y | Baseline inbalances for QoL (mean 61 vs 73) shows a 1 in around 100 chance of happening by chance, and participation (mean 20 vs 25) for 1 in around 20. Walking speed (232 vs 244) compatible to chance. |
|  | Risk of bias judgement | | | Some concerns | Rating based on on suboptimal randomisation method, unclear specification of allocation consealment and large baseline differences incompatible to chance for QoL and participation outcomes, which are the outcomes we include in our systematic review. |
| Bias due to deviations from intended interventions | 2.1.Were participants aware of their assigned intervention during the trial? | | | Y | Both participants and intervention personnell were aware of the assigned interventions. |
|  | 2.2.Were carers and people delivering the interventions aware of participants' assigned intervention during the trial? | | | Y |  |
|  | 2.3. If Y/PY/NI to 2.1 or 2.2: Were there deviations from the intended intervention that arose because of the experimental context? | | | PY | No clear information on deviations reported. Some indicators of deviations are present in form of behavior differences, i.e. the control group had a drop-off of 10% while intervention group had 0%. |
|  | 2.4 If Y/PY to 2.3: Were these deviations likely to have affected the outcome? | | | PY | Since the control group did not get any intervention, it would be likely that they behaved differently compared to the intervention group due to the trial conext. |
|  | 2.5. If Y/PY/NI to 2.4: Were these deviations from intended intervention balanced between groups? | | | PN | Any deviations would probably not be balanced between the groups. |
|  | 2.6 Was an appropriate analysis used to estimate the effect of assignment to intervention? | | | PY | ITT analysis was used, imputing values for missing data. |
|  | 2.7 If N/PN/NI to 2.6: Was there potential for a substantial impact (on the result) of the failure to analyse participants in the group to which they were randomized? | | | NA |  |
|  | Risk of bias judgement | | | High | Both participants and intervention personnell were aware of the assigned interventions. No clear information on deviations reported. Some indicators of deviations are present in form of behavior differences, i.e. the control group had a drop-off of 10% while intervention group had 0%.  Since the control group did not get any intervention, it would be likely that they behaved differently compared to the intervention group due to the trial conext.  Any deviations would probably not be balanced between the groups.  ITT analysis was used, imputing values for missing data. |
| Bias due to missing outcome data | 3.1 Were data for this outcome available for all, or nearly all, participants randomized? | | | PN | Missing data for no intervention group is 7/34 |
|  | 3.2 If N/PN/NI to 3.1: Is there evidence that result was not biased by missing outcome data? | | | PN | No evidence of any way to minimize or explore bias |
|  | 3.3 If N/PN to 3.2: Could missingness in the outcome depend on its true value? | | | PY | Participants that have a higher likelyhood of falls could be more prone to leave the no intervention group since they do not get any intervention to tackle this problem. 20%, or 7 out of 34 were missing for thus outcome domain for the control group. |
|  | 3.4 If Y/PY/NI to 3.3: Is it likely that missingness in the outcome depended on its true value? | | | PY |  |
|  | Risk of bias judgement | | | High | Participants that have a higher likelyhood of falls could be more prone to leave the no intervention group since they do not get any intervention to tackle this problem. 15%, or 7 out of 34 were missing for thus outcome domain for the control group. |
| Bias in measurement of the outcome | 4.1 Was the method of measuring the outcome inappropriate? | | | PN | Using self-reported falls collected in a journal reported on a monthly basis, unclear if this is a valid way to measure the number of falls for participants in both the intervention and inactive control group |
|  | 4.2 Could measurement or ascertainment of the outcome have differed between intervention groups? | | | PY | As the intervention group was meeting regularly with therapists and therefore was reminded about filling out falls monthly compared to the inactive control group, it is possible that the reporting could differ between groups |
|  | 4.3 Were outcome assessors aware of the intervention received by study participants? | | | NA |  |
|  | 4.4 If Y/PY/NI to 4.3: Could assessment of the outcome have been influenced by knowledge of intervention received? | | | NA |  |
|  | 4.5 If Y/PY/NI to 4.4: Is it likely that assessment of the outcome was influenced by knowledge of intervention received? | | | NA |  |
|  | Risk of bias judgement | | | High | Using self-reported falls collected in a journal reported on a monthly basis, unclear if this is a valid way to measure the number of falls for participants in both the intervention and inactive control group As the intervention group was meeting regularly with therapists and therefore was reminded about filling out falls monthly compared to the inactive control group, it is possible that the reporting could differ between groups as they were filled out in a journal each month. |
| Bias in selection of the reported result | 5.1 Were the data that produced this result analysed in accordance with a pre-specified analysis plan that was finalized before unblinded outcome data were available for analysis? | | | Y | Protocol published prospectively in 2008. |
|  | 5.2 ... multiple eligible outcome measurements (e.g. scales, definitions, time points) within the outcome domain? | | | N | All outcome measures mentioned in the protocol |
|  | 5.3 ... multiple eligible analyses of the data? | | | PN | Analysis methods reported in the protocol, but number of analyses and which are given priority across all time-points is not completely specified. |
|  | Risk of bias judgement | | | Low | Rating based on a protocol published prospectively in 2008 specifying all reported outcomes and adequately specifying the analyses conducted. |
| Overall bias | Risk of bias judgement | | | High | Overall high risk of bias due to high risk of bias in the measurement of the outcome due to participants rating their own participation levels, and some concers in the randomisation process due to unclear and large baseline differences, no description of methods for allocation consealment and some concerns for deviations from intended interventions due to having an inactive no intervention-comparison group. |
|  |  |  |  |  |  |
|  |  |  |  |  |  |
| Unique ID | Ada 2012 Participation 6 months | Study ID | Ada 2012 | Assessor | MR |
| Ref or Label |  | Aim | assignment to intervention (the 'intention-to-treat' effect) |  |  |
| Experimental |  | Comparator |  | Source | Journal article(s) |
| Outcome | Participation 6 months | Results |  | Weight | 1 |
| Domain | Signalling question | | | Response | Comments |
| Bias arising from the randomization process | 1.1 Was the allocation sequence random? | | | PY | States to be using a computer-generated randomization sequence, enrolling particiants in cohorts of 15 and by further dividing cohorts by matching based on walking speed of 3 in each subcohort. This indicates very small randomisation units. States to be "consealed" allocation but does not give any more information. |
|  | 1.2 Was the allocation sequence concealed until participants were enrolled and assigned to interventions? | | | PY |  |
|  | 1.3 Did baseline differences between intervention groups suggest a problem with the randomization process? | | | Y | Baseline inbalances for QoL (mean 61 vs 73) shows a 1 in around 100 chance of happening by chance, and participation (mean 20 vs 25) for 1 in around 20. Walking speed (232 vs 244) compatible to chance. |
|  | Risk of bias judgement | | | Some concerns | Rating based on on suboptimal randomisation method, unclear specification of allocation consealment and large baseline differences incompatible to chance for QoL and participation outcomes, which are the outcomes we include in our systematic review. |
| Bias due to deviations from intended interventions | 2.1.Were participants aware of their assigned intervention during the trial? | | | Y | Both participants and intervention personnell were aware of the assigned interventions. |
|  | 2.2.Were carers and people delivering the interventions aware of participants' assigned intervention during the trial? | | | Y |  |
|  | 2.3. If Y/PY/NI to 2.1 or 2.2: Were there deviations from the intended intervention that arose because of the experimental context? | | | PY | No clear information on deviations reported. Some indicators of deviations are present in form of behavior differences, i.e. the control group had a drop-off of 10% while intervention group had 0%. |
|  | 2.4 If Y/PY to 2.3: Were these deviations likely to have affected the outcome? | | | PY | Since the control group did not get any intervention, it would be likely that they behaved differently compared to the intervention group due to the trial conext. |
|  | 2.5. If Y/PY/NI to 2.4: Were these deviations from intended intervention balanced between groups? | | | PN | Any deviations would probably not be balanced between the groups. |
|  | 2.6 Was an appropriate analysis used to estimate the effect of assignment to intervention? | | | PY | ITT analysis was used, imputing values for missing data. |
|  | 2.7 If N/PN/NI to 2.6: Was there potential for a substantial impact (on the result) of the failure to analyse participants in the group to which they were randomized? | | | NA |  |
|  | Risk of bias judgement | | | High | Both participants and intervention personnell were aware of the assigned interventions. No clear information on deviations reported. Some indicators of deviations are present in form of behavior differences, i.e. the control group had a drop-off of 10% while intervention group had 0%.  Since the control group did not get any intervention, it would be likely that they behaved differently compared to the intervention group due to the trial conext.  Any deviations would probably not be balanced between the groups.  ITT analysis was used, imputing values for missing data. |
| Bias due to missing outcome data | 3.1 Were data for this outcome available for all, or nearly all, participants randomized? | | | PY | 94% available data at 6 months in all three groups, while at least 3 out of 34 missing data in the no intervention group. Probably very small or no impact of the overall minor missing data on continuous outcome data. |
|  | 3.2 If N/PN/NI to 3.1: Is there evidence that result was not biased by missing outcome data? | | | NA |  |
|  | 3.3 If N/PN to 3.2: Could missingness in the outcome depend on its true value? | | | NA |  |
|  | 3.4 If Y/PY/NI to 3.3: Is it likely that missingness in the outcome depended on its true value? | | | NA |  |
|  | Risk of bias judgement | | | Low | Rating based on 94% available data at 6 months in all three groups, while at least 3 out of 34 missing data in the no intervention group. Probably very small or no impact of the overall minor missing data on continuous outcome data. Missing data imputed from LOCF. |
| Bias in measurement of the outcome | 4.1 Was the method of measuring the outcome inappropriate? | | | N | Using a validated participation scale |
|  | 4.2 Could measurement or ascertainment of the outcome have differed between intervention groups? | | | NI | No information provided |
|  | 4.3 Were outcome assessors aware of the intervention received by study participants? | | | Y | Participants rating their outcomes were aware of the intervention recieved |
|  | 4.4 If Y/PY/NI to 4.3: Could assessment of the outcome have been influenced by knowledge of intervention received? | | | PY | Both the intervention group and no intervention group could have rated their participation levels differently due to knowledge of group participation. Intervention groups likely more favorably due to getting to know and trust their therapists, and no intervention group by being aware that they did not get any intervention to improve participation as the other group did. |
|  | 4.5 If Y/PY/NI to 4.4: Is it likely that assessment of the outcome was influenced by knowledge of intervention received? | | | PY |  |
|  | Risk of bias judgement | | | High | Rating based on both the intervention group and no intervention group could have rated their participation levels differently due to knowledge of group participation, and that no information is available for when and how outcome measurements were measured in the groups Intervention groups possibly more favorably due to getting to know and trust their therapists, and control intervention group by being aware that they did not get any intervention to improve participation as the other group did. |
| Bias in selection of the reported result | 5.1 Were the data that produced this result analysed in accordance with a pre-specified analysis plan that was finalized before unblinded outcome data were available for analysis? | | | Y | Protocol published prospectively in 2008. |
|  | 5.2 ... multiple eligible outcome measurements (e.g. scales, definitions, time points) within the outcome domain? | | | N | All outcome measures mentioned in the protocol |
|  | 5.3 ... multiple eligible analyses of the data? | | | PN | Analysis methods reported in the protocol, but number of analyses and which are given priority across all time-points is not completely specified. |
|  | Risk of bias judgement | | | Low | Rating based on a protocol published prospectively in 2008 specifying all reported outcomes and adequately specifying the analyses conducted. |
| Overall bias | Risk of bias judgement | | | High | Overall high risk of bias due to high risk of bias in the measurement of the outcome due to participants rating their own participation levels, and some concers in the randomisation process due to unclear and large baseline differences, no description of methods for allocation consealment and some concerns for deviations from intended interventions due to having an inactive no intervention-comparison group. |
|  |  |  |  |  |  |
|  |  |  |  |  |  |
| Unique ID | Ada 2012 Participation 7 months | Study ID | Ada 2012 | Assessor | MR |
| Ref or Label |  | Aim | assignment to intervention (the 'intention-to-treat' effect) |  |  |
| Experimental |  | Comparator |  | Source | Journal article(s) |
| Outcome | Participation 7 months | Results |  | Weight | 1 |
| Domain | Signalling question | | | Response | Comments |
| Bias arising from the randomization process | 1.1 Was the allocation sequence random? | | | PY | States to be using a computer-generated randomization sequence, enrolling particiants in cohorts of 15 and by further dividing cohorts by matching based on walking speed of 3 in each subcohort. This indicates very small randomisation units. States to be "consealed" allocation but does not give any more information. |
|  | 1.2 Was the allocation sequence concealed until participants were enrolled and assigned to interventions? | | | PY |  |
|  | 1.3 Did baseline differences between intervention groups suggest a problem with the randomization process? | | | Y | Baseline inbalances for QoL (mean 61 vs 73) shows a 1 in around 100 chance of happening by chance, and participation (mean 20 vs 25) for 1 in around 20. Walking speed (232 vs 244) compatible to chance. |
|  | Risk of bias judgement | | | Some concerns | Rating based on on suboptimal randomisation method, unclear specification of allocation consealment and large baseline differences incompatible to chance for QoL and participation outcomes, which are the outcomes we include in our systematic review. |
| Bias due to deviations from intended interventions | 2.1.Were participants aware of their assigned intervention during the trial? | | | Y | Both participants and intervention personnell were aware of the assigned interventions. |
|  | 2.2.Were carers and people delivering the interventions aware of participants' assigned intervention during the trial? | | | Y |  |
|  | 2.3. If Y/PY/NI to 2.1 or 2.2: Were there deviations from the intended intervention that arose because of the experimental context? | | | PY | No clear information on deviations reported. Some indicators of deviations are present in form of behavior differences, i.e. the control group had a drop-off of 10% while intervention group had 0%. |
|  | 2.4 If Y/PY to 2.3: Were these deviations likely to have affected the outcome? | | | PY | Since the control group did not get any intervention, it would be likely that they behaved differently compared to the intervention group due to the trial conext. |
|  | 2.5. If Y/PY/NI to 2.4: Were these deviations from intended intervention balanced between groups? | | | PN | Any deviations would probably not be balanced between the groups. |
|  | 2.6 Was an appropriate analysis used to estimate the effect of assignment to intervention? | | | PY | ITT analysis was used, imputing values for missing data. |
|  | 2.7 If N/PN/NI to 2.6: Was there potential for a substantial impact (on the result) of the failure to analyse participants in the group to which they were randomized? | | | NA |  |
|  | Risk of bias judgement | | | High | Both participants and intervention personnell were aware of the assigned interventions. No clear information on deviations reported. Some indicators of deviations are present in form of behavior differences, i.e. the control group had a drop-off of 10% while intervention group had 0%.  Since the control group did not get any intervention, it would be likely that they behaved differently compared to the intervention group due to the trial conext.  Any deviations would probably not be balanced between the groups.  ITT analysis was used, imputing values for missing data. |
| Bias due to missing outcome data | 3.1 Were data for this outcome available for all, or nearly all, participants randomized? | | | PY | 94% available data at 6 months in all three groups, while at least 3 out of 34 missing data in the no intervention group. Probably very small or no impact of the overall minor missing data on continuous outcome data. |
|  | 3.2 If N/PN/NI to 3.1: Is there evidence that result was not biased by missing outcome data? | | | NA |  |
|  | 3.3 If N/PN to 3.2: Could missingness in the outcome depend on its true value? | | | NA |  |
|  | 3.4 If Y/PY/NI to 3.3: Is it likely that missingness in the outcome depended on its true value? | | | NA |  |
|  | Risk of bias judgement | | | Low | Rating based on 94% available data at 6 months in all three groups, while at least 3 out of 34 missing data in the no intervention group. Probably very small or no impact of the overall minor missing data on continuous outcome data. Missing data imputed from LOCF. |
| Bias in measurement of the outcome | 4.1 Was the method of measuring the outcome inappropriate? | | | N | Using a validated participation scale |
|  | 4.2 Could measurement or ascertainment of the outcome have differed between intervention groups? | | | NI | No information provided |
|  | 4.3 Were outcome assessors aware of the intervention received by study participants? | | | Y | Participants rating their outcomes were aware of the intervention recieved |
|  | 4.4 If Y/PY/NI to 4.3: Could assessment of the outcome have been influenced by knowledge of intervention received? | | | PY | Both the intervention group and no intervention group could have rated their participation levels differently due to knowledge of group participation. Intervention groups likely more favorably due to getting to know and trust their therapists, and no intervention group by being aware that they did not get any intervention to improve participation as the other group did. |
|  | 4.5 If Y/PY/NI to 4.4: Is it likely that assessment of the outcome was influenced by knowledge of intervention received? | | | PY |  |
|  | Risk of bias judgement | | | High | Rating based on both the intervention group and no intervention group could have rated their participation levels differently due to knowledge of group participation, and that no information is available for when and how outcome measurements were measured in the groups Intervention groups possibly more favorably due to getting to know and trust their therapists, and control intervention group by being aware that they did not get any intervention to improve participation as the other group did. |
| Bias in selection of the reported result | 5.1 Were the data that produced this result analysed in accordance with a pre-specified analysis plan that was finalized before unblinded outcome data were available for analysis? | | | Y | Protocol published prospectively in 2008. |
|  | 5.2 ... multiple eligible outcome measurements (e.g. scales, definitions, time points) within the outcome domain? | | | N | All outcome measures mentioned in the protocol |
|  | 5.3 ... multiple eligible analyses of the data? | | | PN | Analysis methods reported in the protocol, but number of analyses and which are given priority across all time-points is not completely specified. |
|  | Risk of bias judgement | | | Low | Rating based on a protocol published prospectively in 2008 specifying all reported outcomes and adequately specifying the analyses conducted. |
| Overall bias | Risk of bias judgement | | | High | Overall high risk of bias due to high risk of bias in the measurement of the outcome due to participants rating their own participation levels, and some concers in the randomisation process due to unclear and large baseline differences, no description of methods for allocation consealment and some concerns for deviations from intended interventions due to having an inactive no intervention-comparison group. |
|  |  |  |  |  |  |
|  |  |  |  |  |  |
| Unique ID | Logan 2004 Activity outside the home 6 months | Study ID | Logan 2004 | Assessor | MR |
| Ref or Label |  | Aim | assignment to intervention (the 'intention-to-treat' effect) |  |  |
| Experimental |  | Comparator |  | Source | Journal article(s) |
| Outcome | Activity outside the home 6 months | Results |  | Weight | 1 |
| Domain | Signalling question | | | Response | Comments |
| Bias arising from the randomization process | 1.1 Was the allocation sequence random? | | | PY | Computer generated random sequence, stratified by age and dependency on travel by external telephone randomisation, which we assume is consealed. |
|  | 1.2 Was the allocation sequence concealed until participants were enrolled and assigned to interventions? | | | PY |  |
|  | 1.3 Did baseline differences between intervention groups suggest a problem with the randomization process? | | | PY | Some gender differences (46% vs 62% men) that happens about 1 in 20 due to chance, and for getting out of the house (28% vs 39%) that happens around 1 out of 9 due to chance. Assumed to probably be due to chance. |
|  | Risk of bias judgement | | | Some concerns | Computer generated random sequence, stratified by age and dependency on travel by external telephone randomisation, which we assume is consealed. Some gender differences (46% vs 62% men) that happens about 1 in 20 due to chance, and for getting out of the house (28% vs 39&) that happens around 1 out of 9 due to chance. Assumed to potentially not be due to chance, since these are prognostic variables of the outcomes. |
| Bias due to deviations from intended interventions | 2.1.Were participants aware of their assigned intervention during the trial? | | | Y | Participants and personnel were aware of the assigned intervention. |
|  | 2.2.Were carers and people delivering the interventions aware of participants' assigned intervention during the trial? | | | Y |  |
|  | 2.3. If Y/PY/NI to 2.1 or 2.2: Were there deviations from the intended intervention that arose because of the experimental context? | | | PY | Authors does not analyse or report any specific deviations from the two interventions, but there is a large drop-out from the control intervention (26%) compared to intervention group (12%) indicating a difference in behavior between the two groups due to being in the trial and knowing the allocation of groups. |
|  | 2.4 If Y/PY to 2.3: Were these deviations likely to have affected the outcome? | | | PN | Since the aim of the intervention is to improve outdoor mobility, and the outcome is outdoor mobility, we could assume that the deviations would not have a substancial effect on the outcomes. There is also some attention control as the control group gets one visit and some information about mobility. |
|  | 2.5. If Y/PY/NI to 2.4: Were these deviations from intended intervention balanced between groups? | | | NA |  |
|  | 2.6 Was an appropriate analysis used to estimate the effect of assignment to intervention? | | | PY | Modified intention to treat-analysis, imputing values for missing outcomes, is used and would be considered to be an appropriate analysis. |
|  | 2.7 If N/PN/NI to 2.6: Was there potential for a substantial impact (on the result) of the failure to analyse participants in the group to which they were randomized? | | | NA |  |
|  | Risk of bias judgement | | | Some concerns | Participants and personnel were aware of the assigned intervention. Authors does not analyse or report any specific deviations from the two interventions, but there is a large drop-out from the control intervention (26%) compared to intervention group (12%) indicating a difference in behavior between the two groups due to being in the trial and knowing the allocation of groups.  Since the aim of the intervention is to improve outdoor mobility, and the outcome is outdoor mobility, we could assume that the deviations would not have a substancial effect on the outcomes. There is also some attention control as the control group gets one visit and some information about mobility, limiting the risk of deviations to do other interventions for the control group somewhat. Modified intention to treat-analysis is used, imputing values for missing outcomes, is used and would be considered to be an appropriate analysis. |
| Bias due to missing outcome data | 3.1 Were data for this outcome available for all, or nearly all, participants randomized? | | | PN | Control group has a 10% drop out at 4 months and 25% at 10 months and intervention group has 2% and 12% respectively |
|  | 3.2 If N/PN/NI to 3.1: Is there evidence that result was not biased by missing outcome data? | | | PN | Authors impute values based on previous measurement points (e.g. follow up at 4 month or baseline), which only mitigates any missing outcome data concerns to a small extent. |
|  | 3.3 If N/PN to 3.2: Could missingness in the outcome depend on its true value? | | | Y | Authors do not state any reasons for drop-outs in the groups, but the differences are large between groups. Missing data could influence the outcome and chance the results. |
|  | 3.4 If Y/PY/NI to 3.3: Is it likely that missingness in the outcome depended on its true value? | | | PY |  |
|  | Risk of bias judgement | | | High | Control group has a 10% drop out at 4 months and 25% at 10 months and intervention group has 2% and 12% respectively Authors impute values based on previous measurement points (e.g. follow up at 4 month or baseline), which only mitigates any missing outcome data concerns to a small extent. Authors do not state any reasons for drop-outs in the groups, but the differences are large between groups. Missing data could influence the outcome and chance the results. |
| Bias in measurement of the outcome | 4.1 Was the method of measuring the outcome inappropriate? | | | PN | Self-reported measures of mobility are common and possibly reliable and valid, although there are more appropriate objective methods to measure this outcome by. |
|  | 4.2 Could measurement or ascertainment of the outcome have differed between intervention groups? | | | PN | Measured by post, but we are not told how many in each group had to be contacted by the study administrators to clarify outcomes that were not reported or correctly reported. |
|  | 4.3 Were outcome assessors aware of the intervention received by study participants? | | | Y | The participants rated their own outcomes are were aware of their group allocation. |
|  | 4.4 If Y/PY/NI to 4.3: Could assessment of the outcome have been influenced by knowledge of intervention received? | | | Y | Knowledge of the intervention could influence the self-reported outcome. Since the control intervention contained some time spent to recommend outdoor mobility, the likelyhood of these being substancially different between groups are smaller. |
|  | 4.5 If Y/PY/NI to 4.4: Is it likely that assessment of the outcome was influenced by knowledge of intervention received? | | | PN |  |
|  | Risk of bias judgement | | | Some concerns | Self-reported measures of mobility are common and possibly reliable and valid, although there are more appropriate objective methods to measure this outcome by. Measured by post, but we are not told how many in each group had to be contacted by the study administrators to clarify outcomes that were not reported or correctly reported. The participants rated their own outcomes are were aware of their group allocation.  Knowledge of the intervention could influence the self-reported outcome. Since the control intervention contained some time spent to recommend outdoor mobility, the likelyhood of these being substancially different between groups are smaller. |
| Bias in selection of the reported result | 5.1 Were the data that produced this result analysed in accordance with a pre-specified analysis plan that was finalized before unblinded outcome data were available for analysis? | | | NI | No mention of a protocol and no protocol found after a search. |
|  | 5.2 ... multiple eligible outcome measurements (e.g. scales, definitions, time points) within the outcome domain? | | | PN | Outcomes seem to be transparently reported. |
|  | 5.3 ... multiple eligible analyses of the data? | | | PN | Using analyses to adjust for baseline differences that seem pre-specified, so probably done according to a plan set before looking at the actual baseline data. |
|  | Risk of bias judgement | | | Some concerns | No mention of a protocol and no protocol found after a search. |
| Overall bias | Risk of bias judgement | | | High | Overall high risk of bias due to high risk of bias due to missing outcome data, and some concerns due to non-blinding of participants, outcome assessors, and no available protocol. |
|  |  |  |  |  |  |
|  |  |  |  |  |  |
| Unique ID | Logan 2004 Activity outside the home 7 months | Study ID | Logan 2004 | Assessor | MR |
| Ref or Label |  | Aim | assignment to intervention (the 'intention-to-treat' effect) |  |  |
| Experimental |  | Comparator |  | Source | Journal article(s) |
| Outcome | Activity outside the home 7 months | Results |  | Weight | 1 |
| Domain | Signalling question | | | Response | Comments |
| Bias arising from the randomization process | 1.1 Was the allocation sequence random? | | | PY | Computer generated random sequence, stratified by age and dependency on travel by external telephone randomisation, which we assume is consealed. |
|  | 1.2 Was the allocation sequence concealed until participants were enrolled and assigned to interventions? | | | NI |  |
|  | 1.3 Did baseline differences between intervention groups suggest a problem with the randomization process? | | | PN | Some gender differences (46% vs 62% men) that happens about 1 in 20 due to chance, and for getting out of the house (28% vs 39&) that happens around 1 out of 9 due to chance. Assumed to probably be due to chance. |
|  | Risk of bias judgement | | | Some concerns | Computer generated random sequence, stratified by age and dependency on travel by external telephone randomisation, which we assume is consealed. Some gender differences (46% vs 62% men) that happens about 1 in 20 due to chance, and for getting out of the house (28% vs 39&) that happens around 1 out of 9 due to chance. Assumed to probably not be due to chance. |
| Bias due to deviations from intended interventions | 2.1.Were participants aware of their assigned intervention during the trial? | | | Y | Participants and personnel were aware of the assigned intervention. |
|  | 2.2.Were carers and people delivering the interventions aware of participants' assigned intervention during the trial? | | | Y |  |
|  | 2.3. If Y/PY/NI to 2.1 or 2.2: Were there deviations from the intended intervention that arose because of the experimental context? | | | PY | Authors does not analyse or report any specific deviations from the two interventions, but there is a large drop-out from the control intervention (26%) compared to intervention group (12%) indicating a difference in behavior between the two groups due to being in the trial and knowing the allocation of groups. |
|  | 2.4 If Y/PY to 2.3: Were these deviations likely to have affected the outcome? | | | PN | Since the aim of the intervention is to improve outdoor mobility, and the outcome is outdoor mobility, we could assume that the deviations would not have a substancial effect on the outcomes. There is also some attention control as the control group gets one visit and some information about mobility. |
|  | 2.5. If Y/PY/NI to 2.4: Were these deviations from intended intervention balanced between groups? | | | NA |  |
|  | 2.6 Was an appropriate analysis used to estimate the effect of assignment to intervention? | | | PY | Modified intention to treat-analysis, imputing values for missing outcomes, is used and would be considered to be an appropriate analysis. |
|  | 2.7 If N/PN/NI to 2.6: Was there potential for a substantial impact (on the result) of the failure to analyse participants in the group to which they were randomized? | | | NA |  |
|  | Risk of bias judgement | | | Some concerns | Participants and personnel were aware of the assigned intervention. Authors does not analyse or report any specific deviations from the two interventions, but there is a large drop-out from the control intervention (26%) compared to intervention group (12%) indicating a difference in behavior between the two groups due to being in the trial and knowing the allocation of groups.  Since the aim of the intervention is to improve outdoor mobility, and the outcome is outdoor mobility, we could assume that the deviations would not have a substancial effect on the outcomes. There is also some attention control as the control group gets one visit and some information about mobility, limiting the risk of deviations to do other interventions for the control group somewhat. Modified intention to treat-analysis is used, imputing values for missing outcomes, is used and would be considered to be an appropriate analysis. |
| Bias due to missing outcome data | 3.1 Were data for this outcome available for all, or nearly all, participants randomized? | | | PN | Control group has a 10% drop out at 4 months and 25% at 10 months and intervention group has 2% and 12% respectively |
|  | 3.2 If N/PN/NI to 3.1: Is there evidence that result was not biased by missing outcome data? | | | PN | Authors impute values based on previous measurement points (e.g. follow up at 4 month or baseline), which only mitigates any missing outcome data concerns to a small extent. |
|  | 3.3 If N/PN to 3.2: Could missingness in the outcome depend on its true value? | | | Y | Authors do not state any reasons for drop-outs in the groups, but the differences are large between groups. Missing data could influence the outcome and chance the results. |
|  | 3.4 If Y/PY/NI to 3.3: Is it likely that missingness in the outcome depended on its true value? | | | PY |  |
|  | Risk of bias judgement | | | High | Control group has a 10% drop out at 4 months and 25% at 10 months and intervention group has 2% and 12% respectively Authors impute values based on previous measurement points (e.g. follow up at 4 month or baseline), which only mitigates any missing outcome data concerns to a small extent. Authors do not state any reasons for drop-outs in the groups, but the differences are large between groups. Missing data could influence the outcome and chance the results. |
| Bias in measurement of the outcome | 4.1 Was the method of measuring the outcome inappropriate? | | | PN | Self-reported measures of mobility are common and possibly reliable and valid, although there are more appropriate objective methods to measure this outcome by. |
|  | 4.2 Could measurement or ascertainment of the outcome have differed between intervention groups? | | | PN | Measured by post, but we are not told how many in each group had to be contacted by the study administrators to clarify outcomes that were not reported or correctly reported. |
|  | 4.3 Were outcome assessors aware of the intervention received by study participants? | | | Y | The participants rated their own outcomes are were aware of their group allocation. |
|  | 4.4 If Y/PY/NI to 4.3: Could assessment of the outcome have been influenced by knowledge of intervention received? | | | Y | Knowledge of the intervention could influence the self-reported outcome. Since the control intervention contained some time spent to recommend outdoor mobility, the likelyhood of these being substancially different between groups are smaller. |
|  | 4.5 If Y/PY/NI to 4.4: Is it likely that assessment of the outcome was influenced by knowledge of intervention received? | | | PN |  |
|  | Risk of bias judgement | | | Some concerns | Self-reported measures of mobility are common and possibly reliable and valid, although there are more appropriate objective methods to measure this outcome by. Measured by post, but we are not told how many in each group had to be contacted by the study administrators to clarify outcomes that were not reported or correctly reported. The participants rated their own outcomes are were aware of their group allocation.  Knowledge of the intervention could influence the self-reported outcome. Since the control intervention contained some time spent to recommend outdoor mobility, the likelyhood of these being substancially different between groups are smaller. |
| Bias in selection of the reported result | 5.1 Were the data that produced this result analysed in accordance with a pre-specified analysis plan that was finalized before unblinded outcome data were available for analysis? | | | NI | No mention of a protocol and no protocol found after a search. |
|  | 5.2 ... multiple eligible outcome measurements (e.g. scales, definitions, time points) within the outcome domain? | | | PN | Outcomes seem to be transparently reported. |
|  | 5.3 ... multiple eligible analyses of the data? | | | PN | Using analyses to adjust for baseline differences that seem pre-specified, so probably done according to a plan set before looking at the actual baseline data. |
|  | Risk of bias judgement | | | Some concerns | No mention of a protocol and no protocol found after a search. |
| Overall bias | Risk of bias judgement | | | High | Overall high risk of bias due to high risk of bias due to missing outcome data, and some concerns due to non-blinding of participants, outcome assessors, and no available protocol. |
|  |  |  |  |  |  |
|  |  |  |  |  |  |
| Unique ID | Logan 2004 Engagement in everyday life activities 6 months | Study ID | Logan 2004 | Assessor | MR |
| Ref or Label |  | Aim | assignment to intervention (the 'intention-to-treat' effect) |  |  |
| Experimental |  | Comparator |  | Source | Journal article(s) |
| Outcome | Engagement in erveryday life activities 6 months | Results |  | Weight | 1 |
| Domain | Signalling question | | | Response | Comments |
| Bias arising from the randomization process | 1.1 Was the allocation sequence random? | | | PY | Computer generated random sequence, stratified by age and dependency on travel by external telephone randomisation, which we assume is consealed. |
|  | 1.2 Was the allocation sequence concealed until participants were enrolled and assigned to interventions? | | | NI |  |
|  | 1.3 Did baseline differences between intervention groups suggest a problem with the randomization process? | | | PN | Some gender differences (46% vs 62% men) that happens about 1 in 20 due to chance, and for getting out of the house (28% vs 39&) that happens around 1 out of 9 due to chance. Assumed to probably be due to chance. |
|  | Risk of bias judgement | | | Some concerns | Computer generated random sequence, stratified by age and dependency on travel by external telephone randomisation, which we assume is consealed. Some gender differences (46% vs 62% men) that happens about 1 in 20 due to chance, and for getting out of the house (28% vs 39&) that happens around 1 out of 9 due to chance. Assumed to probably not be due to chance. |
| Bias due to deviations from intended interventions | 2.1.Were participants aware of their assigned intervention during the trial? | | | Y | Participants and personnel were aware of the assigned intervention. |
|  | 2.2.Were carers and people delivering the interventions aware of participants' assigned intervention during the trial? | | | Y |  |
|  | 2.3. If Y/PY/NI to 2.1 or 2.2: Were there deviations from the intended intervention that arose because of the experimental context? | | | PY | Authors does not analyse or report any specific deviations from the two interventions, but there is a large drop-out from the control intervention (26%) compared to intervention group (12%) indicating a difference in behavior between the two groups due to being in the trial and knowing the allocation of groups. |
|  | 2.4 If Y/PY to 2.3: Were these deviations likely to have affected the outcome? | | | PN | Since the aim of the intervention is to improve outdoor mobility, and the outcome is outdoor mobility, we could assume that the deviations would not have a substancial effect on the outcomes. There is also some attention control as the control group gets one visit and some information about mobility. |
|  | 2.5. If Y/PY/NI to 2.4: Were these deviations from intended intervention balanced between groups? | | | NA |  |
|  | 2.6 Was an appropriate analysis used to estimate the effect of assignment to intervention? | | | PY | Modified intention to treat-analysis, imputing values for missing outcomes, is used and would be considered to be an appropriate analysis. |
|  | 2.7 If N/PN/NI to 2.6: Was there potential for a substantial impact (on the result) of the failure to analyse participants in the group to which they were randomized? | | | NA |  |
|  | Risk of bias judgement | | | Some concerns | Participants and personnel were aware of the assigned intervention. Authors does not analyse or report any specific deviations from the two interventions, but there is a large drop-out from the control intervention (26%) compared to intervention group (12%) indicating a difference in behavior between the two groups due to being in the trial and knowing the allocation of groups.  Since the aim of the intervention is to improve outdoor mobility, and the outcome is outdoor mobility, we could assume that the deviations would not have a substancial effect on the outcomes. There is also some attention control as the control group gets one visit and some information about mobility, limiting the risk of deviations to do other interventions for the control group somewhat. Modified intention to treat-analysis is used, imputing values for missing outcomes, is used and would be considered to be an appropriate analysis. |
| Bias due to missing outcome data | 3.1 Were data for this outcome available for all, or nearly all, participants randomized? | | | PN | Control group has a 10% drop out at 4 months and 25% at 10 months and intervention group has 2% and 12% respectively |
|  | 3.2 If N/PN/NI to 3.1: Is there evidence that result was not biased by missing outcome data? | | | PN | Authors impute values based on previous measurement points (e.g. follow up at 4 month or baseline), which only mitigates any missing outcome data concerns to a small extent. |
|  | 3.3 If N/PN to 3.2: Could missingness in the outcome depend on its true value? | | | Y | Authors do not state any reasons for drop-outs in the groups, but the differences are large between groups. Missing data could influence the outcome and chance the results. |
|  | 3.4 If Y/PY/NI to 3.3: Is it likely that missingness in the outcome depended on its true value? | | | PY |  |
|  | Risk of bias judgement | | | High | Control group has a 10% drop out at 4 months and 25% at 10 months and intervention group has 2% and 12% respectively Authors impute values based on previous measurement points (e.g. follow up at 4 month or baseline), which only mitigates any missing outcome data concerns to a small extent. Authors do not state any reasons for drop-outs in the groups, but the differences are large between groups. Missing data could influence the outcome and chance the results. |
| Bias in measurement of the outcome | 4.1 Was the method of measuring the outcome inappropriate? | | | PN | Self-reported measures of mobility are common and possibly reliable and valid, although there are more appropriate objective methods to measure this outcome by. |
|  | 4.2 Could measurement or ascertainment of the outcome have differed between intervention groups? | | | PN | Measured by post, but we are not told how many in each group had to be contacted by the study administrators to clarify outcomes that were not reported or correctly reported. |
|  | 4.3 Were outcome assessors aware of the intervention received by study participants? | | | Y | The participants rated their own outcomes are were aware of their group allocation. |
|  | 4.4 If Y/PY/NI to 4.3: Could assessment of the outcome have been influenced by knowledge of intervention received? | | | Y | Knowledge of the intervention could influence the self-reported outcome. Since the control intervention contained some time spent to recommend outdoor mobility, the likelyhood of these being substancially different between groups are smaller. |
|  | 4.5 If Y/PY/NI to 4.4: Is it likely that assessment of the outcome was influenced by knowledge of intervention received? | | | PN |  |
|  | Risk of bias judgement | | | Some concerns | Self-reported measurement question of getting outside as much as one would like are possibly as reliable as one this subjective experience can be. Measured by post, but we are not told how many in each group had to be contacted by the study administrators to clarify outcomes that were not reported or correctly reported. The participants rated their own outcomes are were aware of their group allocation.  Knowledge of the intervention could influence the self-reported outcome. Since the control intervention contained some time spent to recommend outdoor mobility, the likelyhood of these being substancially different between groups are smaller. |
| Bias in selection of the reported result | 5.1 Were the data that produced this result analysed in accordance with a pre-specified analysis plan that was finalized before unblinded outcome data were available for analysis? | | | NI | No mention of a protocol and no protocol found after a search. |
|  | 5.2 ... multiple eligible outcome measurements (e.g. scales, definitions, time points) within the outcome domain? | | | PN | Outcomes seem to be transparently reported. |
|  | 5.3 ... multiple eligible analyses of the data? | | | PN | Using analyses to adjust for baseline differences that seem pre-specified, so probably done according to a plan set before looking at the actual baseline data. |
|  | Risk of bias judgement | | | Some concerns | No mention of a protocol and no protocol found after a search. |
| Overall bias | Risk of bias judgement | | | High | Overall high risk of bias due to high risk of bias due to missing outcome data, and some concerns due to non-blinding of participants, outcome assessors, and no available protocol. |
|  |  |  |  |  |  |
|  |  |  |  |  |  |
| Unique ID | Logan 2004 Engagement in everyday life activities 7 months | Study ID | Logan 2004 | Assessor | MR |
| Ref or Label |  | Aim | assignment to intervention (the 'intention-to-treat' effect) |  |  |
| Experimental |  | Comparator |  | Source | Journal article(s) |
| Outcome | Engagement in erveryday life activities 7 months | Results |  | Weight | 1 |
| Domain | Signalling question | | | Response | Comments |
| Bias arising from the randomization process | 1.1 Was the allocation sequence random? | | | PY | Computer generated random sequence, stratified by age and dependency on travel by external telephone randomisation, which we assume is consealed. |
|  | 1.2 Was the allocation sequence concealed until participants were enrolled and assigned to interventions? | | | NI |  |
|  | 1.3 Did baseline differences between intervention groups suggest a problem with the randomization process? | | | PN | Some gender differences (46% vs 62% men) that happens about 1 in 20 due to chance, and for getting out of the house (28% vs 39&) that happens around 1 out of 9 due to chance. Assumed to probably be due to chance. |
|  | Risk of bias judgement | | | Some concerns | Computer generated random sequence, stratified by age and dependency on travel by external telephone randomisation, which we assume is consealed. Some gender differences (46% vs 62% men) that happens about 1 in 20 due to chance, and for getting out of the house (28% vs 39&) that happens around 1 out of 9 due to chance. Assumed to probably not be due to chance. |
| Bias due to deviations from intended interventions | 2.1.Were participants aware of their assigned intervention during the trial? | | | Y | Participants and personnel were aware of the assigned intervention. |
|  | 2.2.Were carers and people delivering the interventions aware of participants' assigned intervention during the trial? | | | Y |  |
|  | 2.3. If Y/PY/NI to 2.1 or 2.2: Were there deviations from the intended intervention that arose because of the experimental context? | | | PY | Authors does not analyse or report any specific deviations from the two interventions, but there is a large drop-out from the control intervention (26%) compared to intervention group (12%) indicating a difference in behavior between the two groups due to being in the trial and knowing the allocation of groups. |
|  | 2.4 If Y/PY to 2.3: Were these deviations likely to have affected the outcome? | | | PN | Since the aim of the intervention is to improve outdoor mobility, and the outcome is outdoor mobility, we could assume that the deviations would not have a substancial effect on the outcomes. There is also some attention control as the control group gets one visit and some information about mobility. |
|  | 2.5. If Y/PY/NI to 2.4: Were these deviations from intended intervention balanced between groups? | | | NA |  |
|  | 2.6 Was an appropriate analysis used to estimate the effect of assignment to intervention? | | | PY | Modified intention to treat-analysis, imputing values for missing outcomes, is used and would be considered to be an appropriate analysis. |
|  | 2.7 If N/PN/NI to 2.6: Was there potential for a substantial impact (on the result) of the failure to analyse participants in the group to which they were randomized? | | | NA |  |
|  | Risk of bias judgement | | | Some concerns | Participants and personnel were aware of the assigned intervention. Authors does not analyse or report any specific deviations from the two interventions, but there is a large drop-out from the control intervention (26%) compared to intervention group (12%) indicating a difference in behavior between the two groups due to being in the trial and knowing the allocation of groups.  Since the aim of the intervention is to improve outdoor mobility, and the outcome is outdoor mobility, we could assume that the deviations would not have a substancial effect on the outcomes. There is also some attention control as the control group gets one visit and some information about mobility, limiting the risk of deviations to do other interventions for the control group somewhat. Modified intention to treat-analysis is used, imputing values for missing outcomes, is used and would be considered to be an appropriate analysis. |
| Bias due to missing outcome data | 3.1 Were data for this outcome available for all, or nearly all, participants randomized? | | | PN | Control group has a 10% drop out at 4 months and 25% at 10 months and intervention group has 2% and 12% respectively |
|  | 3.2 If N/PN/NI to 3.1: Is there evidence that result was not biased by missing outcome data? | | | PN | Authors impute values based on previous measurement points (e.g. follow up at 4 month or baseline), which only mitigates any missing outcome data concerns to a small extent. |
|  | 3.3 If N/PN to 3.2: Could missingness in the outcome depend on its true value? | | | Y | Authors do not state any reasons for drop-outs in the groups, but the differences are large between groups. Missing data could influence the outcome and chance the results. |
|  | 3.4 If Y/PY/NI to 3.3: Is it likely that missingness in the outcome depended on its true value? | | | PY |  |
|  | Risk of bias judgement | | | High | Control group has a 10% drop out at 4 months and 25% at 10 months and intervention group has 2% and 12% respectively Authors impute values based on previous measurement points (e.g. follow up at 4 month or baseline), which only mitigates any missing outcome data concerns to a small extent. Authors do not state any reasons for drop-outs in the groups, but the differences are large between groups. Missing data could influence the outcome and chance the results. |
| Bias in measurement of the outcome | 4.1 Was the method of measuring the outcome inappropriate? | | | PN | Self-reported measures of mobility are common and possibly reliable and valid, although there are more appropriate objective methods to measure this outcome by. |
|  | 4.2 Could measurement or ascertainment of the outcome have differed between intervention groups? | | | PN | Measured by post, but we are not told how many in each group had to be contacted by the study administrators to clarify outcomes that were not reported or correctly reported. |
|  | 4.3 Were outcome assessors aware of the intervention received by study participants? | | | Y | The participants rated their own outcomes are were aware of their group allocation. |
|  | 4.4 If Y/PY/NI to 4.3: Could assessment of the outcome have been influenced by knowledge of intervention received? | | | Y | Knowledge of the intervention could influence the self-reported outcome. Since the control intervention contained some time spent to recommend outdoor mobility, the likelyhood of these being substancially different between groups are smaller. |
|  | 4.5 If Y/PY/NI to 4.4: Is it likely that assessment of the outcome was influenced by knowledge of intervention received? | | | PN |  |
|  | Risk of bias judgement | | | Some concerns | Self-reported measurement question of getting outside as much as one would like are possibly as reliable as one this subjective experience can be. Measured by post, but we are not told how many in each group had to be contacted by the study administrators to clarify outcomes that were not reported or correctly reported. The participants rated their own outcomes are were aware of their group allocation.  Knowledge of the intervention could influence the self-reported outcome. Since the control intervention contained some time spent to recommend outdoor mobility, the likelyhood of these being substancially different between groups are smaller. |
| Bias in selection of the reported result | 5.1 Were the data that produced this result analysed in accordance with a pre-specified analysis plan that was finalized before unblinded outcome data were available for analysis? | | | NI | No mention of a protocol and no protocol found after a search. |
|  | 5.2 ... multiple eligible outcome measurements (e.g. scales, definitions, time points) within the outcome domain? | | | PN | Outcomes seem to be transparently reported. |
|  | 5.3 ... multiple eligible analyses of the data? | | | PN | Using analyses to adjust for baseline differences that seem pre-specified, so probably done according to a plan set before looking at the actual baseline data. |
|  | Risk of bias judgement | | | Some concerns | No mention of a protocol and no protocol found after a search. |
| Overall bias | Risk of bias judgement | | | High | Overall high risk of bias due to high risk of bias due to missing outcome data, and some concerns due to non-blinding of participants, outcome assessors, and no available protocol. |
|  |  |  |  |  |  |
|  |  |  |  |  |  |
| Unique ID | Logan 2004 Participation 6 months | Study ID | Logan 2004 | Assessor | MR |
| Ref or Label |  | Aim | assignment to intervention (the 'intention-to-treat' effect) |  |  |
| Experimental |  | Comparator |  | Source | Journal article(s) |
| Outcome | Participation 6 months | Results |  | Weight | 1 |
| Domain | Signalling question | | | Response | Comments |
| Bias arising from the randomization process | 1.1 Was the allocation sequence random? | | | PY | Computer generated random sequence, stratified by age and dependency on travel by external telephone randomisation, which we assume is consealed. |
|  | 1.2 Was the allocation sequence concealed until participants were enrolled and assigned to interventions? | | | NI |  |
|  | 1.3 Did baseline differences between intervention groups suggest a problem with the randomization process? | | | PN | Some gender differences (46% vs 62% men) that happens about 1 in 20 due to chance, and for getting out of the house (28% vs 39&) that happens around 1 out of 9 due to chance. Assumed to probably be due to chance. |
|  | Risk of bias judgement | | | Some concerns | Computer generated random sequence, stratified by age and dependency on travel by external telephone randomisation, which we assume is consealed. Some gender differences (46% vs 62% men) that happens about 1 in 20 due to chance, and for getting out of the house (28% vs 39&) that happens around 1 out of 9 due to chance. Assumed to probably not be due to chance. |
| Bias due to deviations from intended interventions | 2.1.Were participants aware of their assigned intervention during the trial? | | | Y | Participants and personnel were aware of the assigned intervention. |
|  | 2.2.Were carers and people delivering the interventions aware of participants' assigned intervention during the trial? | | | Y |  |
|  | 2.3. If Y/PY/NI to 2.1 or 2.2: Were there deviations from the intended intervention that arose because of the experimental context? | | | PY | Authors does not analyse or report any specific deviations from the two interventions, but there is a large drop-out from the control intervention (26%) compared to intervention group (12%) indicating a difference in behavior between the two groups due to being in the trial and knowing the allocation of groups. |
|  | 2.4 If Y/PY to 2.3: Were these deviations likely to have affected the outcome? | | | PN | Since the aim of the intervention is to improve outdoor mobility, and the outcome is outdoor mobility, we could assume that the deviations would not have a substancial effect on the outcomes. There is also some attention control as the control group gets one visit and some information about mobility. |
|  | 2.5. If Y/PY/NI to 2.4: Were these deviations from intended intervention balanced between groups? | | | NA |  |
|  | 2.6 Was an appropriate analysis used to estimate the effect of assignment to intervention? | | | PY | Modified intention to treat-analysis, imputing values for missing outcomes, is used and would be considered to be an appropriate analysis. |
|  | 2.7 If N/PN/NI to 2.6: Was there potential for a substantial impact (on the result) of the failure to analyse participants in the group to which they were randomized? | | | NA |  |
|  | Risk of bias judgement | | | Some concerns | Participants and personnel were aware of the assigned intervention. Authors does not analyse or report any specific deviations from the two interventions, but there is a large drop-out from the control intervention (26%) compared to intervention group (12%) indicating a difference in behavior between the two groups due to being in the trial and knowing the allocation of groups.  Since the aim of the intervention is to improve outdoor mobility, and the outcome is outdoor mobility, we could assume that the deviations would not have a substancial effect on the outcomes. There is also some attention control as the control group gets one visit and some information about mobility, limiting the risk of deviations to do other interventions for the control group somewhat. Modified intention to treat-analysis is used, imputing values for missing outcomes, is used and would be considered to be an appropriate analysis. |
| Bias due to missing outcome data | 3.1 Were data for this outcome available for all, or nearly all, participants randomized? | | | PN | Control group has a 10% drop out at 4 months and 25% at 10 months and intervention group has 2% and 12% respectively |
|  | 3.2 If N/PN/NI to 3.1: Is there evidence that result was not biased by missing outcome data? | | | PN | Authors impute values based on previous measurement points (e.g. follow up at 4 month or baseline), which only mitigates any missing outcome data concerns to a small extent. |
|  | 3.3 If N/PN to 3.2: Could missingness in the outcome depend on its true value? | | | Y | Authors do not state any reasons for drop-outs in the groups, but the differences are large between groups. Missing data could influence the outcome and chance the results. |
|  | 3.4 If Y/PY/NI to 3.3: Is it likely that missingness in the outcome depended on its true value? | | | PY |  |
|  | Risk of bias judgement | | | High | Control group has a 10% drop out at 4 months and 25% at 10 months and intervention group has 2% and 12% respectively Authors impute values based on previous measurement points (e.g. follow up at 4 month or baseline), which only mitigates any missing outcome data concerns to a small extent. Authors do not state any reasons for drop-outs in the groups, but the differences are large between groups. Missing data could influence the outcome and chance the results. |
| Bias in measurement of the outcome | 4.1 Was the method of measuring the outcome inappropriate? | | | PN | Self-reported measures of mobility are common and possibly reliable and valid, although there are more appropriate objective methods to measure this outcome by. |
|  | 4.2 Could measurement or ascertainment of the outcome have differed between intervention groups? | | | PN | Measured by post, but we are not told how many in each group had to be contacted by the study administrators to clarify outcomes that were not reported or correctly reported. |
|  | 4.3 Were outcome assessors aware of the intervention received by study participants? | | | Y | The participants rated their own outcomes are were aware of their group allocation. |
|  | 4.4 If Y/PY/NI to 4.3: Could assessment of the outcome have been influenced by knowledge of intervention received? | | | Y | Knowledge of the intervention could influence the self-reported outcome. Since the control intervention contained some time spent to recommend outdoor mobility, the likelyhood of these being substancially different between groups are smaller. |
|  | 4.5 If Y/PY/NI to 4.4: Is it likely that assessment of the outcome was influenced by knowledge of intervention received? | | | PN |  |
|  | Risk of bias judgement | | | Some concerns | Self-reported measurement question of getting outside as much as one would like are possibly as reliable as one this subjective experience can be. Measured by post, but we are not told how many in each group had to be contacted by the study administrators to clarify outcomes that were not reported or correctly reported. The participants rated their own outcomes are were aware of their group allocation.  Knowledge of the intervention could influence the self-reported outcome. Since the control intervention contained some time spent to recommend outdoor mobility, the likelyhood of these being substancially different between groups are smaller. |
| Bias in selection of the reported result | 5.1 Were the data that produced this result analysed in accordance with a pre-specified analysis plan that was finalized before unblinded outcome data were available for analysis? | | | NI | No mention of a protocol and no protocol found after a search. |
|  | 5.2 ... multiple eligible outcome measurements (e.g. scales, definitions, time points) within the outcome domain? | | | PN | Outcomes seem to be transparently reported. |
|  | 5.3 ... multiple eligible analyses of the data? | | | PN | Using analyses to adjust for baseline differences that seem pre-specified, so probably done according to a plan set before looking at the actual baseline data. |
|  | Risk of bias judgement | | | Some concerns | No mention of a protocol and no protocol found after a search. |
| Overall bias | Risk of bias judgement | | | High | Overall high risk of bias due to high risk of bias due to missing outcome data, and some concerns due to non-blinding of participants, outcome assessors, and no available protocol. |
|  |  |  |  |  |  |
|  |  |  |  |  |  |
| Unique ID | Logan 2004 Participation 7 months | Study ID | Logan 2004 | Assessor | MR |
| Ref or Label |  | Aim | assignment to intervention (the 'intention-to-treat' effect) |  |  |
| Experimental |  | Comparator |  | Source | Journal article(s) |
| Outcome | Participation 7 months | Results |  | Weight | 1 |
| Domain | Signalling question | | | Response | Comments |
| Bias arising from the randomization process | 1.1 Was the allocation sequence random? | | | PY | Computer generated random sequence, stratified by age and dependency on travel by external telephone randomisation, which we assume is consealed. |
|  | 1.2 Was the allocation sequence concealed until participants were enrolled and assigned to interventions? | | | NI |  |
|  | 1.3 Did baseline differences between intervention groups suggest a problem with the randomization process? | | | PN | Some gender differences (46% vs 62% men) that happens about 1 in 20 due to chance, and for getting out of the house (28% vs 39&) that happens around 1 out of 9 due to chance. Assumed to probably be due to chance. |
|  | Risk of bias judgement | | | Some concerns | Computer generated random sequence, stratified by age and dependency on travel by external telephone randomisation, which we assume is consealed. Some gender differences (46% vs 62% men) that happens about 1 in 20 due to chance, and for getting out of the house (28% vs 39&) that happens around 1 out of 9 due to chance. Assumed to probably be due to chance. |
| Bias due to deviations from intended interventions | 2.1.Were participants aware of their assigned intervention during the trial? | | | Y | Participants and personnel were aware of the assigned intervention. |
|  | 2.2.Were carers and people delivering the interventions aware of participants' assigned intervention during the trial? | | | Y |  |
|  | 2.3. If Y/PY/NI to 2.1 or 2.2: Were there deviations from the intended intervention that arose because of the experimental context? | | | PY | Authors does not analyse or report any specific deviations from the two interventions, but there is a large drop-out from the control intervention (26%) compared to intervention group (12%) indicating a difference in behavior between the two groups due to being in the trial and knowing the allocation of groups. |
|  | 2.4 If Y/PY to 2.3: Were these deviations likely to have affected the outcome? | | | PN | Since the aim of the intervention is to improve outdoor mobility, and the outcome is outdoor mobility, we could assume that the deviations would not have a substancial effect on the outcomes. There is also some attention control as the control group gets one visit and some information about mobility. |
|  | 2.5. If Y/PY/NI to 2.4: Were these deviations from intended intervention balanced between groups? | | | NA |  |
|  | 2.6 Was an appropriate analysis used to estimate the effect of assignment to intervention? | | | PY | Modified intention to treat-analysis, imputing values for missing outcomes, is used and would be considered to be an appropriate analysis. |
|  | 2.7 If N/PN/NI to 2.6: Was there potential for a substantial impact (on the result) of the failure to analyse participants in the group to which they were randomized? | | | NA |  |
|  | Risk of bias judgement | | | Some concerns | Participants and personnel were aware of the assigned intervention. Authors does not analyse or report any specific deviations from the two interventions, but there is a large drop-out from the control intervention (26%) compared to intervention group (12%) indicating a difference in behavior between the two groups due to being in the trial and knowing the allocation of groups.  Since the aim of the intervention is to improve outdoor mobility, and the outcome is outdoor mobility, we could assume that the deviations would not have a substancial effect on the outcomes. There is also some attention control as the control group gets one visit and some information about mobility, limiting the risk of deviations to do other interventions for the control group somewhat. Modified intention to treat-analysis is used, imputing values for missing outcomes, is used and would be considered to be an appropriate analysis. |
| Bias due to missing outcome data | 3.1 Were data for this outcome available for all, or nearly all, participants randomized? | | | PN | Control group has a 10% drop out at 4 months and 25% at 10 months and intervention group has 2% and 12% respectively |
|  | 3.2 If N/PN/NI to 3.1: Is there evidence that result was not biased by missing outcome data? | | | PN | Authors impute values based on previous measurement points (e.g. follow up at 4 month or baseline), which only mitigates any missing outcome data concerns to a small extent. |
|  | 3.3 If N/PN to 3.2: Could missingness in the outcome depend on its true value? | | | Y | Authors do not state any reasons for drop-outs in the groups, but the differences are large between groups. Missing data could influence the outcome and chance the results. |
|  | 3.4 If Y/PY/NI to 3.3: Is it likely that missingness in the outcome depended on its true value? | | | PY |  |
|  | Risk of bias judgement | | | High | Control group has a 10% drop out at 4 months and 25% at 10 months and intervention group has 2% and 12% respectively Authors impute values based on previous measurement points (e.g. follow up at 4 month or baseline), which only mitigates any missing outcome data concerns to a small extent. Authors do not state any reasons for drop-outs in the groups, but the differences are large between groups. Missing data could influence the outcome and chance the results. |
| Bias in measurement of the outcome | 4.1 Was the method of measuring the outcome inappropriate? | | | PN | Self-reported measures of mobility are common and possibly reliable and valid, although there are more appropriate objective methods to measure this outcome by. |
|  | 4.2 Could measurement or ascertainment of the outcome have differed between intervention groups? | | | PN | Measured by post, but we are not told how many in each group had to be contacted by the study administrators to clarify outcomes that were not reported or correctly reported. |
|  | 4.3 Were outcome assessors aware of the intervention received by study participants? | | | Y | The participants rated their own outcomes are were aware of their group allocation. |
|  | 4.4 If Y/PY/NI to 4.3: Could assessment of the outcome have been influenced by knowledge of intervention received? | | | Y | Knowledge of the intervention could influence the self-reported outcome. Since the control intervention contained some time spent to recommend outdoor mobility, the likelyhood of these being substancially different between groups are smaller. |
|  | 4.5 If Y/PY/NI to 4.4: Is it likely that assessment of the outcome was influenced by knowledge of intervention received? | | | PN |  |
|  | Risk of bias judgement | | | Some concerns | Self-reported measurement question of getting outside as much as one would like are possibly as reliable as one this subjective experience can be. Measured by post, but we are not told how many in each group had to be contacted by the study administrators to clarify outcomes that were not reported or correctly reported. The participants rated their own outcomes are were aware of their group allocation.  Knowledge of the intervention could influence the self-reported outcome. Since the control intervention contained some time spent to recommend outdoor mobility, the likelyhood of these being substancially different between groups are smaller. |
| Bias in selection of the reported result | 5.1 Were the data that produced this result analysed in accordance with a pre-specified analysis plan that was finalized before unblinded outcome data were available for analysis? | | | NI | No mention of a protocol and no protocol found after a search. |
|  | 5.2 ... multiple eligible outcome measurements (e.g. scales, definitions, time points) within the outcome domain? | | | PN | Outcomes seem to be transparently reported. |
|  | 5.3 ... multiple eligible analyses of the data? | | | PN | Using analyses to adjust for baseline differences that seem pre-specified, so probably done according to a plan set before looking at the actual baseline data. |
|  | Risk of bias judgement | | | Some concerns | No mention of a protocol and no protocol found after a search. |
| Overall bias | Risk of bias judgement | | | High | Overall high risk of bias due to high risk of bias due to missing outcome data, and some concerns due to non-blinding of participants, outcome assessors, and no available protocol. |
|  |  |  |  |  |  |
|  |  |  |  |  |  |
| Unique ID | Logan 2014 Activity outside the home 6 months | Study ID | Logan 2014 | Assessor | MR |
| Ref or Label |  | Aim | assignment to intervention (the 'intention-to-treat' effect) |  |  |
| Experimental |  | Comparator |  | Source | Journal article(s) |
| Outcome | Activity outside the home 6 months | Results |  | Weight | 1 |
| Domain | Signalling question | | | Response | Comments |
| Bias arising from the randomization process | 1.1 Was the allocation sequence random? | | | PY | Web-based randomisation in varying blocks and stratified by site and age.  The randomisation was done by the local therapist meeting the participant after baseline values was collected and after the control intervention had been delivered. After entering baseline values the therapist got information about the allocation of the participant. |
|  | 1.2 Was the allocation sequence concealed until participants were enrolled and assigned to interventions? | | | PY |  |
|  | 1.3 Did baseline differences between intervention groups suggest a problem with the randomization process? | | | PY | Some baseline differences is present with worse prognostic factors for the intervention group. QoL is 45.9 vs 50.1, which would happen due to changce 1 in 15, NEADL 8.8 vs 10.1 (measure of basline activity levels) which would happen due to chance around 1 in 95, and time since stroke 43 months vs 37 months which would happen around 1 in 8. |
|  | Risk of bias judgement | | | Some concerns | Web-based randomisation in varying blocks and stratified by site and age.  The randomisation was done by the local therapist meeting the participant after baseline values was collected and after the control intervention had been delivered. After entering baseline values the therapist got information about the allocation of the participant.  Some baseline differences is present with worse prognostic factors for the intervention group. QoL is 45.9 vs 50.1, which would happen due to changce 1 in 15, NEADL 8.8 vs 10.1 (measure of basline activity levels) which would happen due to chance around 1 in 95, and time since stroke 43 months vs 37 months which would happen around 1 in 8. It is unclear why these baseline-inbalances are present for some prognostic variables, hence some conserns of risk of bias is present as they could potentially influence the outcomes down the line. |
| Bias due to deviations from intended interventions | 2.1.Were participants aware of their assigned intervention during the trial? | | | Y | Both participants and treatment providers were aware of the assigned interventions. |
|  | 2.2.Were carers and people delivering the interventions aware of participants' assigned intervention during the trial? | | | Y |  |
|  | 2.3. If Y/PY/NI to 2.1 or 2.2: Were there deviations from the intended intervention that arose because of the experimental context? | | | PY | No information provided about deviations. There is a likelyhood of non-protocol intervention being used, but since the control group got some attention control to improve outdoor mobility the risk is less. |
|  | 2.4 If Y/PY to 2.3: Were these deviations likely to have affected the outcome? | | | PN | Control group got attention, limiting the potential of deviations somewhat. |
|  | 2.5. If Y/PY/NI to 2.4: Were these deviations from intended intervention balanced between groups? | | | NA |  |
|  | 2.6 Was an appropriate analysis used to estimate the effect of assignment to intervention? | | | Y | ITT was usped |
|  | 2.7 If N/PN/NI to 2.6: Was there potential for a substantial impact (on the result) of the failure to analyse participants in the group to which they were randomized? | | | NA |  |
|  | Risk of bias judgement | | | Some concerns | Both participants and treatment providers were aware of the assigned interventions.  No information provided about deviations. There is a likelyhood of non-protocol intervention being used, but since the control group got some attention control to improve outdoor mobility the risk would be less. |
| Bias due to missing outcome data | 3.1 Were data for this outcome available for all, or nearly all, participants randomized? | | | N | No tracking of diaries for travel. Only 55.1% retured travel diares during the full study period. |
|  | 3.2 If N/PN/NI to 3.1: Is there evidence that result was not biased by missing outcome data? | | | PN | 73.5% of outcome data was collected from the intervention group and 67.5 was collected from the control group (9% more in intervention group). No evidence to reduce any bias bu this missing outcome data. |
|  | 3.3 If N/PN to 3.2: Could missingness in the outcome depend on its true value? | | | PY | Yes, it is likely that people not going outside (e.g. more disability) and being in the control group (and not getting attention) would leave the study and/or not send in their travel diaries and this could influence the outcomes. |
|  | 3.4 If Y/PY/NI to 3.3: Is it likely that missingness in the outcome depended on its true value? | | | PY |  |
|  | Risk of bias judgement | | | High | No tracking of diaries for travel. Only 55.1% retured travel diares during the full study period.  73.5% of outcome data was collected from the intervention group and 67.5 was collected from the control group (9% more in intervention group). No evidence to reduce any bias bu this missing outcome data. Yes, it is likely that people not going outside (e.g. more disability) and being in the control group (and not getting attention) would leave the study and/or not send in their travel diaries and this could influence the outcomes. |
| Bias in measurement of the outcome | 4.1 Was the method of measuring the outcome inappropriate? | | | PN | There are other and more reliable ways to measure outdoor mobility, but would not be sufficiently inappropriate to warrant a lower rating of bias at this stage. |
|  | 4.2 Could measurement or ascertainment of the outcome have differed between intervention groups? | | | PY | Control condition did not send in diaries as often, and most likely had more incomplete diaries, than the intervention. Hence, the collection of measurements were different between the groups. In addition, the participants were aware of their assignment, and that would likely influence the way partipants rated their outcome. |
|  | 4.3 Were outcome assessors aware of the intervention received by study participants? | | | NA |  |
|  | 4.4 If Y/PY/NI to 4.3: Could assessment of the outcome have been influenced by knowledge of intervention received? | | | NA |  |
|  | 4.5 If Y/PY/NI to 4.4: Is it likely that assessment of the outcome was influenced by knowledge of intervention received? | | | NA |  |
|  | Risk of bias judgement | | | High | There are other and more reliable ways to measure outdoor mobility, but would not be sufficiently inappropriate to warrant a lower rating of bias at this stage.  Control condition did not send in diaries as often, and most likely had more incomplete diaries, than the intervention. Hence, the collection of measurements were different between the groups. In addition, the participants were aware of their assignment, and that would likely influence the way partipants rated their outcome. |
| Bias in selection of the reported result | 5.1 Were the data that produced this result analysed in accordance with a pre-specified analysis plan that was finalized before unblinded outcome data were available for analysis? | | | Y | Protocol and analysis plan was published prospectively. |
|  | 5.2 ... multiple eligible outcome measurements (e.g. scales, definitions, time points) within the outcome domain? | | | N | All outcomes are reported as planned. |
|  | 5.3 ... multiple eligible analyses of the data? | | | N | All analyses are reported as planned, and also included unadjusted values. |
|  | Risk of bias judgement | | | Low | Protocol and analysis plan was published prospectively. All outcomes are reported as planned. All analyses are reported as planned, and also included unadjusted values. |
| Overall bias | Risk of bias judgement | | | High | Overall high risk of bias due to likelyhood of unblinded participants to behave differently in both groups, of missing outcome data and how outcomes was measured. |
|  |  |  |  |  |  |
|  |  |  |  |  |  |
| Unique ID | Logan 2014 Activity outside the home 7 months | Study ID | Logan 2014 | Assessor | MR |
| Ref or Label |  | Aim | assignment to intervention (the 'intention-to-treat' effect) |  |  |
| Experimental |  | Comparator |  | Source | Journal article(s) |
| Outcome | Activity outside the home 7 months | Results |  | Weight | 1 |
| Domain | Signalling question | | | Response | Comments |
| Bias arising from the randomization process | 1.1 Was the allocation sequence random? | | | PY | Web-based randomisation in varying blocks and stratified by site and age.  The randomisation was done by the local therapist meeting the participant after baseline values was collected and after the control intervention had been delivered. After entering baseline values the therapist got information about the allocation of the participant. |
|  | 1.2 Was the allocation sequence concealed until participants were enrolled and assigned to interventions? | | | PY |  |
|  | 1.3 Did baseline differences between intervention groups suggest a problem with the randomization process? | | | PY | Some baseline differences is present with worse prognostic factors for the intervention group. QoL is 45.9 vs 50.1, which would happen due to changce 1 in 15, NEADL 8.8 vs 10.1 (measure of basline activity levels) which would happen due to chance around 1 in 95, and time since stroke 43 months vs 37 months which would happen around 1 in 8. |
|  | Risk of bias judgement | | | Some concerns | Web-based randomisation in varying blocks and stratified by site and age.  The randomisation was done by the local therapist meeting the participant after baseline values was collected and after the control intervention had been delivered. After entering baseline values the therapist got information about the allocation of the participant.  Some baseline differences is present with worse prognostic factors for the intervention group. QoL is 45.9 vs 50.1, which would happen due to changce 1 in 15, NEADL 8.8 vs 10.1 (measure of basline activity levels) which would happen due to chance around 1 in 95, and time since stroke 43 months vs 37 months which would happen around 1 in 8. It is unclear why these baseline-inbalances are present for some prognostic variables, hence some conserns of risk of bias is present as they could potentially influence the outcomes down the line. |
| Bias due to deviations from intended interventions | 2.1.Were participants aware of their assigned intervention during the trial? | | | Y | Both participants and treatment providers were aware of the assigned interventions. |
|  | 2.2.Were carers and people delivering the interventions aware of participants' assigned intervention during the trial? | | | Y |  |
|  | 2.3. If Y/PY/NI to 2.1 or 2.2: Were there deviations from the intended intervention that arose because of the experimental context? | | | NI | No information provided about deviations. There is a likelyhood of non-protocol intervention being used, but since the control group got some attention control to improve outdoor mobility the risk is less. |
|  | 2.4 If Y/PY to 2.3: Were these deviations likely to have affected the outcome? | | | NA |  |
|  | 2.5. If Y/PY/NI to 2.4: Were these deviations from intended intervention balanced between groups? | | | NA |  |
|  | 2.6 Was an appropriate analysis used to estimate the effect of assignment to intervention? | | | Y |  |
|  | 2.7 If N/PN/NI to 2.6: Was there potential for a substantial impact (on the result) of the failure to analyse participants in the group to which they were randomized? | | | NA |  |
|  | Risk of bias judgement | | | Some concerns | Both participants and treatment providers were aware of the assigned interventions.  No information provided about deviations. There is a likelyhood of non-protocol intervention being used, but since the control group got some attention control to improve outdoor mobility the risk would be less. |
| Bias due to missing outcome data | 3.1 Were data for this outcome available for all, or nearly all, participants randomized? | | | N | No tracking of diaries for travel. Only 55.1% retured travel diares during the full study period. |
|  | 3.2 If N/PN/NI to 3.1: Is there evidence that result was not biased by missing outcome data? | | | PN | 73.5% of outcome data was collected from the intervention group and 67.5 was collected from the control group (9% more in intervention group). No evidence to reduce any bias bu this missing outcome data. |
|  | 3.3 If N/PN to 3.2: Could missingness in the outcome depend on its true value? | | | PY | Yes, it is likely that people not going outside (e.g. more disability) and being in the control group (and not getting attention) would leave the study and/or not send in their travel diaries and this could influence the outcomes. |
|  | 3.4 If Y/PY/NI to 3.3: Is it likely that missingness in the outcome depended on its true value? | | | PY |  |
|  | Risk of bias judgement | | | High | No tracking of diaries for travel. Only 55.1% retured travel diares during the full study period.  73.5% of outcome data was collected from the intervention group and 67.5 was collected from the control group (9% more in intervention group). No evidence to reduce any bias bu this missing outcome data. Yes, it is likely that people not going outside (e.g. more disability) and being in the control group (and not getting attention) would leave the study and/or not send in their travel diaries and this could influence the outcomes. |
| Bias in measurement of the outcome | 4.1 Was the method of measuring the outcome inappropriate? | | | PN | There are other and more reliable ways to measure outdoor mobility, but would not be sufficiently inappropriate to warrant a lower rating of bias at this stage. |
|  | 4.2 Could measurement or ascertainment of the outcome have differed between intervention groups? | | | PY | Control condition did not send in diaries as often, and most likely had more incomplete diaries, than the intervention. Hence, the collection of measurements were different between the groups. In addition, the participants were aware of their assignment, and that would likely influence the way partipants rated their outcome. |
|  | 4.3 Were outcome assessors aware of the intervention received by study participants? | | | NA |  |
|  | 4.4 If Y/PY/NI to 4.3: Could assessment of the outcome have been influenced by knowledge of intervention received? | | | NA |  |
|  | 4.5 If Y/PY/NI to 4.4: Is it likely that assessment of the outcome was influenced by knowledge of intervention received? | | | NA |  |
|  | Risk of bias judgement | | | High | There are other and more reliable ways to measure outdoor mobility, but would not be sufficiently inappropriate to warrant a lower rating of bias at this stage.  Control condition did not send in diaries as often, and most likely had more incomplete diaries, than the intervention. Hence, the collection of measurements were different between the groups. In addition, the participants were aware of their assignment, and that would likely influence the way partipants rated their outcome. |
| Bias in selection of the reported result | 5.1 Were the data that produced this result analysed in accordance with a pre-specified analysis plan that was finalized before unblinded outcome data were available for analysis? | | | Y | Protocol and analysis plan was published prospectively. |
|  | 5.2 ... multiple eligible outcome measurements (e.g. scales, definitions, time points) within the outcome domain? | | | N | All outcomes are reported as planned. |
|  | 5.3 ... multiple eligible analyses of the data? | | | N | All analyses are reported as planned, and also included unadjusted values. |
|  | Risk of bias judgement | | | Low | Protocol and analysis plan was published prospectively. All outcomes are reported as planned. All analyses are reported as planned, and also included unadjusted values. |
| Overall bias | Risk of bias judgement | | | High | Overall high risk of bias due to likelyhood of unblinded participants to behave differently in both groups, of missing outcome data and how outcomes was measured. |
|  |  |  |  |  |  |
|  |  |  |  |  |  |
| Unique ID | Logan 2014 Engagement in erveryday life activities 6 months | Study ID | Logan 2014 | Assessor | MR |
| Ref or Label |  | Aim | assignment to intervention (the 'intention-to-treat' effect) |  |  |
| Experimental |  | Comparator |  | Source | Journal article(s) |
| Outcome | Engagement in erveryday life activities 6 months | Results |  | Weight | 1 |
| Domain | Signalling question | | | Response | Comments |
| Bias arising from the randomization process | 1.1 Was the allocation sequence random? | | | PY | Web-based randomisation in varying blocks and stratified by site and age.  The randomisation was done by the local therapist meeting the participant after baseline values was collected and after the control intervention had been delivered. After entering baseline values the therapist got information about the allocation of the participant. |
|  | 1.2 Was the allocation sequence concealed until participants were enrolled and assigned to interventions? | | | PY |  |
|  | 1.3 Did baseline differences between intervention groups suggest a problem with the randomization process? | | | PY | Some baseline differences is present with worse prognostic factors for the intervention group. QoL is 45.9 vs 50.1, which would happen due to changce 1 in 15, NEADL 8.8 vs 10.1 (measure of basline activity levels) which would happen due to chance around 1 in 95, and time since stroke 43 months vs 37 months which would happen around 1 in 8. |
|  | Risk of bias judgement | | | Some concerns | Web-based randomisation in varying blocks and stratified by site and age.  The randomisation was done by the local therapist meeting the participant after baseline values was collected and after the control intervention had been delivered. After entering baseline values the therapist got information about the allocation of the participant.  Some baseline differences is present with worse prognostic factors for the intervention group. QoL is 45.9 vs 50.1, which would happen due to changce 1 in 15, NEADL 8.8 vs 10.1 (measure of basline activity levels) which would happen due to chance around 1 in 95, and time since stroke 43 months vs 37 months which would happen around 1 in 8. It is unclear why these baseline-inbalances are present for some prognostic variables, hence some conserns of risk of bias is present as they could potentially influence the outcomes down the line. |
| Bias due to deviations from intended interventions | 2.1.Were participants aware of their assigned intervention during the trial? | | | Y | Both participants and treatment providers were aware of the assigned interventions. |
|  | 2.2.Were carers and people delivering the interventions aware of participants' assigned intervention during the trial? | | | Y |  |
|  | 2.3. If Y/PY/NI to 2.1 or 2.2: Were there deviations from the intended intervention that arose because of the experimental context? | | | NI | No information provided about deviations. There is a likelyhood of non-protocol intervention being used, but since the control group got some attention control to improve outdoor mobility the risk is less. |
|  | 2.4 If Y/PY to 2.3: Were these deviations likely to have affected the outcome? | | | NA |  |
|  | 2.5. If Y/PY/NI to 2.4: Were these deviations from intended intervention balanced between groups? | | | NA |  |
|  | 2.6 Was an appropriate analysis used to estimate the effect of assignment to intervention? | | | Y |  |
|  | 2.7 If N/PN/NI to 2.6: Was there potential for a substantial impact (on the result) of the failure to analyse participants in the group to which they were randomized? | | | NA |  |
|  | Risk of bias judgement | | | Some concerns | Both participants and treatment providers were aware of the assigned interventions.  No information provided about deviations. There is a likelyhood of non-protocol intervention being used, but since the control group got some attention control to improve outdoor mobility the risk would be less. |
| Bias due to missing outcome data | 3.1 Were data for this outcome available for all, or nearly all, participants randomized? | | | PN | 8% loss to follow up in intervention and 15% in control group at 6 months, mostly becuase more participants withdrew. |
|  | 3.2 If N/PN/NI to 3.1: Is there evidence that result was not biased by missing outcome data? | | | PN | Authors did not report data on who left the study. Results were explored in sensitivity analyses, indicating no major impact of missing data, limiting the risk of bias somewhat. |
|  | 3.3 If N/PN to 3.2: Could missingness in the outcome depend on its true value? | | | PY | Almost double of withdrawn participants in the control group, which could influence the outcomes to some degree at 6 months. |
|  | 3.4 If Y/PY/NI to 3.3: Is it likely that missingness in the outcome depended on its true value? | | | PN |  |
|  | Risk of bias judgement | | | Some concerns | 8% loss to follow up in intervention and 15% in control group at 6 months, mostly becuase more participants withdrew. Authors did not report data on who left the study. Almost double of withdrawn participants in the control group, which could influence the outcomes to some degree at 6 months. |
| Bias in measurement of the outcome | 4.1 Was the method of measuring the outcome inappropriate? | | | PN | Better methods exist, but it would not be considered inappropriate. |
|  | 4.2 Could measurement or ascertainment of the outcome have differed between intervention groups? | | | PN | Probably not. |
|  | 4.3 Were outcome assessors aware of the intervention received by study participants? | | | Y | Participants were aware of their intervention |
|  | 4.4 If Y/PY/NI to 4.3: Could assessment of the outcome have been influenced by knowledge of intervention received? | | | PY | Outcome assessors were not blinded. Since the outcomes were self-reported the measurements could have been different between groups, but since the control group had some attention-control this would be less. |
|  | 4.5 If Y/PY/NI to 4.4: Is it likely that assessment of the outcome was influenced by knowledge of intervention received? | | | PN |  |
|  | Risk of bias judgement | | | Some concerns | Outcome assessors were not blinded. Since the outcomes were self-reported the measurements could have been different between groups, but since the control group had some attention-control this would be less. |
| Bias in selection of the reported result | 5.1 Were the data that produced this result analysed in accordance with a pre-specified analysis plan that was finalized before unblinded outcome data were available for analysis? | | | Y | Protocol and analysis plan was published prospectively. |
|  | 5.2 ... multiple eligible outcome measurements (e.g. scales, definitions, time points) within the outcome domain? | | | N | All outcomes are reported as planned. |
|  | 5.3 ... multiple eligible analyses of the data? | | | N | All analyses are reported as planned, and also included unadjusted values. |
|  | Risk of bias judgement | | | Low | Protocol and analysis plan was published prospectively. All outcomes are reported as planned. All analyses are reported as planned, and also included unadjusted values. |
| Overall bias | Risk of bias judgement | | | High | Overall high risk of bias due to high risk of bias in the measurement of the outcome due to participants rating their own participation levels, and some concers in the randomisation process due to unclear and large baseline differences, no description of methods for allocation consealment and some concerns for deviations from intended interventions due to having an inactive no intervention-comparison group. |
|  |  |  |  |  |  |
|  |  |  |  |  |  |
| Unique ID | Logan 2014 Engagement in erveryday life activities 7 months | Study ID | Logan 2014 | Assessor | MR |
| Ref or Label |  | Aim | assignment to intervention (the 'intention-to-treat' effect) |  |  |
| Experimental |  | Comparator |  | Source | Journal article(s) |
| Outcome | Engagement in erveryday life activities 7 months | Results |  | Weight | 1 |
| Domain | Signalling question | | | Response | Comments |
| Bias arising from the randomization process | 1.1 Was the allocation sequence random? | | | PY | Web-based randomisation in varying blocks and stratified by site and age.  The randomisation was done by the local therapist meeting the participant after baseline values was collected and after the control intervention had been delivered. After entering baseline values the therapist got information about the allocation of the participant. |
|  | 1.2 Was the allocation sequence concealed until participants were enrolled and assigned to interventions? | | | PY |  |
|  | 1.3 Did baseline differences between intervention groups suggest a problem with the randomization process? | | | PY | Some baseline differences is present with worse prognostic factors for the intervention group. QoL is 45.9 vs 50.1, which would happen due to changce 1 in 15, NEADL 8.8 vs 10.1 (measure of basline activity levels) which would happen due to chance around 1 in 95, and time since stroke 43 months vs 37 months which would happen around 1 in 8. |
|  | Risk of bias judgement | | | Some concerns | Web-based randomisation in varying blocks and stratified by site and age.  The randomisation was done by the local therapist meeting the participant after baseline values was collected and after the control intervention had been delivered. After entering baseline values the therapist got information about the allocation of the participant.  Some baseline differences is present with worse prognostic factors for the intervention group. QoL is 45.9 vs 50.1, which would happen due to changce 1 in 15, NEADL 8.8 vs 10.1 (measure of basline activity levels) which would happen due to chance around 1 in 95, and time since stroke 43 months vs 37 months which would happen around 1 in 8. It is unclear why these baseline-inbalances are present for some prognostic variables, hence some conserns of risk of bias is present as they could potentially influence the outcomes down the line. |
| Bias due to deviations from intended interventions | 2.1.Were participants aware of their assigned intervention during the trial? | | | Y | Both participants and treatment providers were aware of the assigned interventions. |
|  | 2.2.Were carers and people delivering the interventions aware of participants' assigned intervention during the trial? | | | Y |  |
|  | 2.3. If Y/PY/NI to 2.1 or 2.2: Were there deviations from the intended intervention that arose because of the experimental context? | | | NI | No information provided about deviations. There is a likelyhood of non-protocol intervention being used, but since the control group got some attention control to improve outdoor mobility the risk is less. |
|  | 2.4 If Y/PY to 2.3: Were these deviations likely to have affected the outcome? | | | NA |  |
|  | 2.5. If Y/PY/NI to 2.4: Were these deviations from intended intervention balanced between groups? | | | NA |  |
|  | 2.6 Was an appropriate analysis used to estimate the effect of assignment to intervention? | | | Y |  |
|  | 2.7 If N/PN/NI to 2.6: Was there potential for a substantial impact (on the result) of the failure to analyse participants in the group to which they were randomized? | | | NA |  |
|  | Risk of bias judgement | | | Some concerns | Both participants and treatment providers were aware of the assigned interventions.  No information provided about deviations. There is a likelyhood of non-protocol intervention being used, but since the control group got some attention control to improve outdoor mobility the risk would be less. |
| Bias due to missing outcome data | 3.1 Were data for this outcome available for all, or nearly all, participants randomized? | | | N | 19% loss to follow up in the intervention group and 25% missing in the control group at 12 months. |
|  | 3.2 If N/PN/NI to 3.1: Is there evidence that result was not biased by missing outcome data? | | | N |  |
|  | 3.3 If N/PN to 3.2: Could missingness in the outcome depend on its true value? | | | PY | Yes, more people withdrew from the trial in the control group, and the missing data in both groups are substancial and could influence the outcomes down the line. |
|  | 3.4 If Y/PY/NI to 3.3: Is it likely that missingness in the outcome depended on its true value? | | | PY |  |
|  | Risk of bias judgement | | | High | 19% loss to follow up in the intervention group and 25% missing in the control group at 12 months. Yes, more people withdrew from the trial in the control group, and the missing data in both groups are substancial and could influence the outcomes down the line. |
| Bias in measurement of the outcome | 4.1 Was the method of measuring the outcome inappropriate? | | | PN | Better methods exist, but it would not be considered inappropriate. |
|  | 4.2 Could measurement or ascertainment of the outcome have differed between intervention groups? | | | PN | Probably not. |
|  | 4.3 Were outcome assessors aware of the intervention received by study participants? | | | Y | Participants were aware of their intervention |
|  | 4.4 If Y/PY/NI to 4.3: Could assessment of the outcome have been influenced by knowledge of intervention received? | | | PY | Outcome assessors were not blinded. Since the outcomes were self-reported the measurements could have been different between groups, but since the control group had some attention-control this would be less. |
|  | 4.5 If Y/PY/NI to 4.4: Is it likely that assessment of the outcome was influenced by knowledge of intervention received? | | | PN |  |
|  | Risk of bias judgement | | | Some concerns | Outcome assessors were not blinded. Since the outcomes were self-reported the measurements could have been different between groups, but since the control group had some attention-control this would be less. |
| Bias in selection of the reported result | 5.1 Were the data that produced this result analysed in accordance with a pre-specified analysis plan that was finalized before unblinded outcome data were available for analysis? | | | Y | Protocol and analysis plan was published prospectively. |
|  | 5.2 ... multiple eligible outcome measurements (e.g. scales, definitions, time points) within the outcome domain? | | | N | All outcomes are reported as planned. |
|  | 5.3 ... multiple eligible analyses of the data? | | | N | All analyses are reported as planned, and also included unadjusted values. |
|  | Risk of bias judgement | | | Low | Protocol and analysis plan was published prospectively. All outcomes are reported as planned. All analyses are reported as planned, and also included unadjusted values. |
| Overall bias | Risk of bias judgement | | | High | Overall high risk of bias due to high risk of bias in the measurement of the outcome due to participants rating their own participation levels, and some concers in the randomisation process due to unclear and large baseline differences, no description of methods for allocation consealment and some concerns for deviations from intended interventions due to having an inactive no intervention-comparison group. |
|  |  |  |  |  |  |
|  |  |  |  |  |  |
| Unique ID | Logan 2014 Health-related quality of life 6 months | Study ID | Logan 2014 | Assessor | MR |
| Ref or Label |  | Aim | assignment to intervention (the 'intention-to-treat' effect) |  |  |
| Experimental |  | Comparator |  | Source | Journal article(s) |
| Outcome | Health-related quality of life 6 months | Results |  | Weight | 1 |
| Domain | Signalling question | | | Response | Comments |
| Bias arising from the randomization process | 1.1 Was the allocation sequence random? | | | PY | Web-based randomisation in varying blocks and stratified by site and age.  The randomisation was done by the local therapist meeting the participant after baseline values was collected and after the control intervention had been delivered. After entering baseline values the therapist got information about the allocation of the participant. |
|  | 1.2 Was the allocation sequence concealed until participants were enrolled and assigned to interventions? | | | PY |  |
|  | 1.3 Did baseline differences between intervention groups suggest a problem with the randomization process? | | | PY | Some baseline differences is present with worse prognostic factors for the intervention group. QoL is 45.9 vs 50.1, which would happen due to changce 1 in 15, NEADL 8.8 vs 10.1 (measure of basline activity levels) which would happen due to chance around 1 in 95, and time since stroke 43 months vs 37 months which would happen around 1 in 8. |
|  | Risk of bias judgement | | | Some concerns | Web-based randomisation in varying blocks and stratified by site and age.  The randomisation was done by the local therapist meeting the participant after baseline values was collected and after the control intervention had been delivered. After entering baseline values the therapist got information about the allocation of the participant.  Some baseline differences is present with worse prognostic factors for the intervention group. QoL is 45.9 vs 50.1, which would happen due to changce 1 in 15, NEADL 8.8 vs 10.1 (measure of basline activity levels) which would happen due to chance around 1 in 95, and time since stroke 43 months vs 37 months which would happen around 1 in 8. It is unclear why these baseline-inbalances are present for some prognostic variables, hence some conserns of risk of bias is present as they could potentially influence the outcomes down the line. |
| Bias due to deviations from intended interventions | 2.1.Were participants aware of their assigned intervention during the trial? | | | Y | Both participants and treatment providers were aware of the assigned interventions. |
|  | 2.2.Were carers and people delivering the interventions aware of participants' assigned intervention during the trial? | | | Y |  |
|  | 2.3. If Y/PY/NI to 2.1 or 2.2: Were there deviations from the intended intervention that arose because of the experimental context? | | | NI | No information provided about deviations. There is a likelyhood of non-protocol intervention being used, but since the control group got some attention control to improve outdoor mobility the risk is less. |
|  | 2.4 If Y/PY to 2.3: Were these deviations likely to have affected the outcome? | | | NA |  |
|  | 2.5. If Y/PY/NI to 2.4: Were these deviations from intended intervention balanced between groups? | | | NA |  |
|  | 2.6 Was an appropriate analysis used to estimate the effect of assignment to intervention? | | | Y | ITT was used |
|  | 2.7 If N/PN/NI to 2.6: Was there potential for a substantial impact (on the result) of the failure to analyse participants in the group to which they were randomized? | | | NA |  |
|  | Risk of bias judgement | | | Some concerns | Both participants and treatment providers were aware of the assigned interventions.  No information provided about deviations. There is a likelyhood of non-protocol intervention being used, but since the control group got some attention control to improve outdoor mobility the risk would be less. |
| Bias due to missing outcome data | 3.1 Were data for this outcome available for all, or nearly all, participants randomized? | | | PN | 8% loss to follow up in intervention and 15% in control group at 6 months, mostly becuase more participants withdrew. |
|  | 3.2 If N/PN/NI to 3.1: Is there evidence that result was not biased by missing outcome data? | | | N | Authors did not report data on who left the study. |
|  | 3.3 If N/PN to 3.2: Could missingness in the outcome depend on its true value? | | | PY | Almost double of withdrawn participants in the control group, which could influence the outcomes to some degree at 6 months. |
|  | 3.4 If Y/PY/NI to 3.3: Is it likely that missingness in the outcome depended on its true value? | | | PY |  |
|  | Risk of bias judgement | | | High | 8% loss to follow up in intervention and 15% in control group at 6 months, mostly becuase more participants withdrew. Authors did not report data on who left the study. Almost double of withdrawn participants in the control group, which could influence the outcomes to some degree at 6 months. |
| Bias in measurement of the outcome | 4.1 Was the method of measuring the outcome inappropriate? | | | PN | Better methods exist, but it would not be considered inappropriate. |
|  | 4.2 Could measurement or ascertainment of the outcome have differed between intervention groups? | | | PN | Probably not. |
|  | 4.3 Were outcome assessors aware of the intervention received by study participants? | | | Y | Participants were aware of their intervention |
|  | 4.4 If Y/PY/NI to 4.3: Could assessment of the outcome have been influenced by knowledge of intervention received? | | | PY | Outcome assessors were not blinded. Since the outcomes were self-reported the measurements could have been different between groups, but since the control group had some attention-control this would be less. |
|  | 4.5 If Y/PY/NI to 4.4: Is it likely that assessment of the outcome was influenced by knowledge of intervention received? | | | PN |  |
|  | Risk of bias judgement | | | Some concerns | Outcome assessors were not blinded. Since the outcomes were self-reported the measurements could have been different between groups, but since the control group had some attention-control this would be less. |
| Bias in selection of the reported result | 5.1 Were the data that produced this result analysed in accordance with a pre-specified analysis plan that was finalized before unblinded outcome data were available for analysis? | | | Y | Protocol and analysis plan was published prospectively. |
|  | 5.2 ... multiple eligible outcome measurements (e.g. scales, definitions, time points) within the outcome domain? | | | N | All outcomes are reported as planned. |
|  | 5.3 ... multiple eligible analyses of the data? | | | N | All analyses are reported as planned, and also included unadjusted values. |
|  | Risk of bias judgement | | | Low | Protocol and analysis plan was published prospectively. All outcomes are reported as planned. All analyses are reported as planned, and also included unadjusted values. |
| Overall bias | Risk of bias judgement | | | High | Overall high risk of bias due to high risk of bias due to missing outcome data, and some concerns due to non-blinding of participants, outcome assessors, and no available protocol. |
|  |  |  |  |  |  |
|  |  |  |  |  |  |
| Unique ID | Logan 2014 Health-related quality of life 7 months | Study ID | Logan 2014 | Assessor | MR |
| Ref or Label |  | Aim | assignment to intervention (the 'intention-to-treat' effect) |  |  |
| Experimental |  | Comparator |  | Source | Journal article(s) |
| Outcome | Health-related quality of life 7 months | Results |  | Weight | 1 |
| Domain | Signalling question | | | Response | Comments |
| Bias arising from the randomization process | 1.1 Was the allocation sequence random? | | | PY | Web-based randomisation in varying blocks and stratified by site and age.  The randomisation was done by the local therapist meeting the participant after baseline values was collected and after the control intervention had been delivered. After entering baseline values the therapist got information about the allocation of the participant. |
|  | 1.2 Was the allocation sequence concealed until participants were enrolled and assigned to interventions? | | | PY |  |
|  | 1.3 Did baseline differences between intervention groups suggest a problem with the randomization process? | | | PY | Some baseline differences is present with worse prognostic factors for the intervention group. QoL is 45.9 vs 50.1, which would happen due to changce 1 in 15, NEADL 8.8 vs 10.1 (measure of basline activity levels) which would happen due to chance around 1 in 95, and time since stroke 43 months vs 37 months which would happen around 1 in 8. |
|  | Risk of bias judgement | | | Some concerns | Web-based randomisation in varying blocks and stratified by site and age.  The randomisation was done by the local therapist meeting the participant after baseline values was collected and after the control intervention had been delivered. After entering baseline values the therapist got information about the allocation of the participant.  Some baseline differences is present with worse prognostic factors for the intervention group. QoL is 45.9 vs 50.1, which would happen due to changce 1 in 15, NEADL 8.8 vs 10.1 (measure of basline activity levels) which would happen due to chance around 1 in 95, and time since stroke 43 months vs 37 months which would happen around 1 in 8. It is unclear why these baseline-inbalances are present for some prognostic variables, hence some conserns of risk of bias is present as they could potentially influence the outcomes down the line. |
| Bias due to deviations from intended interventions | 2.1.Were participants aware of their assigned intervention during the trial? | | | Y | Both participants and treatment providers were aware of the assigned interventions. |
|  | 2.2.Were carers and people delivering the interventions aware of participants' assigned intervention during the trial? | | | Y |  |
|  | 2.3. If Y/PY/NI to 2.1 or 2.2: Were there deviations from the intended intervention that arose because of the experimental context? | | | NI | No information provided about deviations. There is a likelyhood of non-protocol intervention being used, but since the control group got some attention control to improve outdoor mobility the risk is less. |
|  | 2.4 If Y/PY to 2.3: Were these deviations likely to have affected the outcome? | | | NA |  |
|  | 2.5. If Y/PY/NI to 2.4: Were these deviations from intended intervention balanced between groups? | | | NA |  |
|  | 2.6 Was an appropriate analysis used to estimate the effect of assignment to intervention? | | | Y |  |
|  | 2.7 If N/PN/NI to 2.6: Was there potential for a substantial impact (on the result) of the failure to analyse participants in the group to which they were randomized? | | | NA |  |
|  | Risk of bias judgement | | | Some concerns | Both participants and treatment providers were aware of the assigned interventions.  No information provided about deviations. There is a likelyhood of non-protocol intervention being used, but since the control group got some attention control to improve outdoor mobility the risk would be less. |
| Bias due to missing outcome data | 3.1 Were data for this outcome available for all, or nearly all, participants randomized? | | |  | 19% loss to follow up in the intervention group and 25% missing in the control group at 12 months. |
|  | 3.2 If N/PN/NI to 3.1: Is there evidence that result was not biased by missing outcome data? | | | N |  |
|  | 3.3 If N/PN to 3.2: Could missingness in the outcome depend on its true value? | | | PY | Yes, more people withdrew from the trial in the control group, and the missing data in both groups are substancial and could influence the outcomes down the line. |
|  | 3.4 If Y/PY/NI to 3.3: Is it likely that missingness in the outcome depended on its true value? | | | PY |  |
|  | Risk of bias judgement | | | High | 19% loss to follow up in the intervention group and 25% missing in the control group at 12 months. Yes, more people withdrew from the trial in the control group, and the missing data in both groups are substancial and could influence the outcomes down the line. |
| Bias in measurement of the outcome | 4.1 Was the method of measuring the outcome inappropriate? | | | PN | Better methods exist, but it would not be considered inappropriate. |
|  | 4.2 Could measurement or ascertainment of the outcome have differed between intervention groups? | | | PN | Probably not. |
|  | 4.3 Were outcome assessors aware of the intervention received by study participants? | | | Y | Participants were aware of their intervention |
|  | 4.4 If Y/PY/NI to 4.3: Could assessment of the outcome have been influenced by knowledge of intervention received? | | | PY | Outcome assessors were not blinded. Since the outcomes were self-reported the measurements could have been different between groups, but since the control group had some attention-control this would be less. |
|  | 4.5 If Y/PY/NI to 4.4: Is it likely that assessment of the outcome was influenced by knowledge of intervention received? | | | PN |  |
|  | Risk of bias judgement | | | Some concerns | Outcome assessors were not blinded. Since the outcomes were self-reported the measurements could have been different between groups, but since the control group had some attention-control this would be less. |
| Bias in selection of the reported result | 5.1 Were the data that produced this result analysed in accordance with a pre-specified analysis plan that was finalized before unblinded outcome data were available for analysis? | | | Y | Protocol and analysis plan was published prospectively. |
|  | 5.2 ... multiple eligible outcome measurements (e.g. scales, definitions, time points) within the outcome domain? | | | N | All outcomes are reported as planned. |
|  | 5.3 ... multiple eligible analyses of the data? | | | N | All analyses are reported as planned, and also included unadjusted values. |
|  | Risk of bias judgement | | | Low | Protocol and analysis plan was published prospectively. All outcomes are reported as planned. All analyses are reported as planned, and also included unadjusted values. |
| Overall bias | Risk of bias judgement | | | High | Overall high risk of bias due to high risk of bias due to missing outcome data, and some concerns due to non-blinding of participants, outcome assessors, and no available protocol. |
|  |  |  |  |  |  |
|  |  |  |  |  |  |
| Unique ID | Logan 2014 Major harms 6 months | Study ID | Logan 2014 | Assessor | MR |
| Ref or Label |  | Aim | assignment to intervention (the 'intention-to-treat' effect) |  |  |
| Experimental |  | Comparator |  | Source | Journal article(s) |
| Outcome | Major harms 6 months | Results |  | Weight | 1 |
| Domain | Signalling question | | | Response | Comments |
| Bias arising from the randomization process | 1.1 Was the allocation sequence random? | | | PY | Web-based randomisation in varying blocks and stratified by site and age.  The randomisation was done by the local therapist meeting the participant after baseline values was collected and after the control intervention had been delivered. After entering baseline values the therapist got information about the allocation of the participant. |
|  | 1.2 Was the allocation sequence concealed until participants were enrolled and assigned to interventions? | | | PY |  |
|  | 1.3 Did baseline differences between intervention groups suggest a problem with the randomization process? | | | PY | Some baseline differences is present with worse prognostic factors for the intervention group. QoL is 45.9 vs 50.1, which would happen due to changce 1 in 15, NEADL 8.8 vs 10.1 (measure of basline activity levels) which would happen due to chance around 1 in 95, and time since stroke 43 months vs 37 months which would happen around 1 in 8. |
|  | Risk of bias judgement | | | Some concerns | Web-based randomisation in varying blocks and stratified by site and age.  The randomisation was done by the local therapist meeting the participant after baseline values was collected and after the control intervention had been delivered. After entering baseline values the therapist got information about the allocation of the participant.  Some baseline differences is present with worse prognostic factors for the intervention group. QoL is 45.9 vs 50.1, which would happen due to changce 1 in 15, NEADL 8.8 vs 10.1 (measure of basline activity levels) which would happen due to chance around 1 in 95, and time since stroke 43 months vs 37 months which would happen around 1 in 8. It is unclear why these baseline-inbalances are present for some prognostic variables, hence some conserns of risk of bias is present as they could potentially influence the outcomes down the line. |
| Bias due to deviations from intended interventions | 2.1.Were participants aware of their assigned intervention during the trial? | | | Y | Both participants and treatment providers were aware of the assigned interventions. |
|  | 2.2.Were carers and people delivering the interventions aware of participants' assigned intervention during the trial? | | | Y |  |
|  | 2.3. If Y/PY/NI to 2.1 or 2.2: Were there deviations from the intended intervention that arose because of the experimental context? | | | NI | No information provided about deviations. There is a likelyhood of non-protocol intervention being used, but since the control group got some attention control to improve outdoor mobility the risk is less. |
|  | 2.4 If Y/PY to 2.3: Were these deviations likely to have affected the outcome? | | | NA |  |
|  | 2.5. If Y/PY/NI to 2.4: Were these deviations from intended intervention balanced between groups? | | | NA |  |
|  | 2.6 Was an appropriate analysis used to estimate the effect of assignment to intervention? | | | Y |  |
|  | 2.7 If N/PN/NI to 2.6: Was there potential for a substantial impact (on the result) of the failure to analyse participants in the group to which they were randomized? | | | NA |  |
|  | Risk of bias judgement | | | Some concerns | Both participants and treatment providers were aware of the assigned interventions.  No information provided about deviations. There is a likelyhood of non-protocol intervention being used, but since the control group got some attention control to improve outdoor mobility the risk would be less. |
| Bias due to missing outcome data | 3.1 Were data for this outcome available for all, or nearly all, participants randomized? | | | PY | Mortality data collected from comprehensive health registries, also for people who withdrew. |
|  | 3.2 If N/PN/NI to 3.1: Is there evidence that result was not biased by missing outcome data? | | | NA |  |
|  | 3.3 If N/PN to 3.2: Could missingness in the outcome depend on its true value? | | | NA |  |
|  | 3.4 If Y/PY/NI to 3.3: Is it likely that missingness in the outcome depended on its true value? | | | NA |  |
|  | Risk of bias judgement | | | Low | Mortality data collected from comprehensive health registries, also for people who withdrew. |
| Bias in measurement of the outcome | 4.1 Was the method of measuring the outcome inappropriate? | | | N | No |
|  | 4.2 Could measurement or ascertainment of the outcome have differed between intervention groups? | | | N |  |
|  | 4.3 Were outcome assessors aware of the intervention received by study participants? | | | N | Blinded measurements of electronic mortality data |
|  | 4.4 If Y/PY/NI to 4.3: Could assessment of the outcome have been influenced by knowledge of intervention received? | | | NA |  |
|  | 4.5 If Y/PY/NI to 4.4: Is it likely that assessment of the outcome was influenced by knowledge of intervention received? | | | NA |  |
|  | Risk of bias judgement | | | Low | Blinded measurements of electronic mortality data |
| Bias in selection of the reported result | 5.1 Were the data that produced this result analysed in accordance with a pre-specified analysis plan that was finalized before unblinded outcome data were available for analysis? | | | Y | Protocol and analysis plan was published prospectively. |
|  | 5.2 ... multiple eligible outcome measurements (e.g. scales, definitions, time points) within the outcome domain? | | | N | All outcomes are reported as planned. |
|  | 5.3 ... multiple eligible analyses of the data? | | | N | All analyses are reported as planned, and also included unadjusted values. |
|  | Risk of bias judgement | | | Low | Protocol and analysis plan was published prospectively. All outcomes are reported as planned. All analyses are reported as planned, and also included unadjusted values. |
| Overall bias | Risk of bias judgement | | | Some concerns | Overall some concerns for risk of bias due to potential differences at baseline between groups as well as non-blinding of interventions. |
|  |  |  |  |  |  |
|  |  |  |  |  |  |
| Unique ID | Logan 2014 Major harms 7 months | Study ID | Logan 2014 | Assessor | MR |
| Ref or Label |  | Aim | assignment to intervention (the 'intention-to-treat' effect) |  |  |
| Experimental |  | Comparator |  | Source | Journal article(s) |
| Outcome | Major harms 7 months | Results |  | Weight | 1 |
| Domain | Signalling question | | | Response | Comments |
| Bias arising from the randomization process | 1.1 Was the allocation sequence random? | | | PY | Web-based randomisation in varying blocks and stratified by site and age.  The randomisation was done by the local therapist meeting the participant after baseline values was collected and after the control intervention had been delivered. After entering baseline values the therapist got information about the allocation of the participant. |
|  | 1.2 Was the allocation sequence concealed until participants were enrolled and assigned to interventions? | | | PY |  |
|  | 1.3 Did baseline differences between intervention groups suggest a problem with the randomization process? | | | PY | Some baseline differences is present with worse prognostic factors for the intervention group. QoL is 45.9 vs 50.1, which would happen due to changce 1 in 15, NEADL 8.8 vs 10.1 (measure of basline activity levels) which would happen due to chance around 1 in 95, and time since stroke 43 months vs 37 months which would happen around 1 in 8. |
|  | Risk of bias judgement | | | Some concerns | Web-based randomisation in varying blocks and stratified by site and age.  The randomisation was done by the local therapist meeting the participant after baseline values was collected and after the control intervention had been delivered. After entering baseline values the therapist got information about the allocation of the participant.  Some baseline differences is present with worse prognostic factors for the intervention group. QoL is 45.9 vs 50.1, which would happen due to changce 1 in 15, NEADL 8.8 vs 10.1 (measure of basline activity levels) which would happen due to chance around 1 in 95, and time since stroke 43 months vs 37 months which would happen around 1 in 8. It is unclear why these baseline-inbalances are present for some prognostic variables, hence some conserns of risk of bias is present as they could potentially influence the outcomes down the line. |
| Bias due to deviations from intended interventions | 2.1.Were participants aware of their assigned intervention during the trial? | | | Y | Both participants and treatment providers were aware of the assigned interventions. |
|  | 2.2.Were carers and people delivering the interventions aware of participants' assigned intervention during the trial? | | | Y |  |
|  | 2.3. If Y/PY/NI to 2.1 or 2.2: Were there deviations from the intended intervention that arose because of the experimental context? | | | NI | No information provided about deviations. There is a likelyhood of non-protocol intervention being used, but since the control group got some attention control to improve outdoor mobility the risk is less. |
|  | 2.4 If Y/PY to 2.3: Were these deviations likely to have affected the outcome? | | | NA |  |
|  | 2.5. If Y/PY/NI to 2.4: Were these deviations from intended intervention balanced between groups? | | | NA |  |
|  | 2.6 Was an appropriate analysis used to estimate the effect of assignment to intervention? | | | Y |  |
|  | 2.7 If N/PN/NI to 2.6: Was there potential for a substantial impact (on the result) of the failure to analyse participants in the group to which they were randomized? | | | NA |  |
|  | Risk of bias judgement | | | Some concerns | Both participants and treatment providers were aware of the assigned interventions.  No information provided about deviations. There is a likelyhood of non-protocol intervention being used, but since the control group got some attention control to improve outdoor mobility the risk would be less. |
| Bias due to missing outcome data | 3.1 Were data for this outcome available for all, or nearly all, participants randomized? | | | Y | Mortality data collected from comprehensive health registries, also for people who withdrew. |
|  | 3.2 If N/PN/NI to 3.1: Is there evidence that result was not biased by missing outcome data? | | | NA |  |
|  | 3.3 If N/PN to 3.2: Could missingness in the outcome depend on its true value? | | | NA |  |
|  | 3.4 If Y/PY/NI to 3.3: Is it likely that missingness in the outcome depended on its true value? | | | NA |  |
|  | Risk of bias judgement | | | Low | Mortality data collected from comprehensive health registries, also for people who withdrew. |
| Bias in measurement of the outcome | 4.1 Was the method of measuring the outcome inappropriate? | | | N | No |
|  | 4.2 Could measurement or ascertainment of the outcome have differed between intervention groups? | | | N |  |
|  | 4.3 Were outcome assessors aware of the intervention received by study participants? | | | N | Blinded measurements of electronic mortality data |
|  | 4.4 If Y/PY/NI to 4.3: Could assessment of the outcome have been influenced by knowledge of intervention received? | | | NA |  |
|  | 4.5 If Y/PY/NI to 4.4: Is it likely that assessment of the outcome was influenced by knowledge of intervention received? | | | NA |  |
|  | Risk of bias judgement | | | Low | Blinded measurements of electronic mortality data |
| Bias in selection of the reported result | 5.1 Were the data that produced this result analysed in accordance with a pre-specified analysis plan that was finalized before unblinded outcome data were available for analysis? | | | Y | Protocol and analysis plan was published prospectively. |
|  | 5.2 ... multiple eligible outcome measurements (e.g. scales, definitions, time points) within the outcome domain? | | | N | All outcomes are reported as planned. |
|  | 5.3 ... multiple eligible analyses of the data? | | | N | All analyses are reported as planned, and also included unadjusted values. |
|  | Risk of bias judgement | | | Low | Protocol and analysis plan was published prospectively. All outcomes are reported as planned. All analyses are reported as planned, and also included unadjusted values. |
| Overall bias | Risk of bias judgement | | | Some concerns | Overall some conserns due to baseline differences as well as non-blinding of participants and treatment providers. |
|  |  |  |  |  |  |
|  |  |  |  |  |  |
| Unique ID | Logan 2014 Minor harms 6 months | Study ID | Logan 2014 | Assessor | MR |
| Ref or Label |  | Aim | assignment to intervention (the 'intention-to-treat' effect) |  |  |
| Experimental |  | Comparator |  | Source | Journal article(s) |
| Outcome | Minor adverse events 6 months | Results |  | Weight | 1 |
| Domain | Signalling question | | | Response | Comments |
| Bias arising from the randomization process | 1.1 Was the allocation sequence random? | | | PY | Web-based randomisation in varying blocks and stratified by site and age.  The randomisation was done by the local therapist meeting the participant after baseline values was collected and after the control intervention had been delivered. After entering baseline values the therapist got information about the allocation of the participant. |
|  | 1.2 Was the allocation sequence concealed until participants were enrolled and assigned to interventions? | | | PY |  |
|  | 1.3 Did baseline differences between intervention groups suggest a problem with the randomization process? | | | PY | Some baseline differences is present with worse prognostic factors for the intervention group. QoL is 45.9 vs 50.1, which would happen due to changce 1 in 15, NEADL 8.8 vs 10.1 (measure of basline activity levels) which would happen due to chance around 1 in 95, and time since stroke 43 months vs 37 months which would happen around 1 in 8. |
|  | Risk of bias judgement | | | Some concerns | Web-based randomisation in varying blocks and stratified by site and age.  The randomisation was done by the local therapist meeting the participant after baseline values was collected and after the control intervention had been delivered. After entering baseline values the therapist got information about the allocation of the participant.  Some baseline differences is present with worse prognostic factors for the intervention group. QoL is 45.9 vs 50.1, which would happen due to changce 1 in 15, NEADL 8.8 vs 10.1 (measure of basline activity levels) which would happen due to chance around 1 in 95, and time since stroke 43 months vs 37 months which would happen around 1 in 8. It is unclear why these baseline-inbalances are present for some prognostic variables, hence some conserns of risk of bias is present as they could potentially influence the outcomes down the line. |
| Bias due to deviations from intended interventions | 2.1.Were participants aware of their assigned intervention during the trial? | | | Y | Both participants and treatment providers were aware of the assigned interventions. |
|  | 2.2.Were carers and people delivering the interventions aware of participants' assigned intervention during the trial? | | | Y |  |
|  | 2.3. If Y/PY/NI to 2.1 or 2.2: Were there deviations from the intended intervention that arose because of the experimental context? | | | NI | No information provided about deviations. There is a likelyhood of non-protocol intervention being used, but since the control group got some attention control to improve outdoor mobility the risk is less. |
|  | 2.4 If Y/PY to 2.3: Were these deviations likely to have affected the outcome? | | | NA |  |
|  | 2.5. If Y/PY/NI to 2.4: Were these deviations from intended intervention balanced between groups? | | | NA |  |
|  | 2.6 Was an appropriate analysis used to estimate the effect of assignment to intervention? | | | Y |  |
|  | 2.7 If N/PN/NI to 2.6: Was there potential for a substantial impact (on the result) of the failure to analyse participants in the group to which they were randomized? | | | NA |  |
|  | Risk of bias judgement | | | Some concerns | Both participants and treatment providers were aware of the assigned interventions.  No information provided about deviations. There is a likelyhood of non-protocol intervention being used, but since the control group got some attention control to improve outdoor mobility the risk would be less. |
| Bias due to missing outcome data | 3.1 Were data for this outcome available for all, or nearly all, participants randomized? | | | PN | 8% loss to follow up in intervention and 15% in control group at 6 months, mostly becuase more participants withdrew. |
|  | 3.2 If N/PN/NI to 3.1: Is there evidence that result was not biased by missing outcome data? | | | N | Authors did not report data on who left the study. |
|  | 3.3 If N/PN to 3.2: Could missingness in the outcome depend on its true value? | | | PY | Almost double of withdrawn participants in the control group, which could influence the outcomes to some degree at 6 months. |
|  | 3.4 If Y/PY/NI to 3.3: Is it likely that missingness in the outcome depended on its true value? | | | PY |  |
|  | Risk of bias judgement | | | High | 8% loss to follow up in intervention and 15% in control group at 6 months, mostly becuase more participants withdrew. Authors did not report data on who left the study. Almost double of withdrawn participants in the control group, which could influence the outcomes to some degree at 6 months. |
| Bias in measurement of the outcome | 4.1 Was the method of measuring the outcome inappropriate? | | | PN | Better methods exist, but it would not be considered inappropriate. |
|  | 4.2 Could measurement or ascertainment of the outcome have differed between intervention groups? | | | PN | Probably not. |
|  | 4.3 Were outcome assessors aware of the intervention received by study participants? | | | Y | Participants were aware of their intervention |
|  | 4.4 If Y/PY/NI to 4.3: Could assessment of the outcome have been influenced by knowledge of intervention received? | | | PY | Outcome assessors were not blinded. Since the outcomes were self-reported the measurements could have been different between groups, but since the control group had some attention-control this would be less. |
|  | 4.5 If Y/PY/NI to 4.4: Is it likely that assessment of the outcome was influenced by knowledge of intervention received? | | | PN |  |
|  | Risk of bias judgement | | | Some concerns | Outcome assessors were not blinded. Since the outcomes were self-reported the measurements could have been different between groups, but since the control group had some attention-control this would be less. |
| Bias in selection of the reported result | 5.1 Were the data that produced this result analysed in accordance with a pre-specified analysis plan that was finalized before unblinded outcome data were available for analysis? | | | Y | Protocol and analysis plan was published prospectively. |
|  | 5.2 ... multiple eligible outcome measurements (e.g. scales, definitions, time points) within the outcome domain? | | | N | All outcomes are reported as planned. |
|  | 5.3 ... multiple eligible analyses of the data? | | | N | All analyses are reported as planned, and also included unadjusted values. |
|  | Risk of bias judgement | | | Low | Protocol and analysis plan was published prospectively. All outcomes are reported as planned. All analyses are reported as planned, and also included unadjusted values. |
| Overall bias | Risk of bias judgement | | | High | Overall high risk of bias due to likelyhood of unblinded participants to behave differently in both groups, of missing outcome data and how outcomes was measured. |
|  |  |  |  |  |  |
|  |  |  |  |  |  |
| Unique ID | Logan 2014 Minor harms 7 months | Study ID | Logan 2014 | Assessor | MR |
| Ref or Label |  | Aim | assignment to intervention (the 'intention-to-treat' effect) |  |  |
| Experimental |  | Comparator |  | Source | Journal article(s) |
| Outcome | Minor adverse events 7 months | Results |  | Weight | 1 |
| Domain | Signalling question | | | Response | Comments |
| Bias arising from the randomization process | 1.1 Was the allocation sequence random? | | | PY | Web-based randomisation in varying blocks and stratified by site and age.  The randomisation was done by the local therapist meeting the participant after baseline values was collected and after the control intervention had been delivered. After entering baseline values the therapist got information about the allocation of the participant. |
|  | 1.2 Was the allocation sequence concealed until participants were enrolled and assigned to interventions? | | | PY |  |
|  | 1.3 Did baseline differences between intervention groups suggest a problem with the randomization process? | | | PY | Some baseline differences is present with worse prognostic factors for the intervention group. QoL is 45.9 vs 50.1, which would happen due to changce 1 in 15, NEADL 8.8 vs 10.1 (measure of basline activity levels) which would happen due to chance around 1 in 95, and time since stroke 43 months vs 37 months which would happen around 1 in 8. |
|  | Risk of bias judgement | | | Some concerns | Web-based randomisation in varying blocks and stratified by site and age.  The randomisation was done by the local therapist meeting the participant after baseline values was collected and after the control intervention had been delivered. After entering baseline values the therapist got information about the allocation of the participant.  Some baseline differences is present with worse prognostic factors for the intervention group. QoL is 45.9 vs 50.1, which would happen due to changce 1 in 15, NEADL 8.8 vs 10.1 (measure of basline activity levels) which would happen due to chance around 1 in 95, and time since stroke 43 months vs 37 months which would happen around 1 in 8. It is unclear why these baseline-inbalances are present for some prognostic variables, hence some conserns of risk of bias is present as they could potentially influence the outcomes down the line. |
| Bias due to deviations from intended interventions | 2.1.Were participants aware of their assigned intervention during the trial? | | | Y | Both participants and treatment providers were aware of the assigned interventions. |
|  | 2.2.Were carers and people delivering the interventions aware of participants' assigned intervention during the trial? | | | Y |  |
|  | 2.3. If Y/PY/NI to 2.1 or 2.2: Were there deviations from the intended intervention that arose because of the experimental context? | | | NI | No information provided about deviations. There is a likelyhood of non-protocol intervention being used, but since the control group got some attention control to improve outdoor mobility the risk is less. |
|  | 2.4 If Y/PY to 2.3: Were these deviations likely to have affected the outcome? | | | NA |  |
|  | 2.5. If Y/PY/NI to 2.4: Were these deviations from intended intervention balanced between groups? | | | NA |  |
|  | 2.6 Was an appropriate analysis used to estimate the effect of assignment to intervention? | | | Y |  |
|  | 2.7 If N/PN/NI to 2.6: Was there potential for a substantial impact (on the result) of the failure to analyse participants in the group to which they were randomized? | | | NA |  |
|  | Risk of bias judgement | | | Some concerns | Both participants and treatment providers were aware of the assigned interventions.  No information provided about deviations. There is a likelyhood of non-protocol intervention being used, but since the control group got some attention control to improve outdoor mobility the risk would be less. |
| Bias due to missing outcome data | 3.1 Were data for this outcome available for all, or nearly all, participants randomized? | | |  | 19% loss to follow up in the intervention group and 25% missing in the control group at 12 months. |
|  | 3.2 If N/PN/NI to 3.1: Is there evidence that result was not biased by missing outcome data? | | | N |  |
|  | 3.3 If N/PN to 3.2: Could missingness in the outcome depend on its true value? | | | PY | Yes, more people withdrew from the trial in the control group, and the missing data in both groups are substancial and could influence the outcomes down the line. |
|  | 3.4 If Y/PY/NI to 3.3: Is it likely that missingness in the outcome depended on its true value? | | | PY |  |
|  | Risk of bias judgement | | | High | 19% loss to follow up in the intervention group and 25% missing in the control group at 12 months. Yes, more people withdrew from the trial in the control group, and the missing data in both groups are substancial and could influence the outcomes down the line. |
| Bias in measurement of the outcome | 4.1 Was the method of measuring the outcome inappropriate? | | | PN | Better methods exist, but it would not be considered inappropriate. |
|  | 4.2 Could measurement or ascertainment of the outcome have differed between intervention groups? | | | PN | Probably not. |
|  | 4.3 Were outcome assessors aware of the intervention received by study participants? | | | Y | Participants were aware of their intervention |
|  | 4.4 If Y/PY/NI to 4.3: Could assessment of the outcome have been influenced by knowledge of intervention received? | | | PY | Outcome assessors were not blinded. Since the outcomes were self-reported the measurements could have been different between groups, but since the control group had some attention-control this would be less. |
|  | 4.5 If Y/PY/NI to 4.4: Is it likely that assessment of the outcome was influenced by knowledge of intervention received? | | | PN |  |
|  | Risk of bias judgement | | | Some concerns | Outcome assessors were not blinded. Since the outcomes were self-reported the measurements could have been different between groups, but since the control group had some attention-control this would be less. |
| Bias in selection of the reported result | 5.1 Were the data that produced this result analysed in accordance with a pre-specified analysis plan that was finalized before unblinded outcome data were available for analysis? | | | Y | Protocol and analysis plan was published prospectively. |
|  | 5.2 ... multiple eligible outcome measurements (e.g. scales, definitions, time points) within the outcome domain? | | | N | All outcomes are reported as planned. |
|  | 5.3 ... multiple eligible analyses of the data? | | | N | All analyses are reported as planned, and also included unadjusted values. |
|  | Risk of bias judgement | | | Low | Protocol and analysis plan was published prospectively. All outcomes are reported as planned. All analyses are reported as planned, and also included unadjusted values. |
| Overall bias | Risk of bias judgement | | | High | Overall high risk of bias due to likelyhood of unblinded participants to behave differently in both groups, of missing outcome data and how outcomes was measured. |
|  |  |  |  |  |  |
|  |  |  |  |  |  |
| Unique ID | Logan 2014 Participation 6 months | Study ID | Logan 2014 | Assessor | MR |
| Ref or Label |  | Aim | assignment to intervention (the 'intention-to-treat' effect) |  |  |
| Experimental |  | Comparator |  | Source | Journal article(s) |
| Outcome | Participation 6 months | Results |  | Weight | 1 |
| Domain | Signalling question | | | Response | Comments |
| Bias arising from the randomization process | 1.1 Was the allocation sequence random? | | | PY | Web-based randomisation in varying blocks and stratified by site and age.  The randomisation was done by the local therapist meeting the participant after baseline values was collected and after the control intervention had been delivered. After entering baseline values the therapist got information about the allocation of the participant. |
|  | 1.2 Was the allocation sequence concealed until participants were enrolled and assigned to interventions? | | | PY |  |
|  | 1.3 Did baseline differences between intervention groups suggest a problem with the randomization process? | | | PY | Some baseline differences is present with worse prognostic factors for the intervention group. QoL is 45.9 vs 50.1, which would happen due to changce 1 in 15, NEADL 8.8 vs 10.1 (measure of basline activity levels) which would happen due to chance around 1 in 95, and time since stroke 43 months vs 37 months which would happen around 1 in 8. |
|  | Risk of bias judgement | | | Some concerns | Web-based randomisation in varying blocks and stratified by site and age.  The randomisation was done by the local therapist meeting the participant after baseline values was collected and after the control intervention had been delivered. After entering baseline values the therapist got information about the allocation of the participant.  Some baseline differences is present with worse prognostic factors for the intervention group. QoL is 45.9 vs 50.1, which would happen due to changce 1 in 15, NEADL 8.8 vs 10.1 (measure of basline activity levels) which would happen due to chance around 1 in 95, and time since stroke 43 months vs 37 months which would happen around 1 in 8. It is unclear why these baseline-inbalances are present for some prognostic variables, hence some conserns of risk of bias is present as they could potentially influence the outcomes down the line. |
| Bias due to deviations from intended interventions | 2.1.Were participants aware of their assigned intervention during the trial? | | | Y | Both participants and treatment providers were aware of the assigned interventions. |
|  | 2.2.Were carers and people delivering the interventions aware of participants' assigned intervention during the trial? | | | Y |  |
|  | 2.3. If Y/PY/NI to 2.1 or 2.2: Were there deviations from the intended intervention that arose because of the experimental context? | | | NI | No information provided about deviations. There is a likelyhood of non-protocol intervention being used, but since the control group got some attention control to improve outdoor mobility the risk is less. |
|  | 2.4 If Y/PY to 2.3: Were these deviations likely to have affected the outcome? | | | NA |  |
|  | 2.5. If Y/PY/NI to 2.4: Were these deviations from intended intervention balanced between groups? | | | NA |  |
|  | 2.6 Was an appropriate analysis used to estimate the effect of assignment to intervention? | | | Y |  |
|  | 2.7 If N/PN/NI to 2.6: Was there potential for a substantial impact (on the result) of the failure to analyse participants in the group to which they were randomized? | | | NA |  |
|  | Risk of bias judgement | | | Some concerns | Both participants and treatment providers were aware of the assigned interventions.  No information provided about deviations. There is a likelyhood of non-protocol intervention being used, but since the control group got some attention control to improve outdoor mobility the risk would be less. |
| Bias due to missing outcome data | 3.1 Were data for this outcome available for all, or nearly all, participants randomized? | | | PN | 8% loss to follow up in intervention and 15% in control group at 6 months, mostly becuase more participants withdrew. |
|  | 3.2 If N/PN/NI to 3.1: Is there evidence that result was not biased by missing outcome data? | | | N | Authors did not report data on who left the study. |
|  | 3.3 If N/PN to 3.2: Could missingness in the outcome depend on its true value? | | | PY | Almost double of withdrawn participants in the control group, which could influence the outcomes to some degree at 6 months. |
|  | 3.4 If Y/PY/NI to 3.3: Is it likely that missingness in the outcome depended on its true value? | | | PN |  |
|  | Risk of bias judgement | | | Some concerns | 8% loss to follow up in intervention and 15% in control group at 6 months, mostly becuase more participants withdrew. Authors did not report data on who left the study. Almost double of withdrawn participants in the control group, which could influence the outcomes to some degree at 6 months. |
| Bias in measurement of the outcome | 4.1 Was the method of measuring the outcome inappropriate? | | | PN | Better methods exist, but it would not be considered inappropriate. |
|  | 4.2 Could measurement or ascertainment of the outcome have differed between intervention groups? | | | PN | Probably not. |
|  | 4.3 Were outcome assessors aware of the intervention received by study participants? | | | Y | Participants were aware of their intervention |
|  | 4.4 If Y/PY/NI to 4.3: Could assessment of the outcome have been influenced by knowledge of intervention received? | | | PY | Outcome assessors were not blinded. Since the outcomes were self-reported the measurements could have been different between groups, but since the control group had some attention-control this would be less. |
|  | 4.5 If Y/PY/NI to 4.4: Is it likely that assessment of the outcome was influenced by knowledge of intervention received? | | | PN |  |
|  | Risk of bias judgement | | | Some concerns | Outcome assessors were not blinded. Since the outcomes were self-reported the measurements could have been different between groups, but since the control group had some attention-control this would be less. |
| Bias in selection of the reported result | 5.1 Were the data that produced this result analysed in accordance with a pre-specified analysis plan that was finalized before unblinded outcome data were available for analysis? | | | Y | Protocol and analysis plan was published prospectively. |
|  | 5.2 ... multiple eligible outcome measurements (e.g. scales, definitions, time points) within the outcome domain? | | | N | All outcomes are reported as planned. |
|  | 5.3 ... multiple eligible analyses of the data? | | | N | All analyses are reported as planned, and also included unadjusted values. |
|  | Risk of bias judgement | | | Low | Protocol and analysis plan was published prospectively. All outcomes are reported as planned. All analyses are reported as planned, and also included unadjusted values. |
| Overall bias | Risk of bias judgement | | | High | Overall high risk of bias due to high risk of bias in the measurement of the outcome due to participants rating their own participation levels, and some concers in the randomisation process due to unclear and large baseline differences, no description of methods for allocation consealment and some concerns for deviations from intended interventions due to having an inactive no intervention-comparison group. |
|  |  |  |  |  |  |
|  |  |  |  |  |  |
| Unique ID | Logan 2014 Participation 7 months | Study ID | Logan 2014 | Assessor | MR |
| Ref or Label |  | Aim | assignment to intervention (the 'intention-to-treat' effect) |  |  |
| Experimental |  | Comparator |  | Source | Journal article(s) |
| Outcome | Participation 7 months | Results |  | Weight | 1 |
| Domain | Signalling question | | | Response | Comments |
| Bias arising from the randomization process | 1.1 Was the allocation sequence random? | | | PY | Web-based randomisation in varying blocks and stratified by site and age.  The randomisation was done by the local therapist meeting the participant after baseline values was collected and after the control intervention had been delivered. After entering baseline values the therapist got information about the allocation of the participant. |
|  | 1.2 Was the allocation sequence concealed until participants were enrolled and assigned to interventions? | | | PY |  |
|  | 1.3 Did baseline differences between intervention groups suggest a problem with the randomization process? | | | PY | Some baseline differences is present with worse prognostic factors for the intervention group. QoL is 45.9 vs 50.1, which would happen due to changce 1 in 15, NEADL 8.8 vs 10.1 (measure of basline activity levels) which would happen due to chance around 1 in 95, and time since stroke 43 months vs 37 months which would happen around 1 in 8. |
|  | Risk of bias judgement | | | Some concerns | Web-based randomisation in varying blocks and stratified by site and age.  The randomisation was done by the local therapist meeting the participant after baseline values was collected and after the control intervention had been delivered. After entering baseline values the therapist got information about the allocation of the participant.  Some baseline differences is present with worse prognostic factors for the intervention group. QoL is 45.9 vs 50.1, which would happen due to changce 1 in 15, NEADL 8.8 vs 10.1 (measure of basline activity levels) which would happen due to chance around 1 in 95, and time since stroke 43 months vs 37 months which would happen around 1 in 8. It is unclear why these baseline-inbalances are present for some prognostic variables, hence some conserns of risk of bias is present as they could potentially influence the outcomes down the line. |
| Bias due to deviations from intended interventions | 2.1.Were participants aware of their assigned intervention during the trial? | | | Y | Both participants and treatment providers were aware of the assigned interventions. |
|  | 2.2.Were carers and people delivering the interventions aware of participants' assigned intervention during the trial? | | | Y |  |
|  | 2.3. If Y/PY/NI to 2.1 or 2.2: Were there deviations from the intended intervention that arose because of the experimental context? | | | NI | No information provided about deviations. There is a likelyhood of non-protocol intervention being used, but since the control group got some attention control to improve outdoor mobility the risk is less. |
|  | 2.4 If Y/PY to 2.3: Were these deviations likely to have affected the outcome? | | | NA |  |
|  | 2.5. If Y/PY/NI to 2.4: Were these deviations from intended intervention balanced between groups? | | | NA |  |
|  | 2.6 Was an appropriate analysis used to estimate the effect of assignment to intervention? | | | Y |  |
|  | 2.7 If N/PN/NI to 2.6: Was there potential for a substantial impact (on the result) of the failure to analyse participants in the group to which they were randomized? | | | NA |  |
|  | Risk of bias judgement | | | Some concerns | Both participants and treatment providers were aware of the assigned interventions.  No information provided about deviations. There is a likelyhood of non-protocol intervention being used, but since the control group got some attention control to improve outdoor mobility the risk would be less. |
| Bias due to missing outcome data | 3.1 Were data for this outcome available for all, or nearly all, participants randomized? | | | N | 19% loss to follow up in the intervention group and 25% missing in the control group at 12 months. |
|  | 3.2 If N/PN/NI to 3.1: Is there evidence that result was not biased by missing outcome data? | | | N |  |
|  | 3.3 If N/PN to 3.2: Could missingness in the outcome depend on its true value? | | | PY | Yes, more people withdrew from the trial in the control group, and the missing data in both groups are substancial and could influence the outcomes down the line. |
|  | 3.4 If Y/PY/NI to 3.3: Is it likely that missingness in the outcome depended on its true value? | | | PY |  |
|  | Risk of bias judgement | | | High | 19% loss to follow up in the intervention group and 25% missing in the control group at 12 months. Yes, more people withdrew from the trial in the control group, and the missing data in both groups are substancial and could influence the outcomes down the line. |
| Bias in measurement of the outcome | 4.1 Was the method of measuring the outcome inappropriate? | | | PN | Better methods exist, but it would not be considered inappropriate. |
|  | 4.2 Could measurement or ascertainment of the outcome have differed between intervention groups? | | | PN | Probably not. |
|  | 4.3 Were outcome assessors aware of the intervention received by study participants? | | | Y | Participants were aware of their intervention |
|  | 4.4 If Y/PY/NI to 4.3: Could assessment of the outcome have been influenced by knowledge of intervention received? | | | PY | Outcome assessors were not blinded. Since the outcomes were self-reported the measurements could have been different between groups, but since the control group had some attention-control this would be less. |
|  | 4.5 If Y/PY/NI to 4.4: Is it likely that assessment of the outcome was influenced by knowledge of intervention received? | | | PN |  |
|  | Risk of bias judgement | | | Some concerns | Outcome assessors were not blinded. Since the outcomes were self-reported the measurements could have been different between groups, but since the control group had some attention-control this would be less. |
| Bias in selection of the reported result | 5.1 Were the data that produced this result analysed in accordance with a pre-specified analysis plan that was finalized before unblinded outcome data were available for analysis? | | | Y | Protocol and analysis plan was published prospectively. |
|  | 5.2 ... multiple eligible outcome measurements (e.g. scales, definitions, time points) within the outcome domain? | | | N | All outcomes are reported as planned. |
|  | 5.3 ... multiple eligible analyses of the data? | | | N | All analyses are reported as planned, and also included unadjusted values. |
|  | Risk of bias judgement | | | Low | Protocol and analysis plan was published prospectively. All outcomes are reported as planned. All analyses are reported as planned, and also included unadjusted values. |
| Overall bias | Risk of bias judgement | | | High | Overall high risk of bias due to high risk of bias in the measurement of the outcome due to participants rating their own participation levels, and some concers in the randomisation process due to unclear and large baseline differences, no description of methods for allocation consealment and some concerns for deviations from intended interventions due to having an inactive no intervention-comparison group. |
|  |  |  |  |  |  |
|  |  |  |  |  |  |
| Unique ID | DePaul 2015 Activity outside the home 6 months | Study ID | DePaul 2015 | Assessor | MR |
| Ref or Label |  | Aim | assignment to intervention (the 'intention-to-treat' effect) |  |  |
| Experimental |  | Comparator |  | Source | Journal article(s) |
| Outcome | Activity outside the home 6 months | Results |  | Weight | 1 |
| Domain | Signalling question | | | Response | Comments |
| Bias arising from the randomization process | 1.1 Was the allocation sequence random? | | | Y | Used a central randomisation service, and allocation was sent by email to the research coordinator and clinicians. Used a stratified randomisation on walking speed block-randomisation of different sizes. |
|  | 1.2 Was the allocation sequence concealed until participants were enrolled and assigned to interventions? | | | Y |  |
|  | 1.3 Did baseline differences between intervention groups suggest a problem with the randomization process? | | |  | Similar baseline characteristic on all potential prognostic factors. |
|  | Risk of bias judgement | | | Low | Used a central randomisation service, and allocation was sent by email to the research coordinator and clinicians. Used a stratified randomisation on walking speed block-randomisation of different sizes. Similar baseline characteristic on all potential prognostic factors. |
| Bias due to deviations from intended interventions | 2.1.Were participants aware of their assigned intervention during the trial? | | | Y | Participants and treatment providers were aware of their allocation. In an effort to minimize expectation bias, participants and therapists in both groups received information that promoted the rationale and potential benefits of their assigned intervention and were blinded to the study hypotheses. The interventions were delivered in the same way, with the same lenght and intensity. |
|  | 2.2.Were carers and people delivering the interventions aware of participants' assigned intervention during the trial? | | | Y |  |
|  | 2.3. If Y/PY/NI to 2.1 or 2.2: Were there deviations from the intended intervention that arose because of the experimental context? | | | PN | No deviations are reported, apart from some difference in drop-out numbers. Since the two interventions are very similar and substancial measures were taken to make treatment providers and participants not having any preference, any deviations large enough to influence the outcome is probably unlikely. |
|  | 2.4 If Y/PY to 2.3: Were these deviations likely to have affected the outcome? | | | NA |  |
|  | 2.5. If Y/PY/NI to 2.4: Were these deviations from intended intervention balanced between groups? | | | NA |  |
|  | 2.6 Was an appropriate analysis used to estimate the effect of assignment to intervention? | | | Y | ITT-analysis was used, with multiple imputation of values for missing outcome data. Bias due to missing outcome is reflected in domain 3. |
|  | 2.7 If N/PN/NI to 2.6: Was there potential for a substantial impact (on the result) of the failure to analyse participants in the group to which they were randomized? | | | NA |  |
|  | Risk of bias judgement | | | Low | Participants and treatment providers were aware of their allocation. In an effort to minimize expectation bias, participants and therapists in both groups received information that promoted the rationale and potential benefits of their assigned intervention and were blinded to the study hypotheses. The interventions were delivered in the same way, with the same lenght and intensity.  No deviations are reported, apart from some difference in drop-out numbers. Since the two interventions are very similar and substancial measures were taken to make treatment providers and participants not having any preference, any deviations large enough to influence the outcome is probably unlikely.  ITT-analysis was used, with multiple imputation of values for missing outcome data. Bias due to missing outcome is reflected in domain 3. |
| Bias due to missing outcome data | 3.1 Were data for this outcome available for all, or nearly all, participants randomized? | | | N | 9/35 missing in the intervention group and 4/36 in the contron group at 2 months. Multipule imputation-techniques were used to generate outcome data for these participants. |
|  | 3.2 If N/PN/NI to 3.1: Is there evidence that result was not biased by missing outcome data? | | | N | More people left the intervention group due to "health status" and "unable to schedule", but no analyses are presented exploring the impact and potential bias, or sensitivity analyses based on the multiple imputation-techniques. |
|  | 3.3 If N/PN to 3.2: Could missingness in the outcome depend on its true value? | | | PY | As outcome data were missing due to health-status and unable to schedule patients - this could lead to excluding the participants with worse outcomes. This could influence the outcomes in the intervention group to the better. |
|  | 3.4 If Y/PY/NI to 3.3: Is it likely that missingness in the outcome depended on its true value? | | | PY |  |
|  | Risk of bias judgement | | | High | 9/35 missing in the intervention group and 4/36 in the contron group at 2 months. Multipule imputation-techniques were used to generate outcome data for these participants.  More people left the intervention group due to "health status" and "unable to schedule", but no analyses are presented exploring the impact and potential bias, or sensitivity analyses based on the multiple imputation-techniques.  As outcome data were missing due to health-status and unable to schedule patients - this could lead to excluding the participants with worse outcomes. This could influence the outcomes in the intervention group to the better. |
| Bias in measurement of the outcome | 4.1 Was the method of measuring the outcome inappropriate? | | | PN | Validated and commonly used method. |
|  | 4.2 Could measurement or ascertainment of the outcome have differed between intervention groups? | | | PN | Outcomes seem to have been reported the same in both groups, by a blinded assessor delivering the questionnaires. |
|  | 4.3 Were outcome assessors aware of the intervention received by study participants? | | | PY | Participants rated their own outcomes with the help of a blinded assessor, but were aware of their allocation. |
|  | 4.4 If Y/PY/NI to 4.3: Could assessment of the outcome have been influenced by knowledge of intervention received? | | | PN | Since the participants probably did not have a preference of intervention, it would be unlikely that the knowledge would influence their rated outcomes. |
|  | 4.5 If Y/PY/NI to 4.4: Is it likely that assessment of the outcome was influenced by knowledge of intervention received? | | | NA |  |
|  | Risk of bias judgement | | | Low | Validated and commonly used method.  Outcomes seem to have been reported the same in both groups, by a blinded assessor delivering the questionnaires.  Participants rated their own outcomes with the help of a blinded assessor, but were aware of their allocation.  Since the participants probably did not have a preference of intervention, it would be unlikely that the knowledge would influence their rated outcomes. |
| Bias in selection of the reported result | 5.1 Were the data that produced this result analysed in accordance with a pre-specified analysis plan that was finalized before unblinded outcome data were available for analysis? | | | Y | Protocol first posted in 2007, before study enrollment. |
|  | 5.2 ... multiple eligible outcome measurements (e.g. scales, definitions, time points) within the outcome domain? | | | N | All outcomes are specified in the protocol. |
|  | 5.3 ... multiple eligible analyses of the data? | | | PN | States in protocol to analyse outcomes by change-from-baseline, as well as mean differences, as well as adjusting for covariates. The result publication presents both these, and the analysis method does not influence the outcomes. |
|  | Risk of bias judgement | | | Low | Protocol first posted in 2007, before study enrollment.  All outcomes are specified in the protocol. States in protocol to analyse outcomes by change-from-baseline, as well as mean differences, as well as adjusting for covariates. The result publication presents both these, and the analysis method does not influence the outcomes. |
| Overall bias | Risk of bias judgement | | | High | Due to missing outcome data that is not equaliy distributed between the groups and due to reasons that is likely to impact the outcomes (26% in intervention group mostly due to health reasons, and 11% in control group) the overall risk of bias is judged to be high. |
|  |  |  |  |  |  |
|  |  |  |  |  |  |
| Unique ID | DePaul 2015 Participation 6 months | Study ID | DePaul 2015 | Assessor | MR |
| Ref or Label |  | Aim | assignment to intervention (the 'intention-to-treat' effect) |  |  |
| Experimental |  | Comparator |  | Source | Journal article(s) |
| Outcome | Participation 6 months | Results |  | Weight | 1 |
| Domain | Signalling question | | | Response | Comments |
| Bias arising from the randomization process | 1.1 Was the allocation sequence random? | | | Y | Used a central randomisation service, and allocation was sent by email to the research coordinator and clinicians. Used a stratified randomisation on walking speed block-randomisation of different sizes. |
|  | 1.2 Was the allocation sequence concealed until participants were enrolled and assigned to interventions? | | | Y |  |
|  | 1.3 Did baseline differences between intervention groups suggest a problem with the randomization process? | | |  | Similar baseline characteristic on all potential prognostic factors. |
|  | Risk of bias judgement | | | Low | Used a central randomisation service, and allocation was sent by email to the research coordinator and clinicians. Used a stratified randomisation on walking speed block-randomisation of different sizes. Similar baseline characteristic on all potential prognostic factors. |
| Bias due to deviations from intended interventions | 2.1.Were participants aware of their assigned intervention during the trial? | | | Y | Participants and treatment providers were aware of their allocation. In an effort to minimize expectation bias, participants and therapists in both groups received information that promoted the rationale and potential benefits of their assigned intervention and were blinded to the study hypotheses. The interventions were delivered in the same way, with the same lenght and intensity. |
|  | 2.2.Were carers and people delivering the interventions aware of participants' assigned intervention during the trial? | | | Y |  |
|  | 2.3. If Y/PY/NI to 2.1 or 2.2: Were there deviations from the intended intervention that arose because of the experimental context? | | | PN | No deviations are reported, apart from some difference in drop-out numbers. Since the two interventions are very similar and substancial measures were taken to make treatment providers and participants not having any preference, any deviations large enough to influence the outcome is probably unlikely. |
|  | 2.4 If Y/PY to 2.3: Were these deviations likely to have affected the outcome? | | | NA |  |
|  | 2.5. If Y/PY/NI to 2.4: Were these deviations from intended intervention balanced between groups? | | | NA |  |
|  | 2.6 Was an appropriate analysis used to estimate the effect of assignment to intervention? | | | Y | ITT-analysis was used, with multiple imputation of values for missing outcome data. Bias due to missing outcome is reflected in domain 3. |
|  | 2.7 If N/PN/NI to 2.6: Was there potential for a substantial impact (on the result) of the failure to analyse participants in the group to which they were randomized? | | | NA |  |
|  | Risk of bias judgement | | | Low | Participants and treatment providers were aware of their allocation. In an effort to minimize expectation bias, participants and therapists in both groups received information that promoted the rationale and potential benefits of their assigned intervention and were blinded to the study hypotheses. The interventions were delivered in the same way, with the same lenght and intensity.  No deviations are reported, apart from some difference in drop-out numbers. Since the two interventions are very similar and substancial measures were taken to make treatment providers and participants not having any preference, any deviations large enough to influence the outcome is probably unlikely.  ITT-analysis was used, with multiple imputation of values for missing outcome data. Bias due to missing outcome is reflected in domain 3. |
| Bias due to missing outcome data | 3.1 Were data for this outcome available for all, or nearly all, participants randomized? | | | N | 9/35 missing in the intervention group and 4/36 in the contron group at 2 months. Multipule imputation-techniques were used to generate outcome data for these participants. |
|  | 3.2 If N/PN/NI to 3.1: Is there evidence that result was not biased by missing outcome data? | | | N | More people left the intervention group due to "health status" and "unable to schedule", but no analyses are presented exploring the impact and potential bias, or sensitivity analyses based on the multiple imputation-techniques. |
|  | 3.3 If N/PN to 3.2: Could missingness in the outcome depend on its true value? | | | PY | As outcome data were missing due to health-status and unable to schedule patients - this could lead to excluding the participants with worse outcomes. This could influence the outcomes in the intervention group to the better. |
|  | 3.4 If Y/PY/NI to 3.3: Is it likely that missingness in the outcome depended on its true value? | | | PY |  |
|  | Risk of bias judgement | | | High | 9/35 missing in the intervention group and 4/36 in the contron group at 2 months. Multipule imputation-techniques were used to generate outcome data for these participants.  More people left the intervention group due to "health status" and "unable to schedule", but no analyses are presented exploring the impact and potential bias, or sensitivity analyses based on the multiple imputation-techniques.  As outcome data were missing due to health-status and unable to schedule patients - this could lead to excluding the participants with worse outcomes. This could influence the outcomes in the intervention group to the better. |
| Bias in measurement of the outcome | 4.1 Was the method of measuring the outcome inappropriate? | | | PN | Validated and commonly used method. |
|  | 4.2 Could measurement or ascertainment of the outcome have differed between intervention groups? | | | PN | Outcomes seem to have been reported the same in both groups, by a blinded assessor delivering the questionnaires. |
|  | 4.3 Were outcome assessors aware of the intervention received by study participants? | | | PY | Participants rated their own outcomes with the help of a blinded assessor, but were aware of their allocation. |
|  | 4.4 If Y/PY/NI to 4.3: Could assessment of the outcome have been influenced by knowledge of intervention received? | | | PN | Since the participants probably did not have a preference of intervention, it would be unlikely that the knowledge would influence their rated outcomes. |
|  | 4.5 If Y/PY/NI to 4.4: Is it likely that assessment of the outcome was influenced by knowledge of intervention received? | | | NA |  |
|  | Risk of bias judgement | | | Low | Validated and commonly used method.  Outcomes seem to have been reported the same in both groups, by a blinded assessor delivering the questionnaires.  Participants rated their own outcomes with the help of a blinded assessor, but were aware of their allocation.  Since the participants probably did not have a preference of intervention, it would be unlikely that the knowledge would influence their rated outcomes. |
| Bias in selection of the reported result | 5.1 Were the data that produced this result analysed in accordance with a pre-specified analysis plan that was finalized before unblinded outcome data were available for analysis? | | | Y | Protocol first posted in 2007, before study enrollment. |
|  | 5.2 ... multiple eligible outcome measurements (e.g. scales, definitions, time points) within the outcome domain? | | | N | All outcomes are specified in the protocol. |
|  | 5.3 ... multiple eligible analyses of the data? | | | PN | States in protocol to analyse outcomes by change-from-baseline, as well as mean differences, as well as adjusting for covariates. The result publication presents both these, and the analysis method does not influence the outcomes. |
|  | Risk of bias judgement | | | Low | Protocol first posted in 2007, before study enrollment.  All outcomes are specified in the protocol. States in protocol to analyse outcomes by change-from-baseline, as well as mean differences, as well as adjusting for covariates. The result publication presents both these, and the analysis method does not influence the outcomes. |
| Overall bias | Risk of bias judgement | | | High | Due to missing outcome data that is not equaliy distributed between the groups and due to reasons that is likely to impact the outcomes (26% in intervention group mostly due to health reasons, and 11% in control group) the overall risk of bias is judged to be high. |
|  |  |  |  |  |  |
|  |  |  |  |  |  |
| Unique ID | DePaul 2015 Minor harms 6 months | Study ID | DePaul 2015 | Assessor | MR |
| Ref or Label |  | Aim | assignment to intervention (the 'intention-to-treat' effect) |  |  |
| Experimental |  | Comparator |  | Source | Journal article(s) |
| Outcome | Minor adverse events 6 months | Results |  | Weight | 1 |
| Domain | Signalling question | | | Response | Comments |
| Bias arising from the randomization process | 1.1 Was the allocation sequence random? | | | Y | Used a central randomisation service, and allocation was sent by email to the research coordinator and clinicians. Used a stratified randomisation on walking speed block-randomisation of different sizes. |
|  | 1.2 Was the allocation sequence concealed until participants were enrolled and assigned to interventions? | | | Y |  |
|  | 1.3 Did baseline differences between intervention groups suggest a problem with the randomization process? | | |  | Similar baseline characteristic on all potential prognostic factors. |
|  | Risk of bias judgement | | | Low | Used a central randomisation service, and allocation was sent by email to the research coordinator and clinicians. Used a stratified randomisation on walking speed block-randomisation of different sizes. Similar baseline characteristic on all potential prognostic factors. |
| Bias due to deviations from intended interventions | 2.1.Were participants aware of their assigned intervention during the trial? | | | Y | Participants and treatment providers were aware of their allocation. In an effort to minimize expectation bias, participants and therapists in both groups received information that promoted the rationale and potential benefits of their assigned intervention and were blinded to the study hypotheses. The interventions were delivered in the same way, with the same lenght and intensity. |
|  | 2.2.Were carers and people delivering the interventions aware of participants' assigned intervention during the trial? | | | Y |  |
|  | 2.3. If Y/PY/NI to 2.1 or 2.2: Were there deviations from the intended intervention that arose because of the experimental context? | | | PN | No deviations are reported, apart from some difference in drop-out numbers. Since the two interventions are very similar and substancial measures were taken to make treatment providers and participants not having any preference, any deviations large enough to influence the outcome is probably unlikely. |
|  | 2.4 If Y/PY to 2.3: Were these deviations likely to have affected the outcome? | | | NA |  |
|  | 2.5. If Y/PY/NI to 2.4: Were these deviations from intended intervention balanced between groups? | | | NA |  |
|  | 2.6 Was an appropriate analysis used to estimate the effect of assignment to intervention? | | | Y | ITT-analysis was used, with multiple imputation of values for missing outcome data. Bias due to missing outcome is reflected in domain 3. |
|  | 2.7 If N/PN/NI to 2.6: Was there potential for a substantial impact (on the result) of the failure to analyse participants in the group to which they were randomized? | | | NA |  |
|  | Risk of bias judgement | | | Low | Participants and treatment providers were aware of their allocation. In an effort to minimize expectation bias, participants and therapists in both groups received information that promoted the rationale and potential benefits of their assigned intervention and were blinded to the study hypotheses. The interventions were delivered in the same way, with the same lenght and intensity.  No deviations are reported, apart from some difference in drop-out numbers. Since the two interventions are very similar and substancial measures were taken to make treatment providers and participants not having any preference, any deviations large enough to influence the outcome is probably unlikely.  ITT-analysis was used, with multiple imputation of values for missing outcome data. Bias due to missing outcome is reflected in domain 3. |
| Bias due to missing outcome data | 3.1 Were data for this outcome available for all, or nearly all, participants randomized? | | | N | 9/35 missing in the intervention group and 4/36 in the contron group at 2 months. Multipule imputation-techniques were used to generate outcome data for these participants. |
|  | 3.2 If N/PN/NI to 3.1: Is there evidence that result was not biased by missing outcome data? | | | N | More people left the intervention group due to "health status" and "unable to schedule", but no analyses are presented exploring the impact and potential bias, or sensitivity analyses based on the multiple imputation-techniques. |
|  | 3.3 If N/PN to 3.2: Could missingness in the outcome depend on its true value? | | | PY | As outcome data were missing due to health-status and unable to schedule patients - this could lead to excluding the participants with worse outcomes. This could influence the outcomes in the intervention group to the better. |
|  | 3.4 If Y/PY/NI to 3.3: Is it likely that missingness in the outcome depended on its true value? | | | PY |  |
|  | Risk of bias judgement | | | High | 9/35 missing in the intervention group and 4/36 in the contron group at 2 months. Multipule imputation-techniques were used to generate outcome data for these participants.  More people left the intervention group due to "health status" and "unable to schedule", but no analyses are presented exploring the impact and potential bias, or sensitivity analyses based on the multiple imputation-techniques.  As outcome data were missing due to health-status and unable to schedule patients - this could lead to excluding the participants with worse outcomes. This could influence the outcomes in the intervention group to the better. |
| Bias in measurement of the outcome | 4.1 Was the method of measuring the outcome inappropriate? | | | PN | Used a retrospective recollection of any falls, which is not an ideal way to collect outcome data on falls, but used the same procedure in both groups at least. |
|  | 4.2 Could measurement or ascertainment of the outcome have differed between intervention groups? | | | PN | Outcomes seem to have been reported the same in both groups, colleced by a blinded assessors. |
|  | 4.3 Were outcome assessors aware of the intervention received by study participants? | | | PY | Participants rated their own outcomes with the help of a blinded assessor, but were aware of their allocation. |
|  | 4.4 If Y/PY/NI to 4.3: Could assessment of the outcome have been influenced by knowledge of intervention received? | | | PN | Since the participants probably did not have a preference of intervention, it would be unlikely that the knowledge would influence their rated outcomes. |
|  | 4.5 If Y/PY/NI to 4.4: Is it likely that assessment of the outcome was influenced by knowledge of intervention received? | | | NA |  |
|  | Risk of bias judgement | | | Low | Used a retrospective recollection of any falls, which is not an ideal way to collect outcome data on falls, but used the same procedure in both groups at least.  Outcomes seem to have been reported the same in both groups, colleced by a blinded assessors.  Participants rated their own outcomes with the help of a blinded assessor, but were aware of their allocation.  Since the participants probably did not have a preference of intervention, it would be unlikely that the knowledge would influence their rated outcomes. |
| Bias in selection of the reported result | 5.1 Were the data that produced this result analysed in accordance with a pre-specified analysis plan that was finalized before unblinded outcome data were available for analysis? | | | Y | Protocol first posted in 2007, before study enrollment. |
|  | 5.2 ... multiple eligible outcome measurements (e.g. scales, definitions, time points) within the outcome domain? | | | N | All outcomes are specified in the protocol. |
|  | 5.3 ... multiple eligible analyses of the data? | | | PN | States in protocol to analyse outcomes by change-from-baseline, as well as mean differences, as well as adjusting for covariates. The result publication presents both these, and the analysis method does not influence the outcomes. |
|  | Risk of bias judgement | | | Low | Protocol first posted in 2007, before study enrollment.  All outcomes are specified in the protocol. States in protocol to analyse outcomes by change-from-baseline, as well as mean differences, as well as adjusting for covariates. The result publication presents both these, and the analysis method does not influence the outcomes. |
| Overall bias | Risk of bias judgement | | | High | Due to missing outcome data that is not equaliy distributed between the groups and due to reasons that is likely to impact the outcomes (26% in intervention group mostly due to health reasons, and 11% in control group) the overall risk of bias is judged to be high. |
|  |  |  |  |  |  |
|  |  |  |  |  |  |
| Unique ID | DePaul 2015 Major harms 6 months | Study ID | DePaul 2015 | Assessor | MR |
| Ref or Label |  | Aim | assignment to intervention (the 'intention-to-treat' effect) |  |  |
| Experimental |  | Comparator |  | Source | Journal article(s) |
| Outcome | Major adverse events 6 months | Results |  | Weight | 1 |
| Domain | Signalling question | | | Response | Comments |
| Bias arising from the randomization process | 1.1 Was the allocation sequence random? | | | Y | Used a central randomisation service, and allocation was sent by email to the research coordinator and clinicians. Used a stratified randomisation on walking speed block-randomisation of different sizes. |
|  | 1.2 Was the allocation sequence concealed until participants were enrolled and assigned to interventions? | | | Y |  |
|  | 1.3 Did baseline differences between intervention groups suggest a problem with the randomization process? | | |  | Similar baseline characteristic on all potential prognostic factors. |
|  | Risk of bias judgement | | | Low | Used a central randomisation service, and allocation was sent by email to the research coordinator and clinicians. Used a stratified randomisation on walking speed block-randomisation of different sizes. Similar baseline characteristic on all potential prognostic factors. |
| Bias due to deviations from intended interventions | 2.1.Were participants aware of their assigned intervention during the trial? | | | Y | Participants and treatment providers were aware of their allocation. In an effort to minimize expectation bias, participants and therapists in both groups received information that promoted the rationale and potential benefits of their assigned intervention and were blinded to the study hypotheses. The interventions were delivered in the same way, with the same lenght and intensity. |
|  | 2.2.Were carers and people delivering the interventions aware of participants' assigned intervention during the trial? | | | Y |  |
|  | 2.3. If Y/PY/NI to 2.1 or 2.2: Were there deviations from the intended intervention that arose because of the experimental context? | | | PN | No deviations are reported, apart from some difference in drop-out numbers. Since the two interventions are very similar and substancial measures were taken to make treatment providers and participants not having any preference, any deviations large enough to influence the outcome is probably unlikely. |
|  | 2.4 If Y/PY to 2.3: Were these deviations likely to have affected the outcome? | | | NA |  |
|  | 2.5. If Y/PY/NI to 2.4: Were these deviations from intended intervention balanced between groups? | | | NA |  |
|  | 2.6 Was an appropriate analysis used to estimate the effect of assignment to intervention? | | | Y | ITT-analysis was used, with multiple imputation of values for missing outcome data. Bias due to missing outcome is reflected in domain 3. |
|  | 2.7 If N/PN/NI to 2.6: Was there potential for a substantial impact (on the result) of the failure to analyse participants in the group to which they were randomized? | | | NA |  |
|  | Risk of bias judgement | | | Low | Participants and treatment providers were aware of their allocation. In an effort to minimize expectation bias, participants and therapists in both groups received information that promoted the rationale and potential benefits of their assigned intervention and were blinded to the study hypotheses. The interventions were delivered in the same way, with the same lenght and intensity.  No deviations are reported, apart from some difference in drop-out numbers. Since the two interventions are very similar and substancial measures were taken to make treatment providers and participants not having any preference, any deviations large enough to influence the outcome is probably unlikely.  ITT-analysis was used, with multiple imputation of values for missing outcome data. Bias due to missing outcome is reflected in domain 3. |
| Bias due to missing outcome data | 3.1 Were data for this outcome available for all, or nearly all, participants randomized? | | | N | 9/35 missing in the intervention group and 4/36 in the contron group at 2 months. Multipule imputation-techniques were used to generate outcome data for these participants. |
|  | 3.2 If N/PN/NI to 3.1: Is there evidence that result was not biased by missing outcome data? | | | N | More people left the intervention group due to "health status" and "unable to schedule", but no analyses are presented exploring the impact and potential bias, or sensitivity analyses based on the multiple imputation-techniques. |
|  | 3.3 If N/PN to 3.2: Could missingness in the outcome depend on its true value? | | | PY | As outcome data were missing due to health-status and unable to schedule patients - this could lead to excluding the participants with worse outcomes. This could influence the outcomes in the intervention group to the better. |
|  | 3.4 If Y/PY/NI to 3.3: Is it likely that missingness in the outcome depended on its true value? | | | PY |  |
|  | Risk of bias judgement | | | High | 9/35 missing in the intervention group and 4/36 in the contron group at 2 months. Multipule imputation-techniques were used to generate outcome data for these participants.  More people left the intervention group due to "health status" and "unable to schedule", but no analyses are presented exploring the impact and potential bias, or sensitivity analyses based on the multiple imputation-techniques.  As outcome data were missing due to health-status and unable to schedule patients - this could lead to excluding the participants with worse outcomes. This could influence the outcomes in the intervention group to the better. |
| Bias in measurement of the outcome | 4.1 Was the method of measuring the outcome inappropriate? | | | PN | Serious adverse events were collected from participant or caregiver reports and confirmed with the participants primary physician. Not an ideal way to measure outcomes, but are similar between the groups. |
|  | 4.2 Could measurement or ascertainment of the outcome have differed between intervention groups? | | | PN | Outcomes seem to have been reported the same in both groups, by a blinded assessor. |
|  | 4.3 Were outcome assessors aware of the intervention received by study participants? | | | PY | Participants rated their own outcomes with the help of a blinded assessor, but were aware of their allocation. |
|  | 4.4 If Y/PY/NI to 4.3: Could assessment of the outcome have been influenced by knowledge of intervention received? | | | PN | Since the participants probably did not have a preference of intervention, it would be unlikely that the knowledge would influence their rated outcomes. |
|  | 4.5 If Y/PY/NI to 4.4: Is it likely that assessment of the outcome was influenced by knowledge of intervention received? | | | NA |  |
|  | Risk of bias judgement | | | Low | Serious adverse events were collected from participant or caregiver reports and confirmed with the participants primary physician. Not an ideal way to measure outcomes, but are similar between the groups.  Outcomes seem to have been reported the same in both groups, by a blinded assessor.  Participants rated their own outcomes with the help of a blinded assessor, but were aware of their allocation.  Since the participants probably did not have a preference of intervention, it would be unlikely that the knowledge would influence their rated outcomes. |
| Bias in selection of the reported result | 5.1 Were the data that produced this result analysed in accordance with a pre-specified analysis plan that was finalized before unblinded outcome data were available for analysis? | | | Y | Protocol first posted in 2007, before study enrollment. |
|  | 5.2 ... multiple eligible outcome measurements (e.g. scales, definitions, time points) within the outcome domain? | | | N | All outcomes are specified in the protocol. |
|  | 5.3 ... multiple eligible analyses of the data? | | | PN | States in protocol to analyse outcomes by change-from-baseline, as well as mean differences, as well as adjusting for covariates. The result publication presents both these, and the analysis method does not influence the outcomes. |
|  | Risk of bias judgement | | | Low | Protocol first posted in 2007, before study enrollment.  All outcomes are specified in the protocol. States in protocol to analyse outcomes by change-from-baseline, as well as mean differences, as well as adjusting for covariates. The result publication presents both these, and the analysis method does not influence the outcomes. |
| Overall bias | Risk of bias judgement | | | High | Due to missing outcome data that is not equaliy distributed between the groups and due to reasons that is likely to impact the outcomes (26% in intervention group mostly due to health reasons, and 11% in control group) the overall risk of bias is judged to be high. |
|  |  |  |  |  |  |
|  |  |  |  |  |  |
| Unique ID | Kim 2014 Participation 6 months | Study ID | Kim 2014 | Assessor | MR |
| Ref or Label |  | Aim | assignment to intervention (the 'intention-to-treat' effect) |  |  |
| Experimental |  | Comparator |  | Source | Journal article(s) |
| Outcome | Participation 6 months | Results |  | Weight | 1 |
| Domain | Signalling question | | | Response | Comments |
| Bias arising from the randomization process | 1.1 Was the allocation sequence random? | | | NI | No information available about the randomisation sequence or method. States that envelopes with letters inside were used, but no information if and how the allocation sequence was consealed. |
|  | 1.2 Was the allocation sequence concealed until participants were enrolled and assigned to interventions? | | | NI |  |
|  | 1.3 Did baseline differences between intervention groups suggest a problem with the randomization process? | | | PN | Some difference are present between the groups, the largest difference is time since the stoke (190 vs 272 days, p-value 0.06), but could be due to the small group sizes. |
|  | Risk of bias judgement | | | Some concerns | No information available about the randomisation sequence or method. States that envelopes with letters inside were used, but no information if and how the allocation sequence was consealed. Some difference are present between the groups, the largest difference is time since the stoke (190 vs 272 days, p-value 0.06), but could be due to the small group sizes. |
| Bias due to deviations from intended interventions | 2.1.Were participants aware of their assigned intervention during the trial? | | | Y | Neither participants or treatment providers were blinded. |
|  | 2.2.Were carers and people delivering the interventions aware of participants' assigned intervention during the trial? | | | Y |  |
|  | 2.3. If Y/PY/NI to 2.1 or 2.2: Were there deviations from the intended intervention that arose because of the experimental context? | | | PY | Since the control group did not receive any real intervention and control compared to the intervention-add-on and were aware of this, it is likely that there were deviations that could incfluence in the self-reported participation levels. |
|  | 2.4 If Y/PY to 2.3: Were these deviations likely to have affected the outcome? | | | PY |  |
|  | 2.5. If Y/PY/NI to 2.4: Were these deviations from intended intervention balanced between groups? | | | PN |  |
|  | 2.6 Was an appropriate analysis used to estimate the effect of assignment to intervention? | | | PY | Complete case-analysis, 4 out of 26 participants were not included due to dropping out from the study. Analysed by assignment to intervention. |
|  | 2.7 If N/PN/NI to 2.6: Was there potential for a substantial impact (on the result) of the failure to analyse participants in the group to which they were randomized? | | | NA |  |
|  | Risk of bias judgement | | | High | Neither participants or treatment providers were blinded. Since the control group did not receive any real intervention compared to the intervention-add-on and were aware of this, it is likely that there were deviations in either group that could incfluence in the self-reported participation levels. |
| Bias due to missing outcome data | 3.1 Were data for this outcome available for all, or nearly all, participants randomized? | | | PN | 15% missing in intervention group and 15% missing in control group. |
|  | 3.2 If N/PN/NI to 3.1: Is there evidence that result was not biased by missing outcome data? | | | N | No attempts was reported to account for any impact of the participants leaving the study. |
|  | 3.3 If N/PN to 3.2: Could missingness in the outcome depend on its true value? | | | PY | Participants left the study due to "health conditions, personal reasons or discharge". Not stated which reasons were related to each group. These issues is not likely to be random, and would be likely to influence outcome measures. |
|  | 3.4 If Y/PY/NI to 3.3: Is it likely that missingness in the outcome depended on its true value? | | | PY |  |
|  | Risk of bias judgement | | | High | 15% missing in intervention group and 15% missing in control group.  No attempts was reported to account for any impact of the participants leaving the study.  Participants left the study due to "health conditions, personal reasons or discharge". Not stated which reasons were related to each group. These issues is not likely to be random, and would be likely to influence outcome measures. |
| Bias in measurement of the outcome | 4.1 Was the method of measuring the outcome inappropriate? | | | N | Validated method to measure participation. |
|  | 4.2 Could measurement or ascertainment of the outcome have differed between intervention groups? | | | PN | Measurements were done while admitted to the ward by a blinded deliverer, but the participants themselves were not blinded. |
|  | 4.3 Were outcome assessors aware of the intervention received by study participants? | | | Y |  |
|  | 4.4 If Y/PY/NI to 4.3: Could assessment of the outcome have been influenced by knowledge of intervention received? | | | PY | Awareness of the additional intervention to walk outside could influence the participants rating of participation after the intervention. |
|  | 4.5 If Y/PY/NI to 4.4: Is it likely that assessment of the outcome was influenced by knowledge of intervention received? | | | PY |  |
|  | Risk of bias judgement | | | High | Validated method to measure participation.  Measurements were done while admitted to the ward by a blinded deliverer, but the participants themselves were not blinded.  Awareness of the additional intervention to walk outside could influence the participants rating of participation after the intervention. |
| Bias in selection of the reported result | 5.1 Were the data that produced this result analysed in accordance with a pre-specified analysis plan that was finalized before unblinded outcome data were available for analysis? | | | NI | No pre-specified protocol reported or available. |
|  | 5.2 ... multiple eligible outcome measurements (e.g. scales, definitions, time points) within the outcome domain? | | | NI | Just one subscale of Stroke Impact Scale is reported, which is a little odd since the authors probably have results for all outcome domains, but not enough reason for serious consern. |
|  | 5.3 ... multiple eligible analyses of the data? | | | NI | The authors report both change from baseline and post-difference measures, so no real indication of selective decisions on analysis methods. |
|  | Risk of bias judgement | | | Some concerns | No pre-specified protocol reported or available.  Just one subscale of Stroke Impact Scale is reported, which is a little odd since the authors probably have results for all outcome domains, but not enough reason for serious consern.  The authors report both change from baseline and post-difference measures, so no real indication of selective decisions on analysis methods. |
| Overall bias | Risk of bias judgement | | | High | Overall high risk of bias, based on 3 high risk of bias judgements and 2 some consern-judgements. |
|  |  |  |  |  |  |
|  |  |  |  |  |  |
| Unique ID | Lord 2008 Engagement in everyday life activities 6 months | Study ID | Lord 2008 | Assessor | MR |
| Ref or Label |  | Aim | assignment to intervention (the 'intention-to-treat' effect) |  |  |
| Experimental |  | Comparator |  | Source | Journal article(s) |
| Outcome | Activity outside the home 6 months | Results |  | Weight | 1 |
| Domain | Signalling question | | | Response | Comments |
| Bias arising from the randomization process | 1.1 Was the allocation sequence random? | | | Y | Computer-generated randomisation by a central unit by envelopes. Study coordinators were not part of the randomisation and allocation. |
|  | 1.2 Was the allocation sequence concealed until participants were enrolled and assigned to interventions? | | | PY |  |
|  | 1.3 Did baseline differences between intervention groups suggest a problem with the randomization process? | | | PN | No substancial baseline differences present |
|  | Risk of bias judgement | | | Low | Computer-generated randomisation by a central unit by envelopes. Study coordinators were not part of the randomisation and allocation.  No substancial baseline differences present. |
| Bias due to deviations from intended interventions | 2.1.Were participants aware of their assigned intervention during the trial? | | | Y | Participants and treatment providers were aware of the allocated interventions. |
|  | 2.2.Were carers and people delivering the interventions aware of participants' assigned intervention during the trial? | | | Y |  |
|  | 2.3. If Y/PY/NI to 2.1 or 2.2: Were there deviations from the intended intervention that arose because of the experimental context? | | | PY | Deviations would be possible due to non-blinding and different approaches to outdoor mobility where participants might have a preference and seek out other intervetions. One indication is that the physiotherapy group had 5 people leaving during the intervention and control group only 1. In the physiotherapy group 1 person left due to being in a specific group but no other deviations are described in the study. |
|  | 2.4 If Y/PY to 2.3: Were these deviations likely to have affected the outcome? | | | PY | These deviations could influence the outcomes, but since the two interventions were given in the same intensity it is more unlikely that they had a major influence. |
|  | 2.5. If Y/PY/NI to 2.4: Were these deviations from intended intervention balanced between groups? | | | PY | The deviations would probably be somewhat balanced between the groups. |
|  | 2.6 Was an appropriate analysis used to estimate the effect of assignment to intervention? | | | PY | Modified ITT, using a complete case analysis. |
|  | 2.7 If N/PN/NI to 2.6: Was there potential for a substantial impact (on the result) of the failure to analyse participants in the group to which they were randomized? | | | NA |  |
|  | Risk of bias judgement | | | Some concerns | Participants and treatment providers were aware of the allocated interventions.  Deviations would be possible due to non-blinding and different approaches to outdoor mobility where participants might have a preference and seek out other intervetions. One indication is that the physiotherapy group had 5 people leaving during the intervention and control group only 1. In the physiotherapy group 1 person left due to being in a specific group but no other deviations are described in the study.  These deviations could influence the outcomes, but since the two interventions were given in the same intensity it is more unlikely that they had a major influence.  The deviations would probably be somewhat balanced between the groups. Modified ITT, using a complete case analysis. |
| Bias due to missing outcome data | 3.1 Were data for this outcome available for all, or nearly all, participants randomized? | | | N | 6/21 (29%) missing in physiotherapy group and 3/15 (20%) in the outdoor group. |
|  | 3.2 If N/PN/NI to 3.1: Is there evidence that result was not biased by missing outcome data? | | | N | No sensitvity analyses or imputations were made. |
|  | 3.3 If N/PN to 3.2: Could missingness in the outcome depend on its true value? | | | Y | It is likely that people with worse outcomes would leave the study, and amount and reason is not balanced between the groups. |
|  | 3.4 If Y/PY/NI to 3.3: Is it likely that missingness in the outcome depended on its true value? | | | Y |  |
|  | Risk of bias judgement | | | High | 6/21 (29%) missing in physiotherapy group and 3/15 (20%) in the outdoor group.  No sensitvity analyses or imputations were made.  It is likely that people with worse outcomes would leave the study, and amount and reason is not balanced between the groups. |
| Bias in measurement of the outcome | 4.1 Was the method of measuring the outcome inappropriate? | | | PN | Validated questionnaire was used. |
|  | 4.2 Could measurement or ascertainment of the outcome have differed between intervention groups? | | | PN | Measurements seem to have been conduced the same way, but is not very well defined. |
|  | 4.3 Were outcome assessors aware of the intervention received by study participants? | | | Y | Outcome assessors were aware of the allocation. |
|  | 4.4 If Y/PY/NI to 4.3: Could assessment of the outcome have been influenced by knowledge of intervention received? | | | PY | The assessment could have differed between the groups, but it is not very likely since they got a similar amount and intensity of therap. |
|  | 4.5 If Y/PY/NI to 4.4: Is it likely that assessment of the outcome was influenced by knowledge of intervention received? | | | PN |  |
|  | Risk of bias judgement | | | Some concerns | Validated questionnaire was used.  Measurements seem to have been conduced the same way, but is not very well defined.  Outcome assessors were aware of the allocation. The assessment could have differed between the groups, but it is not very likely since they got a similar amount and intensity of therap. |
| Bias in selection of the reported result | 5.1 Were the data that produced this result analysed in accordance with a pre-specified analysis plan that was finalized before unblinded outcome data were available for analysis? | | | NI | No protocol is to be found. |
|  | 5.2 ... multiple eligible outcome measurements (e.g. scales, definitions, time points) within the outcome domain? | | | PY | Multiple possible outcome measures and selecting specific questions is possible. |
|  | 5.3 ... multiple eligible analyses of the data? | | | PN | Analyses of the outcomes are presented in several ways, and no indicuation of selection of just some analyses are present. |
|  | Risk of bias judgement | | | High | No protocol is to be found.  Multiple possible outcome measures and selecting specific questions from these measures are possible.  Analyses of the outcomes are presented in several ways, and no indicuation of selection of just some analyses are present. |
| Overall bias | Risk of bias judgement | | | High | Overall high risk of bias due to large missing outcome data and no available protocol with the potential of selective reporting, as well as lack of blinding or full measures to avoid deviations between groups. |
|  |  |  |  |  |  |
|  |  |  |  |  |  |
| Unique ID | Lord 2008 Participation 6 months | Study ID | Lord 2008 | Assessor | MR |
| Ref or Label |  | Aim | assignment to intervention (the 'intention-to-treat' effect) |  |  |
| Experimental |  | Comparator |  | Source | Journal article(s) |
| Outcome | Participation 6 months | Results |  | Weight | 1 |
| Domain | Signalling question | | | Response | Comments |
| Bias arising from the randomization process | 1.1 Was the allocation sequence random? | | | Y | Computer-generated randomisation by a central unit by envelopes. Study coordinators were not part of the randomisation and allocation. |
|  | 1.2 Was the allocation sequence concealed until participants were enrolled and assigned to interventions? | | | PY |  |
|  | 1.3 Did baseline differences between intervention groups suggest a problem with the randomization process? | | | PN | No substancial baseline differences present |
|  | Risk of bias judgement | | | Low | Computer-generated randomisation by a central unit by envelopes. Study coordinators were not part of the randomisation and allocation.  No substancial baseline differences present. |
| Bias due to deviations from intended interventions | 2.1.Were participants aware of their assigned intervention during the trial? | | | Y | Participants and treatment providers were aware of the allocated interventions. |
|  | 2.2.Were carers and people delivering the interventions aware of participants' assigned intervention during the trial? | | | Y |  |
|  | 2.3. If Y/PY/NI to 2.1 or 2.2: Were there deviations from the intended intervention that arose because of the experimental context? | | | PY | Deviations would be possible due to non-blinding and different approaches to outdoor mobility where participants might have a preference and seek out other intervetions. One indication is that the physiotherapy group had 5 people leaving during the intervention and control group only 1. In the physiotherapy group 1 person left due to being in a specific group but no other deviations are described in the study. |
|  | 2.4 If Y/PY to 2.3: Were these deviations likely to have affected the outcome? | | | PY | These deviations could influence the outcomes, but since the two interventions were given in the same intensity it is more unlikely that they had a major influence. |
|  | 2.5. If Y/PY/NI to 2.4: Were these deviations from intended intervention balanced between groups? | | | PY | The deviations would probably be somewhat balanced between the groups. |
|  | 2.6 Was an appropriate analysis used to estimate the effect of assignment to intervention? | | | PY | Modified ITT, using a complete case analysis. |
|  | 2.7 If N/PN/NI to 2.6: Was there potential for a substantial impact (on the result) of the failure to analyse participants in the group to which they were randomized? | | | NA |  |
|  | Risk of bias judgement | | | Some concerns | Participants and treatment providers were aware of the allocated interventions.  Deviations would be possible due to non-blinding and different approaches to outdoor mobility where participants might have a preference and seek out other intervetions. One indication is that the physiotherapy group had 5 people leaving during the intervention and control group only 1. In the physiotherapy group 1 person left due to being in a specific group but no other deviations are described in the study.  These deviations could influence the outcomes, but since the two interventions were given in the same intensity it is more unlikely that they had a major influence.  The deviations would probably be somewhat balanced between the groups. Modified ITT, using a complete case analysis. |
| Bias due to missing outcome data | 3.1 Were data for this outcome available for all, or nearly all, participants randomized? | | | N | 6/21 (29%) missing in physiotherapy group and 3/15 (20%) in the outdoor group. |
|  | 3.2 If N/PN/NI to 3.1: Is there evidence that result was not biased by missing outcome data? | | | N | No sensitvity analyses or imputations were made. |
|  | 3.3 If N/PN to 3.2: Could missingness in the outcome depend on its true value? | | | Y | It is likely that people with worse outcomes would leave the study, and amount and reason is not balanced between the groups. |
|  | 3.4 If Y/PY/NI to 3.3: Is it likely that missingness in the outcome depended on its true value? | | | Y |  |
|  | Risk of bias judgement | | | High | 6/21 (29%) missing in physiotherapy group and 3/15 (20%) in the outdoor group.  No sensitvity analyses or imputations were made.  It is likely that people with worse outcomes would leave the study, and amount and reason is not balanced between the groups. |
| Bias in measurement of the outcome | 4.1 Was the method of measuring the outcome inappropriate? | | | PN | Validated questionnaire was used. |
|  | 4.2 Could measurement or ascertainment of the outcome have differed between intervention groups? | | | PN | Measurements seem to have been conduced the same way, but is not very well defined. |
|  | 4.3 Were outcome assessors aware of the intervention received by study participants? | | | Y | Outcome assessors were aware of the allocation. |
|  | 4.4 If Y/PY/NI to 4.3: Could assessment of the outcome have been influenced by knowledge of intervention received? | | | PY | The assessment could have differed between the groups, but it is not very likely since they got a similar amount and intensity of therap. |
|  | 4.5 If Y/PY/NI to 4.4: Is it likely that assessment of the outcome was influenced by knowledge of intervention received? | | | PN |  |
|  | Risk of bias judgement | | | Some concerns | Validated questionnaire was used.  Measurements seem to have been conduced the same way, but is not very well defined.  Outcome assessors were aware of the allocation. The assessment could have differed between the groups, but it is not very likely since they got a similar amount and intensity of therap. |
| Bias in selection of the reported result | 5.1 Were the data that produced this result analysed in accordance with a pre-specified analysis plan that was finalized before unblinded outcome data were available for analysis? | | | NI | No protocol is to be found. |
|  | 5.2 ... multiple eligible outcome measurements (e.g. scales, definitions, time points) within the outcome domain? | | | PY | Multiple possible outcome measures and selecting specific questions is possible. |
|  | 5.3 ... multiple eligible analyses of the data? | | | PN | Analyses of the outcomes are presented in several ways, and no indicuation of selection of just some analyses are present. |
|  | Risk of bias judgement | | | High | No protocol is to be found.  Multiple possible outcome measures and selecting specific questions from these measures are possible.  Analyses of the outcomes are presented in several ways, and no indicuation of selection of just some analyses are present. |
| Overall bias | Risk of bias judgement | | | High | Overall high risk of bias due to large missing outcome data and no available protocol with the potential of selective reporting, as well as lack of blinding or full measures to avoid deviations between groups. |
|  |  |  |  |  |  |
|  |  |  |  |  |  |
| Unique ID | Lord 2008 Major harms 6 months | Study ID | Lord 2008 | Assessor | MR |
| Ref or Label |  | Aim | assignment to intervention (the 'intention-to-treat' effect) |  |  |
| Experimental |  | Comparator |  | Source | Journal article(s) |
| Outcome | Major adverse events 6 months | Results |  | Weight | 1 |
| Domain | Signalling question | | | Response | Comments |
| Bias arising from the randomization process | 1.1 Was the allocation sequence random? | | | Y | Computer-generated randomisation by a central unit by envelopes. Study coordinators were not part of the randomisation and allocation. |
|  | 1.2 Was the allocation sequence concealed until participants were enrolled and assigned to interventions? | | | PY |  |
|  | 1.3 Did baseline differences between intervention groups suggest a problem with the randomization process? | | | PN | No substancial baseline differences present |
|  | Risk of bias judgement | | | Low | Computer-generated randomisation by a central unit by envelopes. Study coordinators were not part of the randomisation and allocation.  No substancial baseline differences present. |
| Bias due to deviations from intended interventions | 2.1.Were participants aware of their assigned intervention during the trial? | | | Y | Participants and treatment providers were aware of the allocated interventions. |
|  | 2.2.Were carers and people delivering the interventions aware of participants' assigned intervention during the trial? | | | Y |  |
|  | 2.3. If Y/PY/NI to 2.1 or 2.2: Were there deviations from the intended intervention that arose because of the experimental context? | | | PY | Deviations would be possible due to non-blinding and different approaches to outdoor mobility where participants might have a preference and seek out other intervetions. One indication is that the physiotherapy group had 5 people leaving during the intervention and control group only 1. In the physiotherapy group 1 person left due to being in a specific group but no other deviations are described in the study. |
|  | 2.4 If Y/PY to 2.3: Were these deviations likely to have affected the outcome? | | | PY | These deviations could influence the outcomes, but since the two interventions were given in the same intensity it is more unlikely that they had a major influence. |
|  | 2.5. If Y/PY/NI to 2.4: Were these deviations from intended intervention balanced between groups? | | | PY | The deviations would probably be somewhat balanced between the groups. |
|  | 2.6 Was an appropriate analysis used to estimate the effect of assignment to intervention? | | | PY | Modified ITT, using a complete case analysis. |
|  | 2.7 If N/PN/NI to 2.6: Was there potential for a substantial impact (on the result) of the failure to analyse participants in the group to which they were randomized? | | | NA |  |
|  | Risk of bias judgement | | | Some concerns | Participants and treatment providers were aware of the allocated interventions.  Deviations would be possible due to non-blinding and different approaches to outdoor mobility where participants might have a preference and seek out other intervetions. One indication is that the physiotherapy group had 5 people leaving during the intervention and control group only 1. In the physiotherapy group 1 person left due to being in a specific group but no other deviations are described in the study.  These deviations could influence the outcomes, but since the two interventions were given in the same intensity it is more unlikely that they had a major influence.  The deviations would probably be somewhat balanced between the groups. Modified ITT, using a complete case analysis. |
| Bias due to missing outcome data | 3.1 Were data for this outcome available for all, or nearly all, participants randomized? | | | N | 6/21 (29%) missing in physiotherapy group and 3/15 (20%) in the outdoor group. |
|  | 3.2 If N/PN/NI to 3.1: Is there evidence that result was not biased by missing outcome data? | | | N | No sensitvity analyses or imputations were made. |
|  | 3.3 If N/PN to 3.2: Could missingness in the outcome depend on its true value? | | | Y | It is likely that people with worse outcomes would leave the study, and amount and reason is not balanced between the groups. |
|  | 3.4 If Y/PY/NI to 3.3: Is it likely that missingness in the outcome depended on its true value? | | | Y |  |
|  | Risk of bias judgement | | | High | 6/21 (29%) missing in physiotherapy group and 3/15 (20%) in the outdoor group.  No sensitvity analyses or imputations were made.  It is likely that people with worse outcomes would leave the study, and amount and reason is not balanced between the groups. |
| Bias in measurement of the outcome | 4.1 Was the method of measuring the outcome inappropriate? | | | PN | No mention of how stroke-evenets were collected or measured (physiotherapists listening in to patients, or through medical record) but it is a quite robust outcome to measure. |
|  | 4.2 Could measurement or ascertainment of the outcome have differed between intervention groups? | | | PN | We could assume that measurements of stroke-events could have been conduced the same way. |
|  | 4.3 Were outcome assessors aware of the intervention received by study participants? | | | NI | No mention if outcome assessors of stroke-events were blinded or not. |
|  | 4.4 If Y/PY/NI to 4.3: Could assessment of the outcome have been influenced by knowledge of intervention received? | | | PY | The assessment could have differed between the groups, but would probably be unlikely. |
|  | 4.5 If Y/PY/NI to 4.4: Is it likely that assessment of the outcome was influenced by knowledge of intervention received? | | | PN |  |
|  | Risk of bias judgement | | | Some concerns | No mention of how stroke-evenets were collected or measured, but it is a quite robust outcome to measure.  We could assume that measurements of stroke-events could have been conduced the same way.  No mention if outcome assessors of stroke-events were blinded or not.  The assessment could have differed between the groups, but would probably be unlikely. |
| Bias in selection of the reported result | 5.1 Were the data that produced this result analysed in accordance with a pre-specified analysis plan that was finalized before unblinded outcome data were available for analysis? | | | NI | No protocol is to be found. |
|  | 5.2 ... multiple eligible outcome measurements (e.g. scales, definitions, time points) within the outcome domain? | | | PY | Multiple possible outcome measures and selecting specific questions is possible. |
|  | 5.3 ... multiple eligible analyses of the data? | | | PN | Analyses of the outcomes are presented in several ways, and no indicuation of selection of just some analyses are present. |
|  | Risk of bias judgement | | | High | No protocol is to be found.  Multiple possible outcome measures and selecting specific questions from these measures are possible.  Analyses of the outcomes are presented in several ways, and no indicuation of selection of just some analyses are present. |
| Overall bias | Risk of bias judgement | | | High | Overall high risk of bias due to large missing outcome data and no available protocol with the potential of selective reporting, as well as lack of blinding or full measures to avoid deviations between groups. |
|  |  |  |  |  |  |
|  |  |  |  |  |  |
| Unique ID | Magaziner 2019 Major harms 6 months | Study ID | Magaziner 2019 | Assessor | MR |
| Ref or Label |  | Aim | assignment to intervention (the 'intention-to-treat' effect) |  |  |
| Experimental |  | Comparator |  | Source | Journal article(s) |
| Outcome | Major adverse events 6 months | Results |  | Weight | 1 |
| Domain | Signalling question | | | Response | Comments |
| Bias arising from the randomization process | 1.1 Was the allocation sequence random? | | | PY | The randomisation sequence and consealment is sparsely described, but could assumed to be random. Allocation was delivered in a web-based format by external staff using different block sizes. |
|  | 1.2 Was the allocation sequence concealed until participants were enrolled and assigned to interventions? | | | PY |  |
|  | 1.3 Did baseline differences between intervention groups suggest a problem with the randomization process? | | | PN | Only one indication of baseline imbalance (BMI below 20 13 vs 4 in each group) Around 1 in 25 to occur by chance. |
|  | Risk of bias judgement | | | Low | The randomisation sequence and consealment is sparsely described, but could assumed to be random. Allocation was delivered in a web-based format by staff not actively involved in the study using different block sizes. Only one indication of baseline imbalance (BMI below 20 13 vs 4 in each group) Around 1 in 25 to occur by chance. |
| Bias due to deviations from intended interventions | 2.1.Were participants aware of their assigned intervention during the trial? | | | Y | Participants and therapists delivering the intervention were not blinded. |
|  | 2.2.Were carers and people delivering the interventions aware of participants' assigned intervention during the trial? | | | Y |  |
|  | 2.3. If Y/PY/NI to 2.1 or 2.2: Were there deviations from the intended intervention that arose because of the experimental context? | | | PY | Not explored, but 15 persons withdrew from the intervention and only 6 from the control group - indicating that participants behaved differently due to knowledge of the allocation. |
|  | 2.4 If Y/PY to 2.3: Were these deviations likely to have affected the outcome? | | | PN | The adverse events collected are robust, and would be unlikely to have influenced by the knowledge of the intervention. |
|  | 2.5. If Y/PY/NI to 2.4: Were these deviations from intended intervention balanced between groups? | | | NA |  |
|  | 2.6 Was an appropriate analysis used to estimate the effect of assignment to intervention? | | | PY | Complete case analysis was used. |
|  | 2.7 If N/PN/NI to 2.6: Was there potential for a substantial impact (on the result) of the failure to analyse participants in the group to which they were randomized? | | | NA |  |
|  | Risk of bias judgement | | | Some concerns | Participants and therapists delivering the intervention were not blinded.  Not explored, but 15 persons withdrew from the intervention and only 6 from the control group - indicating that participants behaved differently due to knowledge of the allocation.  The adverse events collected are robust, and would be unlikely to have influenced by the knowledge of the intervention. Modified ITT-analysis/complete case analysis was used. |
| Bias due to missing outcome data | 3.1 Were data for this outcome available for all, or nearly all, participants randomized? | | | N | 23/105 and 13/105 missingness of outcome data in each group. |
|  | 3.2 If N/PN/NI to 3.1: Is there evidence that result was not biased by missing outcome data? | | | PN | Imputations without clear descriptions how was used for most participants, but no sensitivty analysis or similar was conducted. |
|  | 3.3 If N/PN to 3.2: Could missingness in the outcome depend on its true value? | | | Y | Since more people left or was excluded from analysis in the intervention group, and participants with higher risk of adverse events due to any reason would probably be more likely to withdraw, it is likely that this could influence the results. |
|  | 3.4 If Y/PY/NI to 3.3: Is it likely that missingness in the outcome depended on its true value? | | | PY |  |
|  | Risk of bias judgement | | | High | 23/105 and 13/105 missingness of outcome data in each group.  Imputations without clear descriptions how was used for most participants, but no sensitivty analysis or similar was conducted.  Since more people left or was excluded from analysis in the intervention group, and participants with higher risk of adverse events due to any reason would probably be more likely to withdraw, it is likely that this could influence the results. |
| Bias in measurement of the outcome | 4.1 Was the method of measuring the outcome inappropriate? | | | PN | Adverse events was collected by telephone interview every 4 weeks over phone and by physiotherapists in relation to each training session. |
|  | 4.2 Could measurement or ascertainment of the outcome have differed between intervention groups? | | | NI | No information available if outcome collection differed between groups. |
|  | 4.3 Were outcome assessors aware of the intervention received by study participants? | | | Y | Participants and therapists collecting adverse events were aware of their allocation. |
|  | 4.4 If Y/PY/NI to 4.3: Could assessment of the outcome have been influenced by knowledge of intervention received? | | | PY | It is possible that the intervention group, knowing that they are in the active intervention, would report adverse events differently. It's not judged very likely that it would have a substancial impact on reported advese events though. |
|  | 4.5 If Y/PY/NI to 4.4: Is it likely that assessment of the outcome was influenced by knowledge of intervention received? | | | PN |  |
|  | Risk of bias judgement | | | Some concerns | Adverse events was collected by telephone interview every 4 weeks over phone and by physiotherapists in relation to each training session.  No information available if outcome collection differed between groups.  Participants and therapists collecting adverse events were aware of their allocation.  It is possible that the intervention group, knowing that they are in the active intervention, would report adverse events differently. It's not judged very likely that it would have a substancial impact on reported advese events though. |
| Bias in selection of the reported result | 5.1 Were the data that produced this result analysed in accordance with a pre-specified analysis plan that was finalized before unblinded outcome data were available for analysis? | | | PY | Pre-specified analysis plan is available. |
|  | 5.2 ... multiple eligible outcome measurements (e.g. scales, definitions, time points) within the outcome domain? | | | PN | No mention of adverse events in the protocol, or how to analyse them. But the reported adverse events are quite comprehensive and unlikely to have been seleced or analysed in a specific way. |
|  | 5.3 ... multiple eligible analyses of the data? | | | PN |  |
|  | Risk of bias judgement | | | Low | Pre-specified analysis plan is available.No mention of adverse events in the protocol, or how to analyse them. But the reported adverse events reported are quite comprehensive and unlikely to have been seleced or analysed in a specific way. |
| Overall bias | Risk of bias judgement | | | High | Overall high risk of bias due to high risk of bias in missing outcome data (23% vs 13%) and some consern for measurement and collection of the adverse events and deviations from intended intervention due to risk of participants behaving differently due to knowledge of their allocated intervention. |
|  |  |  |  |  |  |
|  |  |  |  |  |  |
| Unique ID | Magaziner 2019 Minor harms 6 months | Study ID | Magaziner 2019 | Assessor | MR |
| Ref or Label |  | Aim | assignment to intervention (the 'intention-to-treat' effect) |  |  |
| Experimental |  | Comparator |  | Source | Journal article(s) |
| Outcome | Minor adverse events 6 months | Results |  | Weight | 1 |
| Domain | Signalling question | | | Response | Comments |
| Bias arising from the randomization process | 1.1 Was the allocation sequence random? | | | PY | The randomisation sequence and consealment is sparsely described, but could assumed to be random. Allocation was delivered in a web-based format by external staff using different block sizes. |
|  | 1.2 Was the allocation sequence concealed until participants were enrolled and assigned to interventions? | | | PY |  |
|  | 1.3 Did baseline differences between intervention groups suggest a problem with the randomization process? | | | PN | Only one indication of baseline imbalance (BMI below 20 13 vs 4 in each group) Around 1 in 25 to occur by chance. |
|  | Risk of bias judgement | | | Low | The randomisation sequence and consealment is sparsely described, but could assumed to be random. Allocation was delivered in a web-based format by staff not actively involved in the study using different block sizes. Only one indication of baseline imbalance (BMI below 20 13 vs 4 in each group) Around 1 in 25 to occur by chance. |
| Bias due to deviations from intended interventions | 2.1.Were participants aware of their assigned intervention during the trial? | | | Y | Participants and therapists delivering the intervention were not blinded. |
|  | 2.2.Were carers and people delivering the interventions aware of participants' assigned intervention during the trial? | | | Y |  |
|  | 2.3. If Y/PY/NI to 2.1 or 2.2: Were there deviations from the intended intervention that arose because of the experimental context? | | | PY | Not explored, but 15 persons withdrew from the intervention and only 6 from the control group - indicating that participants behaved differently due to knowledge of the allocation. |
|  | 2.4 If Y/PY to 2.3: Were these deviations likely to have affected the outcome? | | | PN | The adverse events collected are robust, and would be unlikely to have influenced by the knowledge of the intervention. |
|  | 2.5. If Y/PY/NI to 2.4: Were these deviations from intended intervention balanced between groups? | | | NA |  |
|  | 2.6 Was an appropriate analysis used to estimate the effect of assignment to intervention? | | | PY | Complete case analysis was used. |
|  | 2.7 If N/PN/NI to 2.6: Was there potential for a substantial impact (on the result) of the failure to analyse participants in the group to which they were randomized? | | | NA |  |
|  | Risk of bias judgement | | | Some concerns | Participants and therapists delivering the intervention were not blinded.  Not explored, but 15 persons withdrew from the intervention and only 6 from the control group - indicating that participants behaved differently due to knowledge of the allocation.  The adverse events collected are robust, and would be unlikely to have influenced by the knowledge of the intervention. Modified ITT-analysis/complete case analysis was used. |
| Bias due to missing outcome data | 3.1 Were data for this outcome available for all, or nearly all, participants randomized? | | | N | 23/105 and 13/105 missingness of outcome data in each group. |
|  | 3.2 If N/PN/NI to 3.1: Is there evidence that result was not biased by missing outcome data? | | | PN | Imputations without clear descriptions how was used for most participants, but no sensitivty analysis or similar was conducted. |
|  | 3.3 If N/PN to 3.2: Could missingness in the outcome depend on its true value? | | | Y | Since more people left or was excluded from analysis in the intervention group, and participants with higher risk of adverse events due to any reason would probably be more likely to withdraw, it is likely that this could influence the results. |
|  | 3.4 If Y/PY/NI to 3.3: Is it likely that missingness in the outcome depended on its true value? | | | PY |  |
|  | Risk of bias judgement | | | High | 23/105 and 13/105 missingness of outcome data in each group.  Imputations without clear descriptions how was used for most participants, but no sensitivty analysis or similar was conducted.  Since more people left or was excluded from analysis in the intervention group, and participants with higher risk of adverse events due to any reason would probably be more likely to withdraw, it is likely that this could influence the results. |
| Bias in measurement of the outcome | 4.1 Was the method of measuring the outcome inappropriate? | | | PN | Adverse events was collected by telephone interview every 4 weeks over phone and by physiotherapists in relation to each training session. |
|  | 4.2 Could measurement or ascertainment of the outcome have differed between intervention groups? | | | NI | No information available if outcome collection differed between groups. |
|  | 4.3 Were outcome assessors aware of the intervention received by study participants? | | | Y | Participants were aware of their allocation. |
|  | 4.4 If Y/PY/NI to 4.3: Could assessment of the outcome have been influenced by knowledge of intervention received? | | | PY | It is possible that the intervention group, knowing that they are in the active intervention, would report adverse events differently. It's not judged very likely that it would have a substancial impact on reported advese events though. |
|  | 4.5 If Y/PY/NI to 4.4: Is it likely that assessment of the outcome was influenced by knowledge of intervention received? | | | PN |  |
|  | Risk of bias judgement | | | Some concerns | Adverse events was collected by telephone interview every 4 weeks over phone and by physiotherapists in relation to each training session.  No information available if outcome collection differed between groups.  Participants and therapists collecting adverse events were aware of their allocation.  It is possible that the intervention group, knowing that they are in the active intervention, would report adverse events differently. It's not judged very likely that it would have a substancial impact on reported advese events though. |
| Bias in selection of the reported result | 5.1 Were the data that produced this result analysed in accordance with a pre-specified analysis plan that was finalized before unblinded outcome data were available for analysis? | | | PY | Pre-specified analysis plan is available. |
|  | 5.2 ... multiple eligible outcome measurements (e.g. scales, definitions, time points) within the outcome domain? | | | PN | No mention of adverse events in the protocol, or how to analyse them. But the reported adverse events are quite comprehensive and unlikely to have been seleced or analysed in a specific way. |
|  | 5.3 ... multiple eligible analyses of the data? | | | PN |  |
|  | Risk of bias judgement | | | Low | Pre-specified analysis plan is available.No mention of adverse events in the protocol, or how to analyse them. But the reported adverse events reported are quite comprehensive and unlikely to have been seleced or analysed in a specific way. |
| Overall bias | Risk of bias judgement | | | High | Overall high risk of bias due to high risk of bias in missing outcome data (23% vs 13%) and some consern for measurement and collection of the adverse events and deviations from intended intervention due to risk of participants behaving differently due to knowledge of their allocated intervention. |
|  |  |  |  |  |  |
|  |  |  |  |  |  |
| Unique ID | Ullrich 2021 Activity outside the home 6 months | Study ID | Ullrich 2021 | Assessor | MR |
| Ref or Label |  | Aim | assignment to intervention (the 'intention-to-treat' effect) |  |  |
| Experimental |  | Comparator |  | Source | Journal article(s) |
| Outcome | Activity outside 6 months | Results |  | Weight | 1 |
| Domain | Signalling question | | | Response | Comments |
| Bias arising from the randomization process | 1.1 Was the allocation sequence random? | | | PY | Urn-based randomisation, statified by gender. Randomisation conducted after baseline values where taken, by an external person. Probably random sequence. No further information is given on the randomisation procedure, or if allocation was consealed. |
|  | 1.2 Was the allocation sequence concealed until participants were enrolled and assigned to interventions? | | | NI |  |
|  | 1.3 Did baseline differences between intervention groups suggest a problem with the randomization process? | | | PN | Similar baseline values. Falls last year is 76% vs 56%, which could happen 1 out of around 21 times. Judged as a chance-imbalance. |
|  | Risk of bias judgement | | | Some concerns | Urn-based randomisation, statified by gender. Randomisation conducted after baseline values where taken, by an external person. Probably random sequence. No further information is given on the randomisation procedure, or if allocation was consealed.  Similar baseline values. Falls last year is 76% vs 56%, which could happen 1 out of around 21 times. Judged as a chance-imbalance. |
| Bias due to deviations from intended interventions | 2.1.Were participants aware of their assigned intervention during the trial? | | | PY | Article states that participants were blinded and not aware of their allocation, but due to the nature of the interventions which are different, the participants were aware of their allocation, but was bacially a well designed attention-control with attempts to minimize knowledge about each intervention. Treatment providers were not blinded due to the nature of the interventions. |
|  | 2.2.Were carers and people delivering the interventions aware of participants' assigned intervention during the trial? | | | N |  |
|  | 2.3. If Y/PY/NI to 2.1 or 2.2: Were there deviations from the intended intervention that arose because of the experimental context? | | | PN | The interventions were planned to be delivered in the same intensity and followed up in the same way. No deviations is reported or explored in the article, but since the authors took every precaucion to design the interventions similar as attention control, it is probably not likely. |
|  | 2.4 If Y/PY to 2.3: Were these deviations likely to have affected the outcome? | | | NA |  |
|  | 2.5. If Y/PY/NI to 2.4: Were these deviations from intended intervention balanced between groups? | | | NA |  |
|  | 2.6 Was an appropriate analysis used to estimate the effect of assignment to intervention? | | | PY | ITT-analysis with imputations for missining values. |
|  | 2.7 If N/PN/NI to 2.6: Was there potential for a substantial impact (on the result) of the failure to analyse participants in the group to which they were randomized? | | | NA |  |
|  | Risk of bias judgement | | | Low | Article states that participants were blinded and not aware of their allocation, but due to the nature of the interventions which are different, the participants were aware of their allocation, but was bacially a well designed attention-control with attempts to minimize knowledge about each intervention. Treatment providers were not blinded due to the nature of the interventions.  The interventions were planned to be delivered in the same intensity and followed up in the same way. No deviations is reported or explored in the article, but since the authors took every precaucion to design the interventions similar as attention control, it is probably not likely. ITT-analysis with imputations for missining values. |
| Bias due to missing outcome data | 3.1 Were data for this outcome available for all, or nearly all, participants randomized? | | | N | Missing outcome data was 21% in the intervention group and 22% in the control group. |
|  | 3.2 If N/PN/NI to 3.1: Is there evidence that result was not biased by missing outcome data? | | | N | Imputations was done for missing outcome data based on "maximum likelyhood" without further specifications. Unlikely that this would correct for any bias. |
|  | 3.3 If N/PN to 3.2: Could missingness in the outcome depend on its true value? | | | Y | Missing outcome data is likely to differ between the groups, which was also compared in an analysis where physical function and apathy had a significant difference. Leaving the study due to "motivation" differed (2 vs 6) and due to serious medical events (9 vs 2) also indicates people with worse outcomes not being included, which is unevenly distrubuted. |
|  | 3.4 If Y/PY/NI to 3.3: Is it likely that missingness in the outcome depended on its true value? | | | Y |  |
|  | Risk of bias judgement | | | High | Missing outcome data was 21% in the intervention group and 22% in the control group. Imputations was done for missing outcome data based on "maximum likelyhood" without further specifications. Unlikely that this would correct for any bias. Missing outcome data is likely to differ between the groups, which was also compared in an analysis where physical function and apathy had a significant difference. Leaving the study due to "motivation" differed (2 vs 6) and due to serious medical events (9 vs 2) also indicates people with worse outcomes not being included, which is unevenly distrubuted. |
| Bias in measurement of the outcome | 4.1 Was the method of measuring the outcome inappropriate? | | | N | Yes, LSA-CI is a validated instrument. |
|  | 4.2 Could measurement or ascertainment of the outcome have differed between intervention groups? | | | PN | No description of any differences in outcome collection is stated, and would be unlikely. |
|  | 4.3 Were outcome assessors aware of the intervention received by study participants? | | | PY | Outcome assessors (participants) were aware of their allocation. |
|  | 4.4 If Y/PY/NI to 4.3: Could assessment of the outcome have been influenced by knowledge of intervention received? | | | PN | Since the intervention and control were similar and participants many attempts were made to have participants not having a preference, it is unlikely that it could influcence the outcome assessment by knowledge of the intervention group. |
|  | 4.5 If Y/PY/NI to 4.4: Is it likely that assessment of the outcome was influenced by knowledge of intervention received? | | | NA |  |
|  | Risk of bias judgement | | | Low | Yes, LSA-CI is a validated instrument.  No description of any differences in outcome collection is stated, and would be unlikely.  Outcome assessors (participants) were aware of their allocation.  Since the intervention and control were similar and participants many attempts were made to have participants not having a preference, it is unlikely that it could influcence the outcome assessment by knowledge of the intervention group. |
| Bias in selection of the reported result | 5.1 Were the data that produced this result analysed in accordance with a pre-specified analysis plan that was finalized before unblinded outcome data were available for analysis? | | | Y | Pre-specified protocol is available and published. |
|  | 5.2 ... multiple eligible outcome measurements (e.g. scales, definitions, time points) within the outcome domain? | | | PN | All reported outcomes are pre-specified. |
|  | 5.3 ... multiple eligible analyses of the data? | | | PN | Specific analyses are not presented in the protocol, but "standard" and the most common analyses seems to have been used. |
|  | Risk of bias judgement | | | Low | Pre-specified protocol is available and published.  All reported outcomes are pre-specified.  Specific analyses are not presented in the protocol, but "standard" and the most common analyses seems to have been used. |
| Overall bias | Risk of bias judgement | | | High | High risk of buas due to missing outcome data |
|  |  |  |  |  |  |
|  |  |  |  |  |  |
| Unique ID | Ullrich 2021 Qualirty of life 6 months | Study ID | Ullrich 2021 | Assessor | MR |
| Ref or Label |  | Aim | assignment to intervention (the 'intention-to-treat' effect) |  |  |
| Experimental |  | Comparator |  | Source | Journal article(s) |
| Outcome | Quality of life 6 months | Results |  | Weight | 1 |
| Domain | Signalling question | | | Response | Comments |
| Bias arising from the randomization process | 1.1 Was the allocation sequence random? | | | PY | Urn-based randomisation, statified by gender. Randomisation conducted after baseline values where taken, by an external person. Probably random sequence. No further information is given on the randomisation procedure, or if allocation was consealed. |
|  | 1.2 Was the allocation sequence concealed until participants were enrolled and assigned to interventions? | | | NI |  |
|  | 1.3 Did baseline differences between intervention groups suggest a problem with the randomization process? | | | PN | Similar baseline values. Falls last year is 76% vs 56%, which could happen 1 out of around 21 times. Judged as a chance-imbalance. |
|  | Risk of bias judgement | | | Some concerns | Urn-based randomisation, statified by gender. Randomisation conducted after baseline values where taken, by an external person. Probably random sequence. No further information is given on the randomisation procedure, or if allocation was consealed.  Similar baseline values. Falls last year is 76% vs 56%, which could happen 1 out of around 21 times. Judged as a chance-imbalance. |
| Bias due to deviations from intended interventions | 2.1.Were participants aware of their assigned intervention during the trial? | | | PY | Article states that participants were blinded and not aware of their allocation, but due to the nature of the interventions which are different, the participants were aware of their allocation, but was bacially a well designed attention-control with attempts to minimize knowledge about each intervention. Treatment providers were not blinded due to the nature of the interventions. |
|  | 2.2.Were carers and people delivering the interventions aware of participants' assigned intervention during the trial? | | | N |  |
|  | 2.3. If Y/PY/NI to 2.1 or 2.2: Were there deviations from the intended intervention that arose because of the experimental context? | | | PN | The interventions were planned to be delivered in the same intensity and followed up in the same way. No deviations is reported or explored in the article, but since the authors took every precaucion to design the interventions similar as attention control, it is probably not likely. |
|  | 2.4 If Y/PY to 2.3: Were these deviations likely to have affected the outcome? | | | NA |  |
|  | 2.5. If Y/PY/NI to 2.4: Were these deviations from intended intervention balanced between groups? | | | NA |  |
|  | 2.6 Was an appropriate analysis used to estimate the effect of assignment to intervention? | | | PY | ITT-analysis with imputations for missining values. |
|  | 2.7 If N/PN/NI to 2.6: Was there potential for a substantial impact (on the result) of the failure to analyse participants in the group to which they were randomized? | | | NA |  |
|  | Risk of bias judgement | | | Low | Article states that participants were blinded and not aware of their allocation, but due to the nature of the interventions which are different, the participants were aware of their allocation, but was bacially a well designed attention-control with attempts to minimize knowledge about each intervention. Treatment providers were not blinded due to the nature of the interventions.  The interventions were planned to be delivered in the same intensity and followed up in the same way. No deviations is reported or explored in the article, but since the authors took every precaucion to design the interventions similar as attention control, it is probably not likely. ITT-analysis with imputations for missining values. |
| Bias due to missing outcome data | 3.1 Were data for this outcome available for all, or nearly all, participants randomized? | | | N | Missing outcome data was 21% in the intervention group and 22% in the control group. |
|  | 3.2 If N/PN/NI to 3.1: Is there evidence that result was not biased by missing outcome data? | | | N | Imputations was done for missing outcome data based on "maximum likelyhood" without further specifications. Unlikely that this would correct for any bias. |
|  | 3.3 If N/PN to 3.2: Could missingness in the outcome depend on its true value? | | | Y | Missing outcome data is likely to differ between the groups, which was also compared in an analysis where physical function and apathy had a significant difference. Leaving the study due to "motivation" differed (2 vs 6) and due to serious medical events (9 vs 2) also indicates people with worse outcomes not being included, which is unevenly distrubuted. |
|  | 3.4 If Y/PY/NI to 3.3: Is it likely that missingness in the outcome depended on its true value? | | | Y |  |
|  | Risk of bias judgement | | | High | Missing outcome data was 21% in the intervention group and 22% in the control group. Imputations was done for missing outcome data based on "maximum likelyhood" without further specifications. Unlikely that this would correct for any bias. Missing outcome data is likely to differ between the groups, which was also compared in an analysis where physical function and apathy had a significant difference. Leaving the study due to "motivation" differed (2 vs 6) and due to serious medical events (9 vs 2) also indicates people with worse outcomes not being included, which is unevenly distrubuted. |
| Bias in measurement of the outcome | 4.1 Was the method of measuring the outcome inappropriate? | | | N | Yes, EQ5D is a validated instrument. |
|  | 4.2 Could measurement or ascertainment of the outcome have differed between intervention groups? | | | PN | No description of any differences in outcome collection is stated, and would be unlikely. |
|  | 4.3 Were outcome assessors aware of the intervention received by study participants? | | | PY | Outcome assessors (participants) were aware of their allocation. |
|  | 4.4 If Y/PY/NI to 4.3: Could assessment of the outcome have been influenced by knowledge of intervention received? | | | PN | Since the intervention and control were similar and participants many attempts were made to have participants not having a preference, it is unlikely that it could influcence the outcome assessment by knowledge of the intervention group. |
|  | 4.5 If Y/PY/NI to 4.4: Is it likely that assessment of the outcome was influenced by knowledge of intervention received? | | | NA |  |
|  | Risk of bias judgement | | | Low | Yes, LSA-CI is a validated instrument.  No description of any differences in outcome collection is stated, and would be unlikely.  Outcome assessors (participants) were aware of their allocation.  Since the intervention and control were similar and participants many attempts were made to have participants not having a preference, it is unlikely that it could influcence the outcome assessment by knowledge of the intervention group. |
| Bias in selection of the reported result | 5.1 Were the data that produced this result analysed in accordance with a pre-specified analysis plan that was finalized before unblinded outcome data were available for analysis? | | | Y | Pre-specified protocol is available and published. |
|  | 5.2 ... multiple eligible outcome measurements (e.g. scales, definitions, time points) within the outcome domain? | | | PN | All reported outcomes are pre-specified. |
|  | 5.3 ... multiple eligible analyses of the data? | | | PN | Specific analyses are not presented in the protocol, but "standard" and the most common analyses seems to have been used. |
|  | Risk of bias judgement | | | Low | Pre-specified protocol is available and published.  All reported outcomes are pre-specified.  Specific analyses are not presented in the protocol, but "standard" and the most common analyses seems to have been used. |
| Overall bias | Risk of bias judgement | | | High | High risk of bias due to missing outcome data |
|  |  |  |  |  |  |
|  |  |  |  |  |  |
| Unique ID | Turunen 2020 Engagement in everyday life activities 6 months | Study ID | Turunen 2020 | Assessor | MR |
| Ref or Label |  | Aim | assignment to intervention (the 'intention-to-treat' effect) |  |  |
| Experimental |  | Comparator |  | Source | Journal article(s) |
| Outcome | Engagement in everyday life activities 6 months | Results |  | Weight | 1 |
| Domain | Signalling question | | | Response | Comments |
| Bias arising from the randomization process | 1.1 Was the allocation sequence random? | | | PY | Computer generated random sequence using blocks of 10 and stratfied by gender, age and gait-speed was conducted after baseline assessments by an external statistician and in sealed opaque envelopes. No other detailed descriptions of methods of allocation consealment is given. |
|  | 1.2 Was the allocation sequence concealed until participants were enrolled and assigned to interventions? | | | PY |  |
|  | 1.3 Did baseline differences between intervention groups suggest a problem with the randomization process? | | | N | No substancial differences in baseline characteristcs. |
|  | Risk of bias judgement | | | Low | Computer generated random sequence using blocks of 10, conducted after baseline assessments by an external statistician and in sealed opaque envelopes. No other detailed descriptions of methods of allocation consealment is given.  No substancial differences in baseline characteristcs. |
| Bias due to deviations from intended interventions | 2.1.Were participants aware of their assigned intervention during the trial? | | | Y | Participants and treatment providers were aware of their group allocation. |
|  | 2.2.Were carers and people delivering the interventions aware of participants' assigned intervention during the trial? | | | Y |  |
|  | 2.3. If Y/PY/NI to 2.1 or 2.2: Were there deviations from the intended intervention that arose because of the experimental context? | | | PY | Drop-out analysis was done for physiotherapy-visits outside of the intervention which showed no difference. No other information is reported about any potential deviations, but it is likely that the usual-care group would behave differently when they are enrolled in an intervention to improve physical activity and outdoor mobility and is not given any intervention. The intervention group would probably also be reminded more often to report and remember outdoor mobility. |
|  | 2.4 If Y/PY to 2.3: Were these deviations likely to have affected the outcome? | | | PY | It is likely that these deviations could influence the outcome through other aspects than visiting physiotherapists during the study period. |
|  | 2.5. If Y/PY/NI to 2.4: Were these deviations from intended intervention balanced between groups? | | | PN | The deviations would probably not be balaced between the groups. |
|  | 2.6 Was an appropriate analysis used to estimate the effect of assignment to intervention? | | | PY | Analysed according to ITT. |
|  | 2.7 If N/PN/NI to 2.6: Was there potential for a substantial impact (on the result) of the failure to analyse participants in the group to which they were randomized? | | | NA |  |
|  | Risk of bias judgement | | | High | Participants and treatment providers were aware of their group allocation.  Drop-out analysis was done for physiotherapy-visits outside of the intervention which showed no difference. No other information is reported about any potential deviations, but it is likely that the usual-care group would behave differently when they are enrolled in an intervention to improve physical activity and outdoor mobility and is not given any intervention. The intervention group would probably also be reminded more often to report and remember outdoor mobility. It is likely that these deviations could influence the outcome through other aspects than visiting physiotherapists during the study period.  The deviations would probably not be balaced between the groups. Analysed according to ITT. |
| Bias due to missing outcome data | 3.1 Were data for this outcome available for all, or nearly all, participants randomized? | | | PN | 5% missing outcome data at 6 month and 12% at 12 months for the intervention group and 9% at 6 month and 22% at 12 month in the control group |
|  | 3.2 If N/PN/NI to 3.1: Is there evidence that result was not biased by missing outcome data? | | | PN | Reasons are very similar for missing outcome data at 6 months, apart from 1 more died and 1 more were hospitalized in the control group. |
|  | 3.3 If N/PN to 3.2: Could missingness in the outcome depend on its true value? | | | PY |  |
|  | 3.4 If Y/PY/NI to 3.3: Is it likely that missingness in the outcome depended on its true value? | | | PN |  |
|  | Risk of bias judgement | | | Some concerns | 5% missing outcome data at 6 month and 12% at 12 months for the intervention group and 9% at 6 month and 22% at 12 month in the control groupStated reasons are very similar for missing outcome data at 6 months, apart from 1 more died and 1 more were hospitalized in the control group which potentially could influence the outcomes to some degree. |
| Bias in measurement of the outcome | 4.1 Was the method of measuring the outcome inappropriate? | | | PN | Question about walking outside would be an appropriate method to use. |
|  | 4.2 Could measurement or ascertainment of the outcome have differed between intervention groups? | | | PN | No indication that measurements of the outcome differed between the groups. |
|  | 4.3 Were outcome assessors aware of the intervention received by study participants? | | | Y | Outcome assessors (participants) were aware of the intervention received. |
|  | 4.4 If Y/PY/NI to 4.3: Could assessment of the outcome have been influenced by knowledge of intervention received? | | | PY | It is likely that participants in an intense rehabilitation intervention would judge their outcomes to be better just because of attending the intervention, compared to an intervention group receiving no intervention which might also be disapointed or likely to judge themselves to be mobile outdoors. |
|  | 4.5 If Y/PY/NI to 4.4: Is it likely that assessment of the outcome was influenced by knowledge of intervention received? | | | PY |  |
|  | Risk of bias judgement | | | High | Question about walking outside would be an appropriate method to use.  No indication that measurements of the outcome differed between the groups.  Outcome assessors (participants) were aware of the intervention received. It is likely that participants in an intense rehabilitation intervention would judge their outcomes to be better just because of attending the intervention, compared to an intervention group receiving no intervention which might also be disapointed or likely to judge themselves to be mobile outdoors. |
| Bias in selection of the reported result | 5.1 Were the data that produced this result analysed in accordance with a pre-specified analysis plan that was finalized before unblinded outcome data were available for analysis? | | | Y | A pre-specified protocol is available. |
|  | 5.2 ... multiple eligible outcome measurements (e.g. scales, definitions, time points) within the outcome domain? | | | PY | Protocol states that "Life-space assessment" and "How would you describe your mobility" would be used, but results are repored only for "difficulties walking outside" which is not specified in the protocol. Potential for selective reporting. |
|  | 5.3 ... multiple eligible analyses of the data? | | |  | Analyses used are seem to be conducted according to the protocol (GEE-models) |
|  | Risk of bias judgement | | | High | A pre-specified protocol is available. Protocol states that "Life-space assessment" and "How would you describe your mobility" would be used, but results are repored only for "difficulties walking outside" which is not specified in the protocol. Potential for selective reporting.  Analyses used are seem to be conducted according to the protocol (GEE-models) |
| Overall bias | Risk of bias judgement | | | High | Overall high risk of bias due to non-blinding and use of a usual care control group, measurement of the outcome and potential for selective reporting. |
|  |  |  |  |  |  |
|  |  |  |  |  |  |
| Unique ID | Turunen 2020 Engagement in everyday life activities 7 months | Study ID | Turunen 2020 | Assessor | MR |
| Ref or Label |  | Aim | assignment to intervention (the 'intention-to-treat' effect) |  |  |
| Experimental |  | Comparator |  | Source | Journal article(s) |
| Outcome | Engagement in everyday life activities 7 months | Results |  | Weight | 1 |
| Domain | Signalling question | | | Response | Comments |
| Bias arising from the randomization process | 1.1 Was the allocation sequence random? | | | PY | Computer generated random sequence using blocks of 10 and stratfied by gender, age and gait-speed was conducted after baseline assessments by an external statistician and in sealed opaque envelopes. No other detailed descriptions of methods of allocation consealment is given. |
|  | 1.2 Was the allocation sequence concealed until participants were enrolled and assigned to interventions? | | | PY |  |
|  | 1.3 Did baseline differences between intervention groups suggest a problem with the randomization process? | | | N | No substancial differences in baseline characteristcs. |
|  | Risk of bias judgement | | | Low | Computer generated random sequence using blocks of 10, conducted after baseline assessments by an external statistician and in sealed opaque envelopes. No other detailed descriptions of methods of allocation consealment is given.  No substancial differences in baseline characteristcs. |
| Bias due to deviations from intended interventions | 2.1.Were participants aware of their assigned intervention during the trial? | | | Y | Participants and treatment providers were aware of their group allocation. |
|  | 2.2.Were carers and people delivering the interventions aware of participants' assigned intervention during the trial? | | | Y |  |
|  | 2.3. If Y/PY/NI to 2.1 or 2.2: Were there deviations from the intended intervention that arose because of the experimental context? | | | PY | Drop-out analysis was done for physiotherapy-visits outside of the intervention which showed no difference. No other information is reported about any potential deviations, but it is likely that the usual-care group would behave differently when they are enrolled in an intervention to improve physical activity and outdoor mobility and is not given any intervention. The intervention group would probably also be reminded more often to report and remember outdoor mobility. |
|  | 2.4 If Y/PY to 2.3: Were these deviations likely to have affected the outcome? | | | PY | It is likely that these deviations could influence the outcome through other aspects than visiting physiotherapists during the study period. |
|  | 2.5. If Y/PY/NI to 2.4: Were these deviations from intended intervention balanced between groups? | | | PN | The deviations would probably not be balaced between the groups. |
|  | 2.6 Was an appropriate analysis used to estimate the effect of assignment to intervention? | | | PY | Analysed according to ITT. |
|  | 2.7 If N/PN/NI to 2.6: Was there potential for a substantial impact (on the result) of the failure to analyse participants in the group to which they were randomized? | | | NA |  |
|  | Risk of bias judgement | | | High | Participants and treatment providers were aware of their group allocation.  Drop-out analysis was done for physiotherapy-visits outside of the intervention which showed no difference. No other information is reported about any potential deviations, but it is likely that the usual-care group would behave differently when they are enrolled in an intervention to improve physical activity and outdoor mobility and is not given any intervention. The intervention group would probably also be reminded more often to report and remember outdoor mobility. It is likely that these deviations could influence the outcome through other aspects than visiting physiotherapists during the study period.  The deviations would probably not be balaced between the groups. Analysed according to ITT. |
| Bias due to missing outcome data | 3.1 Were data for this outcome available for all, or nearly all, participants randomized? | | | PN | 5% missing outcome data at 6 month and 12% at 12 months for the intervention group and 9% at 6 month and 22% at 12 month in the control group |
|  | 3.2 If N/PN/NI to 3.1: Is there evidence that result was not biased by missing outcome data? | | | PN | Differences in missing outcome data is unknown at 12 months, and there are differences between the groups. |
|  | 3.3 If N/PN to 3.2: Could missingness in the outcome depend on its true value? | | | PY | It is likely that the larger proportion of participants leaving the control arm would have different outcomes than the rest of the groups. |
|  | 3.4 If Y/PY/NI to 3.3: Is it likely that missingness in the outcome depended on its true value? | | | PY |  |
|  | Risk of bias judgement | | | High | 5% missing outcome data at 6 month and 12% at 12 months for the intervention group and 9% at 6 month and 22% at 12 month in the control group. Differences in missing outcome data is unknown at 12 months, and there are differences between the groups.  It is likely that the larger proportion of participants leaving the control arm would have different outcomes than the rest of the groups. |
| Bias in measurement of the outcome | 4.1 Was the method of measuring the outcome inappropriate? | | | PN | Question about walking outside would be an appropriate method to use. |
|  | 4.2 Could measurement or ascertainment of the outcome have differed between intervention groups? | | | PN | No indication that measurements of the outcome differed between the groups. |
|  | 4.3 Were outcome assessors aware of the intervention received by study participants? | | | Y | Outcome assessors (participants) were aware of the intervention received. |
|  | 4.4 If Y/PY/NI to 4.3: Could assessment of the outcome have been influenced by knowledge of intervention received? | | | PY | It is likely that participants in an intense rehabilitation intervention would judge their outcomes to be better just because of attending the intervention, compared to an intervention group receiving no intervention which might also be disapointed or likely to judge themselves to be mobile outdoors. |
|  | 4.5 If Y/PY/NI to 4.4: Is it likely that assessment of the outcome was influenced by knowledge of intervention received? | | | PY |  |
|  | Risk of bias judgement | | | High | Question about walking outside would be an appropriate method to use.  No indication that measurements of the outcome differed between the groups.  Outcome assessors (participants) were aware of the intervention received. It is likely that participants in an intense rehabilitation intervention would judge their outcomes to be better just because of attending the intervention, compared to an intervention group receiving no intervention which might also be disapointed or likely to judge themselves to be mobile outdoors. |
| Bias in selection of the reported result | 5.1 Were the data that produced this result analysed in accordance with a pre-specified analysis plan that was finalized before unblinded outcome data were available for analysis? | | | Y | A pre-specified protocol is available. |
|  | 5.2 ... multiple eligible outcome measurements (e.g. scales, definitions, time points) within the outcome domain? | | | PY | Protocol states that "Life-space assessment" and "How would you describe your mobility" would be used, but results are repored only for "difficulties walking outside" which is not specified in the protocol. Potential for selective reporting. |
|  | 5.3 ... multiple eligible analyses of the data? | | |  | Analyses used are seem to be conducted according to the protocol (GEE-models) |
|  | Risk of bias judgement | | | High | A pre-specified protocol is available. Protocol states that "Life-space assessment" and "How would you describe your mobility" would be used, but results are repored only for "difficulties walking outside" which is not specified in the protocol. Potential for selective reporting.  Analyses used are seem to be conducted according to the protocol (GEE-models) |
| Overall bias | Risk of bias judgement | | | High | Overall high risk of bias due to non-blinding and use of a usual care control group, measurement of the outcome and potential for selective reporting. |
|  |  |  |  |  |  |
|  |  |  |  |  |  |
| Unique ID | Turunen 2020 Minor adverse events 6 months | Study ID | Turunen 2020 | Assessor | MR |
| Ref or Label |  | Aim | assignment to intervention (the 'intention-to-treat' effect) |  |  |
| Experimental |  | Comparator |  | Source | Journal article(s) |
| Outcome | Minor adverse events 6 months | Results |  | Weight | 1 |
| Domain | Signalling question | | | Response | Comments |
| Bias arising from the randomization process | 1.1 Was the allocation sequence random? | | | PY | Computer generated random sequence using blocks of 10 and stratfied by gender, age and gait-speed was conducted after baseline assessments by an external statistician and in sealed opaque envelopes. No other detailed descriptions of methods of allocation consealment is given. |
|  | 1.2 Was the allocation sequence concealed until participants were enrolled and assigned to interventions? | | | PY |  |
|  | 1.3 Did baseline differences between intervention groups suggest a problem with the randomization process? | | | N | No substancial differences in baseline characteristcs. |
|  | Risk of bias judgement | | | Low | Computer generated random sequence using blocks of 10, conducted after baseline assessments by an external statistician and in sealed opaque envelopes. No other detailed descriptions of methods of allocation consealment is given.  No substancial differences in baseline characteristcs. |
| Bias due to deviations from intended interventions | 2.1.Were participants aware of their assigned intervention during the trial? | | | Y | Participants and treatment providers were aware of their group allocation. |
|  | 2.2.Were carers and people delivering the interventions aware of participants' assigned intervention during the trial? | | | Y |  |
|  | 2.3. If Y/PY/NI to 2.1 or 2.2: Were there deviations from the intended intervention that arose because of the experimental context? | | | PY | Drop-out analysis was done for physiotherapy-visits outside of the intervention which showed no difference. No other information is reported about any potential deviations, but it is likely that the usual-care group would behave differently when they are enrolled in an intervention to improve physical activity and outdoor mobility and is not given any intervention. The intervention group would probably also be reminded more often to report and remember outdoor mobility. |
|  | 2.4 If Y/PY to 2.3: Were these deviations likely to have affected the outcome? | | | PY | It is likely that these deviations could influence the outcome through other aspects than visiting physiotherapists during the study period, e.g. seeking other medical care. |
|  | 2.5. If Y/PY/NI to 2.4: Were these deviations from intended intervention balanced between groups? | | | PN | The deviations would probably not be balaced between the groups. |
|  | 2.6 Was an appropriate analysis used to estimate the effect of assignment to intervention? | | | PY | Analysed according to ITT. |
|  | 2.7 If N/PN/NI to 2.6: Was there potential for a substantial impact (on the result) of the failure to analyse participants in the group to which they were randomized? | | | NA |  |
|  | Risk of bias judgement | | | High | Participants and treatment providers were aware of their group allocation.  Drop-out analysis was done for physiotherapy-visits outside of the intervention which showed no difference. No other information is reported about any potential deviations, but it is likely that the usual-care group would behave differently when they are enrolled in an intervention to improve physical activity and outdoor mobility and is not given any intervention. The intervention group would probably also be reminded more often to report and remember outdoor mobility. It is likely that these deviations could influence the outcome through other aspects than visiting physiotherapists during the study period, e.g. seeking other medical care.  The deviations would probably not be balaced between the groups.  Analysed according to ITT. |
| Bias due to missing outcome data | 3.1 Were data for this outcome available for all, or nearly all, participants randomized? | | | PN | 5% missing outcome data at 6 month and 12% at 12 months for the intervention group and 9% at 6 month and 22% at 12 month in the control group |
|  | 3.2 If N/PN/NI to 3.1: Is there evidence that result was not biased by missing outcome data? | | | PN | Differences in missing outcome data is unknown at 12 months, and there are differences between the groups. |
|  | 3.3 If N/PN to 3.2: Could missingness in the outcome depend on its true value? | | | PY | It is likely that the larger proportion of participants leaving the control arm would have different outcomes than the rest of the groups. |
|  | 3.4 If Y/PY/NI to 3.3: Is it likely that missingness in the outcome depended on its true value? | | | PY |  |
|  | Risk of bias judgement | | | High | 5% missing outcome data at 6 month and 12% at 12 months for the intervention group and 9% at 6 month and 22% at 12 month in the control group. Differences in missing outcome data is unknown at 12 months, and there are differences between the groups.  It is likely that the larger proportion of participants leaving the control arm would have different outcomes than the rest of the groups. |
| Bias in measurement of the outcome | 4.1 Was the method of measuring the outcome inappropriate? | | | PN | Question about healthcare consumption is a possible way to measure harms. |
|  | 4.2 Could measurement or ascertainment of the outcome have differed between intervention groups? | | | PN | No indication that measurements of the outcome differed between the groups. |
|  | 4.3 Were outcome assessors aware of the intervention received by study participants? | | | Y | Outcome assessors (participants) were aware of the intervention received. |
|  | 4.4 If Y/PY/NI to 4.3: Could assessment of the outcome have been influenced by knowledge of intervention received? | | | PY | It is likely that participants in an intense rehabilitation intervention would judge their outcomes to be better just because of attending the intervention, compared to an intervention group receiving no intervention which might also be disapointed or likely to judge themselves to have different levels of adverse events. |
|  | 4.5 If Y/PY/NI to 4.4: Is it likely that assessment of the outcome was influenced by knowledge of intervention received? | | | PY |  |
|  | Risk of bias judgement | | | High | Question about healthcare consumption is a possible way to measure harms.  No indication that measurements of the outcome differed between the groups.  Outcome assessors (participants) were aware of the intervention received. It is likely that participants in an intense rehabilitation intervention would judge their outcomes to be better just because of attending the intervention, compared to an intervention group receiving no intervention which might also be disapointed or likely to judge themselves to have different levels of adverse events. |
| Bias in selection of the reported result | 5.1 Were the data that produced this result analysed in accordance with a pre-specified analysis plan that was finalized before unblinded outcome data were available for analysis? | | | Y | A pre-specified protocol is available. |
|  | 5.2 ... multiple eligible outcome measurements (e.g. scales, definitions, time points) within the outcome domain? | | | PY | Protocol states that use of healthcare will be collected, but nothing more. |
|  | 5.3 ... multiple eligible analyses of the data? | | | PN | Analyses used are seem to be conducted according to the protocol (GEE-models) |
|  | Risk of bias judgement | | | High | A pre-specified protocol is available.  Protocol states that use of healthcare will be collected, but nothing more.  Analyses used are seem to be conducted according to the protocol (GEE-models) |
| Overall bias | Risk of bias judgement | | | High | Overall high risk of bias due to non-blinding and use of a usual care control group, measurement of the outcome and potential for selective reporting. |
|  |  |  |  |  |  |
|  |  |  |  |  |  |
| Unique ID | Turunen 2020 Minor adverse events 7 months | Study ID | Turunen 2020 | Assessor | MR |
| Ref or Label |  | Aim | assignment to intervention (the 'intention-to-treat' effect) |  |  |
| Experimental |  | Comparator |  | Source | Journal article(s) |
| Outcome | Minor adverse events 7 months | Results |  | Weight | 1 |
| Domain | Signalling question | | | Response | Comments |
| Bias arising from the randomization process | 1.1 Was the allocation sequence random? | | | PY | Computer generated random sequence using blocks of 10 and stratfied by gender, age and gait-speed was conducted after baseline assessments by an external statistician and in sealed opaque envelopes. No other detailed descriptions of methods of allocation consealment is given. |
|  | 1.2 Was the allocation sequence concealed until participants were enrolled and assigned to interventions? | | | PY |  |
|  | 1.3 Did baseline differences between intervention groups suggest a problem with the randomization process? | | | N | No substancial differences in baseline characteristcs. |
|  | Risk of bias judgement | | | Low | Computer generated random sequence using blocks of 10, conducted after baseline assessments by an external statistician and in sealed opaque envelopes. No other detailed descriptions of methods of allocation consealment is given.  No substancial differences in baseline characteristcs. |
| Bias due to deviations from intended interventions | 2.1.Were participants aware of their assigned intervention during the trial? | | | Y | Participants and treatment providers were aware of their group allocation. |
|  | 2.2.Were carers and people delivering the interventions aware of participants' assigned intervention during the trial? | | | Y |  |
|  | 2.3. If Y/PY/NI to 2.1 or 2.2: Were there deviations from the intended intervention that arose because of the experimental context? | | | PY | Drop-out analysis was done for physiotherapy-visits outside of the intervention which showed no difference. No other information is reported about any potential deviations, but it is likely that the usual-care group would behave differently when they are enrolled in an intervention to improve physical activity and outdoor mobility and is not given any intervention. The intervention group would probably also be reminded more often to report and remember outdoor mobility. |
|  | 2.4 If Y/PY to 2.3: Were these deviations likely to have affected the outcome? | | | PY | It is likely that these deviations could influence the outcome through other aspects than visiting physiotherapists during the study period. |
|  | 2.5. If Y/PY/NI to 2.4: Were these deviations from intended intervention balanced between groups? | | | PN | The deviations would probably not be balaced between the groups. |
|  | 2.6 Was an appropriate analysis used to estimate the effect of assignment to intervention? | | | PY | Analysed according to ITT. |
|  | 2.7 If N/PN/NI to 2.6: Was there potential for a substantial impact (on the result) of the failure to analyse participants in the group to which they were randomized? | | | NA |  |
|  | Risk of bias judgement | | | High | Participants and treatment providers were aware of their group allocation.  Drop-out analysis was done for physiotherapy-visits outside of the intervention which showed no difference. No other information is reported about any potential deviations, but it is likely that the usual-care group would behave differently when they are enrolled in an intervention to improve physical activity and outdoor mobility and is not given any intervention. The intervention group would probably also be reminded more often to report and remember outdoor mobility. It is likely that these deviations could influence the outcome through other aspects than visiting physiotherapists during the study period.  The deviations would probably not be balaced between the groups. Analysed according to ITT. |
| Bias due to missing outcome data | 3.1 Were data for this outcome available for all, or nearly all, participants randomized? | | | PN | 5% missing outcome data at 6 month and 12% at 12 months for the intervention group and 9% at 6 month and 22% at 12 month in the control group |
|  | 3.2 If N/PN/NI to 3.1: Is there evidence that result was not biased by missing outcome data? | | | PN | Differences in missing outcome data is unknown at 12 months, and there are differences between the groups. |
|  | 3.3 If N/PN to 3.2: Could missingness in the outcome depend on its true value? | | | PY | It is likely that the larger proportion of participants leaving the control arm would have different outcomes than the rest of the groups. |
|  | 3.4 If Y/PY/NI to 3.3: Is it likely that missingness in the outcome depended on its true value? | | | PY |  |
|  | Risk of bias judgement | | | High | 5% missing outcome data at 6 month and 12% at 12 months for the intervention group and 9% at 6 month and 22% at 12 month in the control group. Differences in missing outcome data is unknown at 12 months, and there are differences between the groups.  It is likely that the larger proportion of participants leaving the control arm would have different outcomes than the rest of the groups. |
| Bias in measurement of the outcome | 4.1 Was the method of measuring the outcome inappropriate? | | | PN | Question about walking outside would be an appropriate method to use. |
|  | 4.2 Could measurement or ascertainment of the outcome have differed between intervention groups? | | | PN | No indication that measurements of the outcome differed between the groups. |
|  | 4.3 Were outcome assessors aware of the intervention received by study participants? | | | Y | Outcome assessors (participants) were aware of the intervention received. |
|  | 4.4 If Y/PY/NI to 4.3: Could assessment of the outcome have been influenced by knowledge of intervention received? | | | PY | It is likely that participants in an intense rehabilitation intervention would judge their outcomes to be better just because of attending the intervention, compared to an intervention group receiving no intervention which might also be disapointed or likely to judge themselves to be mobile outdoors. |
|  | 4.5 If Y/PY/NI to 4.4: Is it likely that assessment of the outcome was influenced by knowledge of intervention received? | | | PY |  |
|  | Risk of bias judgement | | | High | Question about healthcare consumption is a possible way to measure harms.  No indication that measurements of the outcome differed between the groups.  Outcome assessors (participants) were aware of the intervention received. It is likely that participants in an intense rehabilitation intervention would judge their outcomes to be better just because of attending the intervention, compared to an intervention group receiving no intervention which might also be disapointed or likely to judge themselves to have different levels of adverse events. |
| Bias in selection of the reported result | 5.1 Were the data that produced this result analysed in accordance with a pre-specified analysis plan that was finalized before unblinded outcome data were available for analysis? | | | Y | A pre-specified protocol is available. |
|  | 5.2 ... multiple eligible outcome measurements (e.g. scales, definitions, time points) within the outcome domain? | | | PY | Protocol states that use of healthcare will be collected, but nothing more. |
|  | 5.3 ... multiple eligible analyses of the data? | | | PN | Analyses used are seem to be conducted according to the protocol (GEE-models) |
|  | Risk of bias judgement | | | High | A pre-specified protocol is available.  Protocol states that use of healthcare will be collected, but nothing more.  Analyses used are seem to be conducted according to the protocol (GEE-models) |
| Overall bias | Risk of bias judgement | | | High | Overall high risk of bias due to non-blinding and use of a usual care control group, measurement of the outcome and potential for selective reporting. |
|  |  |  |  |  |  |
|  |  |  |  |  |  |
| Unique ID | Turunen 2020 Major adverse events 6 months | Study ID | Turunen 2020 | Assessor | MR |
| Ref or Label |  | Aim | assignment to intervention (the 'intention-to-treat' effect) |  |  |
| Experimental |  | Comparator |  | Source | Journal article(s) |
| Outcome | Major adverse events 6 months | Results |  | Weight | 1 |
| Domain | Signalling question | | | Response | Comments |
| Bias arising from the randomization process | 1.1 Was the allocation sequence random? | | | PY | Computer generated random sequence using blocks of 10 and stratfied by gender, age and gait-speed was conducted after baseline assessments by an external statistician and in sealed opaque envelopes. No other detailed descriptions of methods of allocation consealment is given. |
|  | 1.2 Was the allocation sequence concealed until participants were enrolled and assigned to interventions? | | | PY |  |
|  | 1.3 Did baseline differences between intervention groups suggest a problem with the randomization process? | | | N | No substancial differences in baseline characteristcs. |
|  | Risk of bias judgement | | | Low | Computer generated random sequence using blocks of 10, conducted after baseline assessments by an external statistician and in sealed opaque envelopes. No other detailed descriptions of methods of allocation consealment is given.  No substancial differences in baseline characteristcs. |
| Bias due to deviations from intended interventions | 2.1.Were participants aware of their assigned intervention during the trial? | | | Y | Participants and treatment providers were aware of their group allocation. |
|  | 2.2.Were carers and people delivering the interventions aware of participants' assigned intervention during the trial? | | | Y |  |
|  | 2.3. If Y/PY/NI to 2.1 or 2.2: Were there deviations from the intended intervention that arose because of the experimental context? | | | PY | Drop-out analysis was done for physiotherapy-visits outside of the intervention which showed no difference. No other information is reported about any potential deviations, but it is likely that the usual-care group would behave differently when they are enrolled in an intervention to improve physical activity and outdoor mobility and is not given any intervention. The intervention group would probably also be reminded more often to report and remember outdoor mobility. |
|  | 2.4 If Y/PY to 2.3: Were these deviations likely to have affected the outcome? | | | PY | It is likely that these deviations could influence the outcome through other aspects than visiting physiotherapists during the study period. |
|  | 2.5. If Y/PY/NI to 2.4: Were these deviations from intended intervention balanced between groups? | | | It is unlikely that these deviations could influence the outcome of major adverse events through other aspects than visiting physiotherapists during the study period. | NA |
|  | 2.6 Was an appropriate analysis used to estimate the effect of assignment to intervention? | | |  | PY |
|  | 2.7 If N/PN/NI to 2.6: Was there potential for a substantial impact (on the result) of the failure to analyse participants in the group to which they were randomized? | | | Analysed according to ITT. | NA |
|  | Risk of bias judgement | | | Some concerns | Some concerns |
| Bias due to missing outcome data | 3.1 Were data for this outcome available for all, or nearly all, participants randomized? | | |  | 5% missing outcome data at 6 month and 12% at 12 months for the intervention group and 9% at 6 month and 22% at 12 month in the control group |
|  | 3.2 If N/PN/NI to 3.1: Is there evidence that result was not biased by missing outcome data? | | | PN | Differences in missing outcome data is unknown at 12 months, and there are differences between the groups. |
|  | 3.3 If N/PN to 3.2: Could missingness in the outcome depend on its true value? | | | PY | It is likely that the larger proportion of participants leaving the control arm would have different outcomes than the rest of the groups. |
|  | 3.4 If Y/PY/NI to 3.3: Is it likely that missingness in the outcome depended on its true value? | | | PY |  |
|  | Risk of bias judgement | | | High | 5% missing outcome data at 6 month and 12% at 12 months for the intervention group and 9% at 6 month and 22% at 12 month in the control group. Differences in missing outcome data is unknown at 12 months, and there are differences between the groups.  It is likely that the larger proportion of participants leaving the control arm would have different outcomes than the rest of the groups. |
| Bias in measurement of the outcome | 4.1 Was the method of measuring the outcome inappropriate? | | | PN | Question about walking outside would be an appropriate method to use. |
|  | 4.2 Could measurement or ascertainment of the outcome have differed between intervention groups? | | | PN | No indication that measurements of the outcome differed between the groups. |
|  | 4.3 Were outcome assessors aware of the intervention received by study participants? | | | Y | Outcome assessors (participants) were aware of the intervention received. |
|  | 4.4 If Y/PY/NI to 4.3: Could assessment of the outcome have been influenced by knowledge of intervention received? | | | PY | It is likely that participants in an intense rehabilitation intervention would judge their outcomes to be better just because of attending the intervention, compared to an intervention group receiving no intervention which might also be disapointed or likely to judge themselves to be mobile outdoors. |
|  | 4.5 If Y/PY/NI to 4.4: Is it likely that assessment of the outcome was influenced by knowledge of intervention received? | | | PY |  |
|  | Risk of bias judgement | | | High | Question about healthcare consumption is a possible way to measure harms.  No indication that measurements of the outcome differed between the groups.  Outcome assessors (participants) were aware of the intervention received. It is likely that participants in an intense rehabilitation intervention would judge their outcomes to be better just because of attending the intervention, compared to an intervention group receiving no intervention which might also be disapointed or likely to judge themselves to have different levels of adverse events. |
| Bias in selection of the reported result | 5.1 Were the data that produced this result analysed in accordance with a pre-specified analysis plan that was finalized before unblinded outcome data were available for analysis? | | | Y | A pre-specified protocol is available. |
|  | 5.2 ... multiple eligible outcome measurements (e.g. scales, definitions, time points) within the outcome domain? | | | PY | Protocol states that use of healthcare will be collected, but nothing more. |
|  | 5.3 ... multiple eligible analyses of the data? | | | PN | Analyses used are seem to be conducted according to the protocol (GEE-models) |
|  | Risk of bias judgement | | | High | A pre-specified protocol is available.  Protocol states that use of healthcare will be collected, but nothing more.  Analyses used are seem to be conducted according to the protocol (GEE-models) |
| Overall bias | Risk of bias judgement | | | High | Overall high risk of bias due to non-blinding and use of a usual care control group, measurement of the outcome and potential for selective reporting. |
|  |  |  |  |  |  |
|  |  |  |  |  |  |
| Unique ID | Turunen 2020 Major adverse events 7 months | Study ID | Turunen 2020 | Assessor | MR |
| Ref or Label |  | Aim | assignment to intervention (the 'intention-to-treat' effect) |  |  |
| Experimental |  | Comparator |  | Source | Journal article(s) |
| Outcome | Major adverse events 7 months | Results |  | Weight | 1 |
| Domain | Signalling question | | | Response | Comments |
| Bias arising from the randomization process | 1.1 Was the allocation sequence random? | | | PY | Computer generated random sequence using blocks of 10 and stratfied by gender, age and gait-speed was conducted after baseline assessments by an external statistician and in sealed opaque envelopes. No other detailed descriptions of methods of allocation consealment is given. |
|  | 1.2 Was the allocation sequence concealed until participants were enrolled and assigned to interventions? | | | PY |  |
|  | 1.3 Did baseline differences between intervention groups suggest a problem with the randomization process? | | | N | No substancial differences in baseline characteristcs. |
|  | Risk of bias judgement | | | Low | Computer generated random sequence using blocks of 10, conducted after baseline assessments by an external statistician and in sealed opaque envelopes. No other detailed descriptions of methods of allocation consealment is given.  No substancial differences in baseline characteristcs. |
| Bias due to deviations from intended interventions | 2.1.Were participants aware of their assigned intervention during the trial? | | | Y | Participants and treatment providers were aware of their group allocation. |
|  | 2.2.Were carers and people delivering the interventions aware of participants' assigned intervention during the trial? | | | Y |  |
|  | 2.3. If Y/PY/NI to 2.1 or 2.2: Were there deviations from the intended intervention that arose because of the experimental context? | | | PY | Drop-out analysis was done for physiotherapy-visits outside of the intervention which showed no difference. No other information is reported about any potential deviations, but it is likely that the usual-care group would behave differently when they are enrolled in an intervention to improve physical activity and outdoor mobility and is not given any intervention. The intervention group would probably also be reminded more often to report and remember outdoor mobility. |
|  | 2.4 If Y/PY to 2.3: Were these deviations likely to have affected the outcome? | | | PN | It is unlikely that these deviations could influence the outcome of major adverse events through other aspects than visiting physiotherapists during the study period. |
|  | 2.5. If Y/PY/NI to 2.4: Were these deviations from intended intervention balanced between groups? | | | NA |  |
|  | 2.6 Was an appropriate analysis used to estimate the effect of assignment to intervention? | | | PY | Analysed according to ITT. |
|  | 2.7 If N/PN/NI to 2.6: Was there potential for a substantial impact (on the result) of the failure to analyse participants in the group to which they were randomized? | | | NA |  |
|  | Risk of bias judgement | | | Some concerns | Participants and treatment providers were aware of their group allocation.  Drop-out analysis was done for physiotherapy-visits outside of the intervention which showed no difference. No other information is reported about any potential deviations, but it is likely that the usual-care group would behave differently when they are enrolled in an intervention to improve physical activity and outdoor mobility and is not given any intervention. The intervention group would probably also be reminded more often to report and remember outdoor mobility. It is unlikely that these deviations could influence the outcome of major adverse events through other aspects than visiting physiotherapists during the study period.   Analysed according to ITT. |
| Bias due to missing outcome data | 3.1 Were data for this outcome available for all, or nearly all, participants randomized? | | | PN | 5% missing outcome data at 6 month and 12% at 12 months for the intervention group and 9% at 6 month and 22% at 12 month in the control group |
|  | 3.2 If N/PN/NI to 3.1: Is there evidence that result was not biased by missing outcome data? | | | PN | Differences in missing outcome data is unknown at 12 months, and there are differences between the groups. |
|  | 3.3 If N/PN to 3.2: Could missingness in the outcome depend on its true value? | | | PY | It is likely that the larger proportion of participants leaving the control arm would have different outcomes than the rest of the groups. |
|  | 3.4 If Y/PY/NI to 3.3: Is it likely that missingness in the outcome depended on its true value? | | | PY |  |
|  | Risk of bias judgement | | | High | 5% missing outcome data at 6 month and 12% at 12 months for the intervention group and 9% at 6 month and 22% at 12 month in the control group. Differences in missing outcome data is unknown at 12 months, and there are differences between the groups.  It is likely that the larger proportion of participants leaving the control arm would have different outcomes than the rest of the groups. |
| Bias in measurement of the outcome | 4.1 Was the method of measuring the outcome inappropriate? | | | PN | Question about walking outside would be an appropriate method to use. |
|  | 4.2 Could measurement or ascertainment of the outcome have differed between intervention groups? | | | PN | No indication that measurements of the outcome differed between the groups. |
|  | 4.3 Were outcome assessors aware of the intervention received by study participants? | | | Y | Outcome assessors (participants) were aware of the intervention received. |
|  | 4.4 If Y/PY/NI to 4.3: Could assessment of the outcome have been influenced by knowledge of intervention received? | | | PY | It is likely that participants in an intense rehabilitation intervention would judge their outcomes to be better just because of attending the intervention, compared to an intervention group receiving no intervention which might also be disapointed or likely to judge themselves to be mobile outdoors. |
|  | 4.5 If Y/PY/NI to 4.4: Is it likely that assessment of the outcome was influenced by knowledge of intervention received? | | | PY |  |
|  | Risk of bias judgement | | | High | Question about healthcare consumption is a possible way to measure harms.  No indication that measurements of the outcome differed between the groups.  Outcome assessors (participants) were aware of the intervention received. It is likely that participants in an intense rehabilitation intervention would judge their outcomes to be better just because of attending the intervention, compared to an intervention group receiving no intervention which might also be disapointed or likely to judge themselves to have different levels of adverse events. |
| Bias in selection of the reported result | 5.1 Were the data that produced this result analysed in accordance with a pre-specified analysis plan that was finalized before unblinded outcome data were available for analysis? | | | Y | A pre-specified protocol is available. |
|  | 5.2 ... multiple eligible outcome measurements (e.g. scales, definitions, time points) within the outcome domain? | | | PY | Protocol states that use of healthcare will be collected, but nothing more. |
|  | 5.3 ... multiple eligible analyses of the data? | | | PN | Analyses used are seem to be conducted according to the protocol (GEE-models) |
|  | Risk of bias judgement | | | High | A pre-specified protocol is available.  Protocol states that use of healthcare will be collected, but nothing more.  Analyses used are seem to be conducted according to the protocol (GEE-models) |
| Overall bias | Risk of bias judgement | | | High | Overall high risk of bias due to non-blinding and use of a usual care control group, measurement of the outcome and potential for selective reporting. |
|  |  |  |  |  |  |
|  |  |  |  |  |  |
| Unique ID | Rantanen 2015 Health-related quality of life 6 months | Study ID | Rantanen 2015 | Assessor | MR |
| Ref or Label |  | Aim | assignment to intervention (the 'intention-to-treat' effect) |  |  |
| Experimental |  | Comparator |  | Source | Journal article(s) |
| Outcome | Health-related quality of life 6 months | Results |  | Weight | 1 |
| Domain | Signalling question | | | Response | Comments |
| Bias arising from the randomization process | 1.1 Was the allocation sequence random? | | | Y | Randomsation with a computer-generated list after baseline assessment by a statistican not involved in the study. |
|  | 1.2 Was the allocation sequence concealed until participants were enrolled and assigned to interventions? | | | NI |  |
|  | 1.3 Did baseline differences between intervention groups suggest a problem with the randomization process? | | | PY | Some baseline differences in prognostic variables are present that favors the intervention group. Control group has 46% vs 23% in poor self-rated health, 21% vs 13% in self-rated loneliness and 57% vs 44% in self-rated difficulties in using public transport. |
|  | Risk of bias judgement | | | High | Randomsation with a computer-generated list after baseline assessment by a statistican not involved in the study Baseline-differences, in particular for baseline QoL and prognostic factors for OM are present indicates a problem. |
| Bias due to deviations from intended interventions | 2.1.Were participants aware of their assigned intervention during the trial? | | | Y | Participants and treatment providers were aware of the assigned intervention. |
|  | 2.2.Were carers and people delivering the interventions aware of participants' assigned intervention during the trial? | | | Y |  |
|  | 2.3. If Y/PY/NI to 2.1 or 2.2: Were there deviations from the intended intervention that arose because of the experimental context? | | | PY | No deviations are reported, but 8 participants left the wait-list control group compared to 3 in the intervention group which indicates a different behavior between groups based on group allocation, possibly most in the wait-list control. |
|  | 2.4 If Y/PY to 2.3: Were these deviations likely to have affected the outcome? | | | PN | It is possible that these deviations could influence the outcomes, like seeking care or other ways to improve outdoor mobility which could influence quality of life. But this is somewhat less due to the control group knowledge of getting a similar intervention after waiting for 3 months. |
|  | 2.5. If Y/PY/NI to 2.4: Were these deviations from intended intervention balanced between groups? | | | NA |  |
|  | 2.6 Was an appropriate analysis used to estimate the effect of assignment to intervention? | | | PY | Yes, ITT with complete case-analyses. |
|  | 2.7 If N/PN/NI to 2.6: Was there potential for a substantial impact (on the result) of the failure to analyse participants in the group to which they were randomized? | | | NA |  |
|  | Risk of bias judgement | | | Some concerns | Participants and treatment providers were aware of the assigned intervention.  No deviations are reported, but 8 participants left the wait-list control group compared to 3 in the intervention group which indicates a different behavior between groups based on group allocation, possibly most in the wait-list control.  It is possible that these deviations could influence the outcomes, like seeking care or other ways to improve outdoor mobility which could influence quality of life. But this is somewhat less due to the control group knowledge of getting a similar intervention after waiting for 3 months. ITT with complete case-analyses was used. |
| Bias due to missing outcome data | 3.1 Were data for this outcome available for all, or nearly all, participants randomized? | | | N | 6% missing outcome data in intervention group and 13% in the control group at 3 months. |
|  | 3.2 If N/PN/NI to 3.1: Is there evidence that result was not biased by missing outcome data? | | | PN | No attempts were made to assess the impact of missing outcome data, but "expected score" based on baseline values were used for missing outcome data. |
|  | 3.3 If N/PN to 3.2: Could missingness in the outcome depend on its true value? | | | PY | The reason for leaving the study was mostly due to "not willing to continue", which was 8 vs 3 for the comparison group, indicating that people with larger needs and wanting an intervention before the 3 month perioid i in the control group left, which is likely to influence the outcomes. |
|  | 3.4 If Y/PY/NI to 3.3: Is it likely that missingness in the outcome depended on its true value? | | | PN |  |
|  | Risk of bias judgement | | | Some concerns | 6% missing outcome data in intervention group and 13% in the control group at 3 months.  No attempts were made to assess the impact of missing outcome data, but imputations based on baseline-values and expected scores were used that lessens the potential risk of bias . Nhe reason for leaving the study was mostly due to "not willing to continue", which was 8 vs 3 for the comparison group, indicating that people with larger needs and wanting an intervention before the 3 month perioid i in the control group left, which is possibly could influence the outcomes. |
| Bias in measurement of the outcome | 4.1 Was the method of measuring the outcome inappropriate? | | | N | Used a validated instrument. |
|  | 4.2 Could measurement or ascertainment of the outcome have differed between intervention groups? | | | N | No indication of any differences in outcome measurements between the groups. |
|  | 4.3 Were outcome assessors aware of the intervention received by study participants? | | | Y | Participants were aware of their group allocation. |
|  | 4.4 If Y/PY/NI to 4.3: Could assessment of the outcome have been influenced by knowledge of intervention received? | | | PY | It is possible that the jugdement of Quality of life could differ between the groups due to knowledge of getting attention or no attention during the study period. But as the deliverer and helper to fill out the questionnaire was blinded to study allocation and that the participants in the control group were aware that they would get a similar intervention to the intervention group closely after the wait-list-period, this would somewhat lessen the risk of bias in this domain. |
|  | 4.5 If Y/PY/NI to 4.4: Is it likely that assessment of the outcome was influenced by knowledge of intervention received? | | | PY |  |
|  | Risk of bias judgement | | | High | Used a validated instrument.  No indication of any differences in outcome measurements between the groups.  Participants were aware of their group allocation. It is possible that the jugdement of Quality of life could differ between the groups due to knowledge of getting attention or no attention during the study period. But as the deliverer and helper to fill out the questionnaire was blinded to study allocation and that the participants in the control group were aware that they would get a similar intervention to the intervention group closely after the wait-list-period, this somewhat lessen the risk of bias in this domain. |
| Bias in selection of the reported result | 5.1 Were the data that produced this result analysed in accordance with a pre-specified analysis plan that was finalized before unblinded outcome data were available for analysis? | | | PN | Study is stated to be retrospectively registered in ISRCTN-registry. Enrollment started in January 2009, so any trial registration at this point in time would not be very likely. |
|  | 5.2 ... multiple eligible outcome measurements (e.g. scales, definitions, time points) within the outcome domain? | | | PN | Register states that the main outcome are specifically the Social and Environmental domains of the QoL measurement, but all parts and the overall score are presented. But since all data is provided it is unlikely to have been selectively reported. |
|  | 5.3 ... multiple eligible analyses of the data? | | | PN | No specific analyses plan or how outcome data would be handled is presented. |
|  | Risk of bias judgement | | | Some concerns | Study is stated to be retrospectively registered in ISRCTN-registry. Enrollment started in January 2009, so any trial registration at this point in time would not be very likely. Register states that the main outcome are specifically the Social and Environmental domains of the QoL measurement, but all parts and the overall score are presented. But since all data is provided it is unlikely to have been selectively reported. No specific analyses plan or how outcome data would be handled is presented. |
| Overall bias | Risk of bias judgement | | | High | Overall high risk of bias due to missing outcome data and differences between groups, as well as some conserns in the rest of the domains, mainly some baseline differences, non-blinding of participants and using a wait-list control group, and no pre-specified protocol. |
|  |  |  |  |  |  |
|  |  |  |  |  |  |
| Unique ID | Fairhall 2012 Activity outside the home 6 months | Study ID | Fairhall 2012 | Assessor | MR |
| Ref or Label |  | Aim | assignment to intervention (the 'intention-to-treat' effect) |  |  |
| Experimental |  | Comparator |  | Source | Journal article(s) |
| Outcome | Activity outside the home 6 months | Results |  | Weight | 1 |
| Domain | Signalling question | | | Response | Comments |
| Bias arising from the randomization process | 1.1 Was the allocation sequence random? | | | PY | An external data analyst created a computer-generated random numbers sequence using blocks and stratifications which was stored off site. Allocation was consealed until after baseline assessments. |
|  | 1.2 Was the allocation sequence concealed until participants were enrolled and assigned to interventions? | | | PY |  |
|  | 1.3 Did baseline differences between intervention groups suggest a problem with the randomization process? | | | N | No substancial baseline differences are present. |
|  | Risk of bias judgement | | | Low | An external data analyst created a computer-generated random numbers sequence using blocks and stratifications which was stored off site. Allocation was consealed until after baseline assessments.  No substancial baseline differences are present. |
| Bias due to deviations from intended interventions | 2.1.Were participants aware of their assigned intervention during the trial? | | | Y | Participants and treatment providers were aware of the group allocation. |
|  | 2.2.Were carers and people delivering the interventions aware of participants' assigned intervention during the trial? | | | Y |  |
|  | 2.3. If Y/PY/NI to 2.1 or 2.2: Were there deviations from the intended intervention that arose because of the experimental context? | | | PY | The control group got usual care after wanting to be enrolled in a study to improve outdoor mobility. The authors did not assess any deviations, but it is likely that the control group behaved differently due to not recieving any intervention at all. |
|  | 2.4 If Y/PY to 2.3: Were these deviations likely to have affected the outcome? | | | PY | It is likely that deviations (e.g. seeking additional care) could influence the outcomes. |
|  | 2.5. If Y/PY/NI to 2.4: Were these deviations from intended intervention balanced between groups? | | | PN | The deviations were likely not balanced between groups. |
|  | 2.6 Was an appropriate analysis used to estimate the effect of assignment to intervention? | | | PY | Intention to treat-analysis was used. |
|  | 2.7 If N/PN/NI to 2.6: Was there potential for a substantial impact (on the result) of the failure to analyse participants in the group to which they were randomized? | | | NA |  |
|  | Risk of bias judgement | | | High | Participants and treatment providers were aware of the group allocation.  The control group got usual care after wanting to be enrolled in a study to improve outdoor mobility. The authors did not assess any deviations, but it is likely that the control group behaved differently due to not recieving any intervention.  It is likely that deviations (e.g. seeking additional care) could influence the outcomes.  The deviations were likely not balanced between groups. Intention to treat-analysis was used. |
| Bias due to missing outcome data | 3.1 Were data for this outcome available for all, or nearly all, participants randomized? | | | PN | At 3 months the intervention group had 9% missingness of outcome data, and control group 3%. |
|  | 3.2 If N/PN/NI to 3.1: Is there evidence that result was not biased by missing outcome data? | | | PN | Authors do not report any attempt to explore any potential bias by missing outcome data. |
|  | 3.3 If N/PN to 3.2: Could missingness in the outcome depend on its true value? | | | PY | 6 vs 1 in the groups had missing outcome data due to "death", and 3 vs 0 declined to be interviewed - indicating that outcomes was not missing on random at this timepoint, and could influence outcomes (e.g. people who died at 3 months can be assume have less outdoor mobility, QoL and participation which would influence any group-differences). But numbers of missing outcome data are quite small (9 vs 3%) which lessens the potential risk of bias for this domain. |
|  | 3.4 If Y/PY/NI to 3.3: Is it likely that missingness in the outcome depended on its true value? | | | PN |  |
|  | Risk of bias judgement | | | Some concerns | At 3 months the intervention group had 9% missingness of outcome data, and control group 3%.  Authors do not report any attempt to explore any potential bias by missing outcome data.  6 vs 1 in the groups had missing outcome data due to "death", and 3 vs 0 declined to be interviewed - indicating that outcomes was not missing on random at this timepoint, and could influence outcomes (e.g. people who died at 3 months can be assume have less outdoor mobility, QoL and participation which would influence any group-differences). But numbers of missing outcome data are quite small (9 vs 3%) which lessens the potential risk of bias for this domain. |
| Bias in measurement of the outcome | 4.1 Was the method of measuring the outcome inappropriate? | | | PN | A validated intrument was used. |
|  | 4.2 Could measurement or ascertainment of the outcome have differed between intervention groups? | | | PN | Not reported, but unlikely that the outcome collection differered between groups, as the plan was to use blinded outcome assessors. |
|  | 4.3 Were outcome assessors aware of the intervention received by study participants? | | | Y | Participants rating their own outcomes were not blinded, and researchers collecting outcomes were planned to be unblinded but a majority (51%) became unblinded while supporting the participants with the outcome assessments. |
|  | 4.4 If Y/PY/NI to 4.3: Could assessment of the outcome have been influenced by knowledge of intervention received? | | | PY | It is likely that the assessment of the subjectively reported outcomes could differ between the groups due to the design of one group getting a lot of attention and focus on QoL and mobility, and the other did not get any attention at all. |
|  | 4.5 If Y/PY/NI to 4.4: Is it likely that assessment of the outcome was influenced by knowledge of intervention received? | | | PY |  |
|  | Risk of bias judgement | | | High | A validated intrument was used.  Not reported, but unlikely that the outcome collection differered between groups, as the plan was to use blinded outcome assessors.  Participants rating their own outcomes were not blinded, and researchers collecting outcomes were planned to be unblinded but a majority (51%) became unblinded while supporting the participants with the outcome assessments.  It is likely that the assessment of the subjectively reported outcomes could differ between the groups due to the design of one group getting a lot of attention and focus on QoL and mobility, and the other did not get any attention at all. |
| Bias in selection of the reported result | 5.1 Were the data that produced this result analysed in accordance with a pre-specified analysis plan that was finalized before unblinded outcome data were available for analysis? | | | Y | A pre-specified analysis plan was published in 2008. |
|  | 5.2 ... multiple eligible outcome measurements (e.g. scales, definitions, time points) within the outcome domain? | | | N | All outcomes are reported in the protocol. |
|  | 5.3 ... multiple eligible analyses of the data? | | | N | All analyses and justifications for adjustments are reported in the protocol. |
|  | Risk of bias judgement | | | Low | A pre-specified analysis plan was published in 2008.  All outcomes are reported in the protocol.  All analyses and justifications for adjustments are reported in the protocol. |
| Overall bias | Risk of bias judgement | | | High | Overall high risk of bias due to non-blinding of participants and personell and use of a usual care group without any attempt for attention control or assessing any deviations between interventions because of these choices. |
|  |  |  |  |  |  |
|  |  |  |  |  |  |
| Unique ID | Fairhall 2012 Activity outside the home 7 months | Study ID | Fairhall 2012 | Assessor | MR |
| Ref or Label |  | Aim | assignment to intervention (the 'intention-to-treat' effect) |  |  |
| Experimental |  | Comparator |  | Source | Journal article(s) |
| Outcome | Activity outside the home 7 months | Results |  | Weight |  |
| Domain | Signalling question | | | Response | Comments |
| Bias arising from the randomization process | 1.1 Was the allocation sequence random? | | | PY | An external data analyst created a computer-generated random numbers sequence using blocks and stratifications which was stored off site. Allocation was consealed until after baseline assessments. |
|  | 1.2 Was the allocation sequence concealed until participants were enrolled and assigned to interventions? | | | PY |  |
|  | 1.3 Did baseline differences between intervention groups suggest a problem with the randomization process? | | | N | No substancial baseline differences are present. |
|  | Risk of bias judgement | | | Low | An external data analyst created a computer-generated random numbers sequence using blocks and stratifications which was stored off site. Allocation was consealed until after baseline assessments.  No substancial baseline differences are present. |
| Bias due to deviations from intended interventions | 2.1.Were participants aware of their assigned intervention during the trial? | | | Y | Participants and treatment providers were aware of the group allocation. |
|  | 2.2.Were carers and people delivering the interventions aware of participants' assigned intervention during the trial? | | | Y |  |
|  | 2.3. If Y/PY/NI to 2.1 or 2.2: Were there deviations from the intended intervention that arose because of the experimental context? | | | PY | The control group got usual care after wanting to be enrolled in a study to improve outdoor mobility. The authors did not assess any deviations, but it is likely that the control group behaved differently due to not recieving any intervention at all. |
|  | 2.4 If Y/PY to 2.3: Were these deviations likely to have affected the outcome? | | | PY | It is likely that deviations (e.g. seeking additional care) could influence the outcomes. |
|  | 2.5. If Y/PY/NI to 2.4: Were these deviations from intended intervention balanced between groups? | | | PN | The deviations were likely not balanced between groups. |
|  | 2.6 Was an appropriate analysis used to estimate the effect of assignment to intervention? | | | PY | Intention to treat-analysis was used. |
|  | 2.7 If N/PN/NI to 2.6: Was there potential for a substantial impact (on the result) of the failure to analyse participants in the group to which they were randomized? | | | NA |  |
|  | Risk of bias judgement | | | High | Participants and treatment providers were aware of the group allocation.  The control group got usual care after wanting to be enrolled in a study to improve outdoor mobility. The authors did not assess any deviations, but it is likely that the control group behaved differently due to not recieving any intervention.  It is likely that deviations (e.g. seeking additional care) could influence the outcomes.  The deviations were likely not balanced between groups. Intention to treat-analysis was used. |
| Bias due to missing outcome data | 3.1 Were data for this outcome available for all, or nearly all, participants randomized? | | | PN | At 12 months the intervention group had 11% missingness of outcome data, and control group 10%. |
|  | 3.2 If N/PN/NI to 3.1: Is there evidence that result was not biased by missing outcome data? | | | PN | Authors do not report any attempt to explore any potential bias by missing outcome data. |
|  | 3.3 If N/PN to 3.2: Could missingness in the outcome depend on its true value? | | | PY | 12 vs 10 in the groups had missing outcome data due to "death", which is similar between the groups, but unclear how this influences the outcomes. Due to the similarities for reasons for missing data, the impact is judged not to be very large. |
|  | 3.4 If Y/PY/NI to 3.3: Is it likely that missingness in the outcome depended on its true value? | | | PN |  |
|  | Risk of bias judgement | | | Some concerns | At 12 months the intervention group had 11% missingness of outcome data, and control group 10%.  Authors do not report any attempt to explore any potential bias by missing outcome data.  12 vs 10 in the groups had missing outcome data due to "death", which is similar between the groups, but unclear how this influences the outcomes. Due to the similarities for reasons for missing data, the impact is judged not to be very large. |
| Bias in measurement of the outcome | 4.1 Was the method of measuring the outcome inappropriate? | | | PN | A validated intrument was used. |
|  | 4.2 Could measurement or ascertainment of the outcome have differed between intervention groups? | | | PN | Not reported, but unlikely that the outcome collection differered between groups, as the plan was to use blinded outcome assessors. |
|  | 4.3 Were outcome assessors aware of the intervention received by study participants? | | | Y | Participants rating their own outcomes were not blinded, and researchers collecting outcomes were planned to be unblinded but a majority (51%) became unblinded while supporting the participants with the outcome assessments. |
|  | 4.4 If Y/PY/NI to 4.3: Could assessment of the outcome have been influenced by knowledge of intervention received? | | | PY | It is likely that the assessment of the subjectively reported outcomes could differ between the groups due to the design of one group getting a lot of attention and focus on QoL and mobility, and the other did not get any attention at all. |
|  | 4.5 If Y/PY/NI to 4.4: Is it likely that assessment of the outcome was influenced by knowledge of intervention received? | | | PY |  |
|  | Risk of bias judgement | | | High | A validated intrument was used.  Not reported, but unlikely that the outcome collection differered between groups, as the plan was to use blinded outcome assessors.  Participants rating their own outcomes were not blinded, and researchers collecting outcomes were planned to be unblinded but a majority (51%) became unblinded while supporting the participants with the outcome assessments.  It is likely that the assessment of the subjectively reported outcomes could differ between the groups due to the design of one group getting a lot of attention and focus on QoL and mobility, and the other did not get any attention at all. |
| Bias in selection of the reported result | 5.1 Were the data that produced this result analysed in accordance with a pre-specified analysis plan that was finalized before unblinded outcome data were available for analysis? | | | Y | A pre-specified analysis plan was published in 2008. |
|  | 5.2 ... multiple eligible outcome measurements (e.g. scales, definitions, time points) within the outcome domain? | | | N | All outcomes are reported in the protocol. |
|  | 5.3 ... multiple eligible analyses of the data? | | | N | All analyses and justifications for adjustments are reported in the protocol. |
|  | Risk of bias judgement | | | Low | A pre-specified analysis plan was published in 2008.  All outcomes are reported in the protocol.  All analyses and justifications for adjustments are reported in the protocol. |
| Overall bias | Risk of bias judgement | | | High | Overall high risk of bias due to non-blinding of participants and personell and use of a usual care group without any attempt for attention control or assessing any deviations between interventions because of these choices. |
|  |  |  |  |  |  |
|  |  |  |  |  |  |
| Unique ID | Fairhall 2012 Engagement in everyday life activities 6 months | Study ID | Fairhall 2012 | Assessor | MR |
| Ref or Label |  | Aim | assignment to intervention (the 'intention-to-treat' effect) |  |  |
| Experimental |  | Comparator |  | Source | Journal article(s) |
| Outcome | Engagement in everydat activities 6 months | Results |  | Weight | 1 |
| Domain | Signalling question | | | Response | Comments |
| Bias arising from the randomization process | 1.1 Was the allocation sequence random? | | | PY | An external data analyst created a computer-generated random numbers sequence using blocks and stratifications which was stored off site. Allocation was consealed until after baseline assessments. |
|  | 1.2 Was the allocation sequence concealed until participants were enrolled and assigned to interventions? | | | PY |  |
|  | 1.3 Did baseline differences between intervention groups suggest a problem with the randomization process? | | | N | No substancial baseline differences are present. |
|  | Risk of bias judgement | | | Low | An external data analyst created a computer-generated random numbers sequence using blocks and stratifications which was stored off site. Allocation was consealed until after baseline assessments.  No substancial baseline differences are present. |
| Bias due to deviations from intended interventions | 2.1.Were participants aware of their assigned intervention during the trial? | | | Y | Participants and treatment providers were aware of the group allocation. |
|  | 2.2.Were carers and people delivering the interventions aware of participants' assigned intervention during the trial? | | | Y |  |
|  | 2.3. If Y/PY/NI to 2.1 or 2.2: Were there deviations from the intended intervention that arose because of the experimental context? | | | PY | The control group got usual care after wanting to be enrolled in a study to improve outdoor mobility. The authors did not assess any deviations, but it is likely that the control group behaved differently due to not recieving any intervention at all. |
|  | 2.4 If Y/PY to 2.3: Were these deviations likely to have affected the outcome? | | | PY | It is likely that deviations (e.g. seeking additional care) could influence the outcomes. |
|  | 2.5. If Y/PY/NI to 2.4: Were these deviations from intended intervention balanced between groups? | | | PN | The deviations were likely not balanced between groups. |
|  | 2.6 Was an appropriate analysis used to estimate the effect of assignment to intervention? | | | PY | Intention to treat-analysis was used. |
|  | 2.7 If N/PN/NI to 2.6: Was there potential for a substantial impact (on the result) of the failure to analyse participants in the group to which they were randomized? | | | NA |  |
|  | Risk of bias judgement | | | High | Participants and treatment providers were aware of the group allocation.  The control group got usual care after wanting to be enrolled in a study to improve outdoor mobility. The authors did not assess any deviations, but it is likely that the control group behaved differently due to not recieving any intervention.  It is likely that deviations (e.g. seeking additional care) could influence the outcomes.  The deviations were likely not balanced between groups. Intention to treat-analysis was used. |
| Bias due to missing outcome data | 3.1 Were data for this outcome available for all, or nearly all, participants randomized? | | | PN | At 3 months the intervention group had 9% missingness of outcome data, and control group 3%. |
|  | 3.2 If N/PN/NI to 3.1: Is there evidence that result was not biased by missing outcome data? | | | PN | Authors do not report any attempt to explore any potential bias by missing outcome data. |
|  | 3.3 If N/PN to 3.2: Could missingness in the outcome depend on its true value? | | | PY | 6 vs 1 in the groups had missing outcome data due to "death", and 3 vs 0 declined to be interviewed - indicating that outcomes was not missing on random at this timepoint, and could influence outcomes (e.g. people who died at 3 months can be assume have less outdoor mobility, QoL and participation which would influence any group-differences). But numbers of missing outcome data are quite small (9 vs 3%) which lessens the potential risk of bias for this domain. |
|  | 3.4 If Y/PY/NI to 3.3: Is it likely that missingness in the outcome depended on its true value? | | | PN |  |
|  | Risk of bias judgement | | | Some concerns | At 3 months the intervention group had 9% missingness of outcome data, and control group 3%.  Authors do not report any attempt to explore any potential bias by missing outcome data.  6 vs 1 in the groups had missing outcome data due to "death", and 3 vs 0 declined to be interviewed - indicating that outcomes was not missing on random at this timepoint, and could influence outcomes (e.g. people who died at 3 months can be assume have less outdoor mobility, QoL and participation which would influence any group-differences). But numbers of missing outcome data are quite small (9 vs 3%) which lessens the potential risk of bias for this domain. |
| Bias in measurement of the outcome | 4.1 Was the method of measuring the outcome inappropriate? | | | PN | A validated intrument was used. |
|  | 4.2 Could measurement or ascertainment of the outcome have differed between intervention groups? | | | PN | Not reported, but unlikely that the outcome collection differered between groups, as the plan was to use blinded outcome assessors. |
|  | 4.3 Were outcome assessors aware of the intervention received by study participants? | | | Y | Participants rating their own outcomes were not blinded, and researchers collecting outcomes were planned to be unblinded but a majority (51%) became unblinded while supporting the participants with the outcome assessments. |
|  | 4.4 If Y/PY/NI to 4.3: Could assessment of the outcome have been influenced by knowledge of intervention received? | | | PY | It is likely that the assessment of the subjectively reported outcomes could differ between the groups due to the design of one group getting a lot of attention and focus on QoL and mobility, and the other did not get any attention at all. |
|  | 4.5 If Y/PY/NI to 4.4: Is it likely that assessment of the outcome was influenced by knowledge of intervention received? | | | PY |  |
|  | Risk of bias judgement | | | High | A validated intrument was used.  Not reported, but unlikely that the outcome collection differered between groups, as the plan was to use blinded outcome assessors.  Participants rating their own outcomes were not blinded, and researchers collecting outcomes were planned to be unblinded but a majority (51%) became unblinded while supporting the participants with the outcome assessments.  It is likely that the assessment of the subjectively reported outcomes could differ between the groups due to the design of one group getting a lot of attention and focus on QoL and mobility, and the other did not get any attention at all. |
| Bias in selection of the reported result | 5.1 Were the data that produced this result analysed in accordance with a pre-specified analysis plan that was finalized before unblinded outcome data were available for analysis? | | | Y | A pre-specified analysis plan was published in 2008. |
|  | 5.2 ... multiple eligible outcome measurements (e.g. scales, definitions, time points) within the outcome domain? | | | N | All outcomes are reported in the protocol. |
|  | 5.3 ... multiple eligible analyses of the data? | | | N | All analyses and justifications for adjustments are reported in the protocol. |
|  | Risk of bias judgement | | | Low | A pre-specified analysis plan was published in 2008.  All outcomes are reported in the protocol.  All analyses and justifications for adjustments are reported in the protocol. |
| Overall bias | Risk of bias judgement | | | High | Overall high risk of bias due to non-blinding of participants and personell and use of a usual care group without any attempt for attention control or assessing any deviations between interventions because of these choices. |
|  |  |  |  |  |  |
|  |  |  |  |  |  |
| Unique ID | Fairhall 2012 Engagement in everyday life activities 7 months | Study ID | Fairhall 2012 | Assessor | MR |
| Ref or Label |  | Aim | assignment to intervention (the 'intention-to-treat' effect) |  |  |
| Experimental |  | Comparator |  | Source | Journal article(s) |
| Outcome | Engagement in everydat activities 7 months | Results |  | Weight | 1 |
| Domain | Signalling question | | | Response | Comments |
| Bias arising from the randomization process | 1.1 Was the allocation sequence random? | | | PY | An external data analyst created a computer-generated random numbers sequence using blocks and stratifications which was stored off site. Allocation was consealed until after baseline assessments. |
|  | 1.2 Was the allocation sequence concealed until participants were enrolled and assigned to interventions? | | | PY |  |
|  | 1.3 Did baseline differences between intervention groups suggest a problem with the randomization process? | | | N | No substancial baseline differences are present. |
|  | Risk of bias judgement | | | Low | An external data analyst created a computer-generated random numbers sequence using blocks and stratifications which was stored off site. Allocation was consealed until after baseline assessments.  No substancial baseline differences are present. |
| Bias due to deviations from intended interventions | 2.1.Were participants aware of their assigned intervention during the trial? | | | Y | Participants and treatment providers were aware of the group allocation. |
|  | 2.2.Were carers and people delivering the interventions aware of participants' assigned intervention during the trial? | | | Y |  |
|  | 2.3. If Y/PY/NI to 2.1 or 2.2: Were there deviations from the intended intervention that arose because of the experimental context? | | | PY | The control group got usual care after wanting to be enrolled in a study to improve outdoor mobility. The authors did not assess any deviations, but it is likely that the control group behaved differently due to not recieving any intervention at all. |
|  | 2.4 If Y/PY to 2.3: Were these deviations likely to have affected the outcome? | | | PY | It is likely that deviations (e.g. seeking additional care) could influence the outcomes. |
|  | 2.5. If Y/PY/NI to 2.4: Were these deviations from intended intervention balanced between groups? | | | PN | The deviations were likely not balanced between groups. |
|  | 2.6 Was an appropriate analysis used to estimate the effect of assignment to intervention? | | | PY | Intention to treat-analysis was used. |
|  | 2.7 If N/PN/NI to 2.6: Was there potential for a substantial impact (on the result) of the failure to analyse participants in the group to which they were randomized? | | | NA |  |
|  | Risk of bias judgement | | | High | Participants and treatment providers were aware of the group allocation.  The control group got usual care after wanting to be enrolled in a study to improve outdoor mobility. The authors did not assess any deviations, but it is likely that the control group behaved differently due to not recieving any intervention.  It is likely that deviations (e.g. seeking additional care) could influence the outcomes.  The deviations were likely not balanced between groups. Intention to treat-analysis was used. |
| Bias due to missing outcome data | 3.1 Were data for this outcome available for all, or nearly all, participants randomized? | | | PN | At 12 months the intervention group had 11% missingness of outcome data, and control group 10%. |
|  | 3.2 If N/PN/NI to 3.1: Is there evidence that result was not biased by missing outcome data? | | | PN | Authors do not report any attempt to explore any potential bias by missing outcome data. |
|  | 3.3 If N/PN to 3.2: Could missingness in the outcome depend on its true value? | | | PY | 12 vs 10 in the groups had missing outcome data due to "death", which is similar between the groups, but unclear how this influences the outcomes. Due to the similarities for reasons for missing data, the impact is judged not to be very large. |
|  | 3.4 If Y/PY/NI to 3.3: Is it likely that missingness in the outcome depended on its true value? | | | PN |  |
|  | Risk of bias judgement | | | Some concerns | At 12 months the intervention group had 11% missingness of outcome data, and control group 10%.  Authors do not report any attempt to explore any potential bias by missing outcome data.  12 vs 10 in the groups had missing outcome data due to "death", which is similar between the groups, but unclear how this influences the outcomes. Due to the similarities for reasons for missing data, the impact is judged not to be very large. |
| Bias in measurement of the outcome | 4.1 Was the method of measuring the outcome inappropriate? | | | PN | A validated intrument was used. |
|  | 4.2 Could measurement or ascertainment of the outcome have differed between intervention groups? | | | PN | Not reported, but unlikely that the outcome collection differered between groups, as the plan was to use blinded outcome assessors. |
|  | 4.3 Were outcome assessors aware of the intervention received by study participants? | | | Y | Participants rating their own outcomes were not blinded, and researchers collecting outcomes were planned to be unblinded but a majority (51%) became unblinded while supporting the participants with the outcome assessments. |
|  | 4.4 If Y/PY/NI to 4.3: Could assessment of the outcome have been influenced by knowledge of intervention received? | | | PY | It is likely that the assessment of the subjectively reported outcomes could differ between the groups due to the design of one group getting a lot of attention and focus on QoL and mobility, and the other did not get any attention at all. |
|  | 4.5 If Y/PY/NI to 4.4: Is it likely that assessment of the outcome was influenced by knowledge of intervention received? | | | PY |  |
|  | Risk of bias judgement | | | High | A validated intrument was used.  Not reported, but unlikely that the outcome collection differered between groups, as the plan was to use blinded outcome assessors.  Participants rating their own outcomes were not blinded, and researchers collecting outcomes were planned to be unblinded but a majority (51%) became unblinded while supporting the participants with the outcome assessments.  It is likely that the assessment of the subjectively reported outcomes could differ between the groups due to the design of one group getting a lot of attention and focus on QoL and mobility, and the other did not get any attention at all. |
| Bias in selection of the reported result | 5.1 Were the data that produced this result analysed in accordance with a pre-specified analysis plan that was finalized before unblinded outcome data were available for analysis? | | | Y | A pre-specified analysis plan was published in 2008. |
|  | 5.2 ... multiple eligible outcome measurements (e.g. scales, definitions, time points) within the outcome domain? | | | N | All outcomes are reported in the protocol. |
|  | 5.3 ... multiple eligible analyses of the data? | | | N | All analyses and justifications for adjustments are reported in the protocol. |
|  | Risk of bias judgement | | | Low | A pre-specified analysis plan was published in 2008.  All outcomes are reported in the protocol.  All analyses and justifications for adjustments are reported in the protocol. |
| Overall bias | Risk of bias judgement | | | High | Overall high risk of bias due to non-blinding of participants and personell and use of a usual care group without any attempt for attention control or assessing any deviations between interventions because of these choices. |
|  |  |  |  |  |  |
|  |  |  |  |  |  |
| Unique ID | Fairhall 2012 Participation 6 months | Study ID | Fairhall 2012 | Assessor | MR |
| Ref or Label |  | Aim | assignment to intervention (the 'intention-to-treat' effect) |  |  |
| Experimental |  | Comparator |  | Source | Journal article(s) |
| Outcome | Participation 6 months | Results |  | Weight | 1 |
| Domain | Signalling question | | | Response | Comments |
| Bias arising from the randomization process | 1.1 Was the allocation sequence random? | | | PY | An external data analyst created a computer-generated random numbers sequence using blocks and stratifications which was stored off site. Allocation was consealed until after baseline assessments. |
|  | 1.2 Was the allocation sequence concealed until participants were enrolled and assigned to interventions? | | | PY |  |
|  | 1.3 Did baseline differences between intervention groups suggest a problem with the randomization process? | | | N | No substancial baseline differences are present. |
|  | Risk of bias judgement | | | Low | An external data analyst created a computer-generated random numbers sequence using blocks and stratifications which was stored off site. Allocation was consealed until after baseline assessments.  No substancial baseline differences are present. |
| Bias due to deviations from intended interventions | 2.1.Were participants aware of their assigned intervention during the trial? | | | Y | Participants and treatment providers were aware of the group allocation. |
|  | 2.2.Were carers and people delivering the interventions aware of participants' assigned intervention during the trial? | | | Y |  |
|  | 2.3. If Y/PY/NI to 2.1 or 2.2: Were there deviations from the intended intervention that arose because of the experimental context? | | | PY | The control group got usual care after wanting to be enrolled in a study to improve outdoor mobility. The authors did not assess any deviations, but it is likely that the control group behaved differently due to not recieving any intervention at all. |
|  | 2.4 If Y/PY to 2.3: Were these deviations likely to have affected the outcome? | | | PY | It is likely that deviations (e.g. seeking additional care) could influence the outcomes. |
|  | 2.5. If Y/PY/NI to 2.4: Were these deviations from intended intervention balanced between groups? | | | PN | The deviations were likely not balanced between groups. |
|  | 2.6 Was an appropriate analysis used to estimate the effect of assignment to intervention? | | | PY | Intention to treat-analysis was used. |
|  | 2.7 If N/PN/NI to 2.6: Was there potential for a substantial impact (on the result) of the failure to analyse participants in the group to which they were randomized? | | | NA |  |
|  | Risk of bias judgement | | | High | Participants and treatment providers were aware of the group allocation.  The control group got usual care after wanting to be enrolled in a study to improve outdoor mobility. The authors did not assess any deviations, but it is likely that the control group behaved differently due to not recieving any intervention.  It is likely that deviations (e.g. seeking additional care) could influence the outcomes.  The deviations were likely not balanced between groups. Intention to treat-analysis was used. |
| Bias due to missing outcome data | 3.1 Were data for this outcome available for all, or nearly all, participants randomized? | | | PN | At 3 months the intervention group had 9% missingness of outcome data, and control group 3%. |
|  | 3.2 If N/PN/NI to 3.1: Is there evidence that result was not biased by missing outcome data? | | | PN | Authors do not report any attempt to explore any potential bias by missing outcome data. |
|  | 3.3 If N/PN to 3.2: Could missingness in the outcome depend on its true value? | | | PY | 6 vs 1 in the groups had missing outcome data due to "death", and 3 vs 0 declined to be interviewed - indicating that outcomes was not missing on random at this timepoint, and could influence outcomes (e.g. people who died at 3 months can be assume have less outdoor mobility, QoL and participation which would influence any group-differences). But numbers of missing outcome data are quite small (9 vs 3%) which lessens the potential risk of bias for this domain. |
|  | 3.4 If Y/PY/NI to 3.3: Is it likely that missingness in the outcome depended on its true value? | | | PN |  |
|  | Risk of bias judgement | | | Some concerns | At 3 months the intervention group had 9% missingness of outcome data, and control group 3%.  Authors do not report any attempt to explore any potential bias by missing outcome data.  6 vs 1 in the groups had missing outcome data due to "death", and 3 vs 0 declined to be interviewed - indicating that outcomes was not missing on random at this timepoint, and could influence outcomes (e.g. people who died at 3 months can be assume have less outdoor mobility, QoL and participation which would influence any group-differences). But numbers of missing outcome data are quite small (9 vs 3%) which lessens the potential risk of bias for this domain. |
| Bias in measurement of the outcome | 4.1 Was the method of measuring the outcome inappropriate? | | | PN | A validated intrument was used. |
|  | 4.2 Could measurement or ascertainment of the outcome have differed between intervention groups? | | | PN | Not reported, but unlikely that the outcome collection differered between groups, as the plan was to use blinded outcome assessors. |
|  | 4.3 Were outcome assessors aware of the intervention received by study participants? | | | Y | Participants rating their own outcomes were not blinded, and researchers collecting outcomes were planned to be unblinded but a majority (51%) became unblinded while supporting the participants with the outcome assessments. |
|  | 4.4 If Y/PY/NI to 4.3: Could assessment of the outcome have been influenced by knowledge of intervention received? | | | PY | It is likely that the assessment of the subjectively reported outcomes could differ between the groups due to the design of one group getting a lot of attention and focus on QoL and mobility, and the other did not get any attention at all. |
|  | 4.5 If Y/PY/NI to 4.4: Is it likely that assessment of the outcome was influenced by knowledge of intervention received? | | | PY |  |
|  | Risk of bias judgement | | | High | A validated intrument was used.  Not reported, but unlikely that the outcome collection differered between groups, as the plan was to use blinded outcome assessors.  Participants rating their own outcomes were not blinded, and researchers collecting outcomes were planned to be unblinded but a majority (51%) became unblinded while supporting the participants with the outcome assessments.  It is likely that the assessment of the subjectively reported outcomes could differ between the groups due to the design of one group getting a lot of attention and focus on QoL and mobility, and the other did not get any attention at all. |
| Bias in selection of the reported result | 5.1 Were the data that produced this result analysed in accordance with a pre-specified analysis plan that was finalized before unblinded outcome data were available for analysis? | | | Y | A pre-specified analysis plan was published in 2008. |
|  | 5.2 ... multiple eligible outcome measurements (e.g. scales, definitions, time points) within the outcome domain? | | | N | All outcomes are reported in the protocol. |
|  | 5.3 ... multiple eligible analyses of the data? | | | N | All analyses and justifications for adjustments are reported in the protocol. |
|  | Risk of bias judgement | | | Low | A pre-specified analysis plan was published in 2008.  All outcomes are reported in the protocol.  All analyses and justifications for adjustments are reported in the protocol. |
| Overall bias | Risk of bias judgement | | | High | Overall high risk of bias due to non-blinding of participants and personell and use of a usual care group without any attempt for attention control or assessing any deviations between interventions because of these choices. |
|  |  |  |  |  |  |
|  |  |  |  |  |  |
| Unique ID | Fairhall 2012 Participation 7 months | Study ID | Fairhall 2012 | Assessor | MR |
| Ref or Label |  | Aim | assignment to intervention (the 'intention-to-treat' effect) |  |  |
| Experimental |  | Comparator |  | Source | Journal article(s) |
| Outcome | Participation 7 months | Results |  | Weight | 1 |
| Domain | Signalling question | | | Response | Comments |
| Bias arising from the randomization process | 1.1 Was the allocation sequence random? | | | PY | An external data analyst created a computer-generated random numbers sequence using blocks and stratifications which was stored off site. Allocation was consealed until after baseline assessments. |
|  | 1.2 Was the allocation sequence concealed until participants were enrolled and assigned to interventions? | | | PY |  |
|  | 1.3 Did baseline differences between intervention groups suggest a problem with the randomization process? | | | N | No substancial baseline differences are present. |
|  | Risk of bias judgement | | | Low | An external data analyst created a computer-generated random numbers sequence using blocks and stratifications which was stored off site. Allocation was consealed until after baseline assessments.  No substancial baseline differences are present. |
| Bias due to deviations from intended interventions | 2.1.Were participants aware of their assigned intervention during the trial? | | | Y | Participants and treatment providers were aware of the group allocation. |
|  | 2.2.Were carers and people delivering the interventions aware of participants' assigned intervention during the trial? | | | Y |  |
|  | 2.3. If Y/PY/NI to 2.1 or 2.2: Were there deviations from the intended intervention that arose because of the experimental context? | | | PY | The control group got usual care after wanting to be enrolled in a study to improve outdoor mobility. The authors did not assess any deviations, but it is likely that the control group behaved differently due to not recieving any intervention at all. |
|  | 2.4 If Y/PY to 2.3: Were these deviations likely to have affected the outcome? | | | PY | It is likely that deviations (e.g. seeking additional care) could influence the outcomes. |
|  | 2.5. If Y/PY/NI to 2.4: Were these deviations from intended intervention balanced between groups? | | | PN | The deviations were likely not balanced between groups. |
|  | 2.6 Was an appropriate analysis used to estimate the effect of assignment to intervention? | | | PY | Intention to treat-analysis was used. |
|  | 2.7 If N/PN/NI to 2.6: Was there potential for a substantial impact (on the result) of the failure to analyse participants in the group to which they were randomized? | | | NA |  |
|  | Risk of bias judgement | | | High | Participants and treatment providers were aware of the group allocation.  The control group got usual care after wanting to be enrolled in a study to improve outdoor mobility. The authors did not assess any deviations, but it is likely that the control group behaved differently due to not recieving any intervention.  It is likely that deviations (e.g. seeking additional care) could influence the outcomes.  The deviations were likely not balanced between groups. Intention to treat-analysis was used. |
| Bias due to missing outcome data | 3.1 Were data for this outcome available for all, or nearly all, participants randomized? | | | PN | At 12 months the intervention group had 11% missingness of outcome data, and control group 10%. |
|  | 3.2 If N/PN/NI to 3.1: Is there evidence that result was not biased by missing outcome data? | | | PN | Authors do not report any attempt to explore any potential bias by missing outcome data. |
|  | 3.3 If N/PN to 3.2: Could missingness in the outcome depend on its true value? | | | PY | 12 vs 10 in the groups had missing outcome data due to "death", which is similar between the groups, but unclear how this influences the outcomes. Due to the similarities for reasons for missing data, the impact is judged not to be very large. |
|  | 3.4 If Y/PY/NI to 3.3: Is it likely that missingness in the outcome depended on its true value? | | | PN |  |
|  | Risk of bias judgement | | | Some concerns | At 12 months the intervention group had 11% missingness of outcome data, and control group 10%.  Authors do not report any attempt to explore any potential bias by missing outcome data.  12 vs 10 in the groups had missing outcome data due to "death", which is similar between the groups, but unclear how this influences the outcomes. Due to the similarities for reasons for missing data, the impact is judged not to be very large. |
| Bias in measurement of the outcome | 4.1 Was the method of measuring the outcome inappropriate? | | | PN | A validated intrument was used. |
[truncated: 59,396 more chars]
