# Supplementary material for: Interventions to improve outdoor mobility among people living with disabilities: A systematic review
Source: Campbell Syst Rev. 2024 Jun 14;20(2):e1407. doi: 10.1002/cl2.1407 (PMC11177337; doi:10.1002/cl2.1407)
Supplement: Supplementary file 2 — Supporting information. [file CL2-20-e1407-s001.docx]

Characteristics of studies

Characteristics of included studies [ordered by study ID]

Ada 2012

| ***Study characteristics*** | | |
| --- | --- | --- |
| Country |  | |
| Population |  | |
| Intervention |  | |
| Comparison |  | |
| Outcomes |  | |
| Notes |  | |
|  | | |
| **Item** | **Authors' judgement** | **Support for judgement** |
| Deviation from intended intervention Self-reported subjective outcomes | No |  |
| Deviation from intended intervention Adverse events | No |  |
| Missing outcome data Self-reporte subjective outcomes | Yes |  |
| Missing outcome data Adverse events | No |  |
| Measurement of the outcome Self-reported subjective outcomes | No |  |
| Measurement of the outcome Adverse events | No |  |
| Selection of reported results All risks | Yes |  |

Best 2016

| ***Study characteristics*** | | |
| --- | --- | --- |
| Country |  | |
| Population |  | |
| Intervention |  | |
| Comparison |  | |
| Outcomes |  | |
| Notes |  | |
|  | | |
| **Item** | **Authors' judgement** | **Support for judgement** |
| Randomisation process All risks | No |  |
| Deviation from intended intervention Activity outside the home | No |  |
| Missing outcome data Activity outside the home | Yes |  |
| Measurement of the outcome Activity outside the home | No |  |
| Selection of reported results All risks | Yes |  |

Brock 2011

| ***Study characteristics*** | |
| --- | --- |
| Country |  |
| Population |  |
| Intervention |  |
| Comparison |  |
| Outcomes |  |
| Notes |  |

DePaul 2015

| ***Study characteristics*** | | |
| --- | --- | --- |
| Country |  | |
| Population |  | |
| Intervention |  | |
| Comparison |  | |
| Outcomes |  | |
| Notes |  | |
|  | | |
| **Item** | **Authors' judgement** | **Support for judgement** |
| Randomisation process All risks | Yes |  |
| Deviation from intended intervention Activity outside the home | Yes |  |
| Deviation from intended intervention Self-reported subjective outcomes | Yes |  |
| Deviation from intended intervention Adverse events | Yes |  |
| Missing outcome data Activity outside the home | No |  |
| Missing outcome data Self-reporte subjective outcomes | No |  |
| Missing outcome data Adverse events | No |  |
| Measurement of the outcome Activity outside the home | Yes |  |
| Measurement of the outcome Self-reported subjective outcomes | Yes |  |
| Measurement of the outcome Adverse events | Yes |  |
| Selection of reported results All risks | Yes |  |

Fairhall 2012

| ***Study characteristics*** | | |
| --- | --- | --- |
| Country |  | |
| Population |  | |
| Intervention |  | |
| Comparison |  | |
| Outcomes |  | |
| Notes |  | |
|  | | |
| **Item** | **Authors' judgement** | **Support for judgement** |
| Randomisation process All risks | Yes |  |
| Deviation from intended intervention Activity outside the home | No |  |
| Deviation from intended intervention Self-reported subjective outcomes | No |  |
| Deviation from intended intervention Adverse events | No |  |
| Measurement of the outcome Activity outside the home | No |  |
| Measurement of the outcome Self-reported subjective outcomes | No |  |
| Measurement of the outcome Adverse events | No |  |
| Selection of reported results All risks | Yes |  |

Jeong 2016

| ***Study characteristics*** | |
| --- | --- |
| Country |  |
| Population |  |
| Intervention |  |
| Comparison |  |
| Outcomes |  |
| Notes |  |

Kim 2014

| ***Study characteristics*** | | |
| --- | --- | --- |
| Country |  | |
| Population |  | |
| Intervention |  | |
| Comparison |  | |
| Outcomes |  | |
| Notes |  | |
|  | | |
| **Item** | **Authors' judgement** | **Support for judgement** |
| Deviation from intended intervention Self-reported subjective outcomes | No |  |
| Missing outcome data Self-reporte subjective outcomes | No |  |
| Measurement of the outcome Self-reported subjective outcomes | No |  |

Kim 2016

| ***Study characteristics*** | |
| --- | --- |
| Country |  |
| Population |  |
| Intervention |  |
| Comparison |  |
| Outcomes |  |
| Notes |  |

Logan 2004

| ***Study characteristics*** | | |
| --- | --- | --- |
| Country |  | |
| Population |  | |
| Intervention |  | |
| Comparison |  | |
| Outcomes |  | |
| Notes |  | |
|  | | |
| **Item** | **Authors' judgement** | **Support for judgement** |
| Missing outcome data Activity outside the home | No |  |
| Missing outcome data Self-reporte subjective outcomes | No |  |

Logan 2014

| ***Study characteristics*** | | |
| --- | --- | --- |
| Country |  | |
| Population |  | |
| Intervention |  | |
| Comparison |  | |
| Outcomes |  | |
| Notes |  | |
|  | | |
| **Item** | **Authors' judgement** | **Support for judgement** |
| Missing outcome data Activity outside the home | No |  |
| Missing outcome data Adverse events | No |  |
| Measurement of the outcome Activity outside the home | No |  |
| Selection of reported results All risks | Yes |  |

Lord 2008

| ***Study characteristics*** | | |
| --- | --- | --- |
| Country |  | |
| Population |  | |
| Intervention |  | |
| Comparison |  | |
| Outcomes |  | |
| Notes |  | |
|  | | |
| **Item** | **Authors' judgement** | **Support for judgement** |
| Randomisation process All risks | Yes |  |
| Missing outcome data Activity outside the home | No |  |
| Missing outcome data Self-reporte subjective outcomes | No |  |
| Missing outcome data Adverse events | No |  |
| Selection of reported results All risks | No |  |

Magaziner 2019

| ***Study characteristics*** | | |
| --- | --- | --- |
| Country |  | |
| Population |  | |
| Intervention |  | |
| Comparison |  | |
| Outcomes |  | |
| Notes |  | |
|  | | |
| **Item** | **Authors' judgement** | **Support for judgement** |
| Randomisation process All risks | Yes |  |
| Missing outcome data Adverse events | No |  |
| Selection of reported results All risks | Yes |  |

Mendoza 2015

| ***Study characteristics*** | |
| --- | --- |
| Country |  |
| Population |  |
| Intervention |  |
| Comparison |  |
| Outcomes |  |
| Notes |  |

Miller 2019

| ***Study characteristics*** | | |
| --- | --- | --- |
| Country |  | |
| Population |  | |
| Intervention |  | |
| Comparison |  | |
| Outcomes |  | |
| Notes |  | |
|  | | |
| **Item** | **Authors' judgement** | **Support for judgement** |
| Randomisation process All risks | No |  |
| Deviation from intended intervention Activity outside the home | Yes |  |
| Deviation from intended intervention Self-reported subjective outcomes | Yes |  |
| Measurement of the outcome Activity outside the home | Yes |  |
| Measurement of the outcome Self-reported subjective outcomes | Yes |  |
| Selection of reported results All risks | Yes |  |

Mänty 2009

| ***Study characteristics*** | |
| --- | --- |
| Country |  |
| Population |  |
| Intervention |  |
| Comparison |  |
| Outcomes |  |
| Notes |  |

Park 2011

| ***Study characteristics*** | |
| --- | --- |
| Country |  |
| Population |  |
| Intervention |  |
| Comparison |  |
| Outcomes |  |
| Notes |  |

Park 2016

| ***Study characteristics*** | |
| --- | --- |
| Country |  |
| Population |  |
| Intervention |  |
| Comparison |  |
| Outcomes |  |
| Notes |  |

Rantanen 2015

| ***Study characteristics*** | | |
| --- | --- | --- |
| Country |  | |
| Population |  | |
| Intervention |  | |
| Comparison |  | |
| Outcomes |  | |
| Notes |  | |
|  | | |
| **Item** | **Authors' judgement** | **Support for judgement** |
| Randomisation process All risks | No |  |
| Measurement of the outcome Self-reported subjective outcomes | No |  |

Turunen 2020

| ***Study characteristics*** | | |
| --- | --- | --- |
| Country |  | |
| Population |  | |
| Intervention |  | |
| Comparison |  | |
| Outcomes |  | |
| Notes |  | |
|  | | |
| **Item** | **Authors' judgement** | **Support for judgement** |
| Randomisation process All risks | Yes |  |
| Deviation from intended intervention Self-reported subjective outcomes | No |  |
| Deviation from intended intervention Adverse events | No |  |
| Missing outcome data Self-reporte subjective outcomes | No |  |
| Missing outcome data Adverse events | No |  |
| Measurement of the outcome Self-reported subjective outcomes | No |  |
| Measurement of the outcome Adverse events | No |  |
| Selection of reported results All risks | No |  |

Ullrich 2021

| ***Study characteristics*** | | |
| --- | --- | --- |
| Country |  | |
| Population |  | |
| Intervention |  | |
| Comparison |  | |
| Outcomes |  | |
| Notes |  | |
|  | | |
| **Item** | **Authors' judgement** | **Support for judgement** |
| Deviation from intended intervention Activity outside the home | Yes |  |
| Deviation from intended intervention Self-reported subjective outcomes | Yes |  |
| Missing outcome data Activity outside the home | No |  |
| Missing outcome data Self-reporte subjective outcomes | No |  |
| Measurement of the outcome Activity outside the home | Yes |  |
| Measurement of the outcome Self-reported subjective outcomes | Yes |  |
| Selection of reported results All risks | Yes |  |

Wang 2021

| ***Study characteristics*** | |
| --- | --- |
| Country |  |
| Population |  |
| Intervention |  |
| Comparison |  |
| Outcomes |  |
| Notes |  |

Yang 2008

| ***Study characteristics*** | |
| --- | --- |
| Country |  |
| Population |  |
| Intervention |  |
| Comparison |  |
| Outcomes |  |
| Notes |  |

Characteristics of excluded studies [ordered by study ID]

| Study | Reason for exclusion |
| --- | --- |
| Aalto 2011 |  |
| Abasıyanık 2020 |  |
| Abe 2001 |  |
| Acton 2016 |  |
| Actrn 2005 |  |
| Actrn 2006 |  |
| Actrn 2008 |  |
| Actrn 2017 |  |
| Actrn 2017a |  |
| Actrn 2018 |  |
| Actrn 2019 |  |
| Actrn 2019a |  |
| Actrn 2019b |  |
| Actrn 2020 |  |
| Actrn 2021 |  |
| Ada 2003 |  |
| Ada 2007a |  |
| Ada 2011 |  |
| Ada 2013 |  |
| Ades 2003 |  |
| Aftab 2020 |  |
| Aguiar 2018 |  |
| Aguiar 2020 |  |
| Ahmadi 2010 |  |
| Aitken 2021 |  |
| Ajiboye 2015 |  |
| Akin 2021 |  |
| Alabdulwahab 2015 |  |
| Alcobendas-Maestro 2012 |  |
| Alexeeva 2011 |  |
| Alfieri 2010 |  |
| Ali 2020 |  |
| Allen 1996 |  |
| Almuklass 2018 |  |
| Amatachaya 2021 |  |
| Ambrosini 2011 |  |
| Ambrosini 2020 |  |
| Ammendolia 2016 |  |
| Ammendolia 2017 |  |
| Ammendolia 2018 |  |
| Ammendolia 2019 |  |
| Ammendolia 2019a |  |
| Anderson 2004 |  |
| Aquilani 2019 |  |
| Arbillaga-Etxarri 2018 |  |
| Aries 2021 |  |
| Avelino 2018 |  |
| Avelino 2021 |  |
| Awad 2016 |  |
| Awosika 2020 |  |
| Aytekin 2012 |  |
| Bae 2019 |  |
| Baker 2008 |  |
| Baker 2010 |  |
| Baker 2010a |  |
| Ballemans 2012 |  |
| Bang 2013 |  |
| Bang 2014 |  |
| Bang 2016 |  |
| Bang 2016a |  |
| Bang 2016b |  |
| Bang 2017 |  |
| Barclay 2016 |  |
| Barclay 2018 |  |
| Bauer 2015 |  |
| Bearne 2019 |  |
| Behrman 2011 |  |
| Beijersbergen 2016 |  |
| Bennett 2007 |  |
| Berriozabalgoitia 2020 |  |
| Berriozabalgoitia 2021 |  |
| Best 2005 |  |
| Bethoux 2015 |  |
| Binder 2004 |  |
| Bisson 2007 |  |
| Bizovičar 2017 |  |
| Bondoc 2015 |  |
| Bosner 2012 |  |
| Botoseneanu 2017 |  |
| Boult 2001 |  |
| Bourque 2019 |  |
| Brach 2015 |  |
| Brach 2015a |  |
| Brach 2020 |  |
| Brach 2022 |  |
| Brandão 2018 |  |
| Brauer 2011 |  |
| Brauer 2014 |  |
| Brauer 2018 |  |
| Brauer 2022 |  |
| Braun 2019 |  |
| Breyer 2010 |  |
| Briken 2014 |  |
| Brosseau 2011 |  |
| Brouwer 2018 |  |
| Brown 2012 |  |
| Brown 2016 |  |
| Brustio 2018 |  |
| Buesing 2015 |  |
| Buhagiar 2013 |  |
| Buhagiar 2017 |  |
| Bulińska 2016 |  |
| Bunout 2001 |  |
| Busse 2016 |  |
| Byl 2015 |  |
| Cai 2021 |  |
| Caldow 2019 |  |
| Callahan 2016 |  |
| Callahan 2021 |  |
| Cameron 2013 |  |
| Canli 2020 |  |
| Cesari 2015 |  |
| Cezar 2021 |  |
| Chao 2011 |  |
| Chen 2021 |  |
| Clanchy 2016 |  |
| Clegg 2011 |  |
| Clegg 2014 |  |
| Collins 2009 |  |
| Collins 2010 |  |
| Collins 2018 |  |
| Combs-Miller 2014 |  |
| Corbett 2018 |  |
| Courtney 2011 |  |
| Courtney 2012 |  |
| Coxon 2017 |  |
| Cunningham 2020 |  |
| Dautel 2019 |  |
| Dawes 2011 |  |
| de Rooij 2019 |  |
| de Rooij 2021 |  |
| Dean 2009 |  |
| Dean 2010 |  |
| Dean 2011 |  |
| Dean 2012 |  |
| Dean 2012a |  |
| Demers 2016 |  |
| DePaul 2011 |  |
| DePaul 2011a |  |
| DePaul 2012 |  |
| Dobkin 2003 |  |
| Dobkin 2010 |  |
| Dohrn 2017 |  |
| Donatoni 2022 |  |
| Dorresteijn 2011 |  |
| Dorsch 2015 |  |
| Drks 2020 |  |
| Drks 2020a |  |
| Drks 2021a |  |
| Duru 2010 |  |
| Duvall 2011 |  |
| Duvall 2011a |  |
| Duvall 2012 |  |
| Duvall 2013 |  |
| Edgren 2015 |  |
| Esmaeili 2020 |  |
| Ferney 2009 |  |
| Fielding 2011 |  |
| Fielding 2017 |  |
| Figueiredo 2013 |  |
| Figueiredo 2017 |  |
| Fisher 2004 |  |
| Froelicher 1994 |  |
| Fukui 2019 |  |
| Galea 2019 |  |
| Galvin 2011 |  |
| Gardner 2001 |  |
| Gardner 2011 |  |
| Giesbrecht 2013 |  |
| Giesbrecht 2016 |  |
| Giesbrecht 2017 |  |
| Giesbrecht 2019 |  |
| Gilboa 2019 |  |
| Gill 2004 |  |
| Gitlin 2006 |  |
| Gleeson 2014 |  |
| Gleeson 2015 |  |
| Gleeson 2017 |  |
| Gray 2018 |  |
| Green 2002 |  |
| Green 2004 |  |
| Greiman 2022 |  |
| Halsne 2020 |  |
| Harrington 2010 |  |
| Helbostad 2004 |  |
| Hendriks 2008 |  |
| Hitzig 2013 |  |
| Hoenig 2007 |  |
| Hornby 2008 |  |
| Hornby 2016 |  |
| Horne 2010 |  |
| Hsu 2021 |  |
| Isrctn 2015a |  |
| Isrctn 2016b |  |
| Isrctn 2018 |  |
| Jones 2014 |  |
| Jones 2014a |  |
| Jprn 2016 |  |
| Jprn 2018 |  |
| Jprn 2018a |  |
| Jprn 2018b |  |
| Jprn 2018c |  |
| Jprn 2019 |  |
| Jprn 2019a |  |
| Jprn 2019b |  |
| King 2017 |  |
| Kirby 2015 |  |
| Klochkov 2018 |  |
| Krishnamurthi 2020 |  |
| Kumaran 2016 |  |
| Liddle 2014 |  |
| Lightbody 2002 |  |
| Lihavainen 2012 |  |
| Lin 2020 |  |
| Liu 2014 |  |
| Liu 2021 |  |
| Logan 2003 |  |
| Mackey 2019 |  |
| MacPhee 2004 |  |
| Mansfield 2013 |  |
| Mansfield 2015 |  |
| Marottoli 2007 |  |
| Mayo 2015 |  |
| Mazer 2015 |  |
| McDermott 2004 |  |
| McDermott 2018 |  |
| Melin 1992 |  |
| Melin 1995 |  |
| Miller 2014 |  |
| Mirelman 2009 |  |
| Mirelman 2013 |  |
| Mishra 2015 |  |
| Molteni 2021 |  |
| Mouri 2018 |  |
| Mudge 2009 |  |
| Mulder 2022 |  |
| Munneke 2012 |  |
| Murphy 2009 |  |
| Murphy 2011 |  |
| Nadeau 2013 |  |
| Nagai 2018 |  |
| Nct 2005 |  |
| Nct 2012 |  |
| Nct 2012a |  |
| Nct 2012b |  |
| Nct 2012c |  |
| Nct 2012d |  |
| Nct 2013a |  |
| Nct 2013b |  |
| Nct 2014 |  |
| Nct 2014a |  |
| Nct 2015a |  |
| Nct 2015b |  |
| Nct 2015c |  |
| Nct 2016 |  |
| Nct 2016a |  |
| Nct 2017a |  |
| Nct 2017b |  |
| Nct 2018b |  |
| Nct 2018c |  |
| Nct 2018d |  |
| Nct 2018e |  |
| Nct 2018f |  |
| Nct 2019 |  |
| Nct 2019a |  |
| Nct 2019b |  |
| Nct 2019c |  |
| Nct 2020b |  |
| Nct 2020c |  |
| Nct 2021 |  |
| Nct 2021a |  |
| Nct 2021b |  |
| Nct 2021c |  |
| Nct 2022 |  |
| Nikaido 2022 |  |
| Oliveira 2019 |  |
| Pactr 2019 |  |
| Pahor 2014 |  |
| Pang 2018 |  |
| Patch 2021 |  |
| Portegijs 2008 |  |
| Portegijs 2013 |  |
| Ramakrishna 2021 |  |
| Rantanen 2019 |  |
| Rantanen 2020 |  |
| Rawson 2020 |  |
| Sakakibara 2013 |  |
| Sethy 2021 |  |
| Shaughnessy 2012 |  |
| Sherrington 2016 |  |
| Sherrington 2020 |  |
| Shimokihara 2021 |  |
| Siltanen 2020 |  |
| Sipilä 2018 |  |
| Steadman 2003 |  |
| Sullivan 2011 |  |
| Takeda 2022 |  |
| Taylor 2021 |  |
| Timmermans 2016 |  |
| Tsai 2018 |  |
| Uemura 2021 |  |
| van de Port 2012 |  |
| van den 2016 |  |
| van der Kolk 2014 |  |
| VanSwearingen 2011 |  |
| Vincent 2020 |  |
| Vloothuis 2015 |  |
| Vloothuis 2019 |  |
| Vluggen 2021 |  |
| von Bonsdorff 2008 |  |
